# Supplementary material for: Ti(Oi-Pr)4-promoted photoenolization Diels–Alder reaction to construct polycyclic rings and its synthetic applications
Source: Nat Commun. 2017 Sep 20;8:622. doi: 10.1038/s41467-017-00440-8 (PMC5607006; doi:10.1038/s41467-017-00440-8)
Supplement: Supplementary file 1 — Supplementary Information [file 41467_2017_440_MOESM1_ESM.pdf]

Description of Supplementary Files

File Name: Supplementary Information
Description: Supplementary Figures, Supplementary Tables, Supplementary Discussion, Supplementary Methods and Supplementary References

File Name: Peer Review File

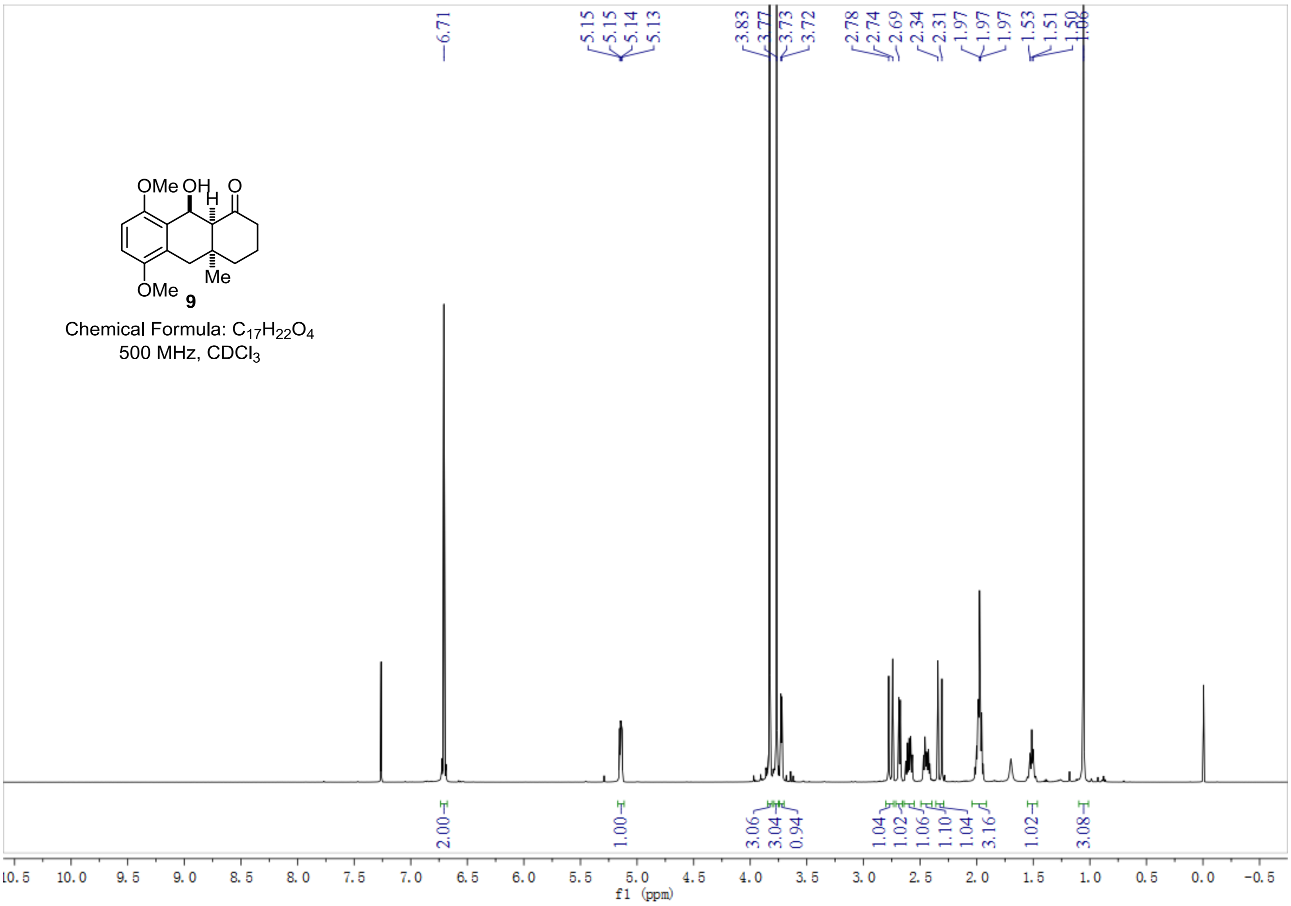

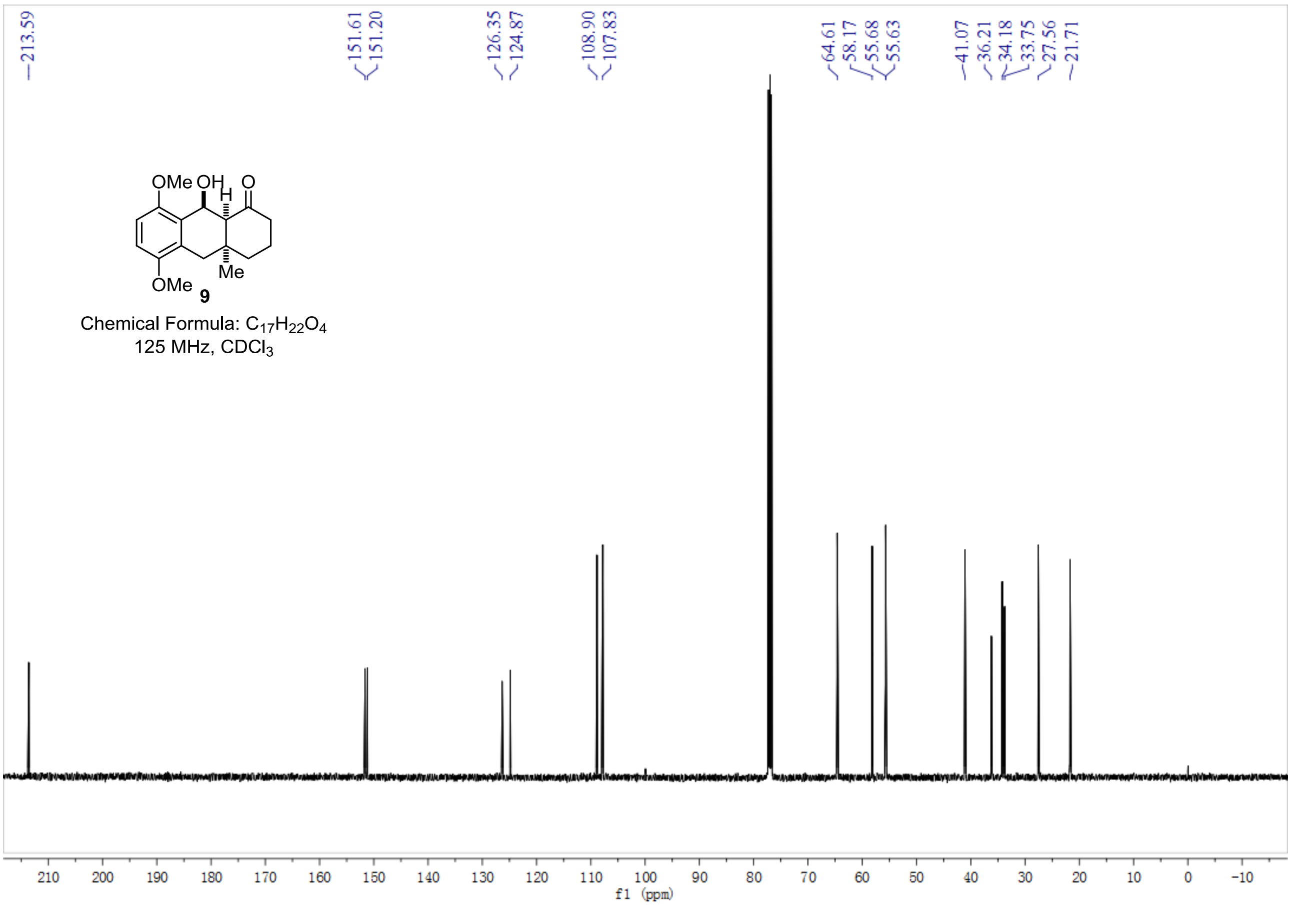

Supplementary Figure 1. ^{1}**H and** ^{13}**C NMR spectra for 9.**

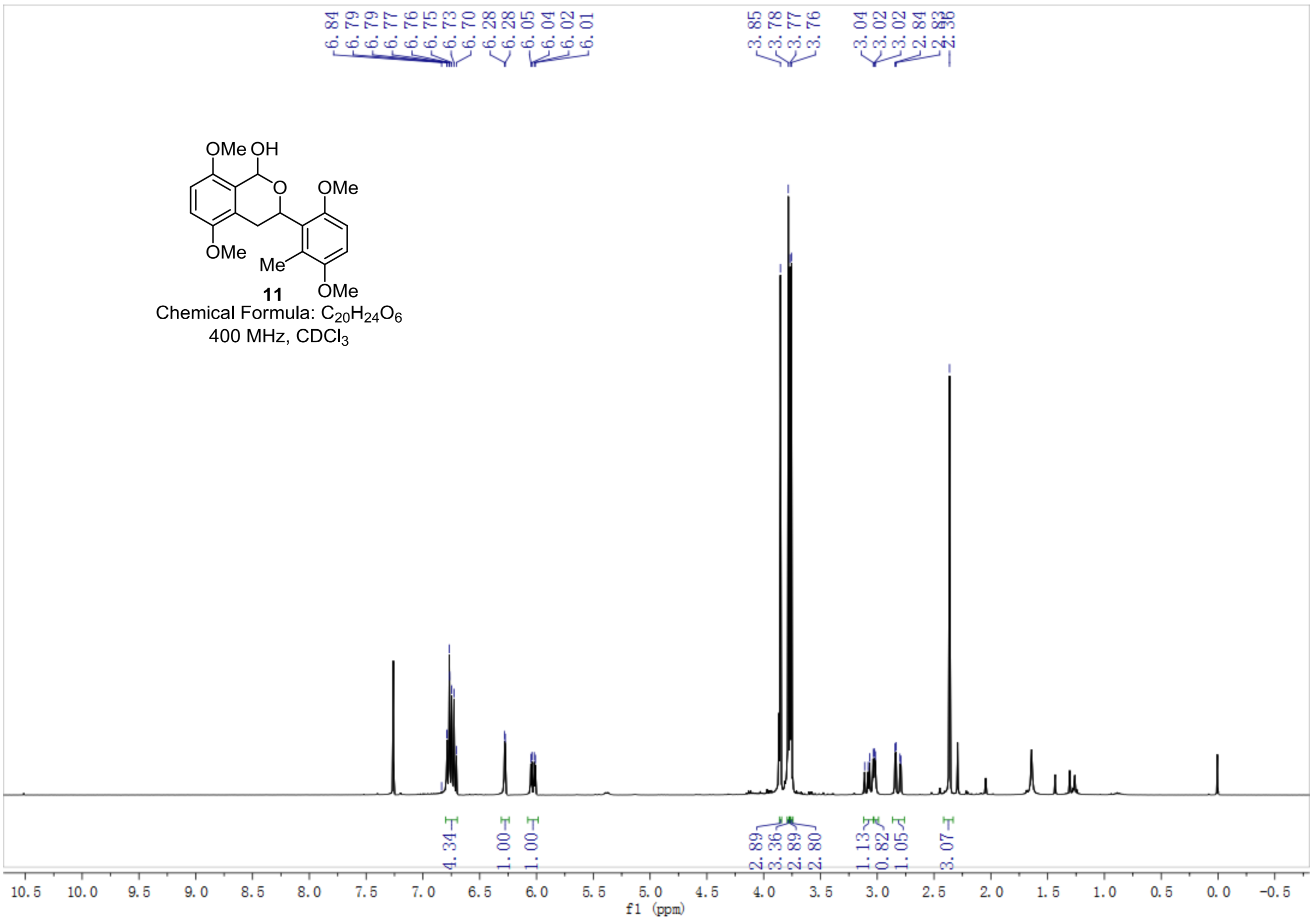

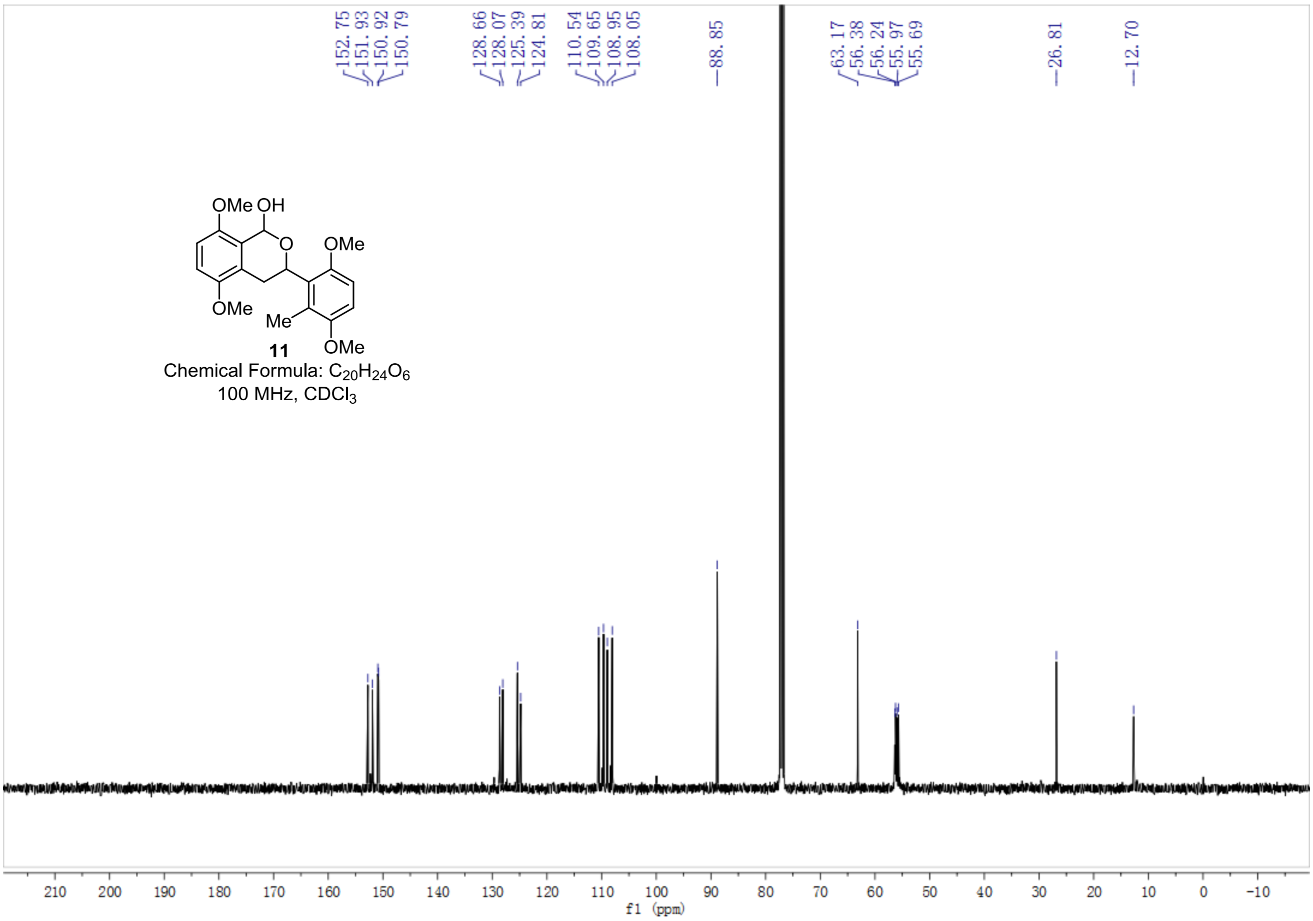

Supplementary Figure 2. ^{1}H and ^{13}C NMR spectra for 11.

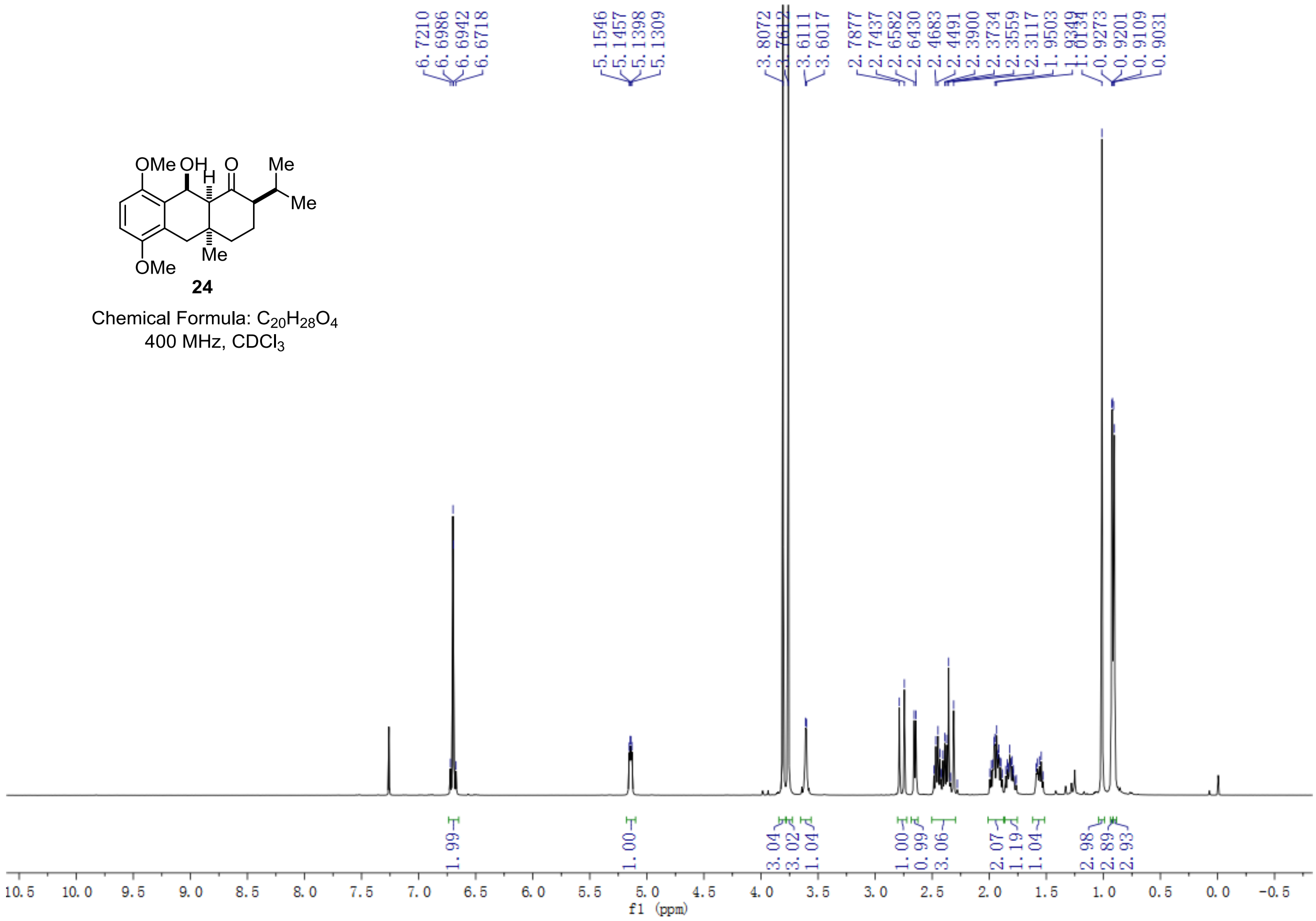

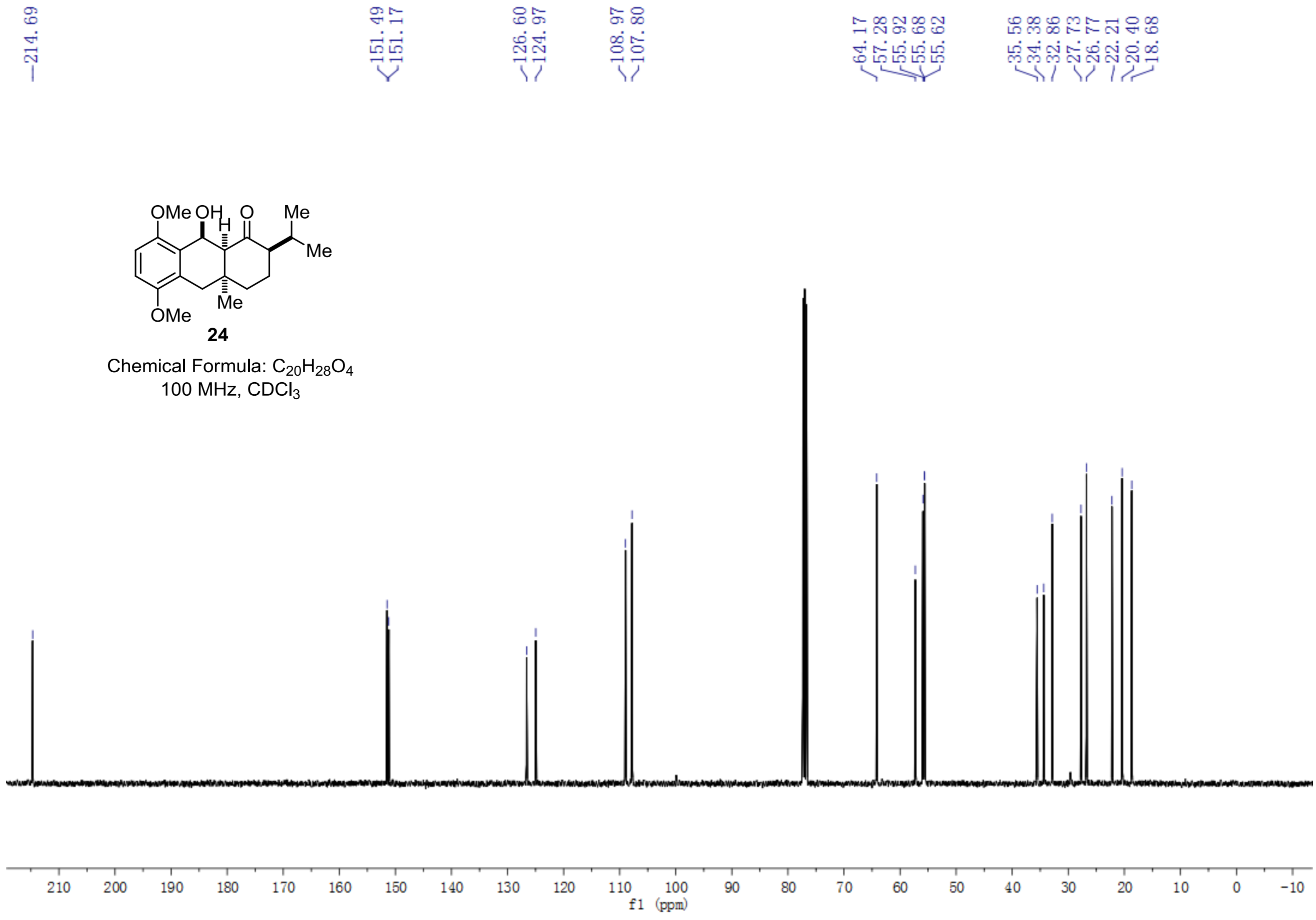

Supplementary Figure 3. ^{1}H **and** ^{13}C **NMR spectra for 24.**

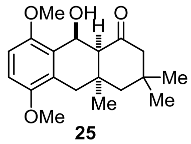

25

Chemical Formula: $C_{19}H_{26}O_4$

400 MHz, $CDCl_3$

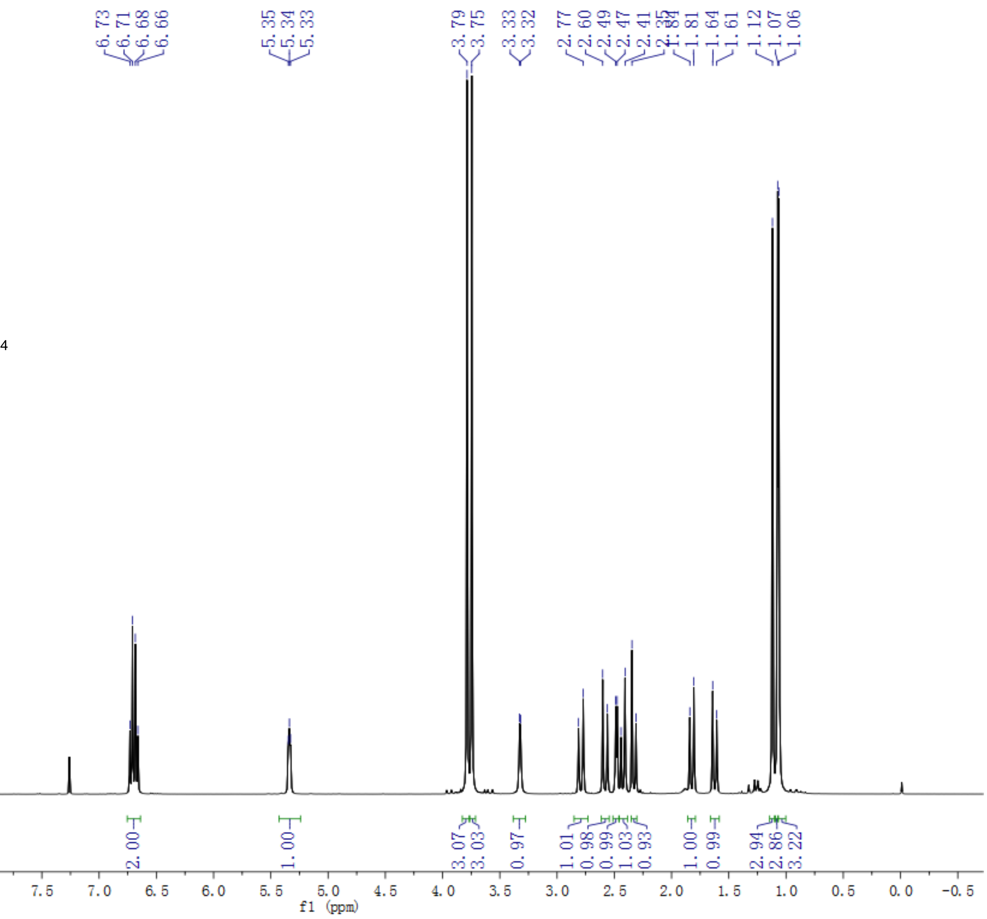

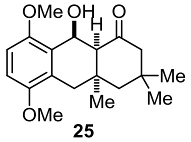

25

Chemical Formula: $C_{19}H_{26}O_4$

100 MHz, $CDCl_3$

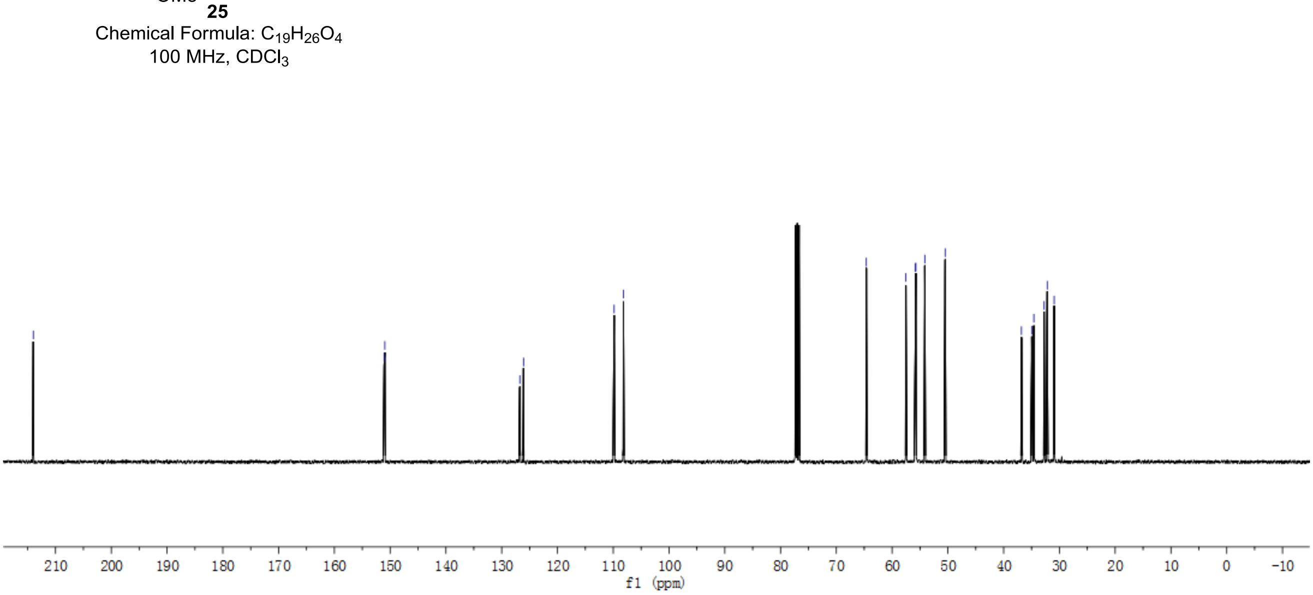

Supplementary Figure 4. ^{1}H and ^{13}C NMR spectra for 25.

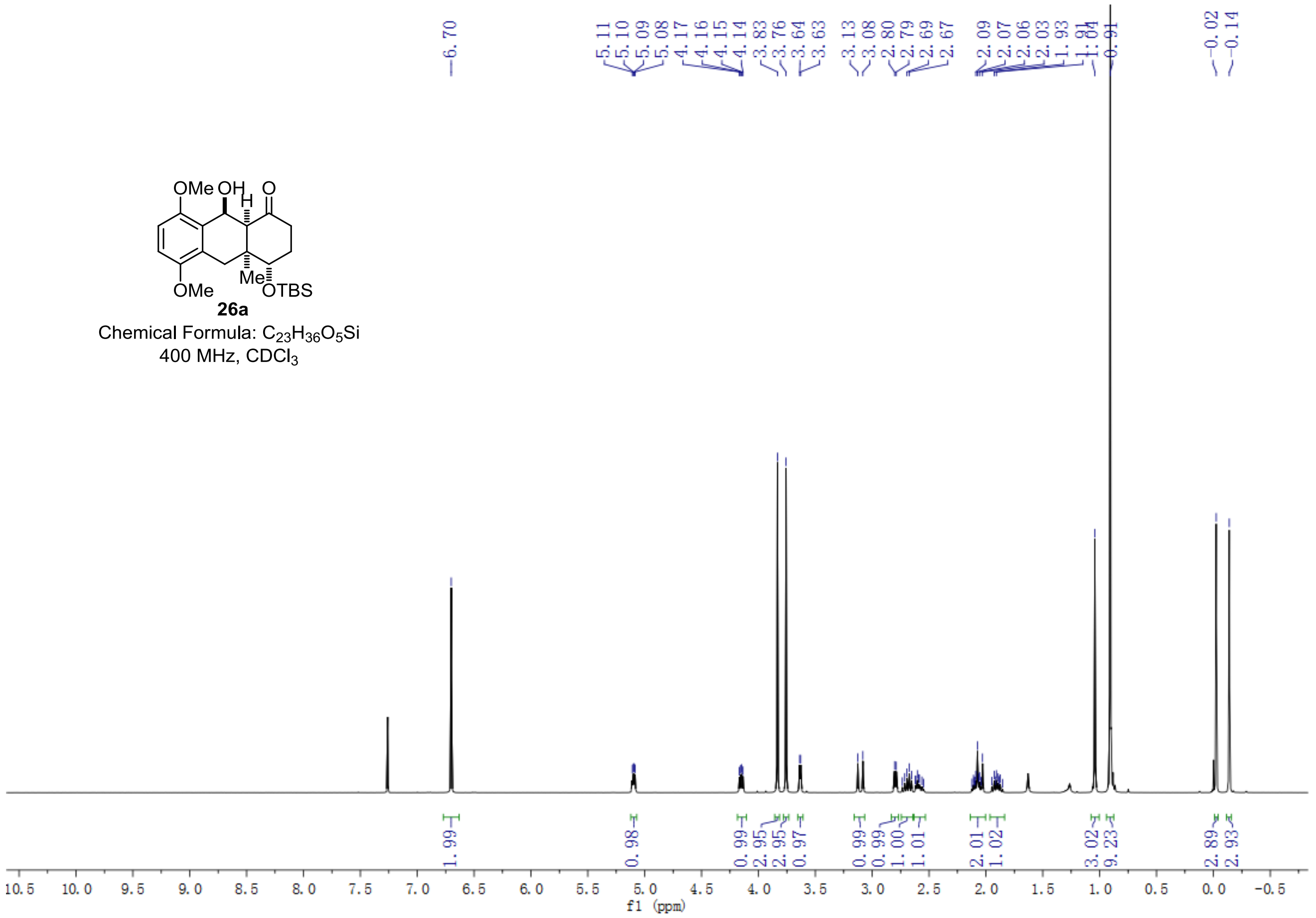

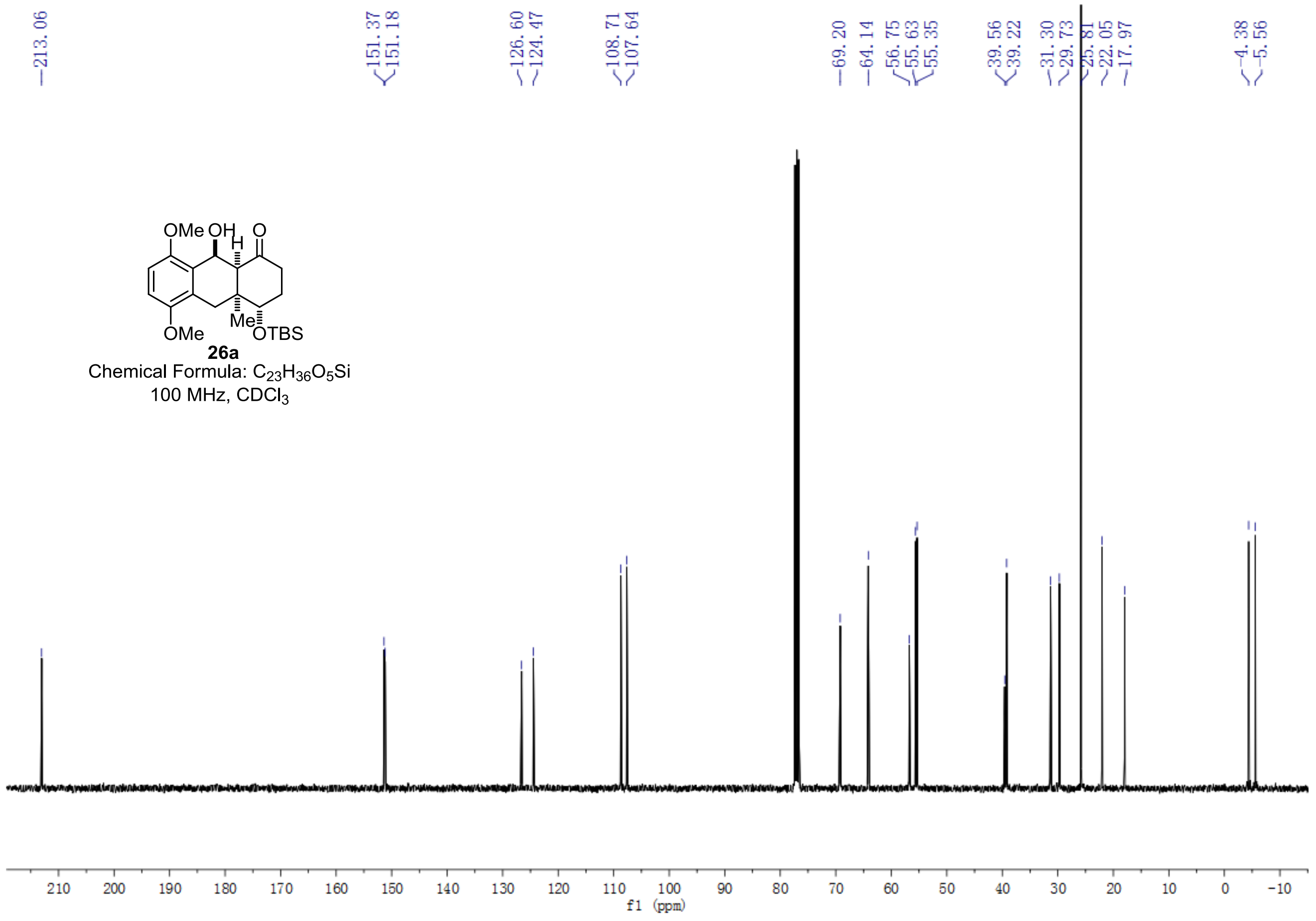

Supplementary Figure 5.^{1}H and ^{13}C NMR spectra for 26a.

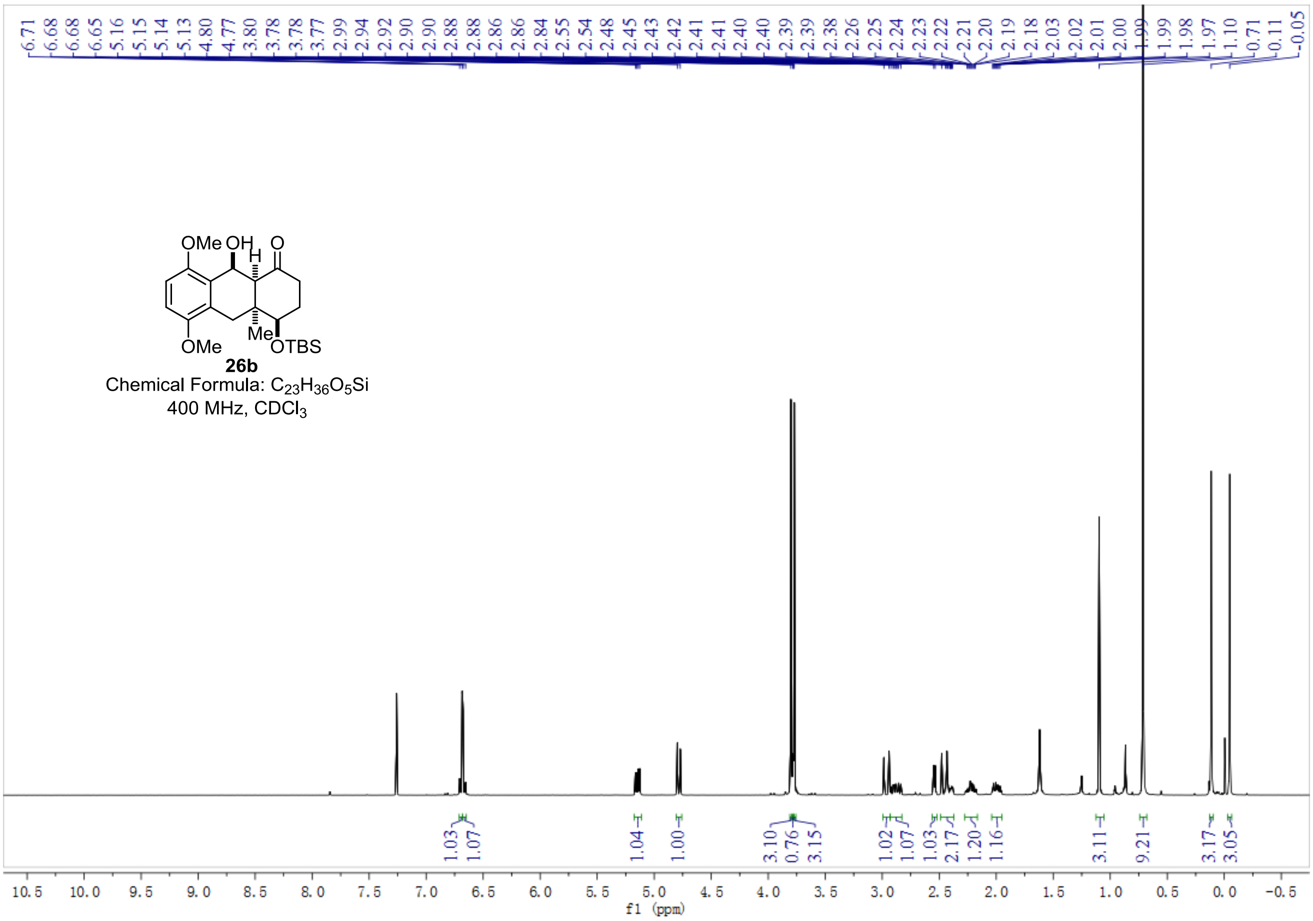

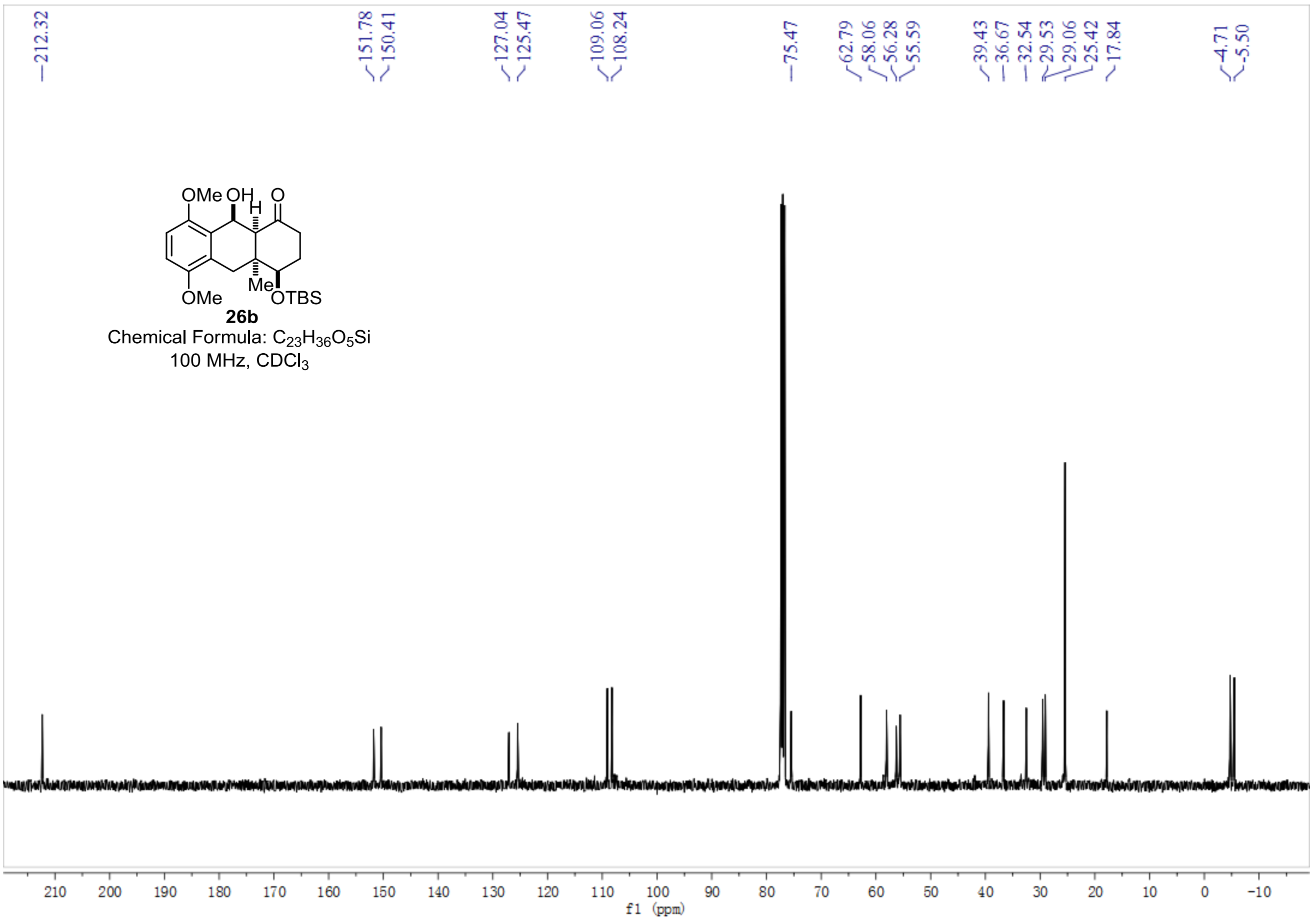

Supplementary Figure 6.^{1}H and ^{13}C NMR spectra for 26b.

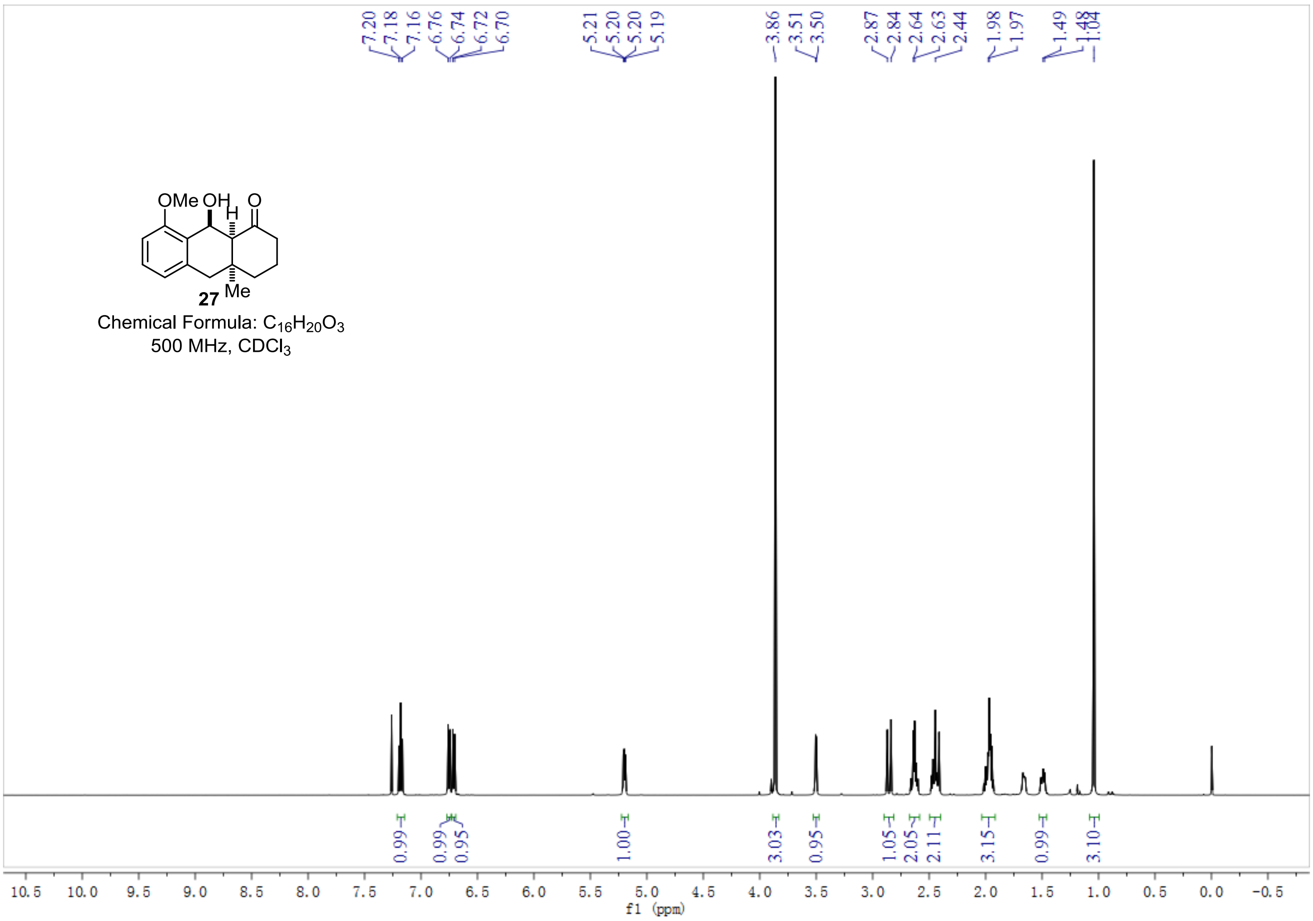

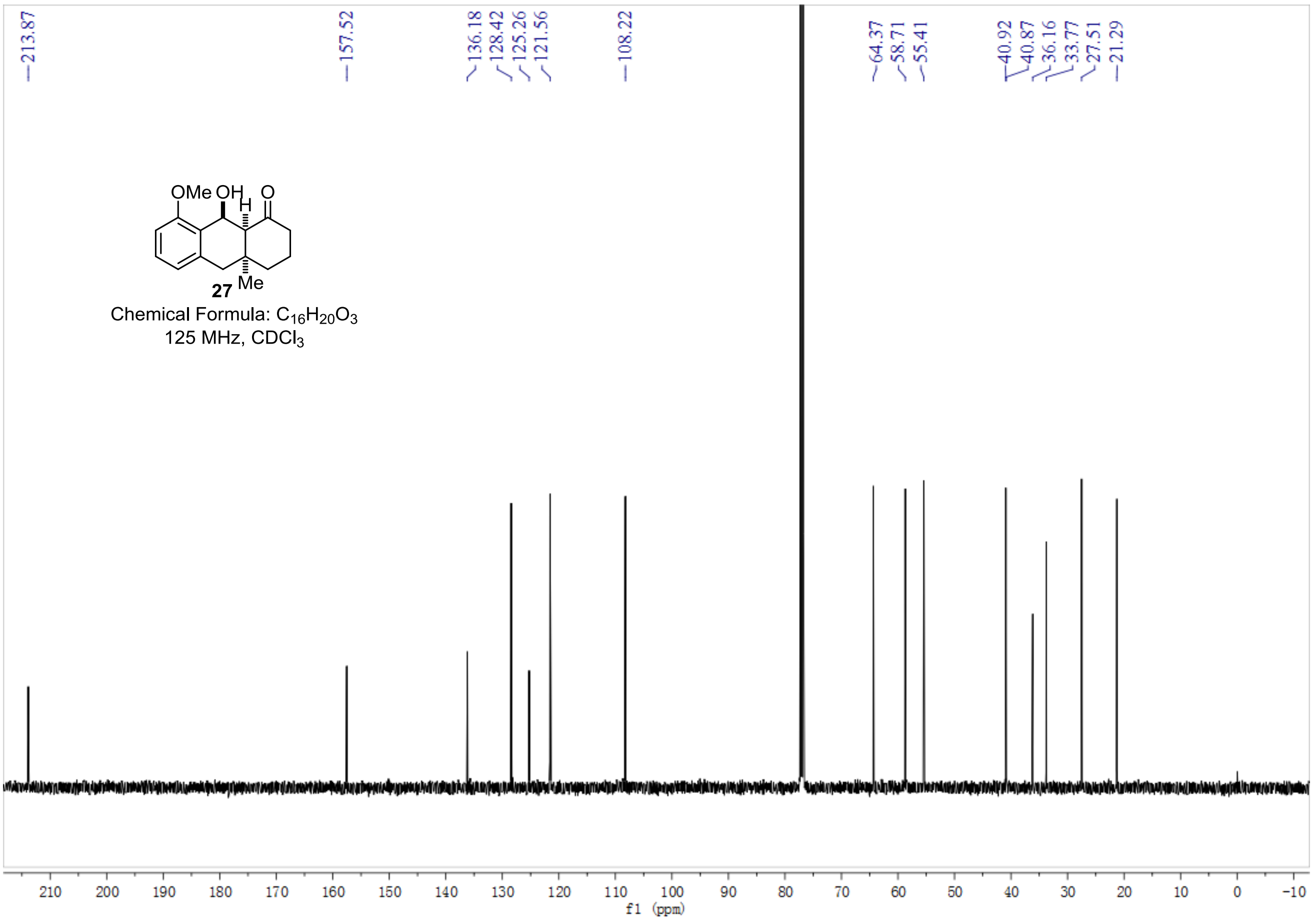

Supplementary Figure 7. ^{1}H and ^{13}C NMR spectra for 27.

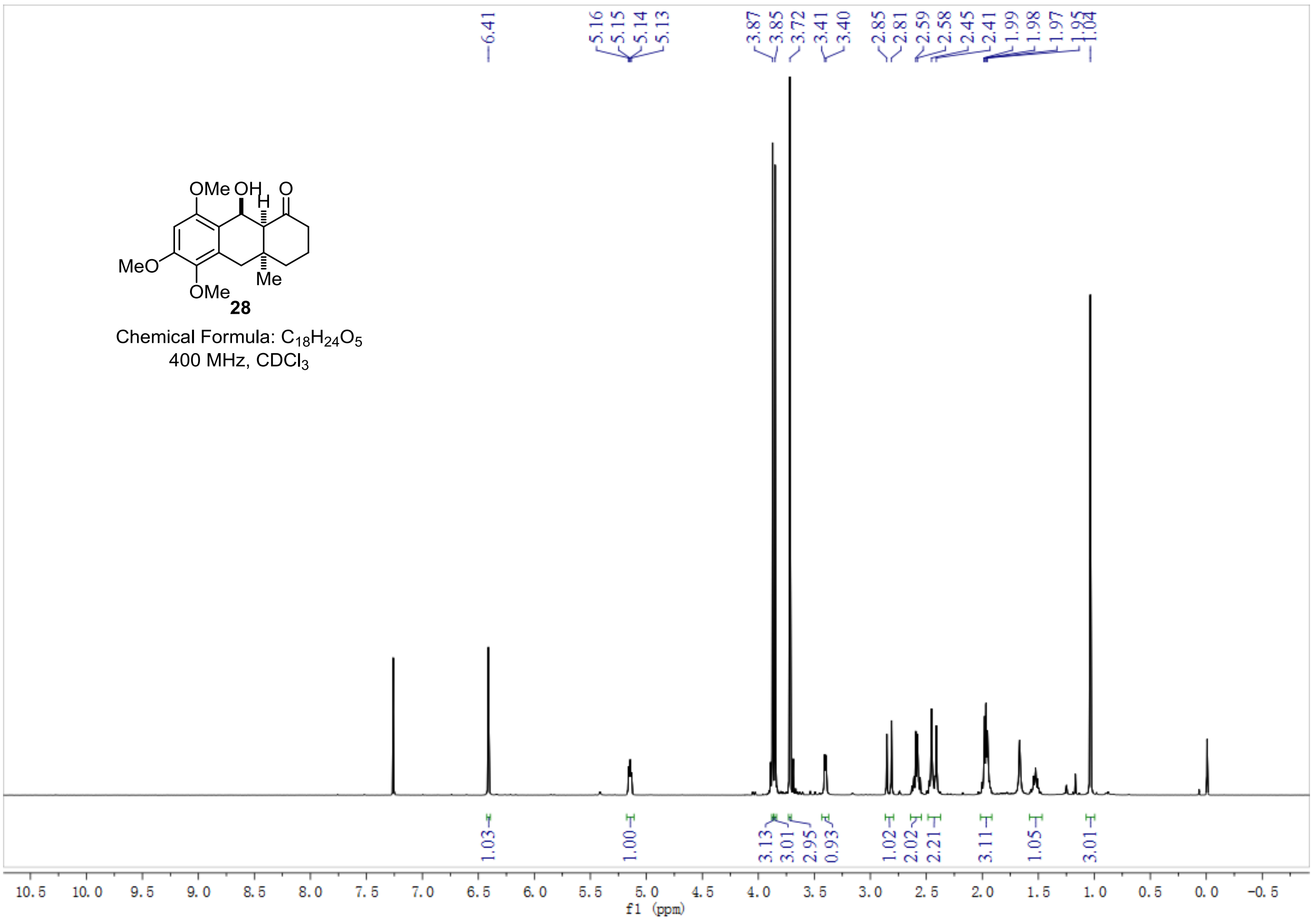

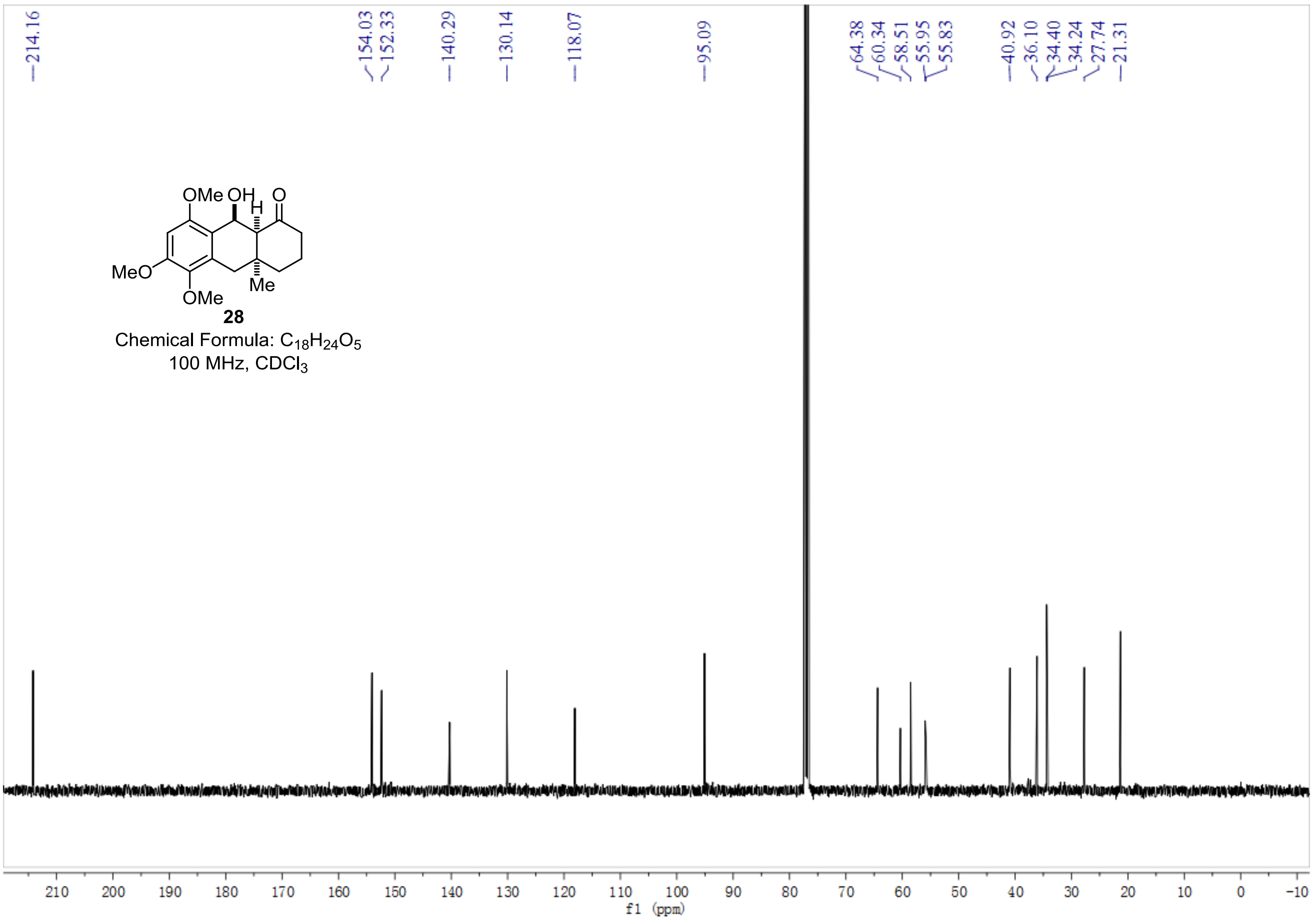

Supplementary Figure 8. ^{1}H and ^{13}C NMR spectra for 28.

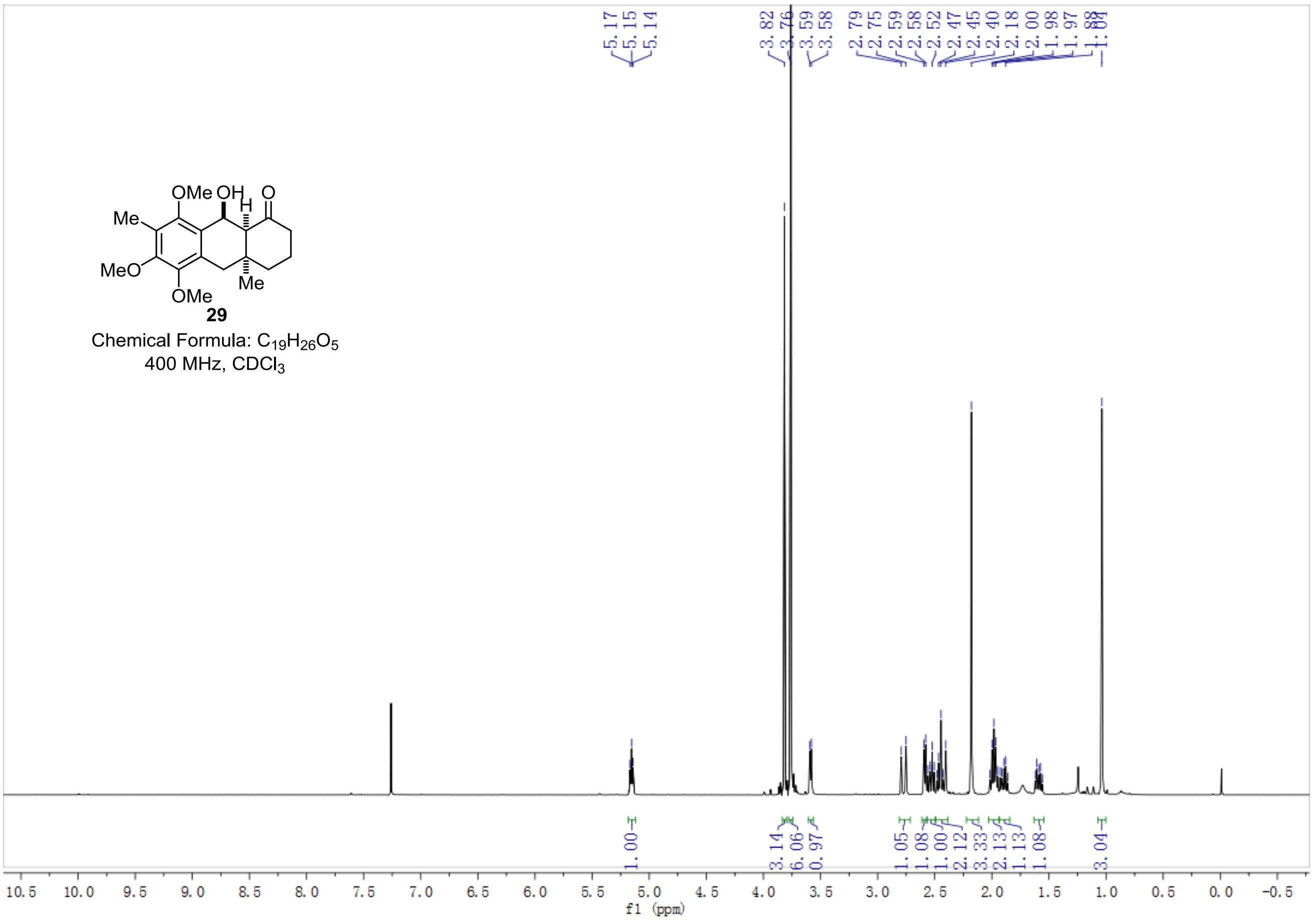

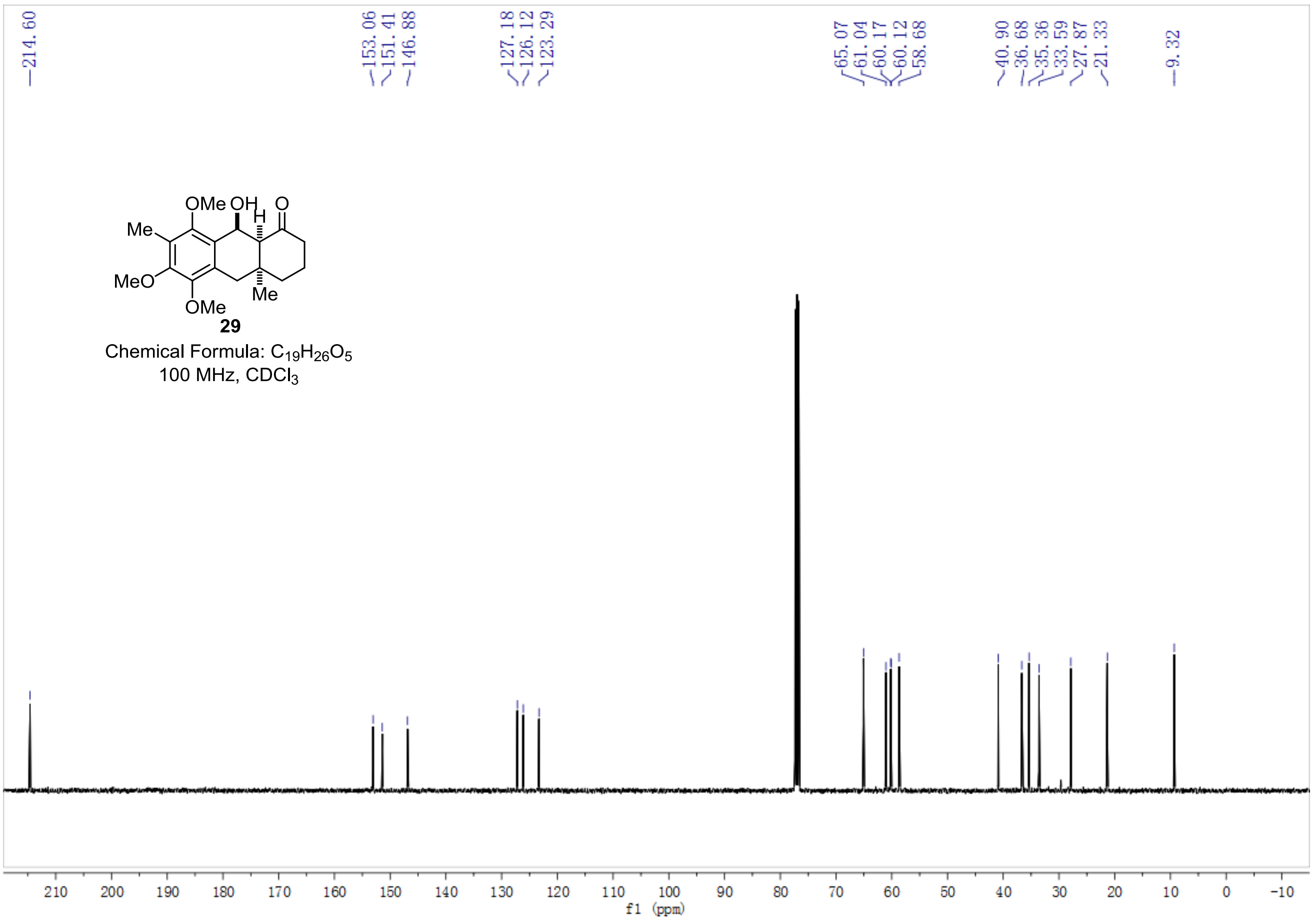

Supplementary Figure 9. ^{1}H and ^{13}C NMR spectra for 29.

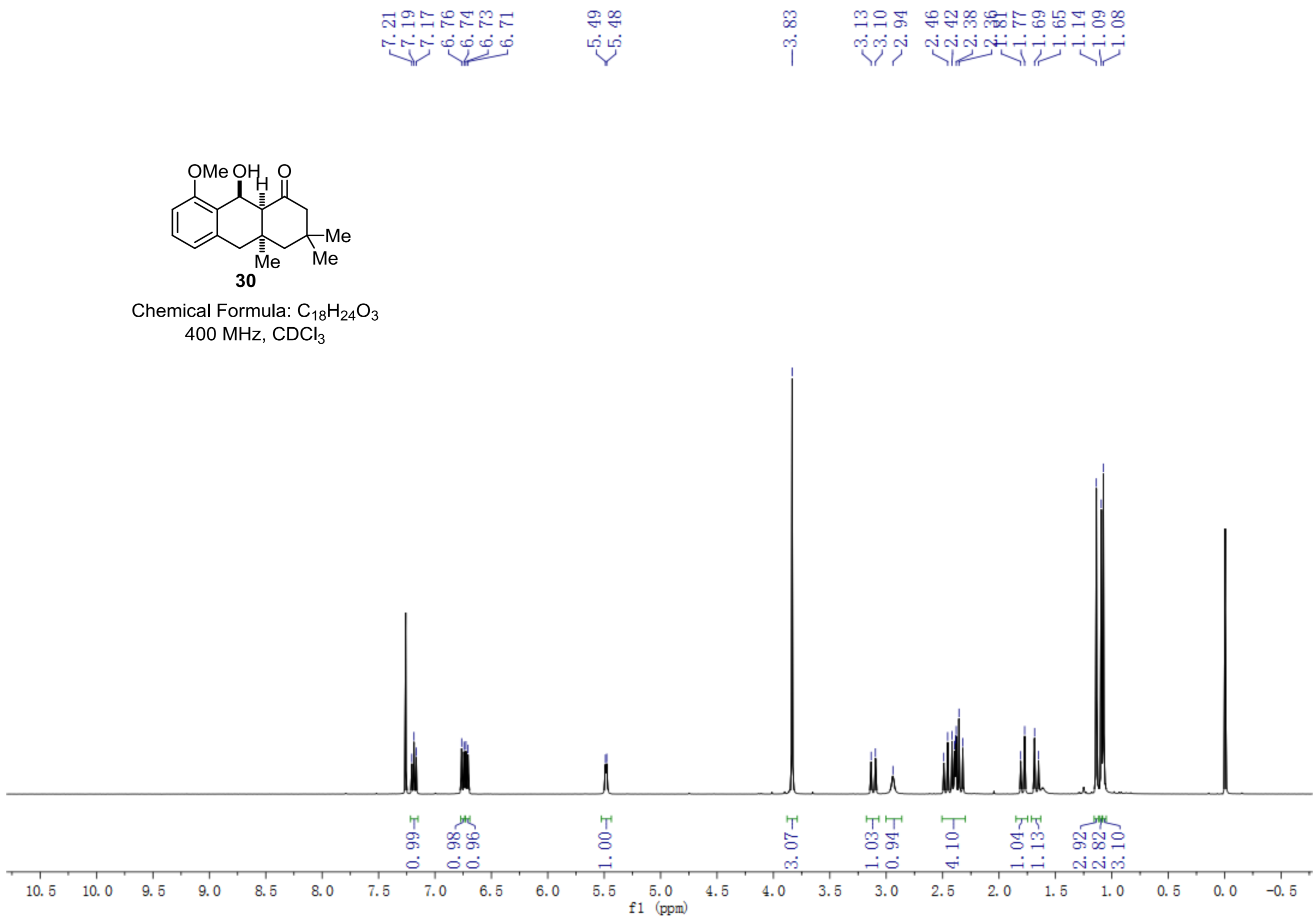

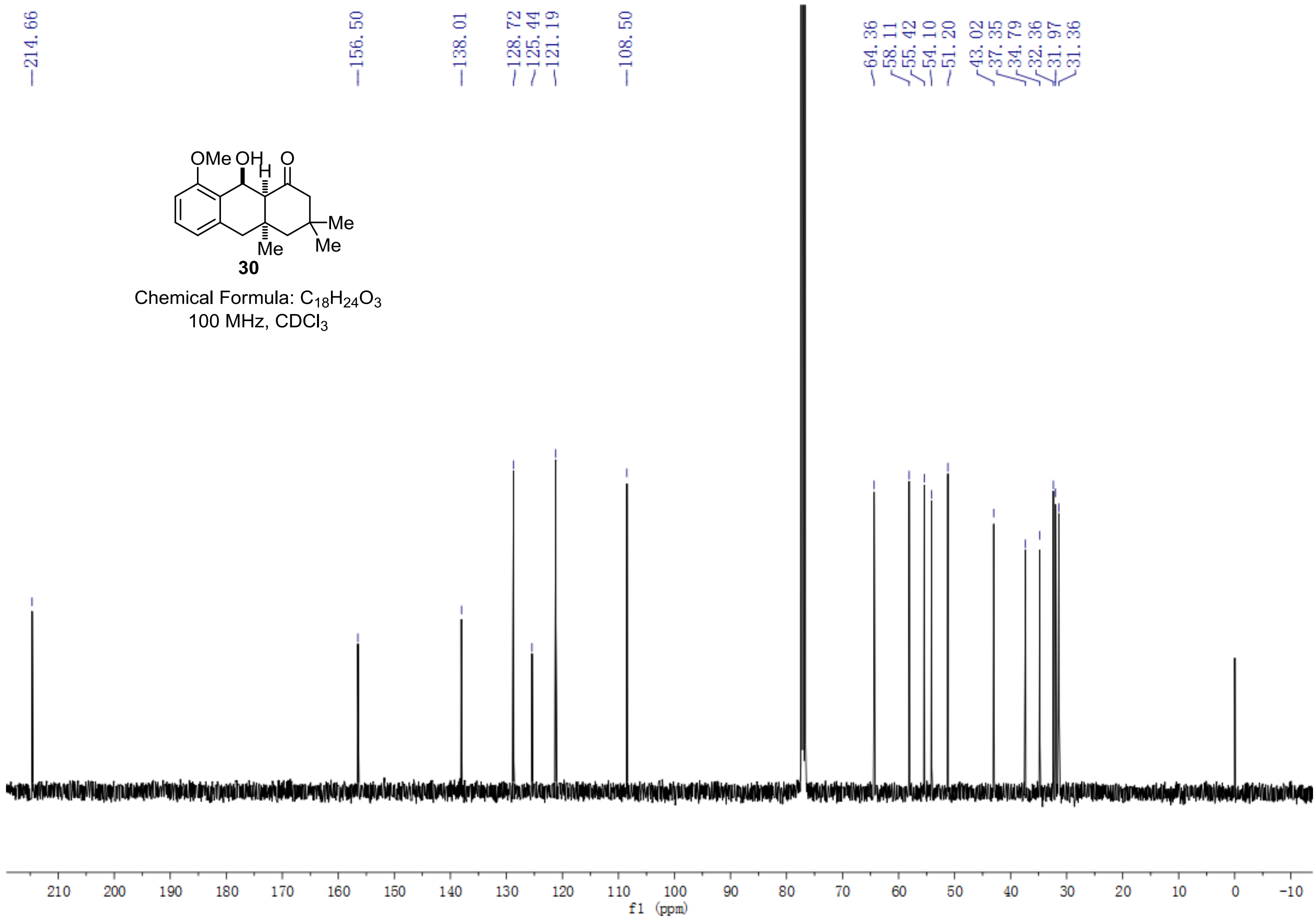

Supplementary Figure 10. ^{1}H **and** ^{13}C **NMR spectra for 30.**

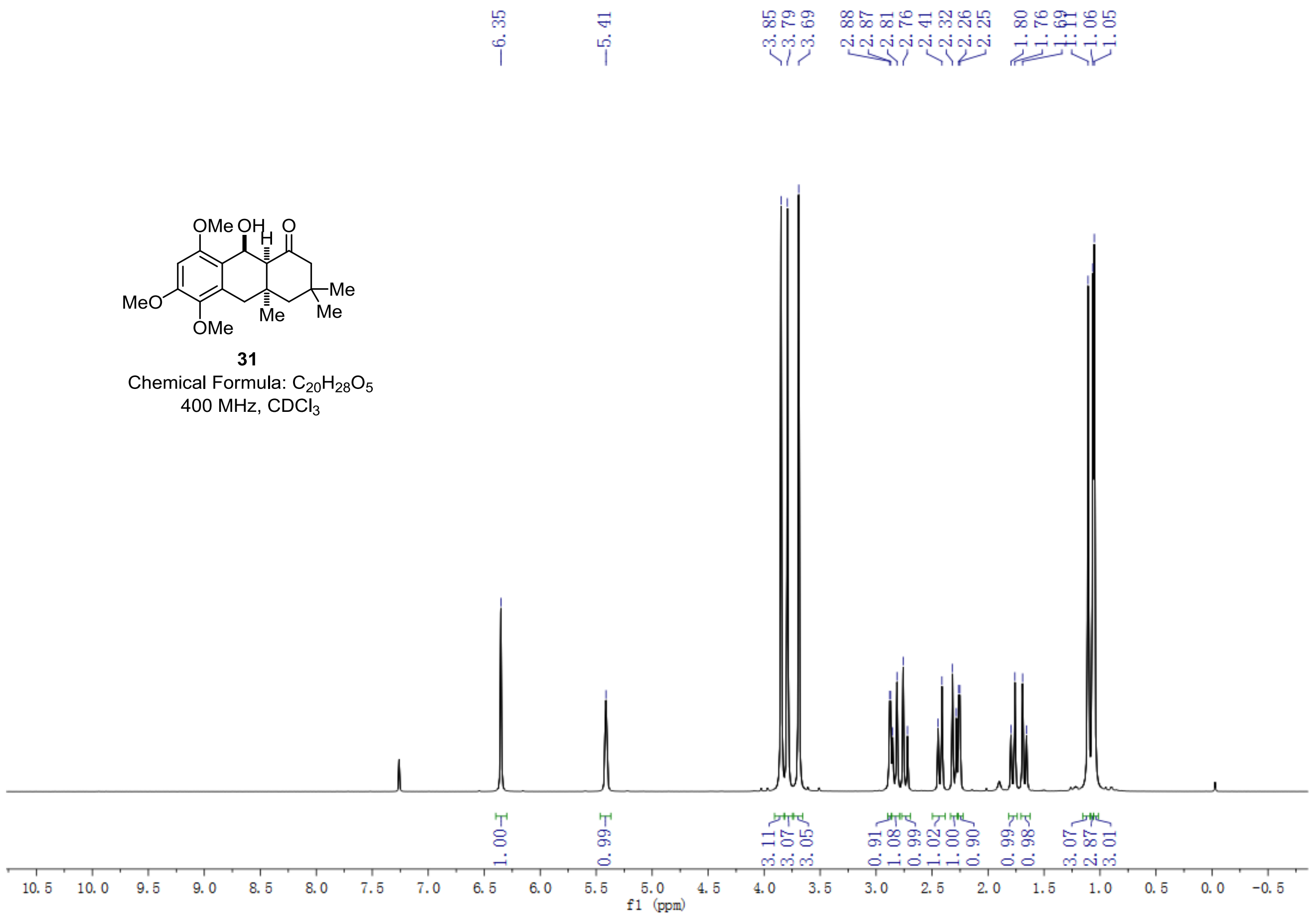

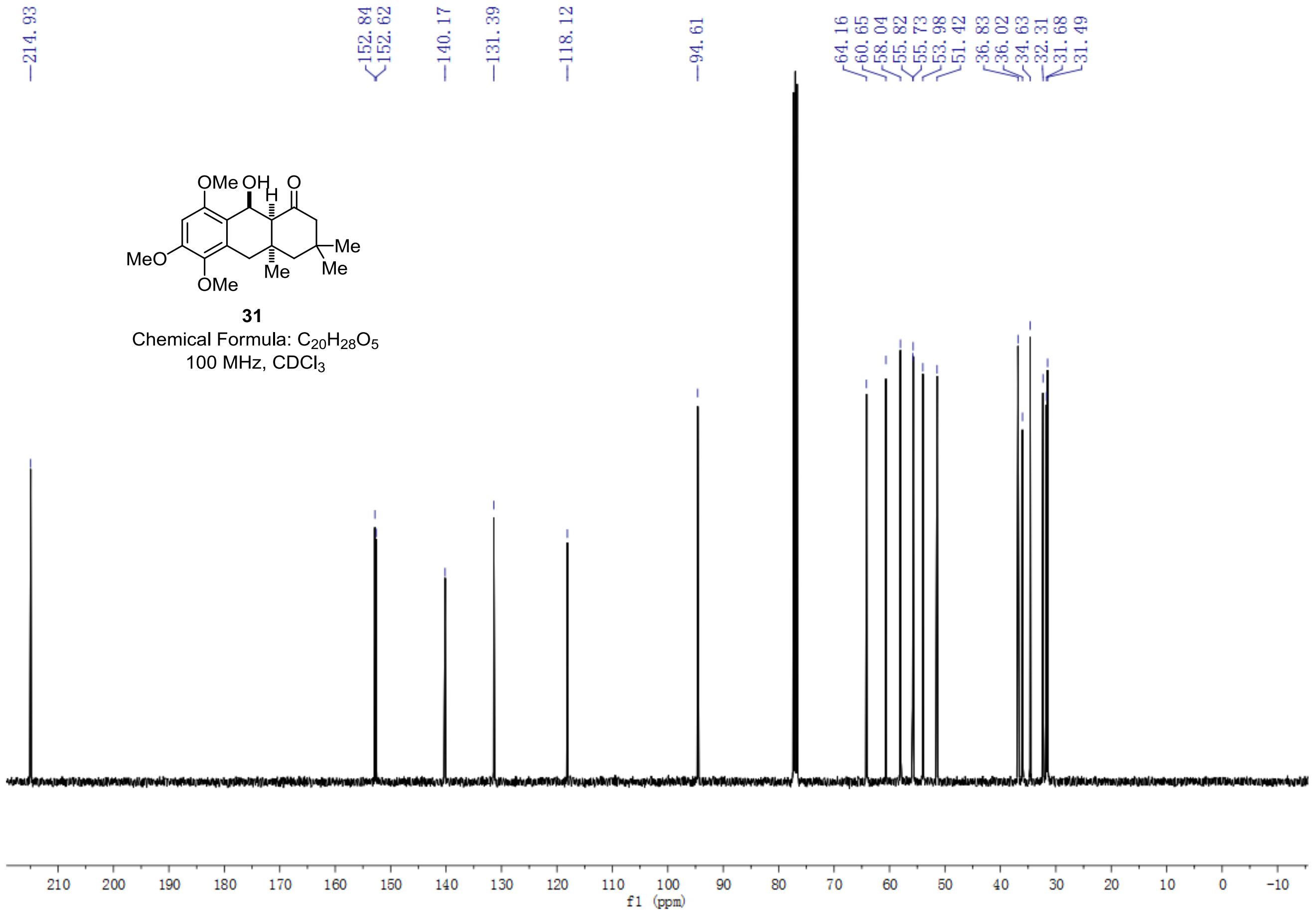

Supplementary Figure 11. ^{1}H **and** ^{13}C **NMR spectra for 31.**

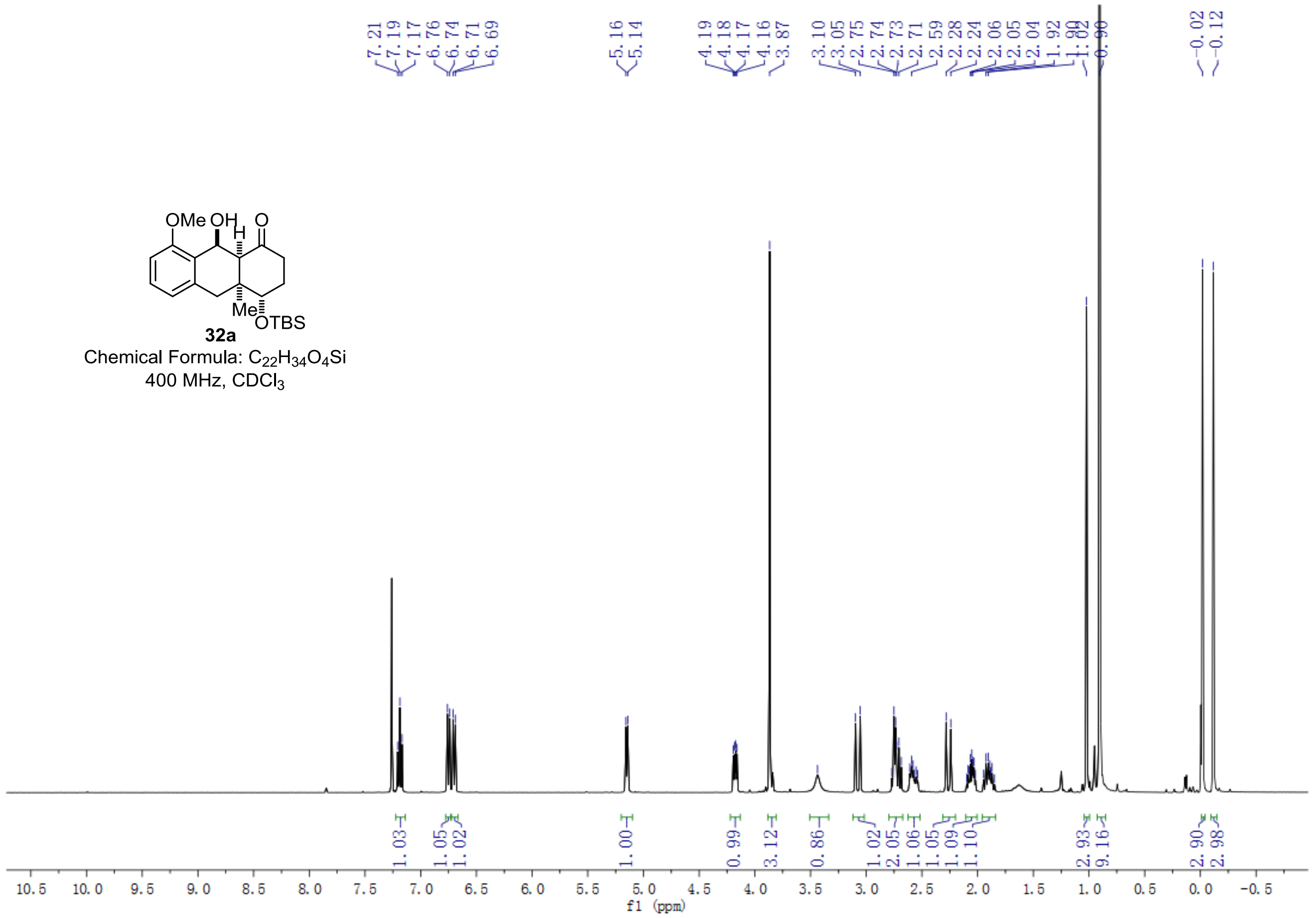

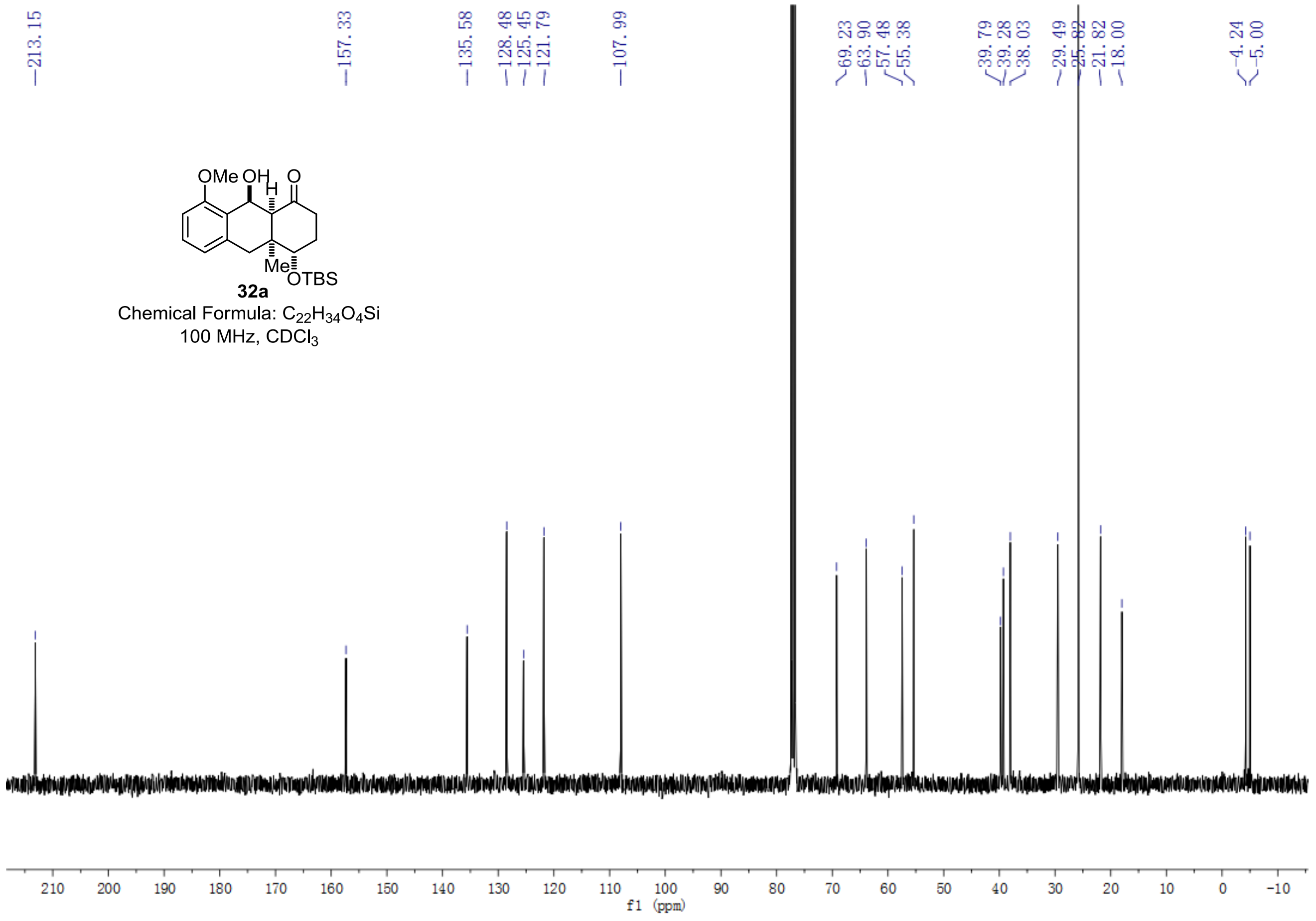

Supplementary Figure 12. ^{1}H and ^{13}C NMR spectra for 32a.

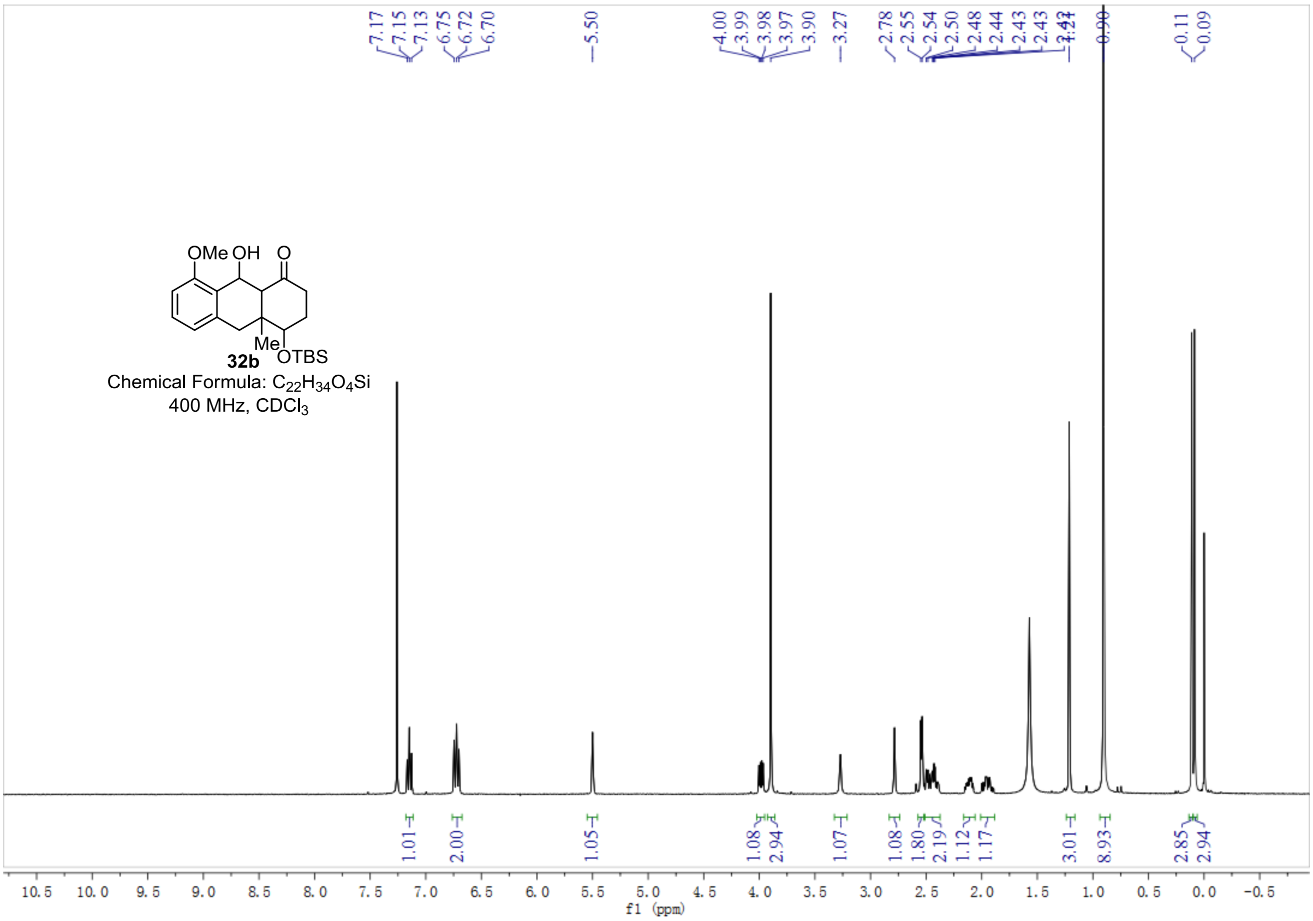

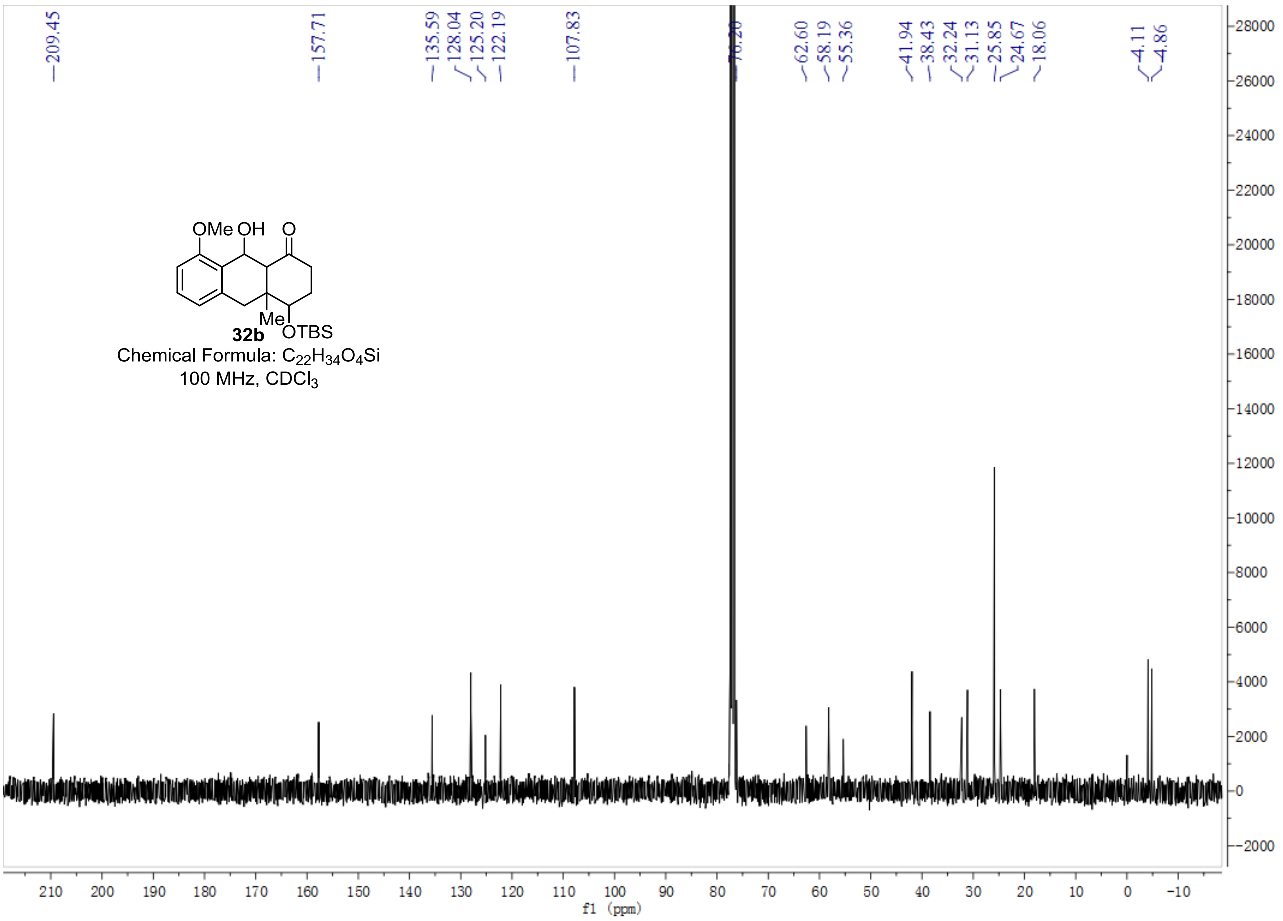

Supplementary Figure 13. ^{1}H **and** ^{13}C **NMR spectra for 32b.**

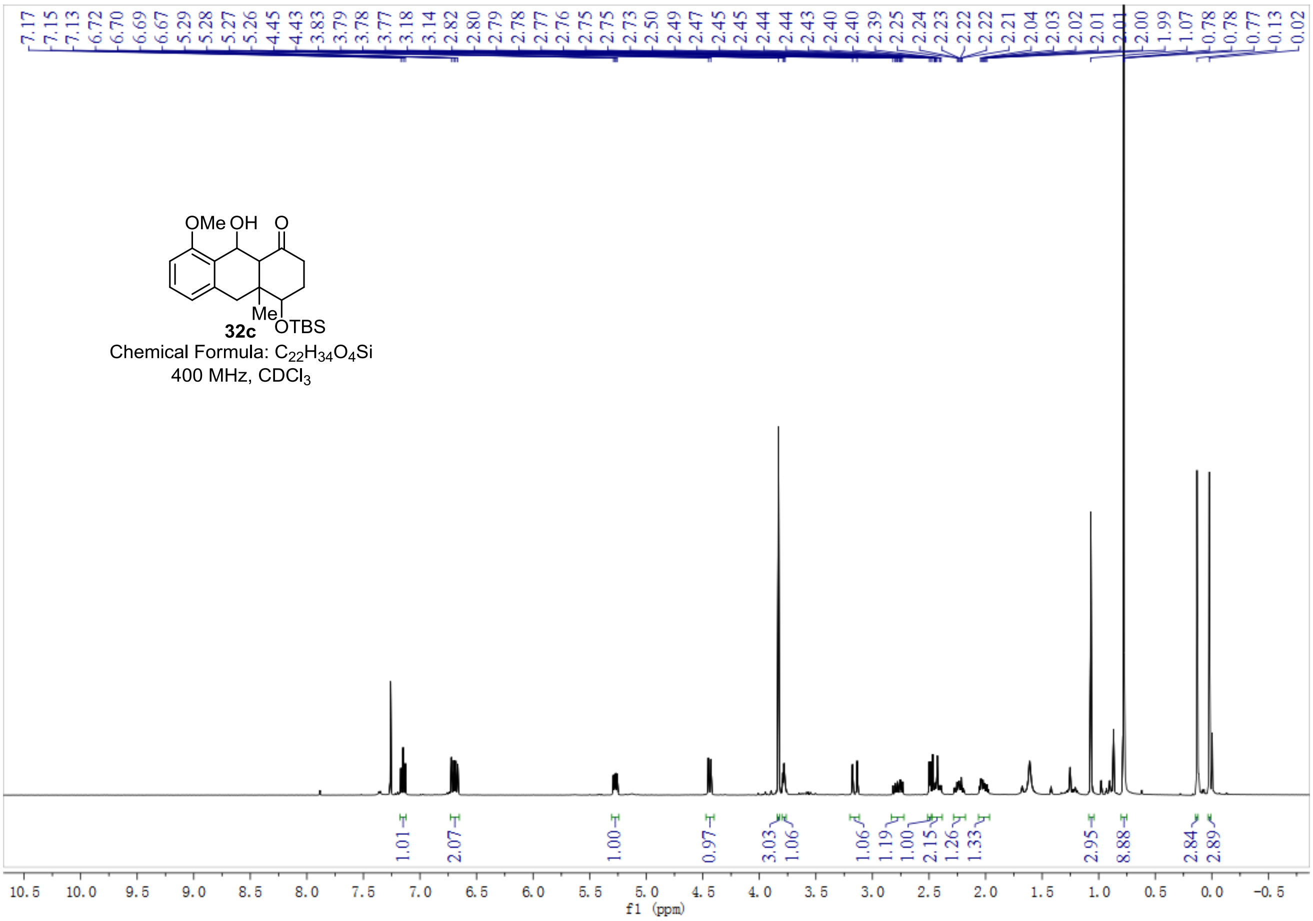

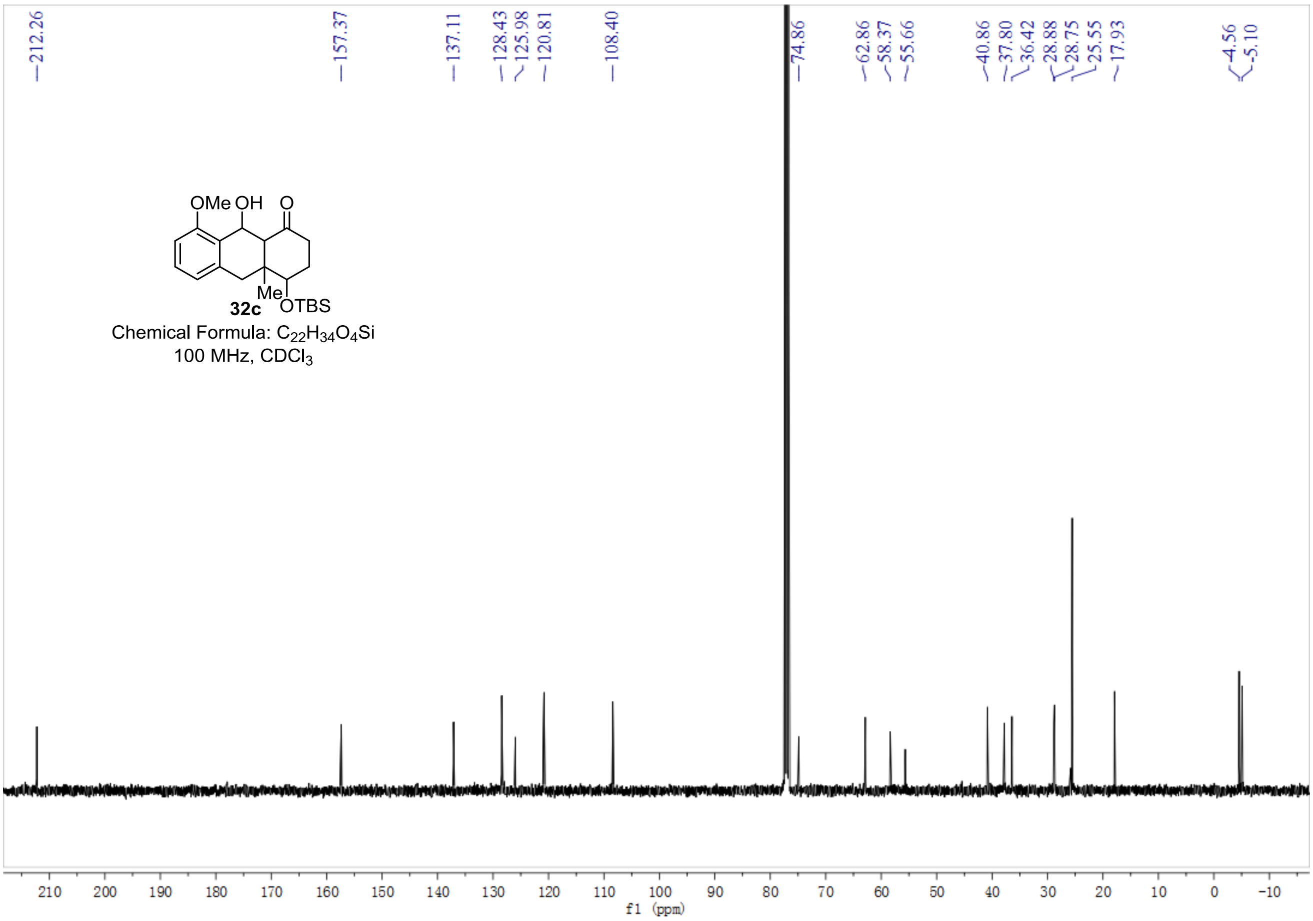

Supplementary Figure 14. ^{1}**H and** ^{13}**C NMR spectra for 32c.**

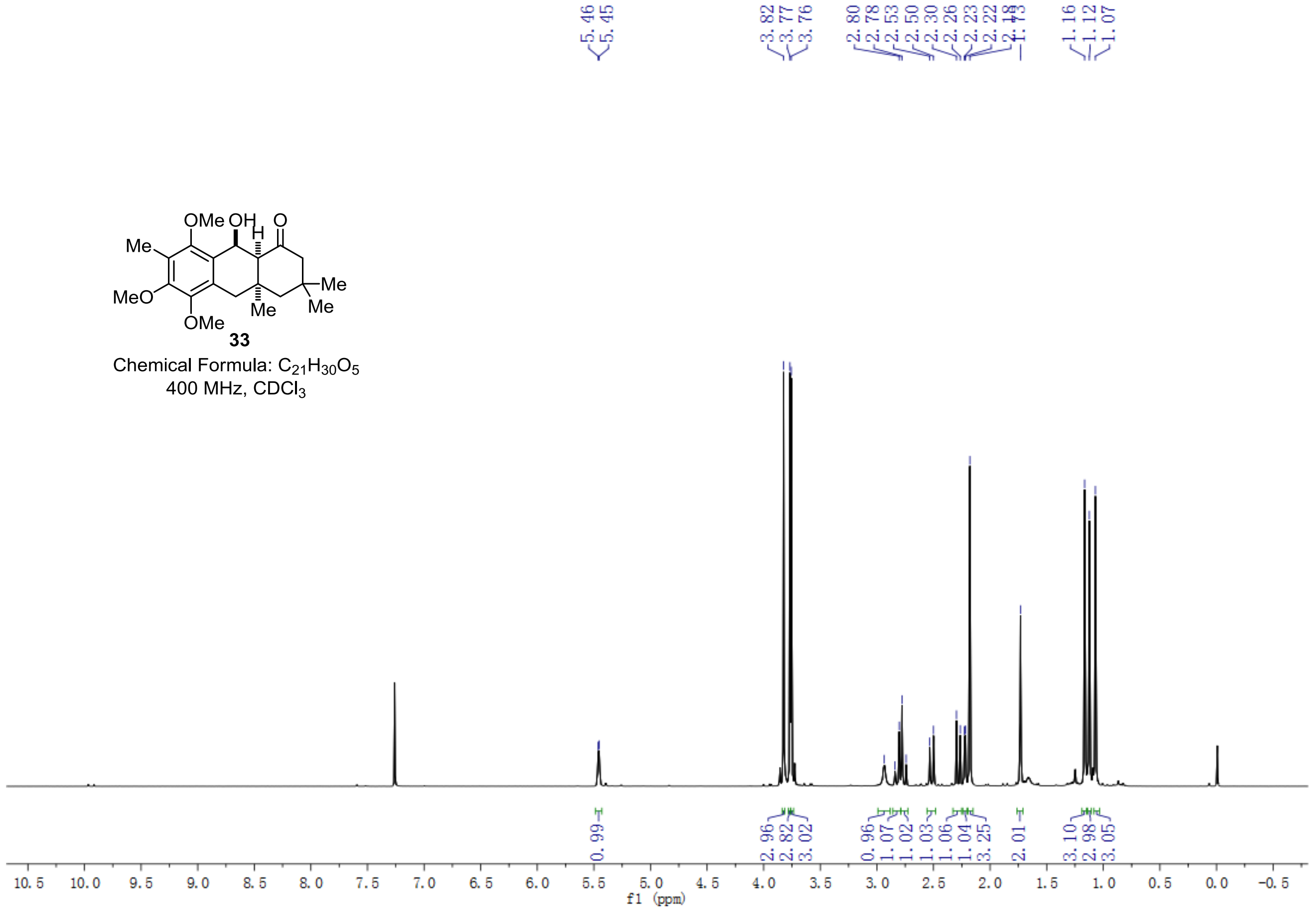

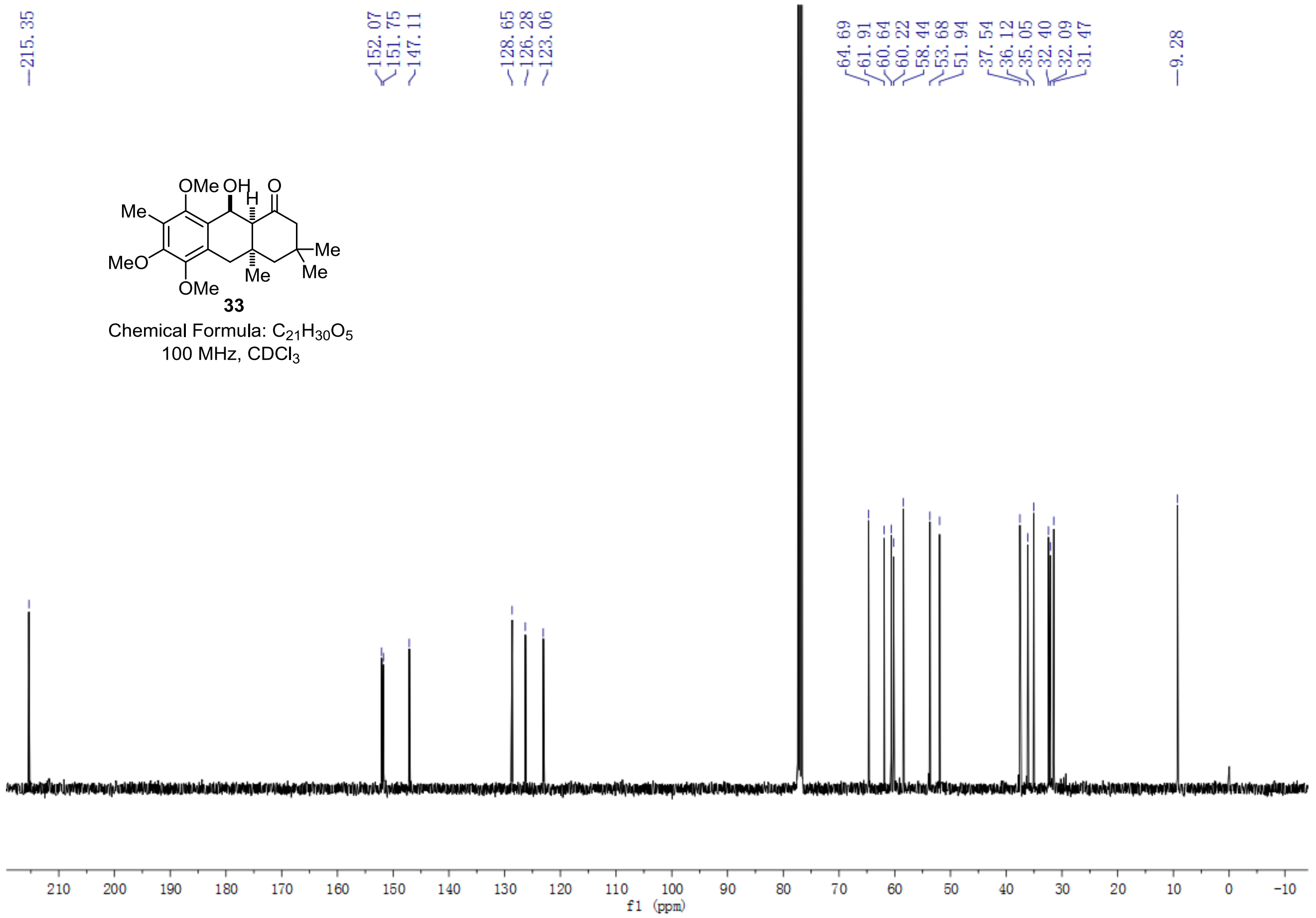

Supplementary Figure 15. ^{1}H and ^{13}C NMR spectra for 33.

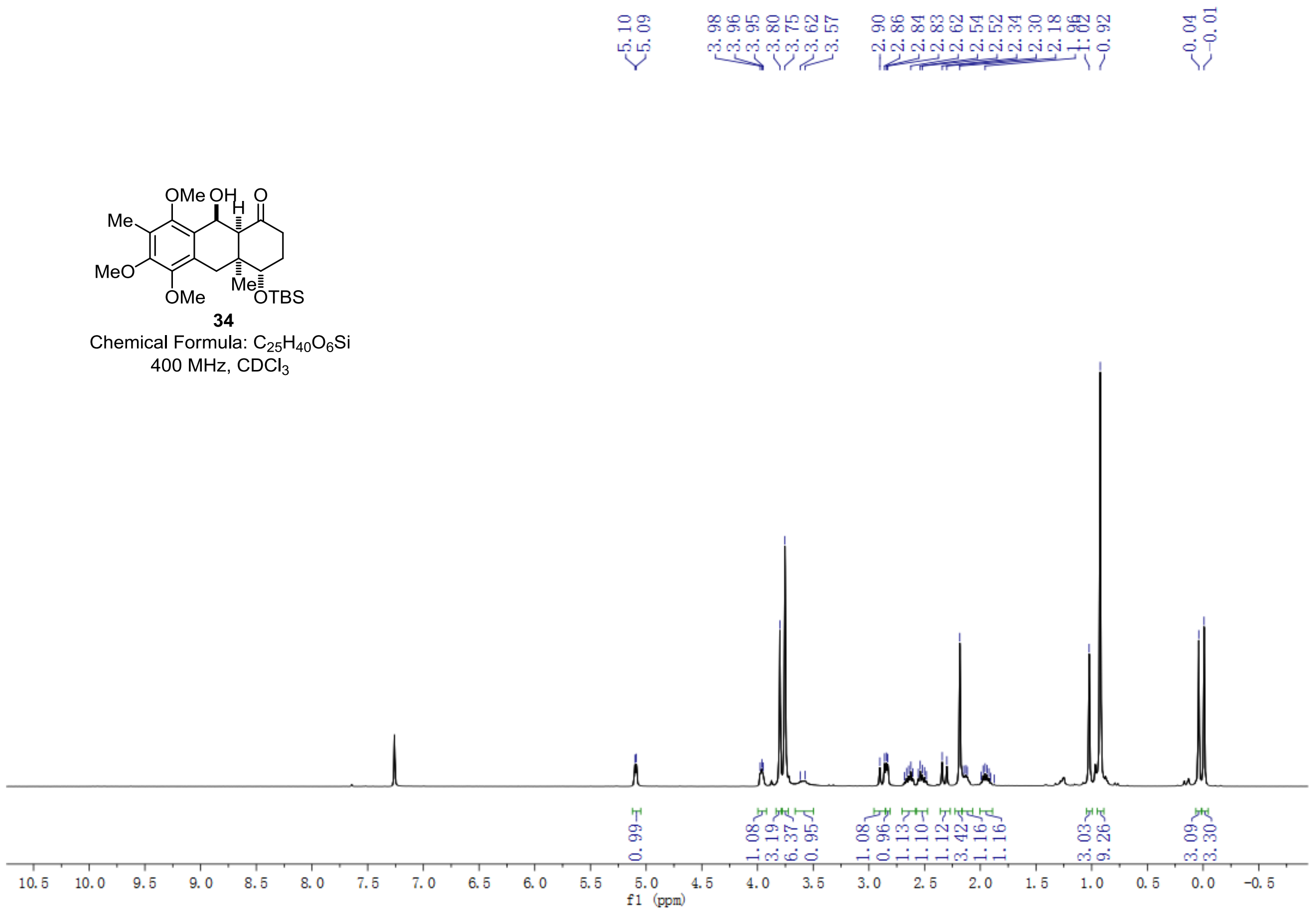

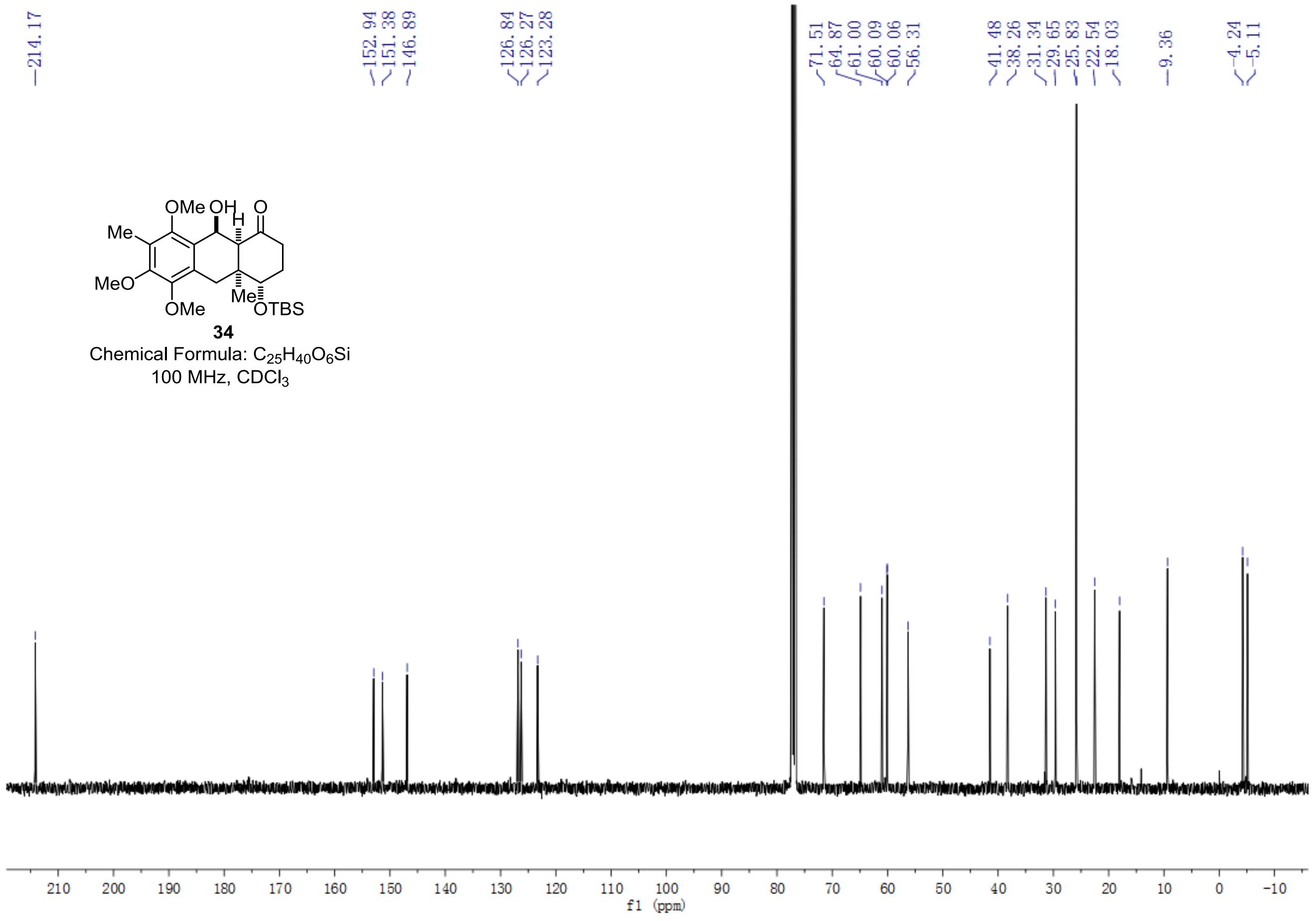

Supplementary Figure 16.^{1}**H and** ^{13}**C NMR spectra for 34.**

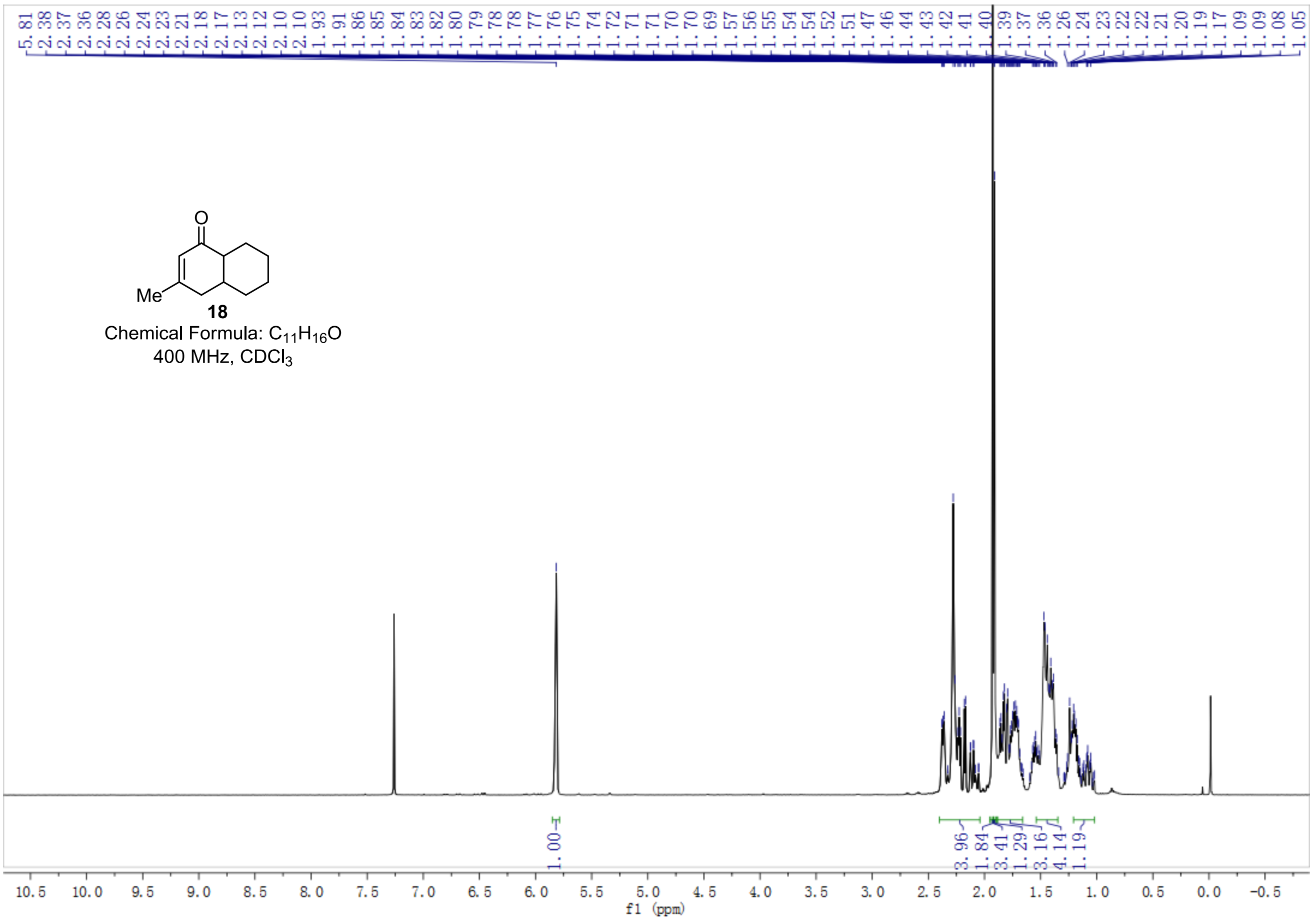

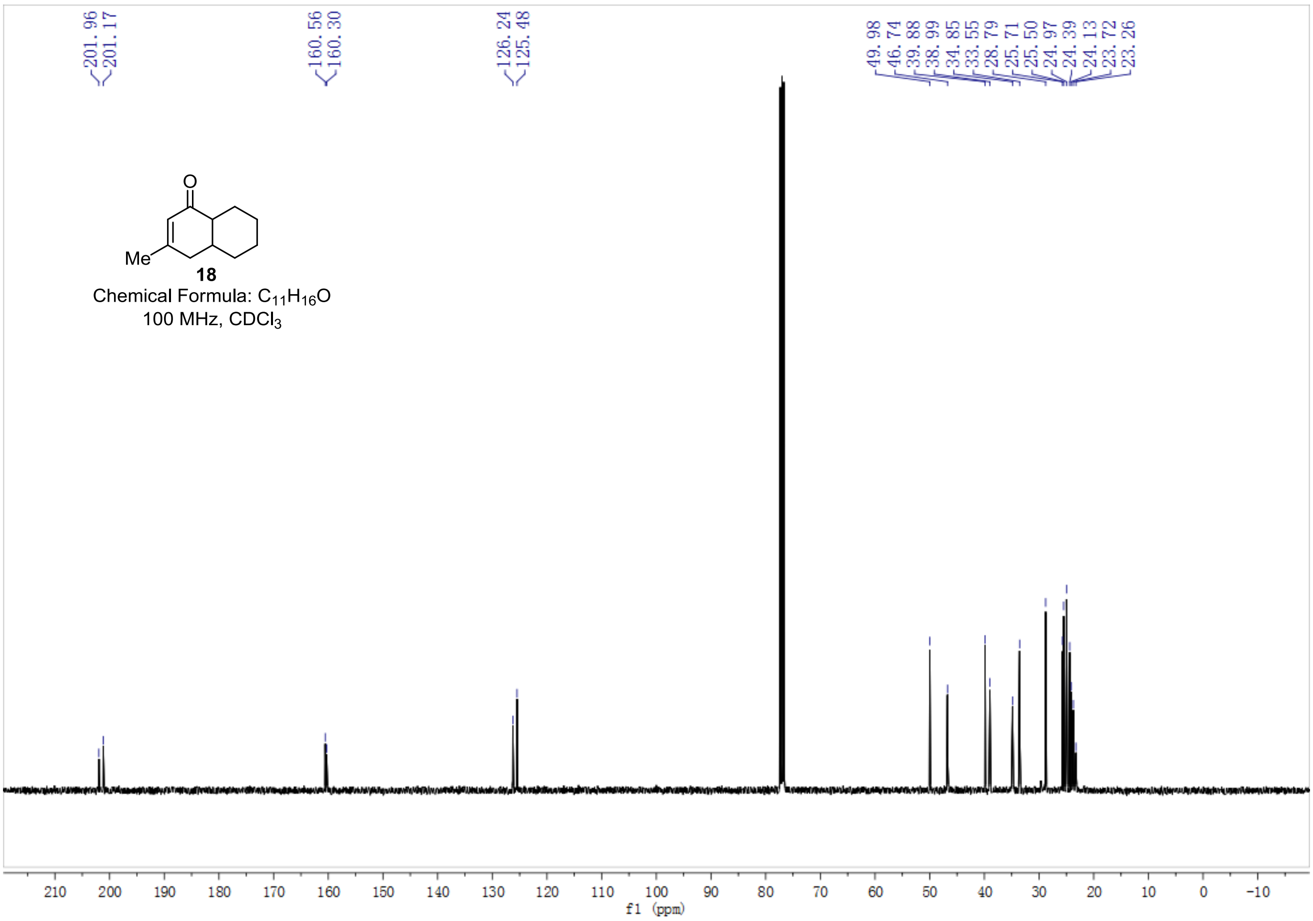

Supplementary Figure 17.^{1}H and ^{13}C NMR spectra for 18.

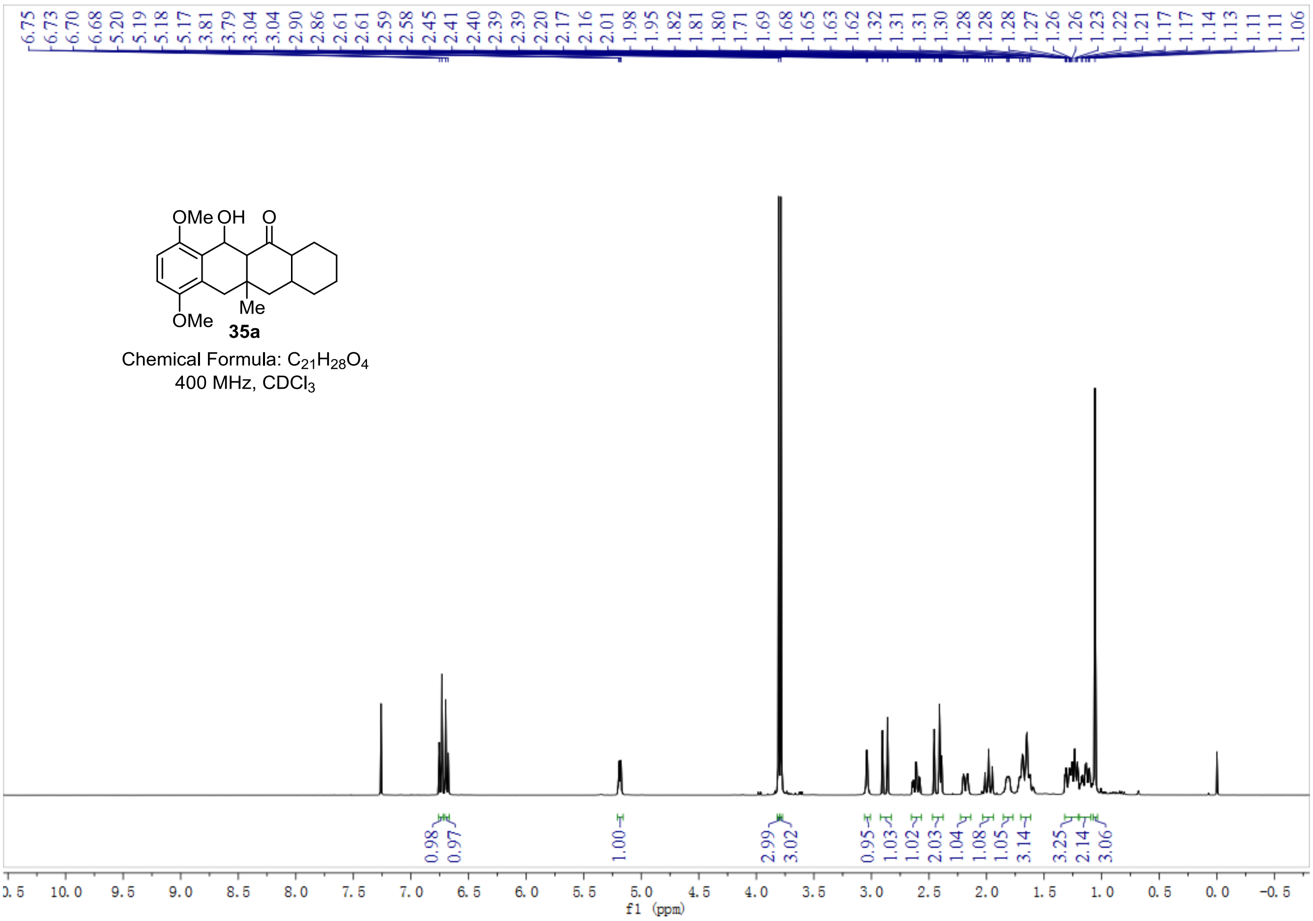

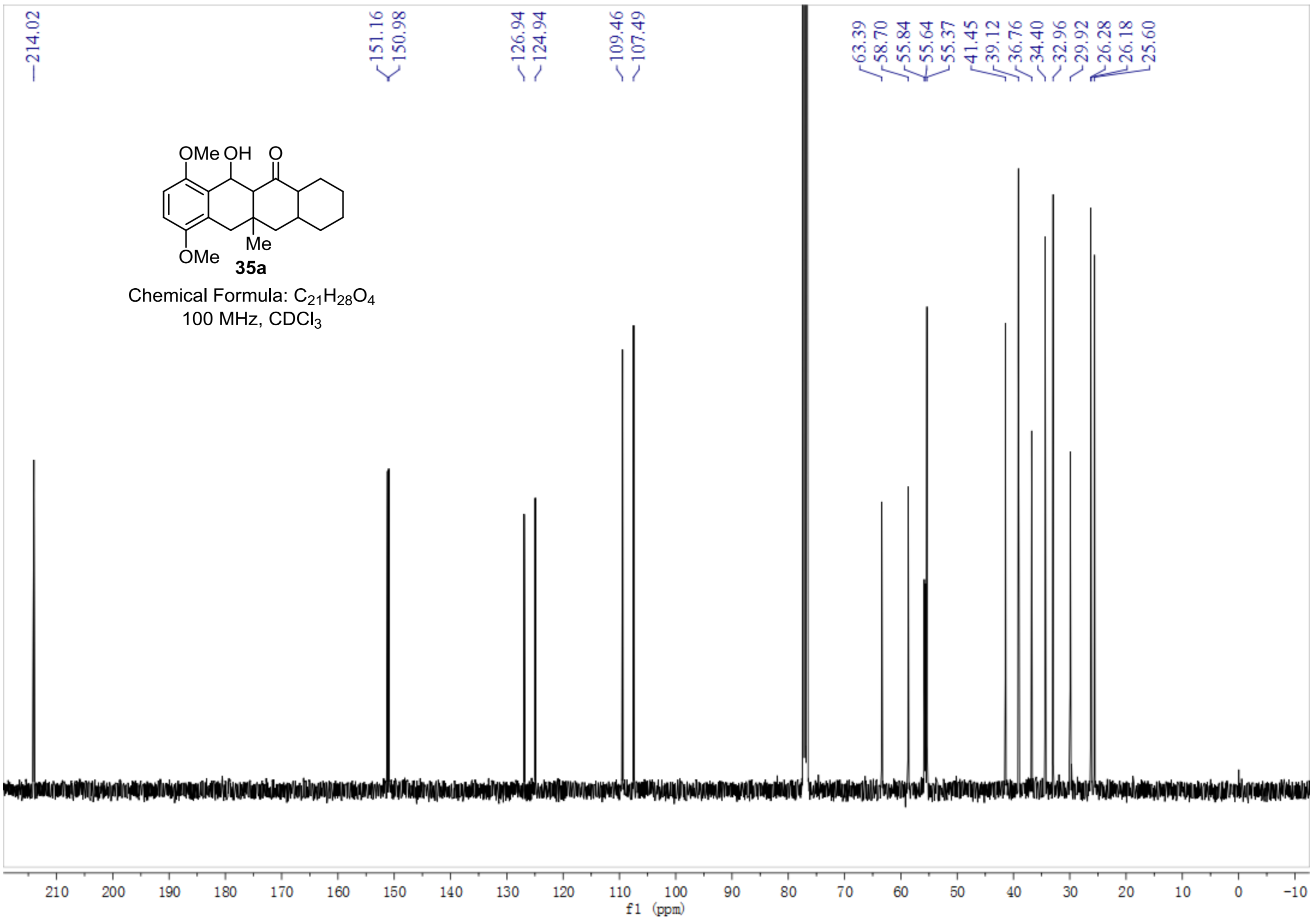

Supplementary Figure 18. ^{1}H **and** ^{13}C **NMR spectra for 35a.**

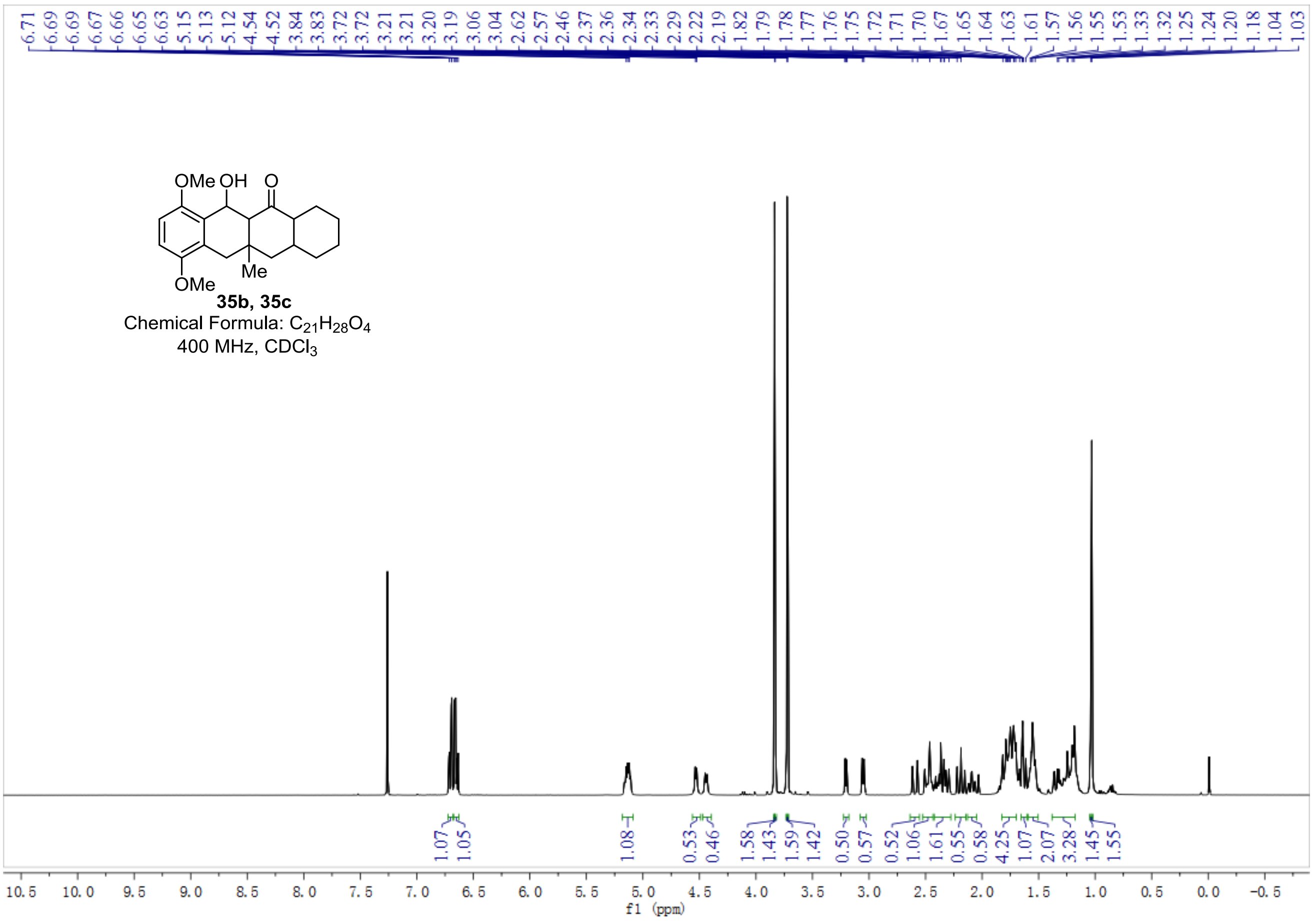

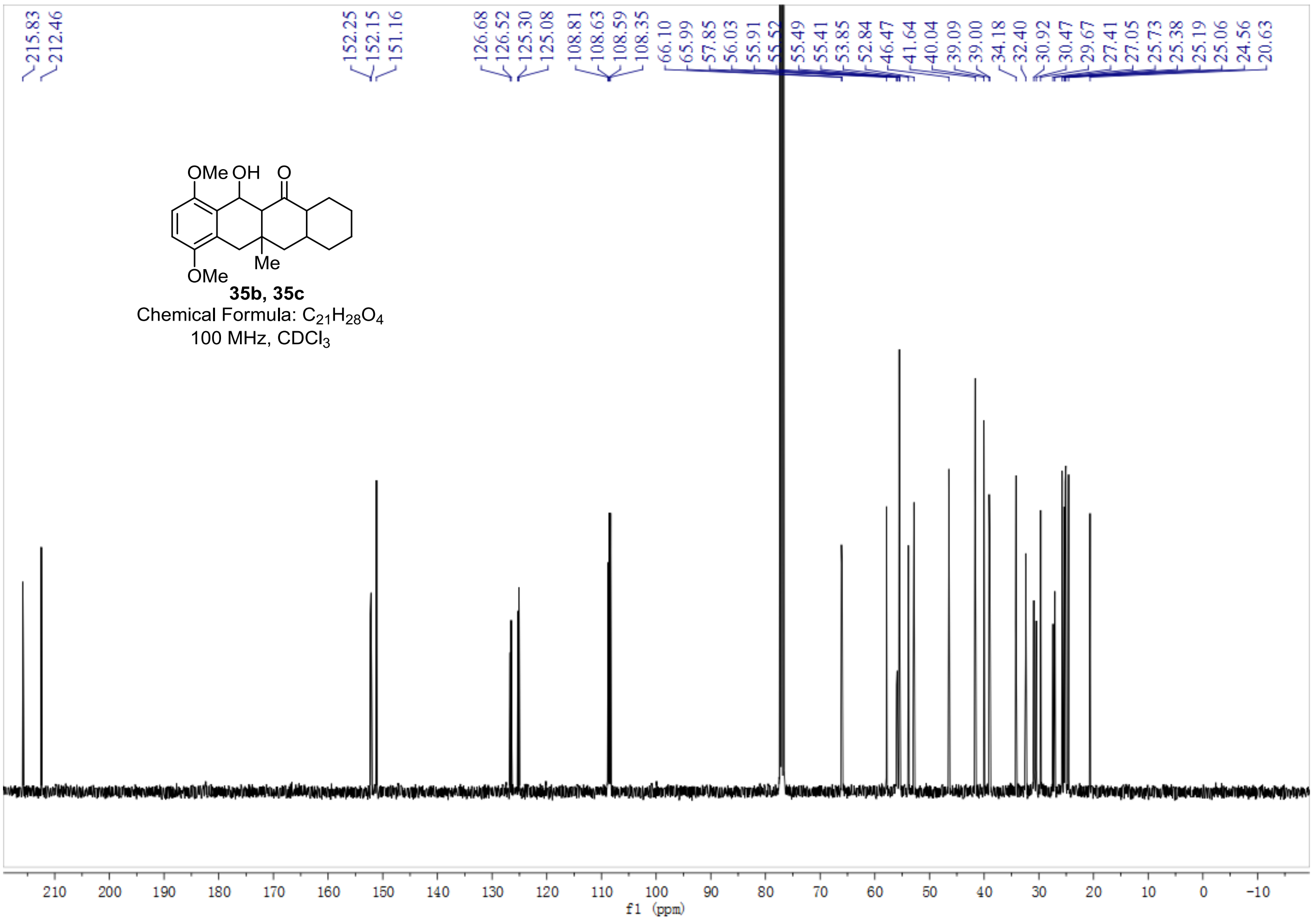

Supplementary Figure 19. ^{1}H **and** ^{13}C **NMR spectra for 35b, 35c.**

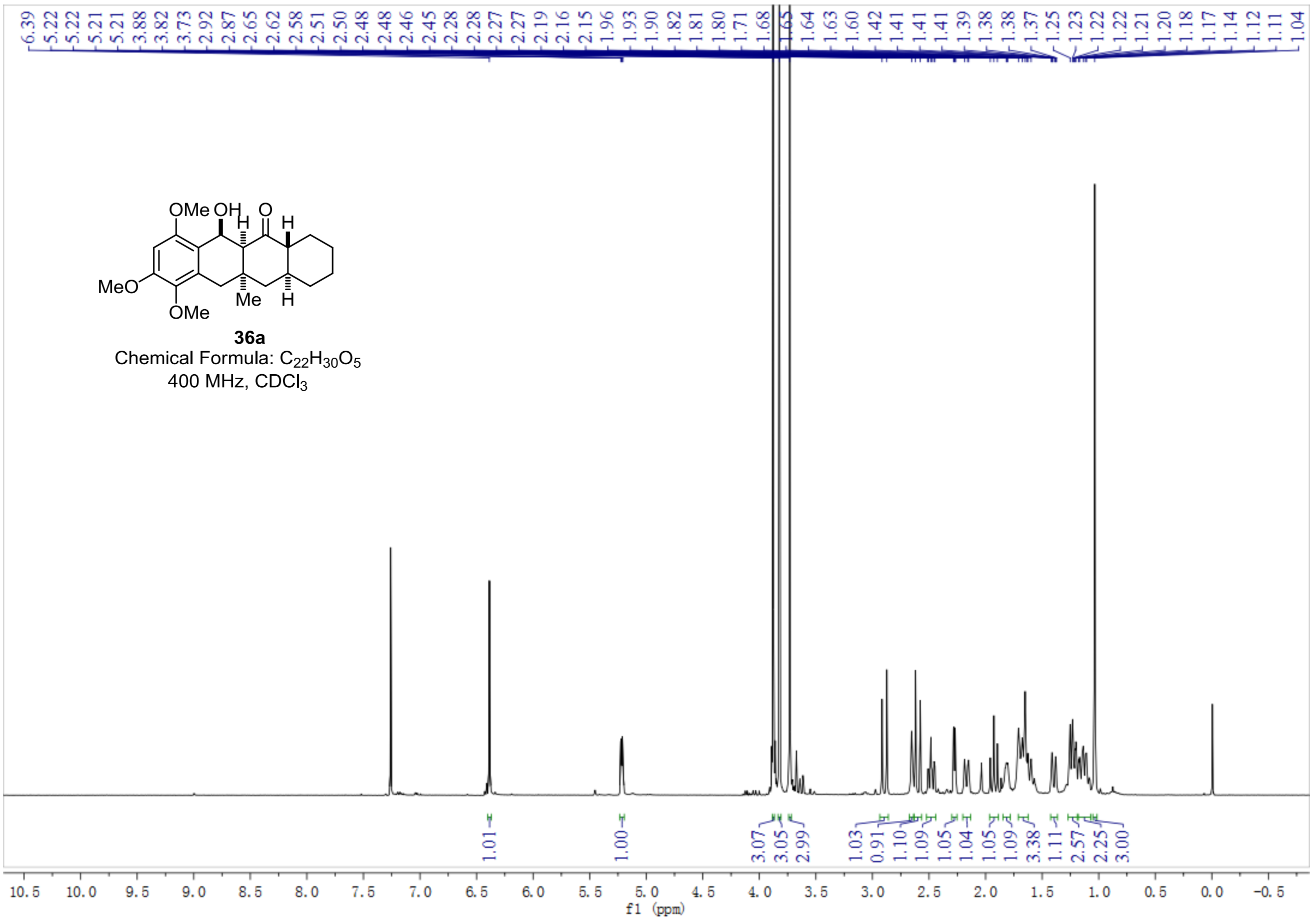

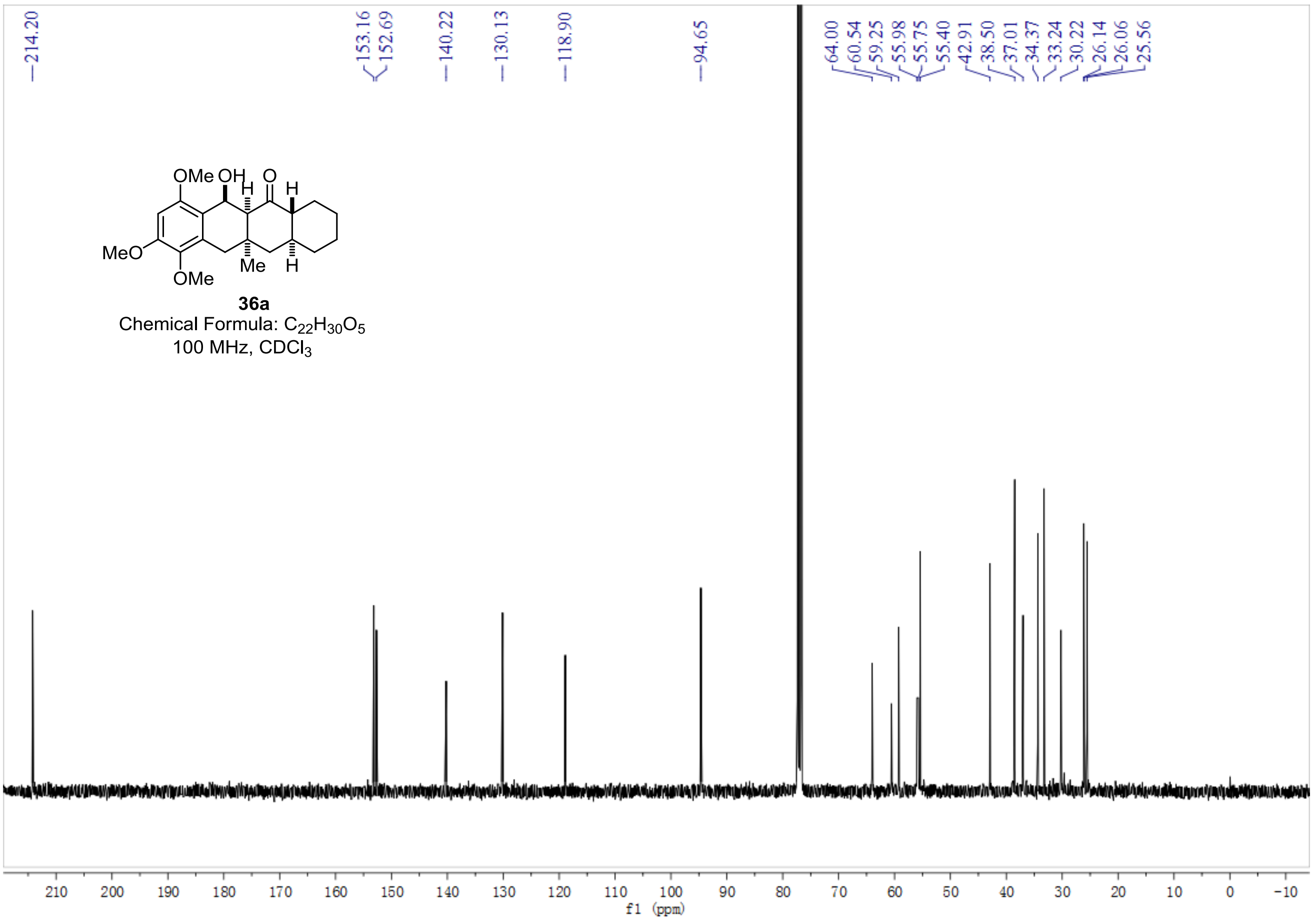

Supplementary Figure 20. ^{1}H and ^{13}C NMR spectra for 36a.

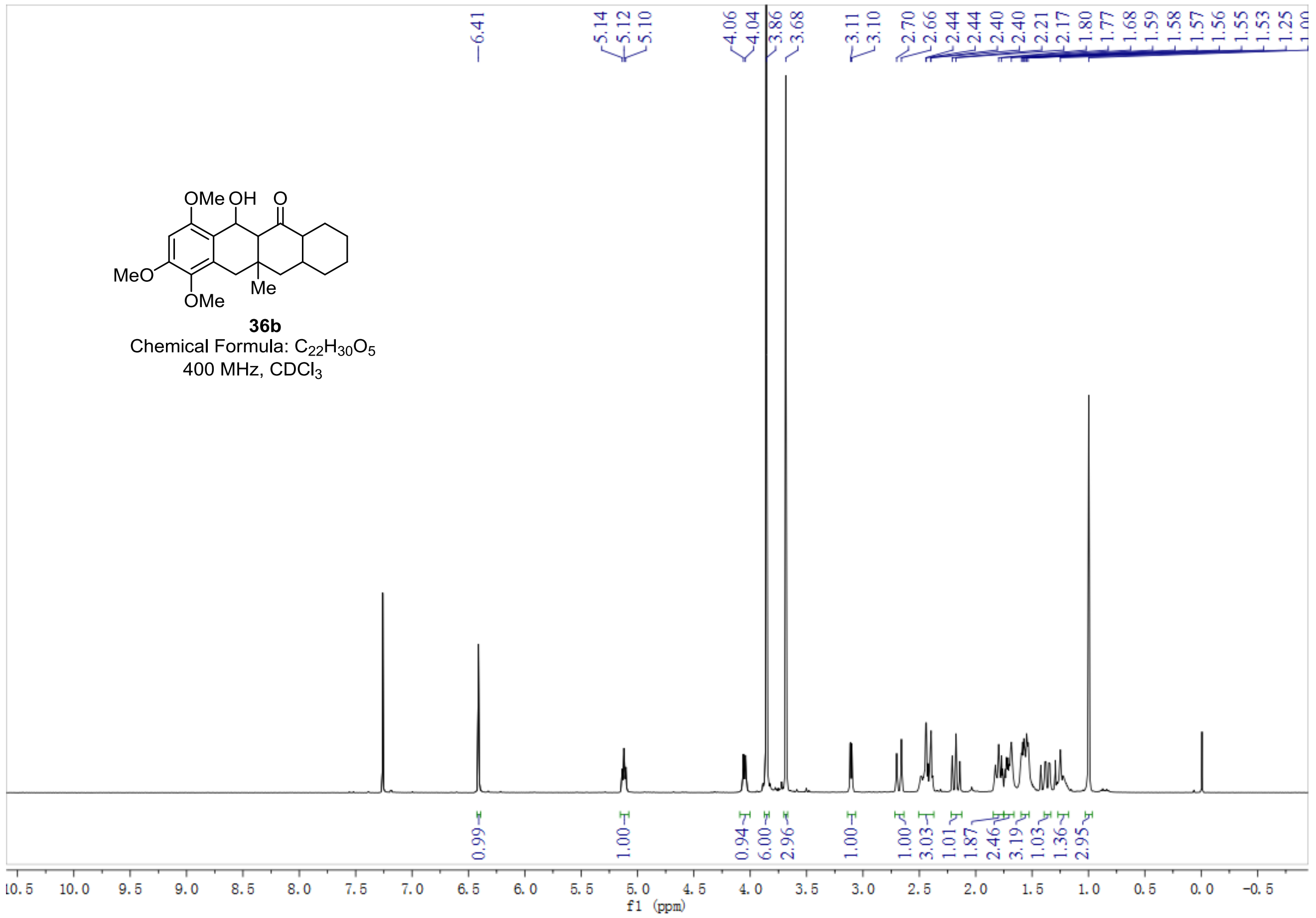

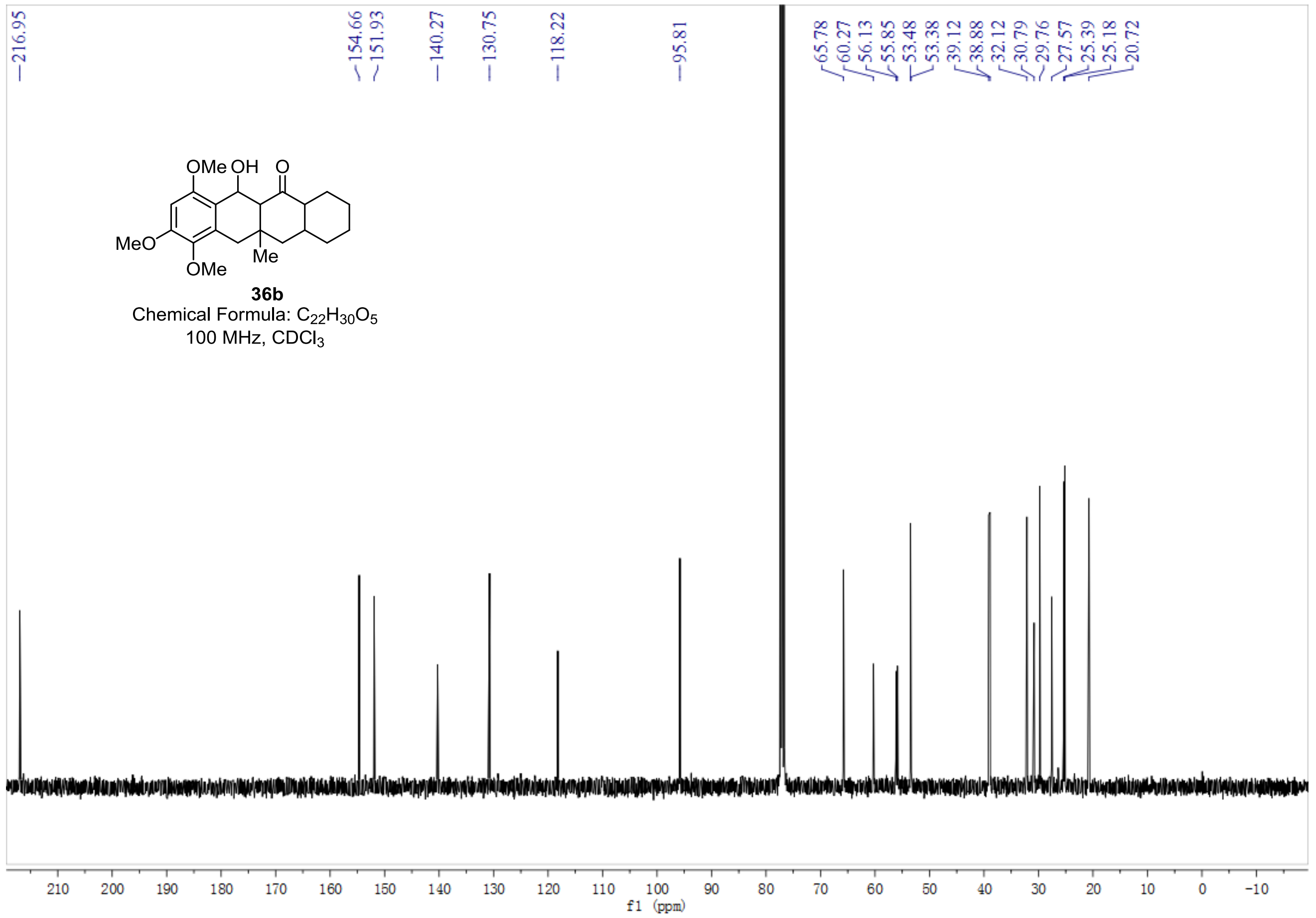

Supplementary Figure 21.^{1}H and ^{13}C NMR spectra for 36b.

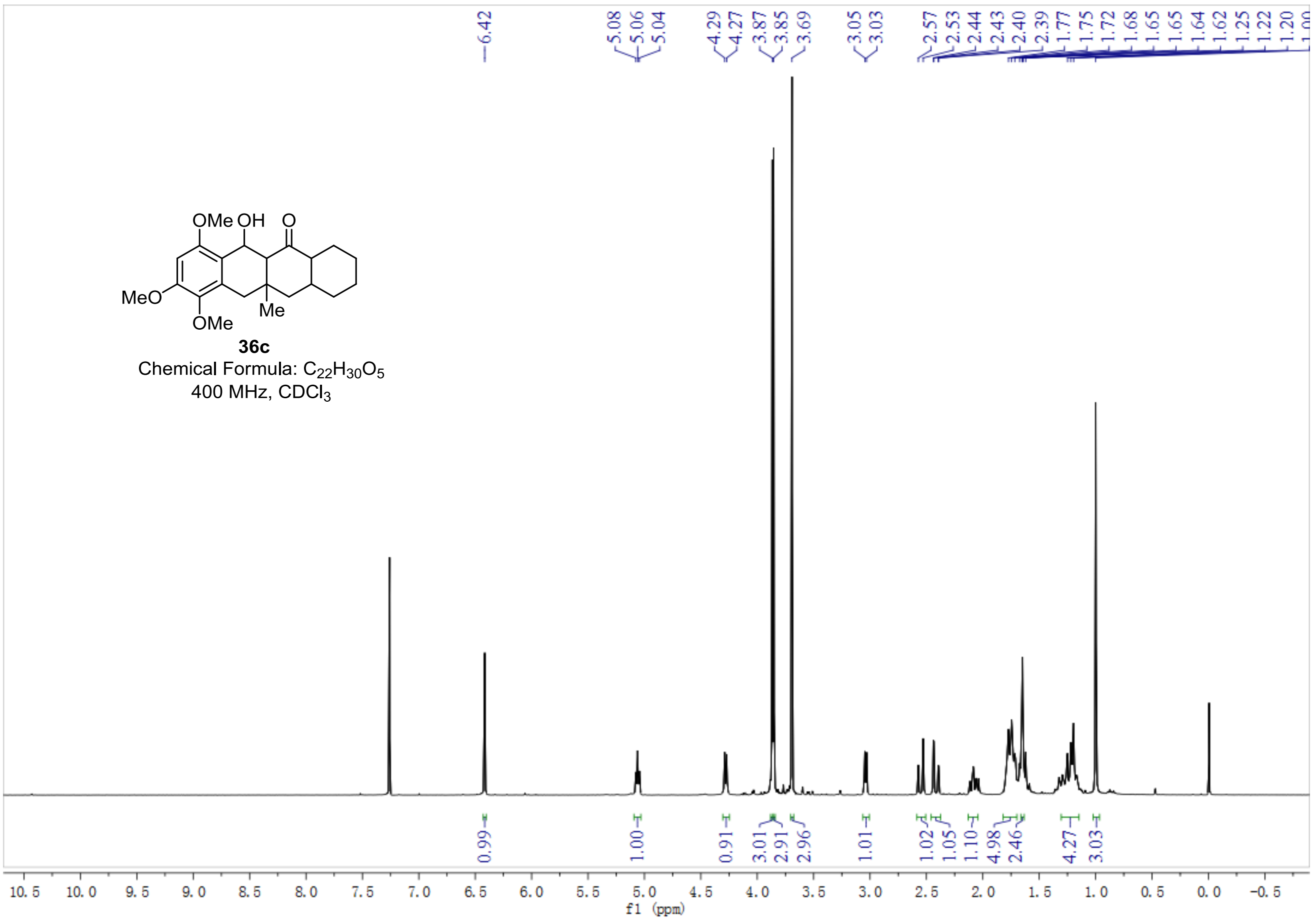

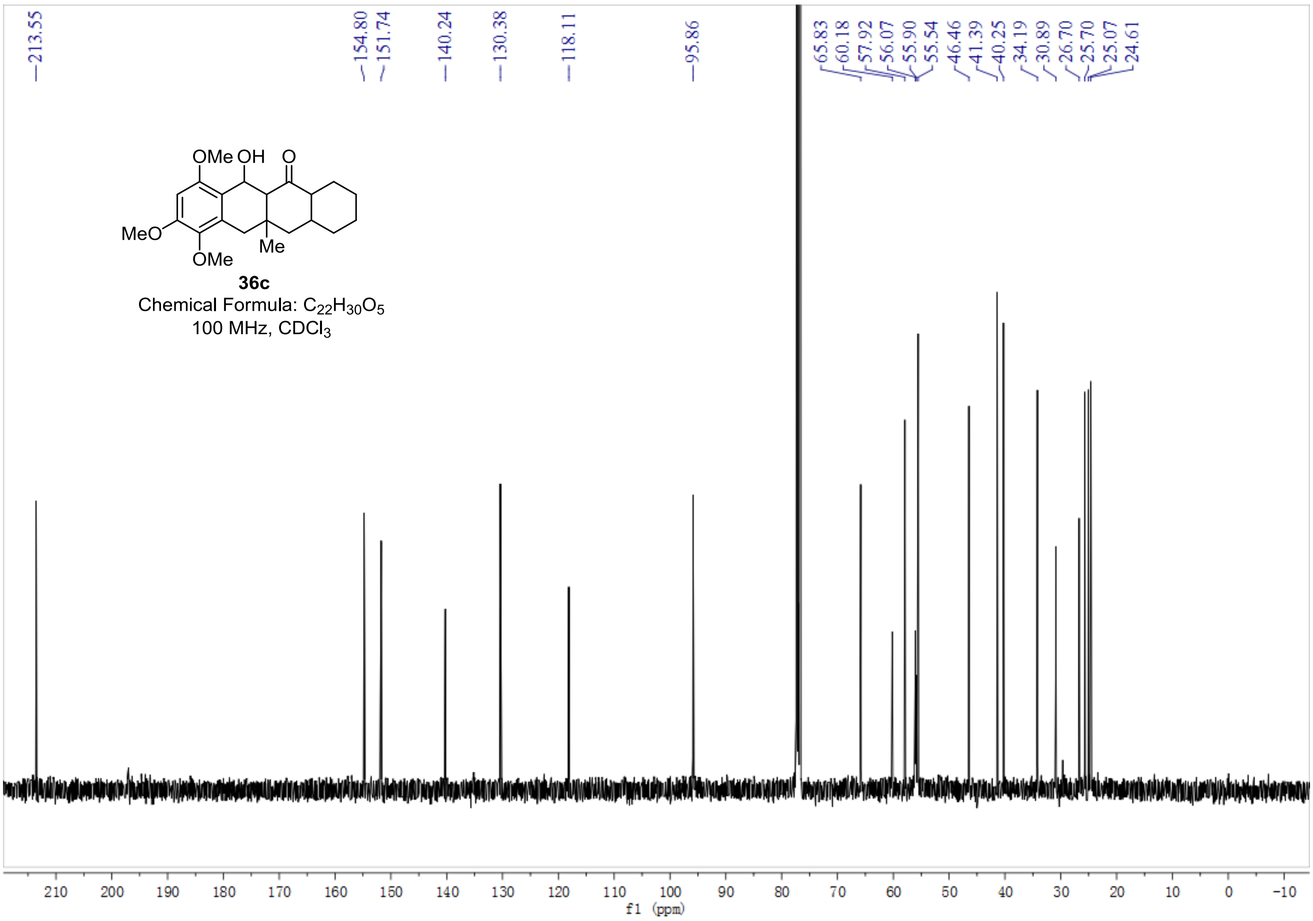

Supplementary Figure 22. ^{1}H **and** ^{13}C **NMR spectra for 36c.**

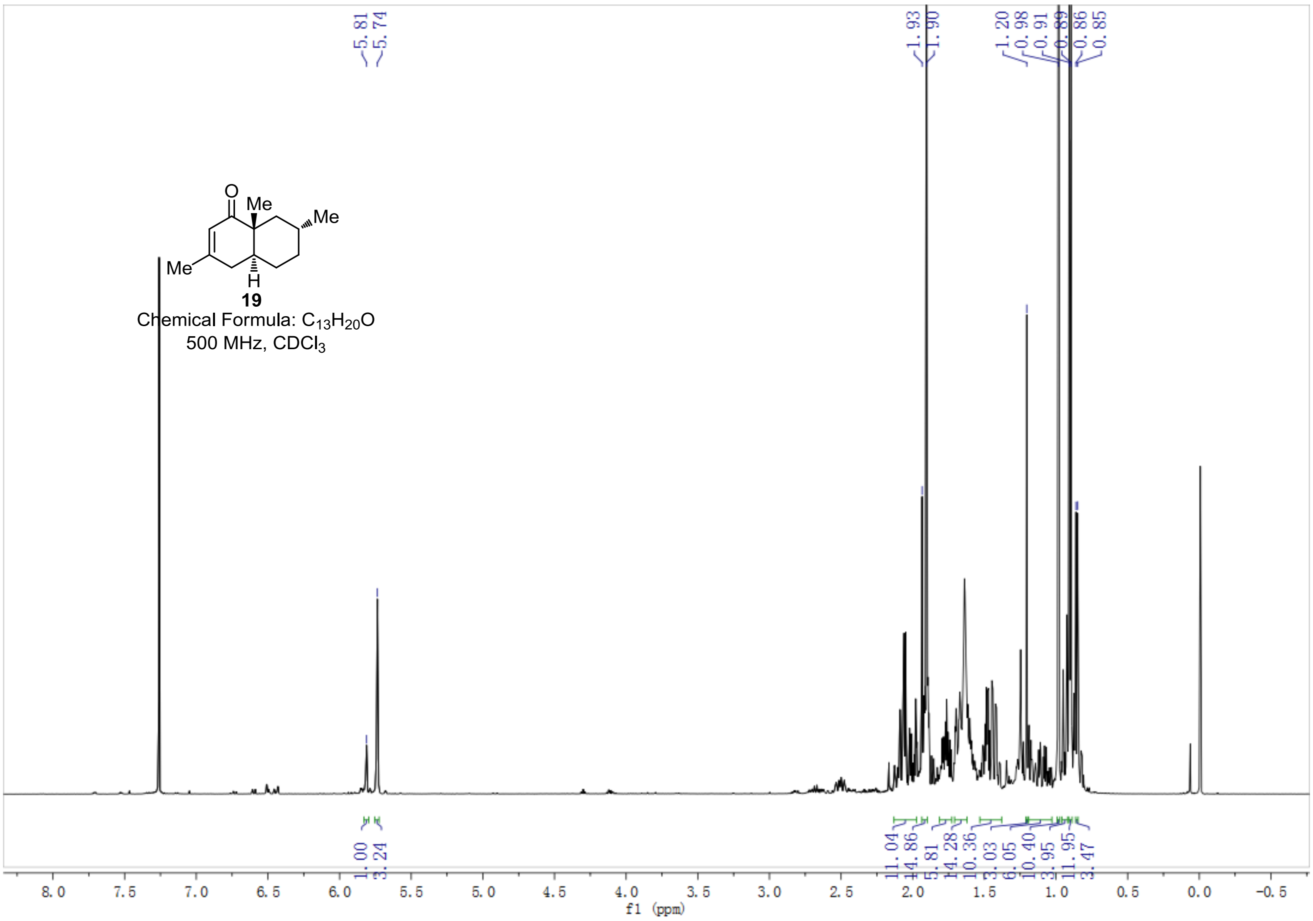

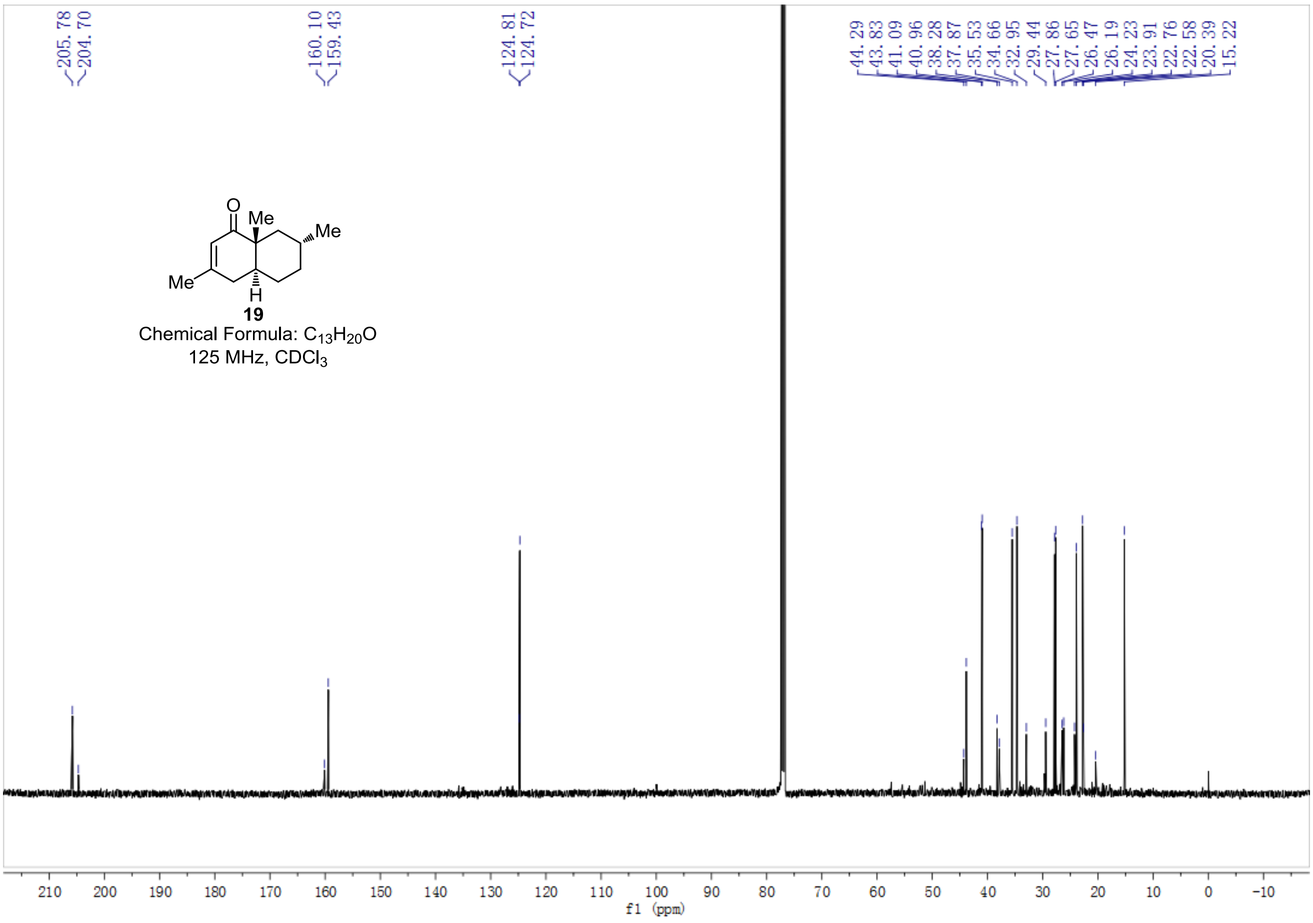

Supplementary Figure 23. ^{1}H **and** ^{13}C **NMR spectra for 19.**

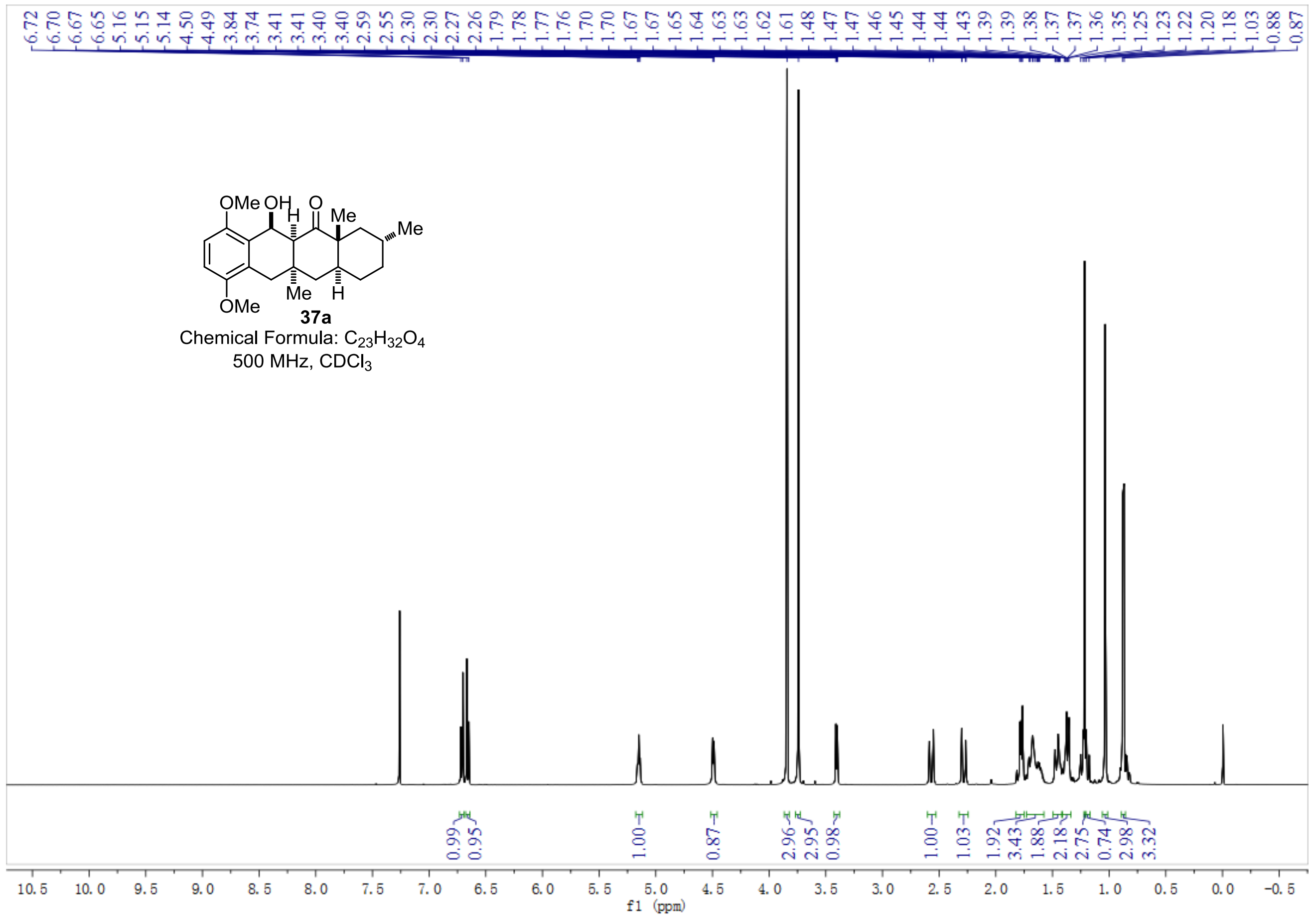

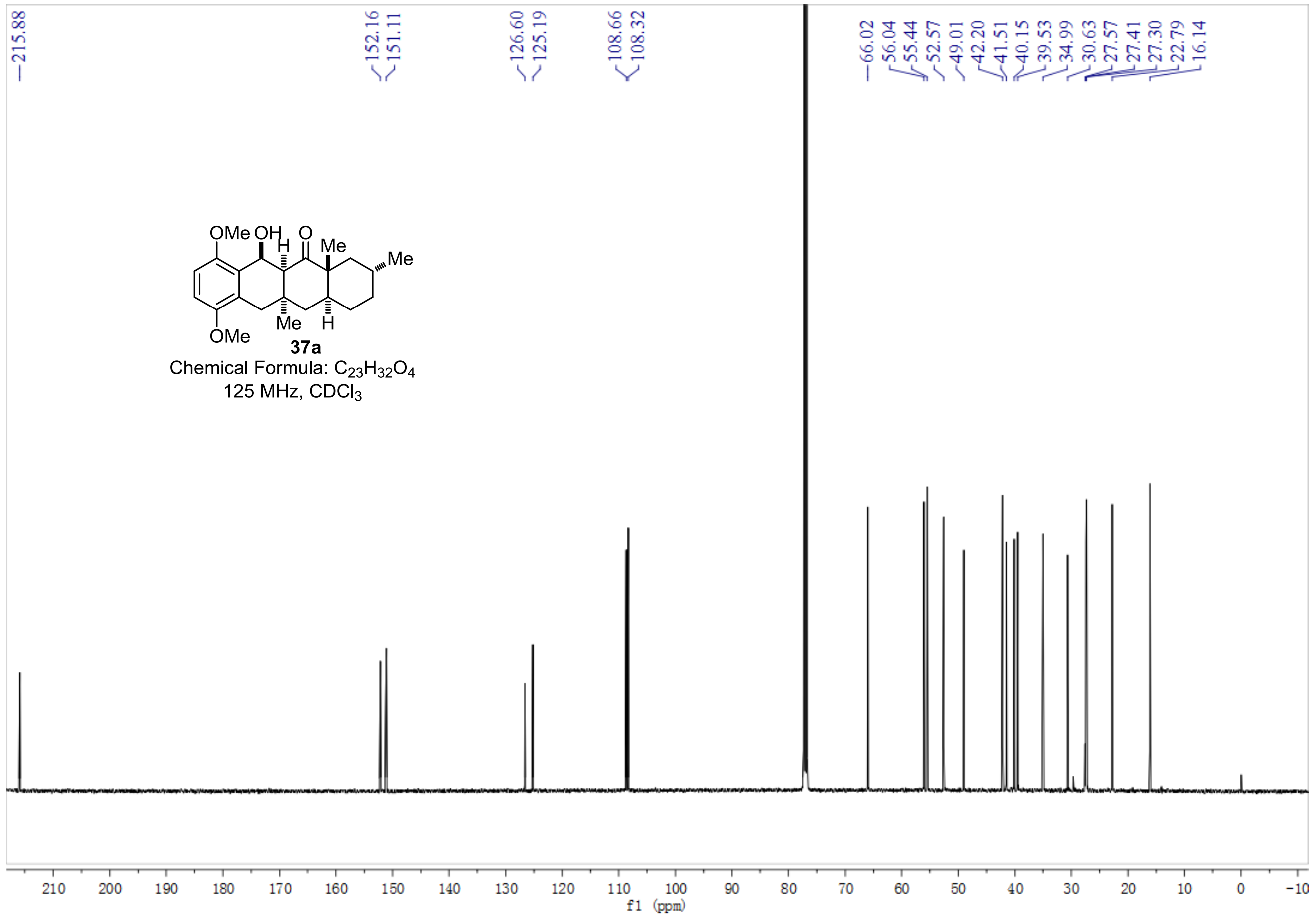

Supplementary Figure 24. ^{1}**H and** ^{13}**C NMR spectra for 37a.**

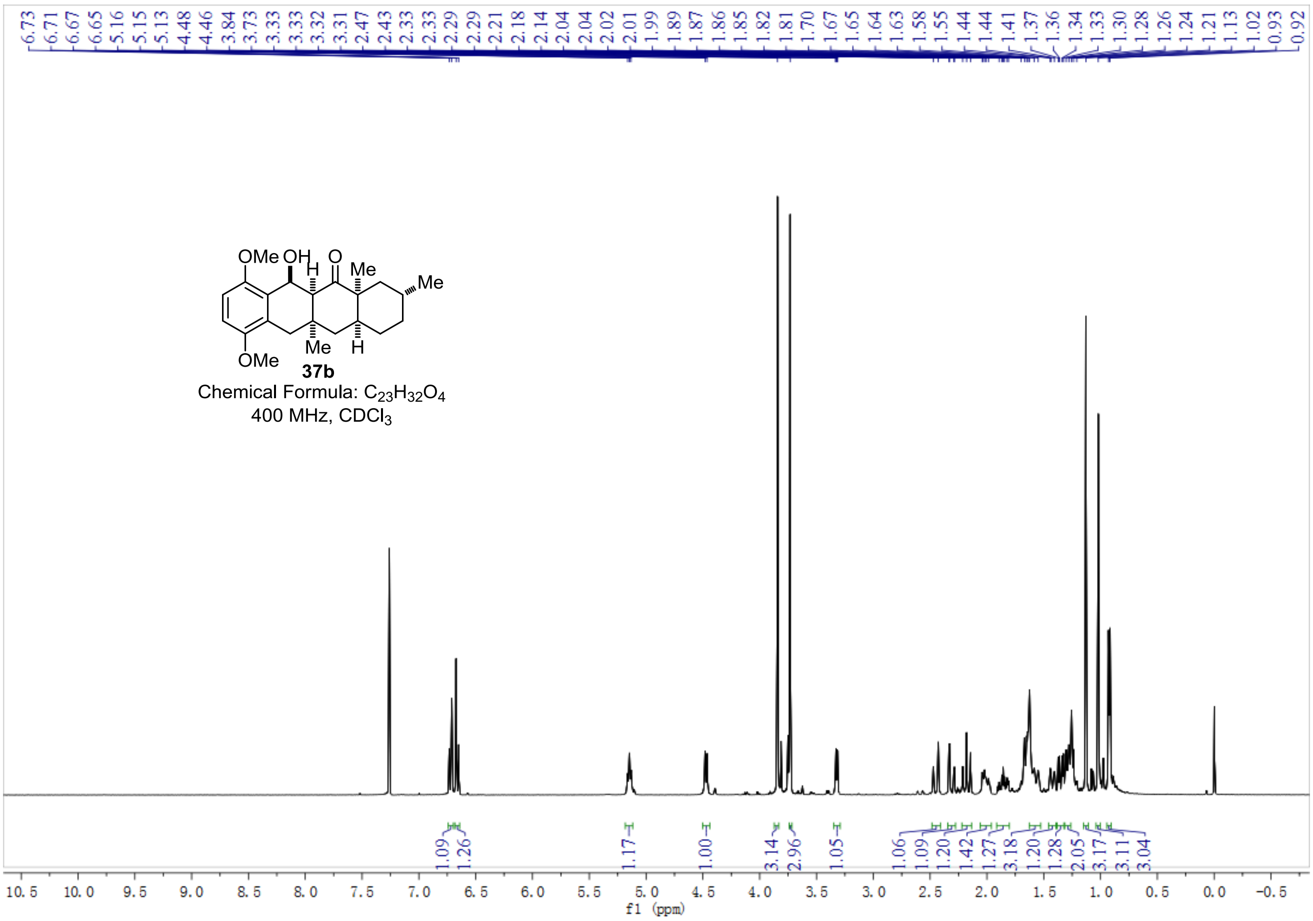

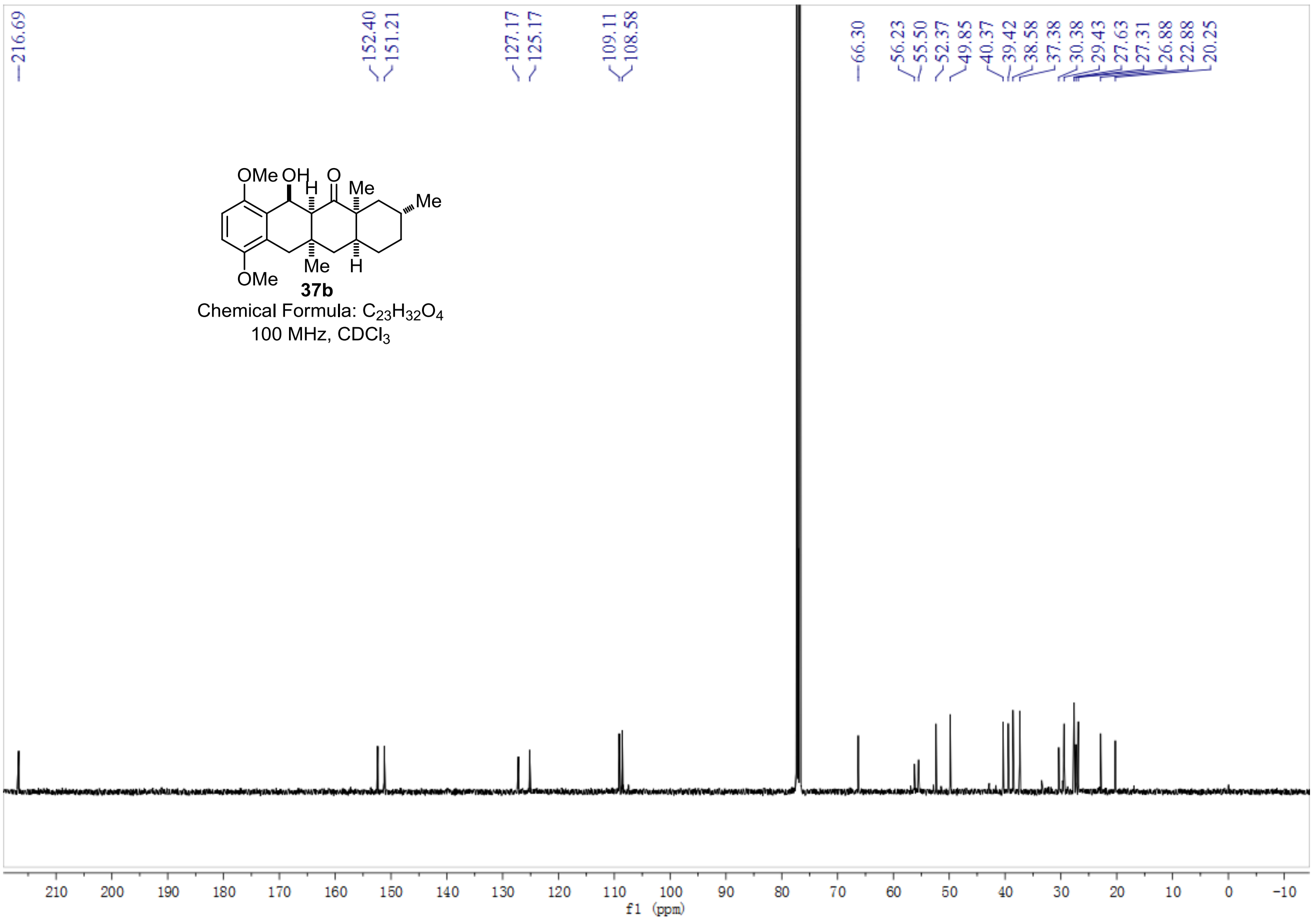

Supplementary Figure 25. ^{1}H **and** ^{13}C **NMR spectra for 37b.**

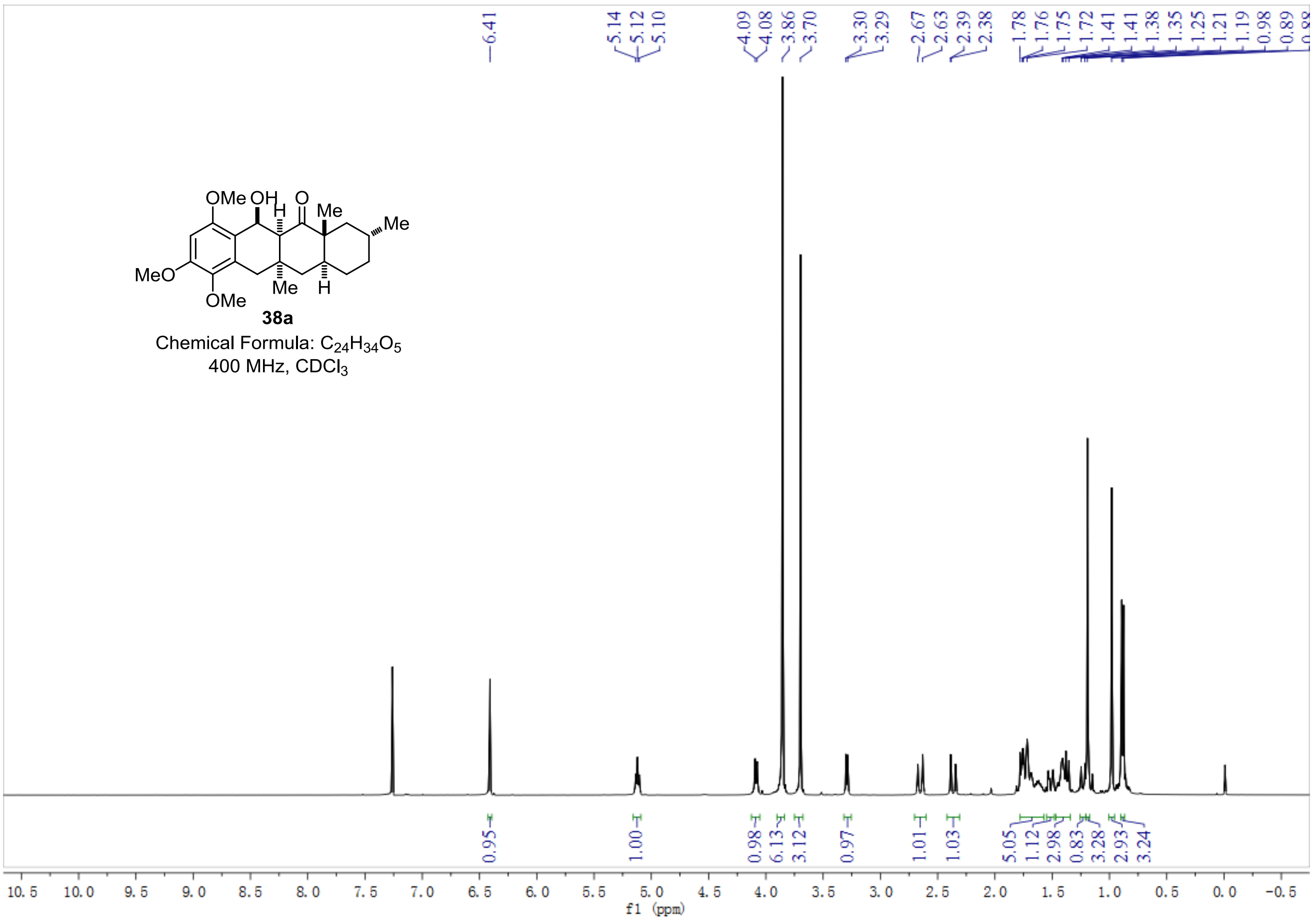

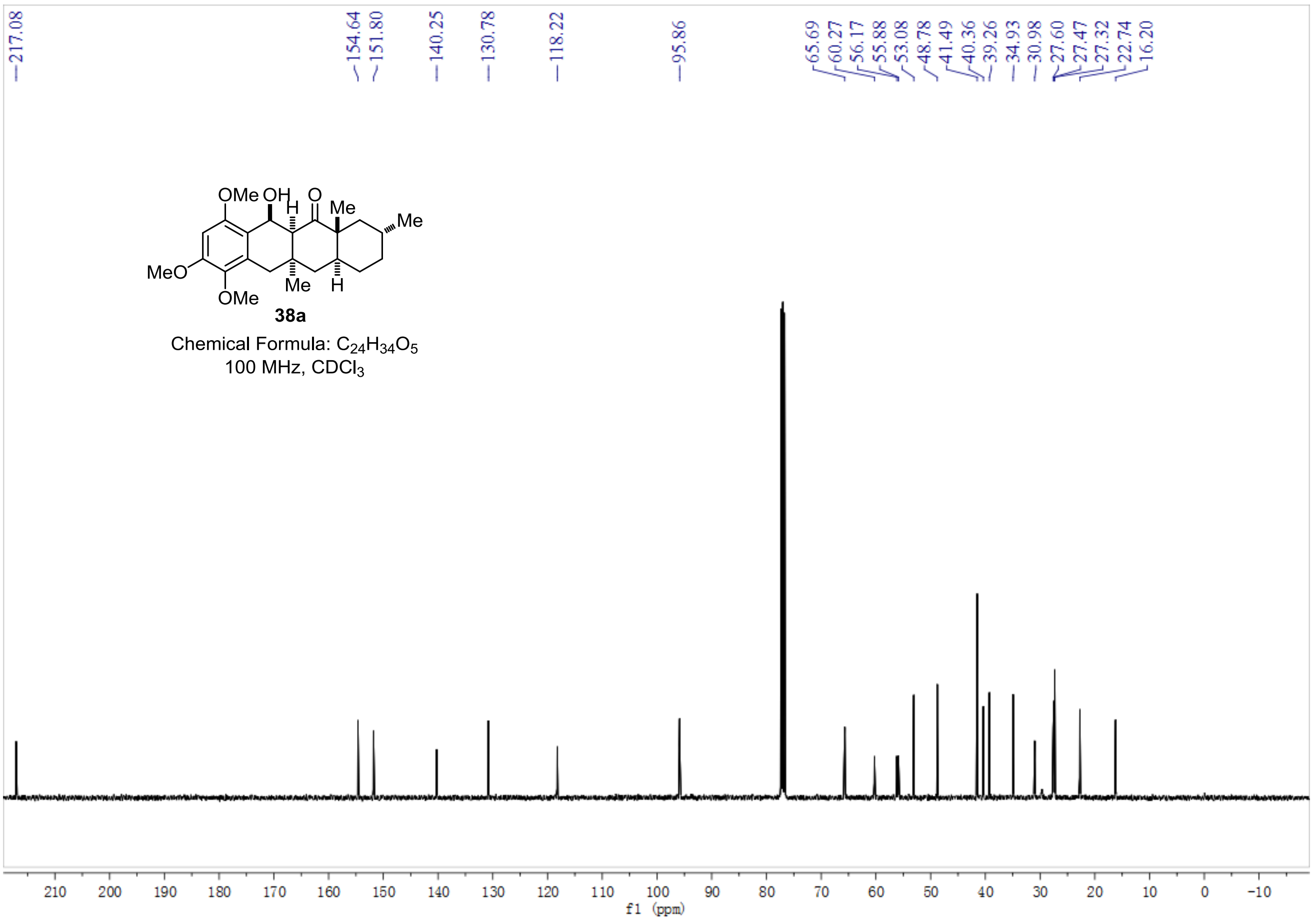

Supplementary Figure 26. ^{1}H and ^{13}C NMR spectra for 38a.

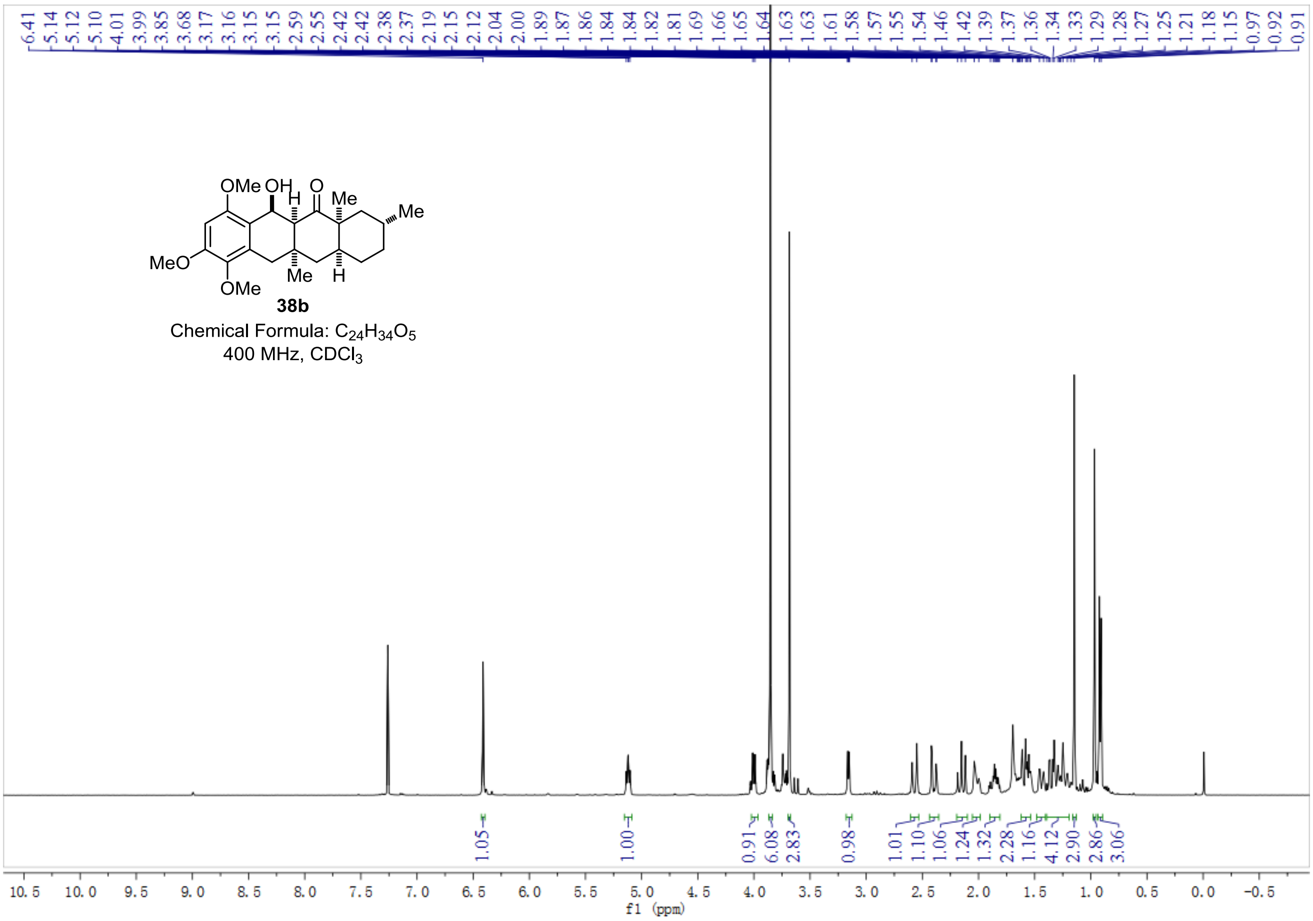

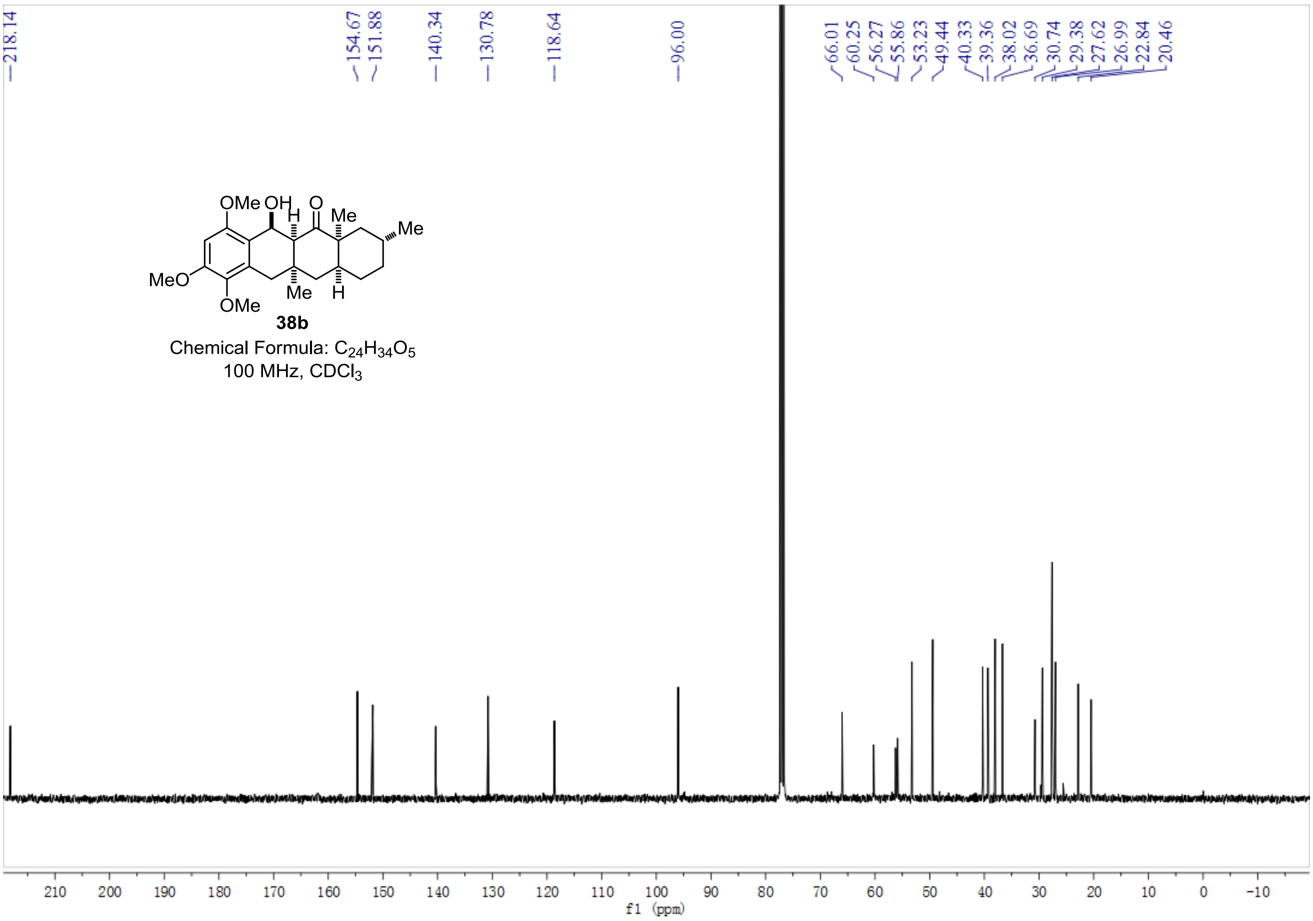

Supplementary Figure 27.^{1}H and ^{13}C NMR spectra for 38b.

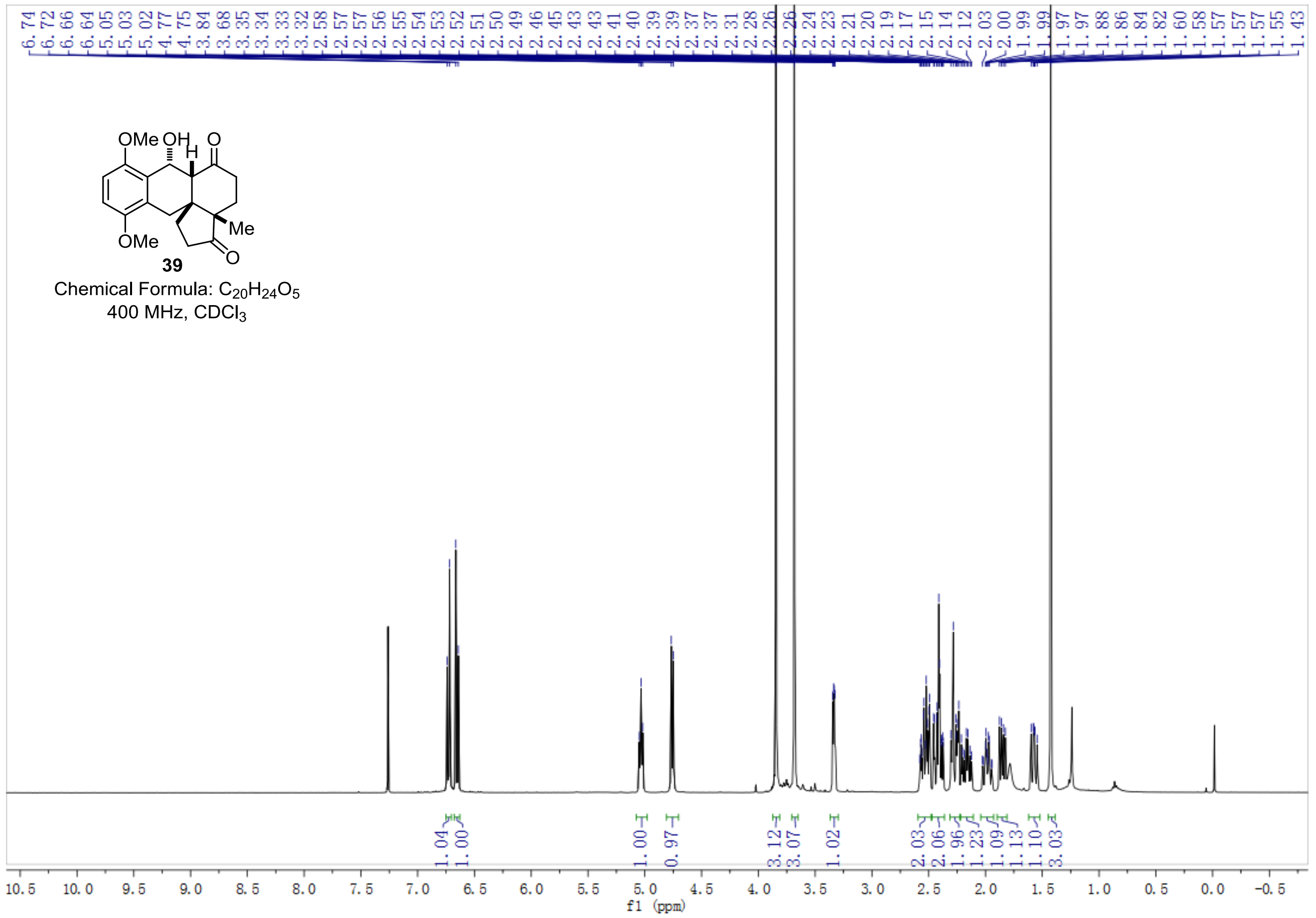

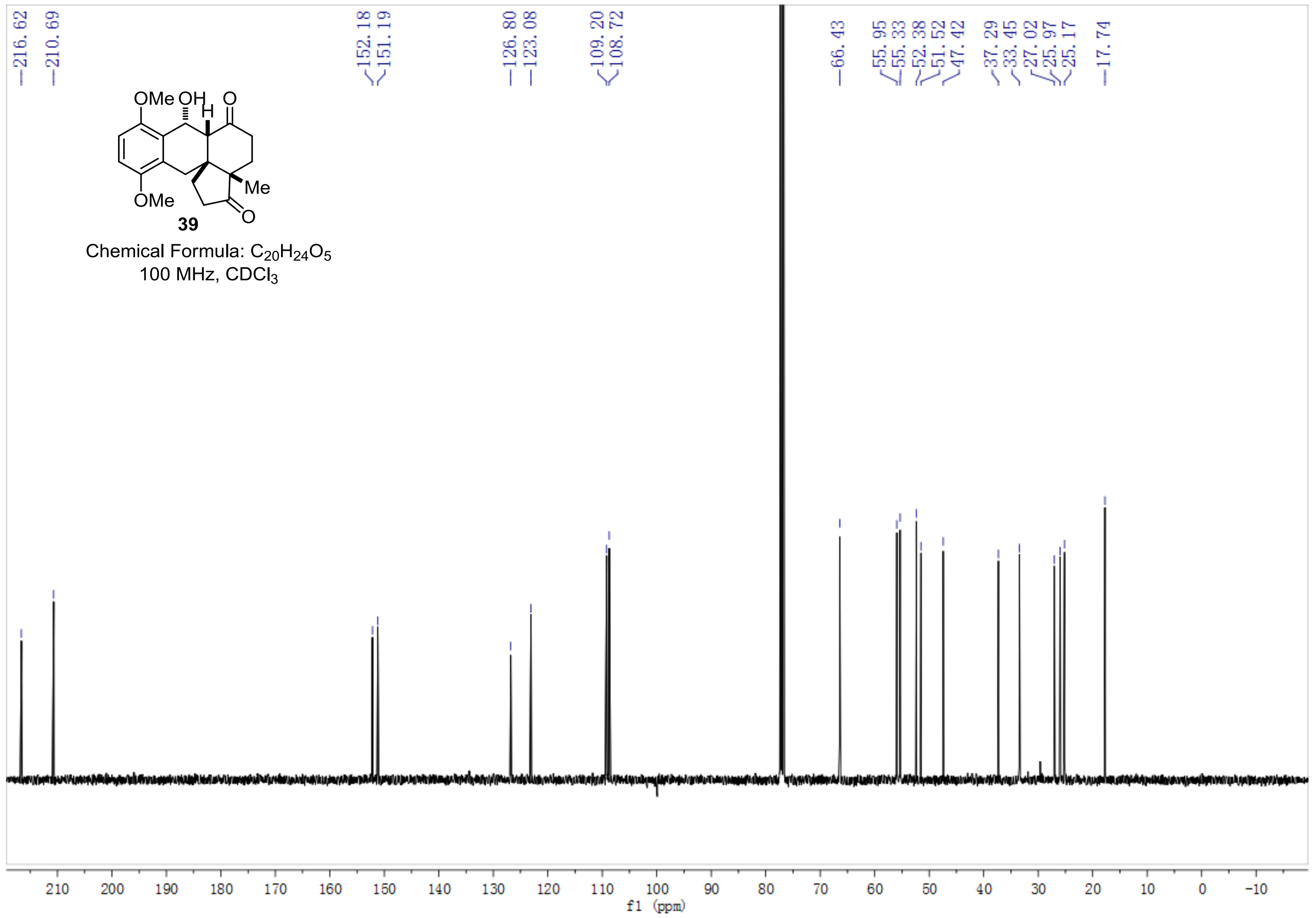

Supplementary Figure 28.^{1}H and ^{13}C NMR spectra for 39.

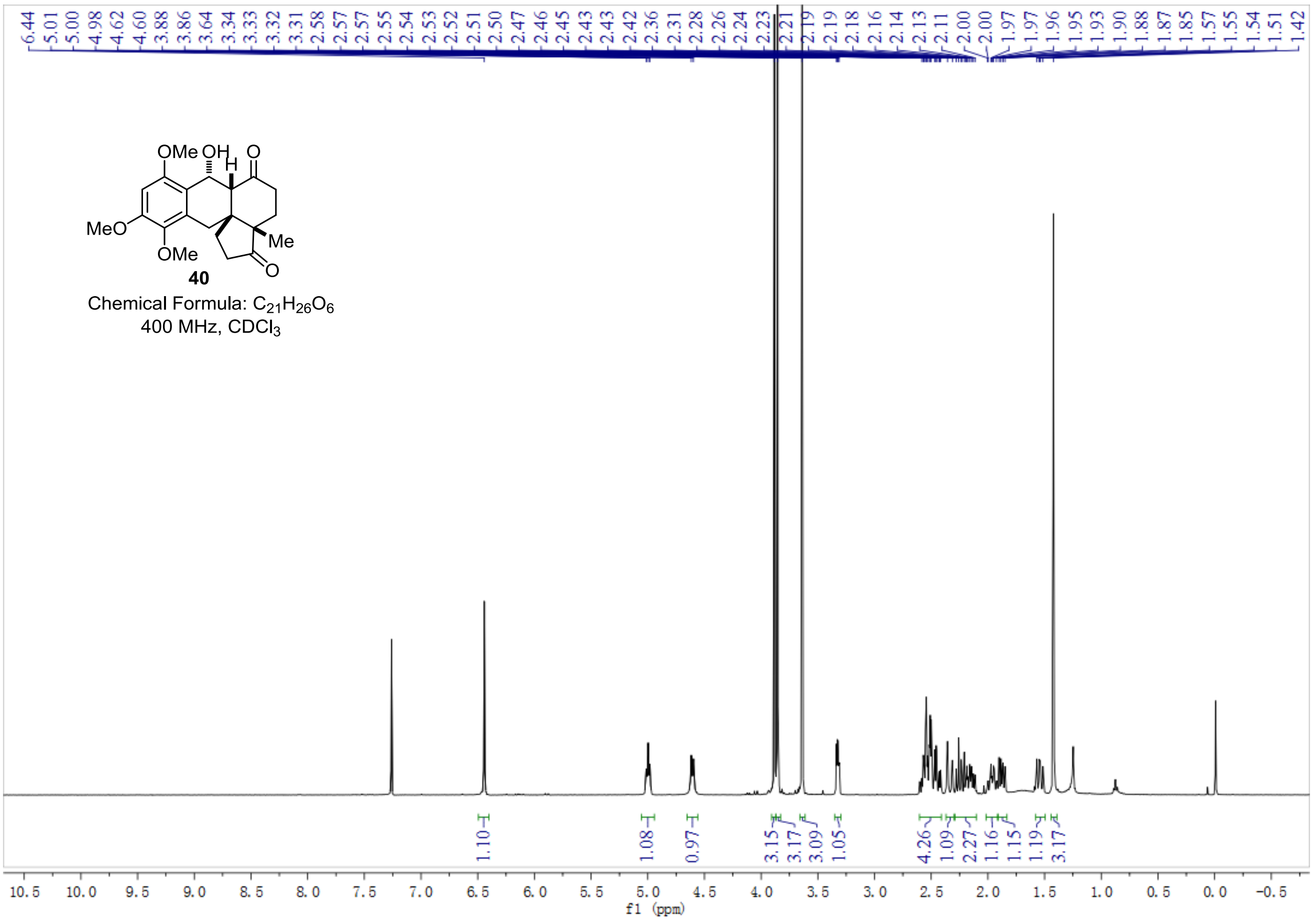

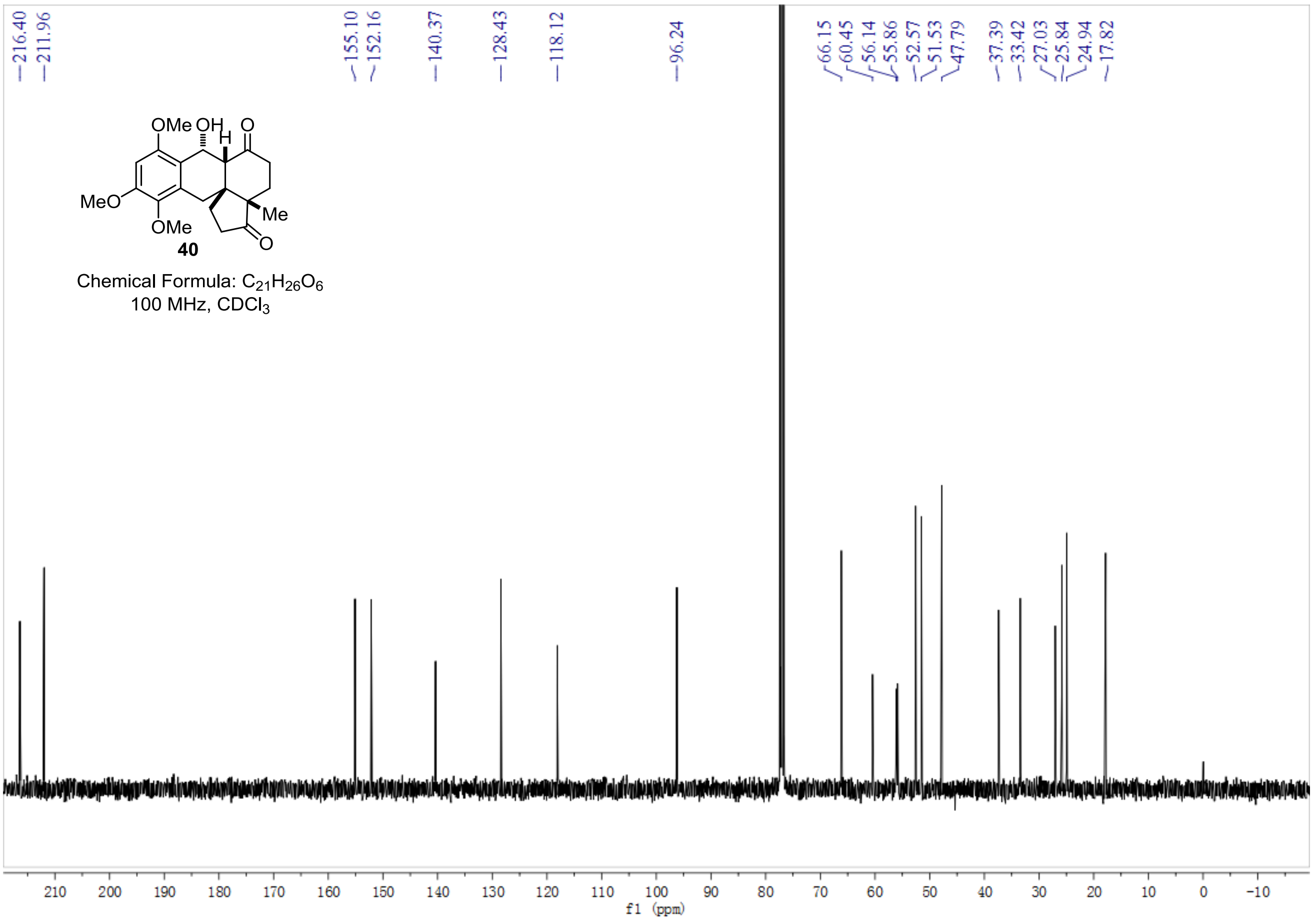

Supplementary Figure 29.^{1}H and ^{13}C NMR spectra for 40.

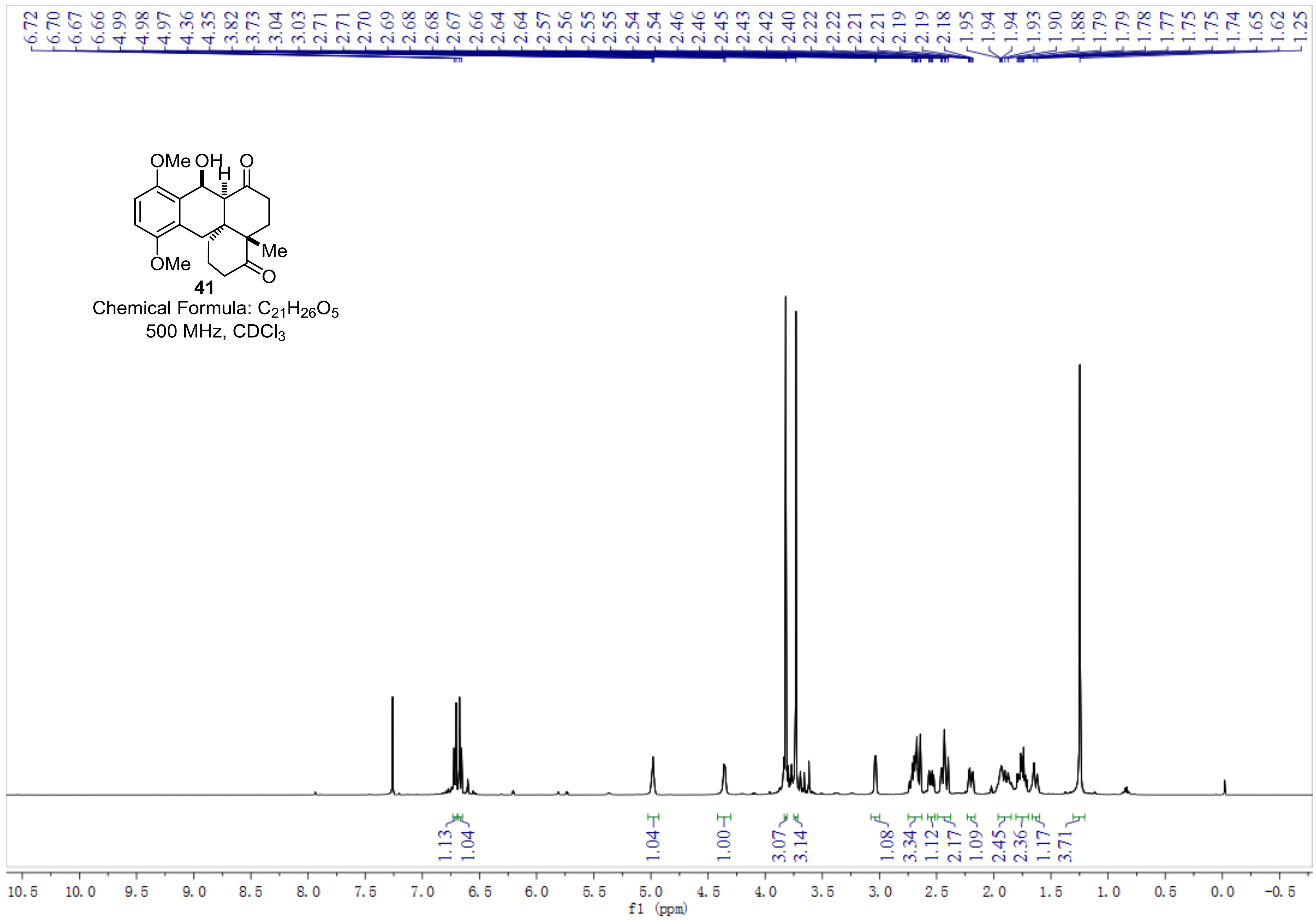

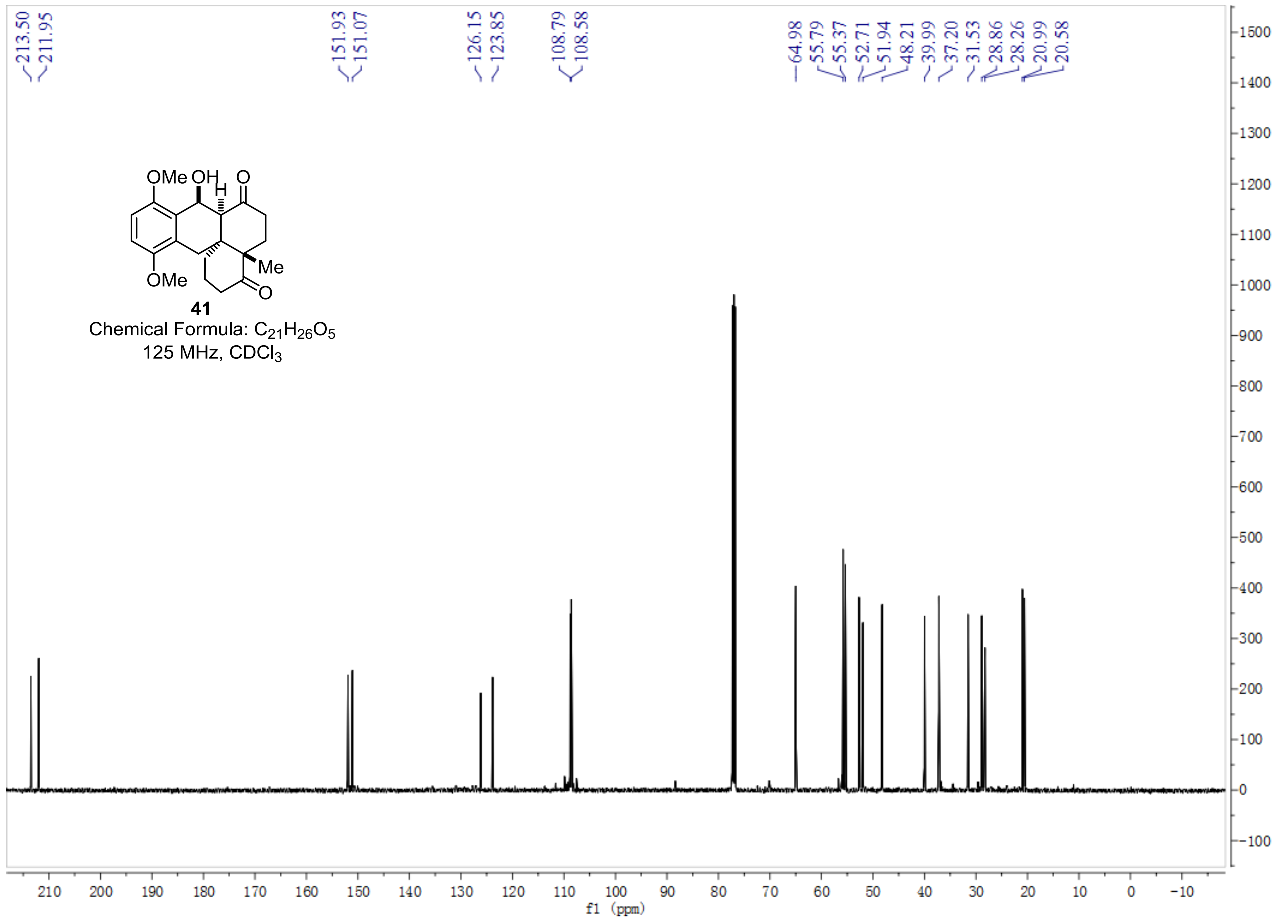

Supplementary Figure 30. ^{1}H **and** ^{13}C **NMR spectra for 41.**

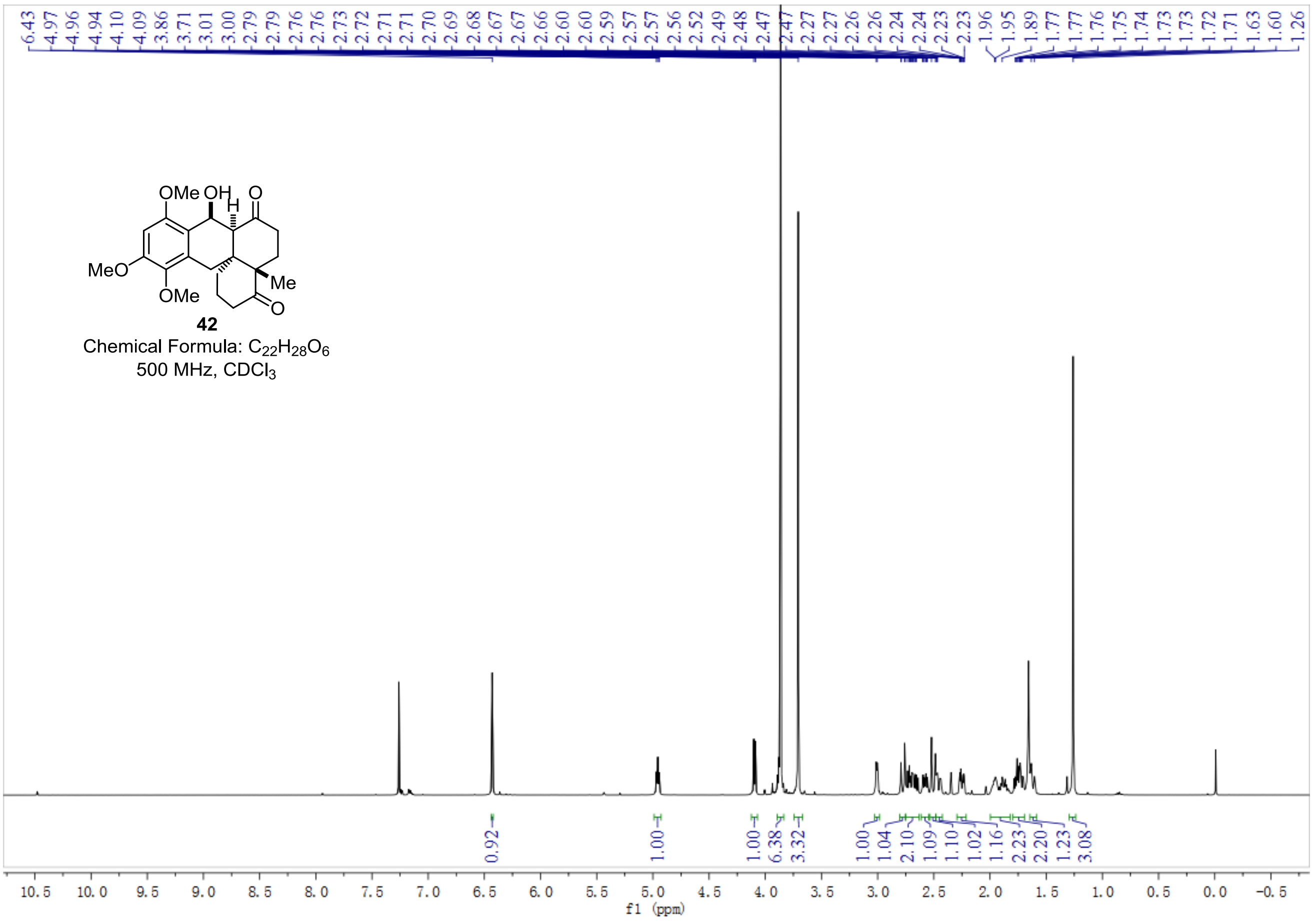

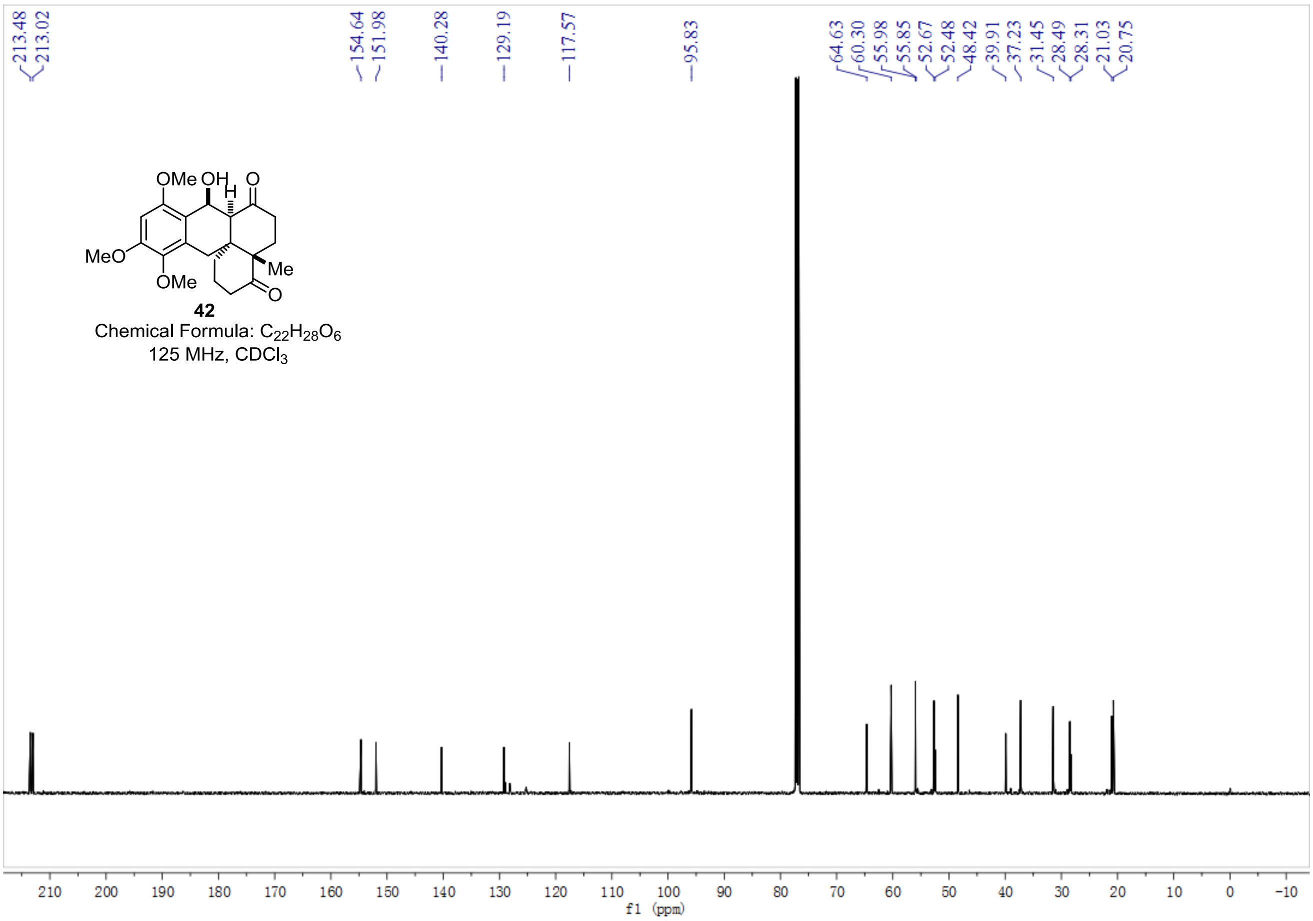

Supplementary Figure 31.^{1}H and ^{13}C NMR spectra for 42.

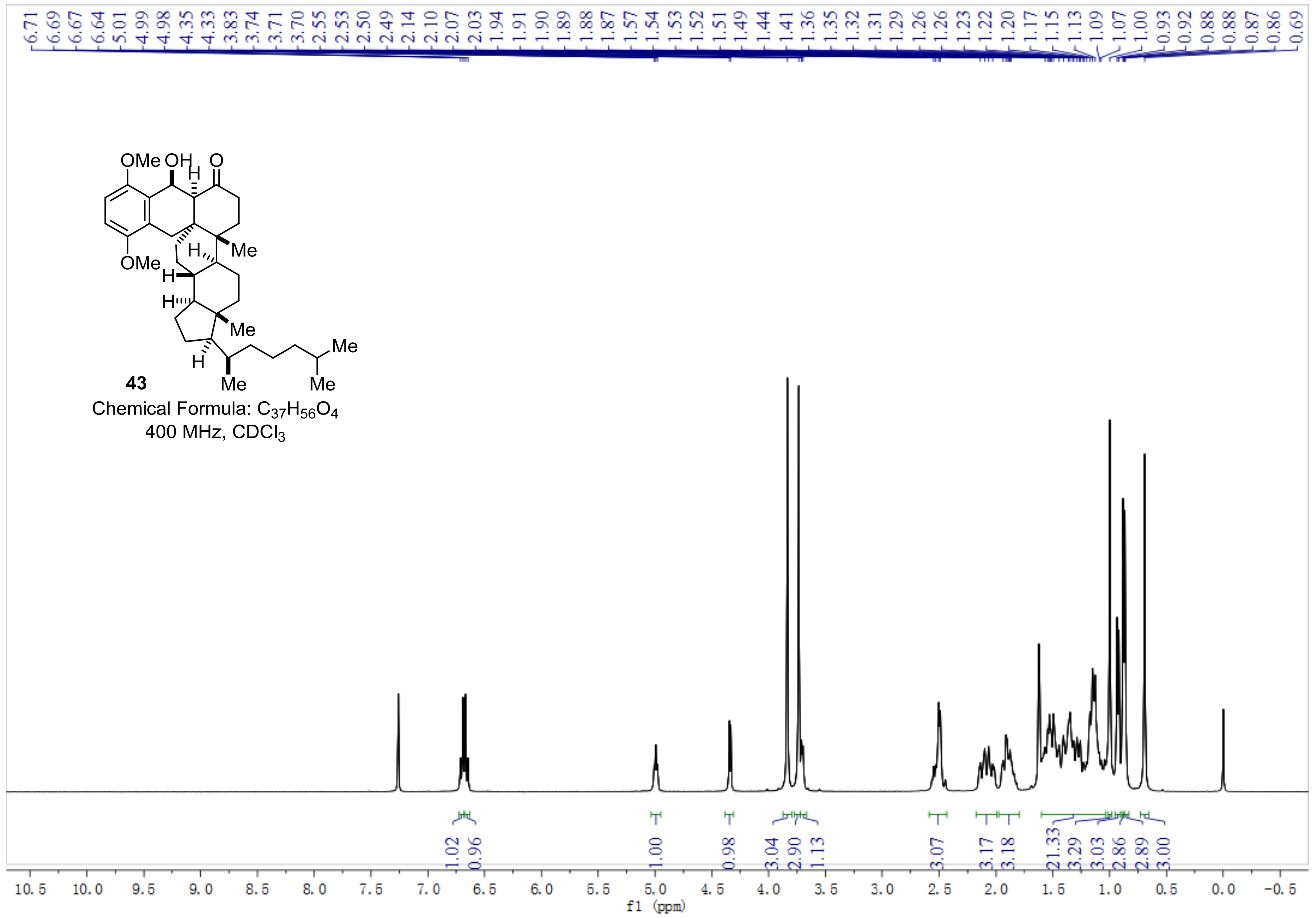

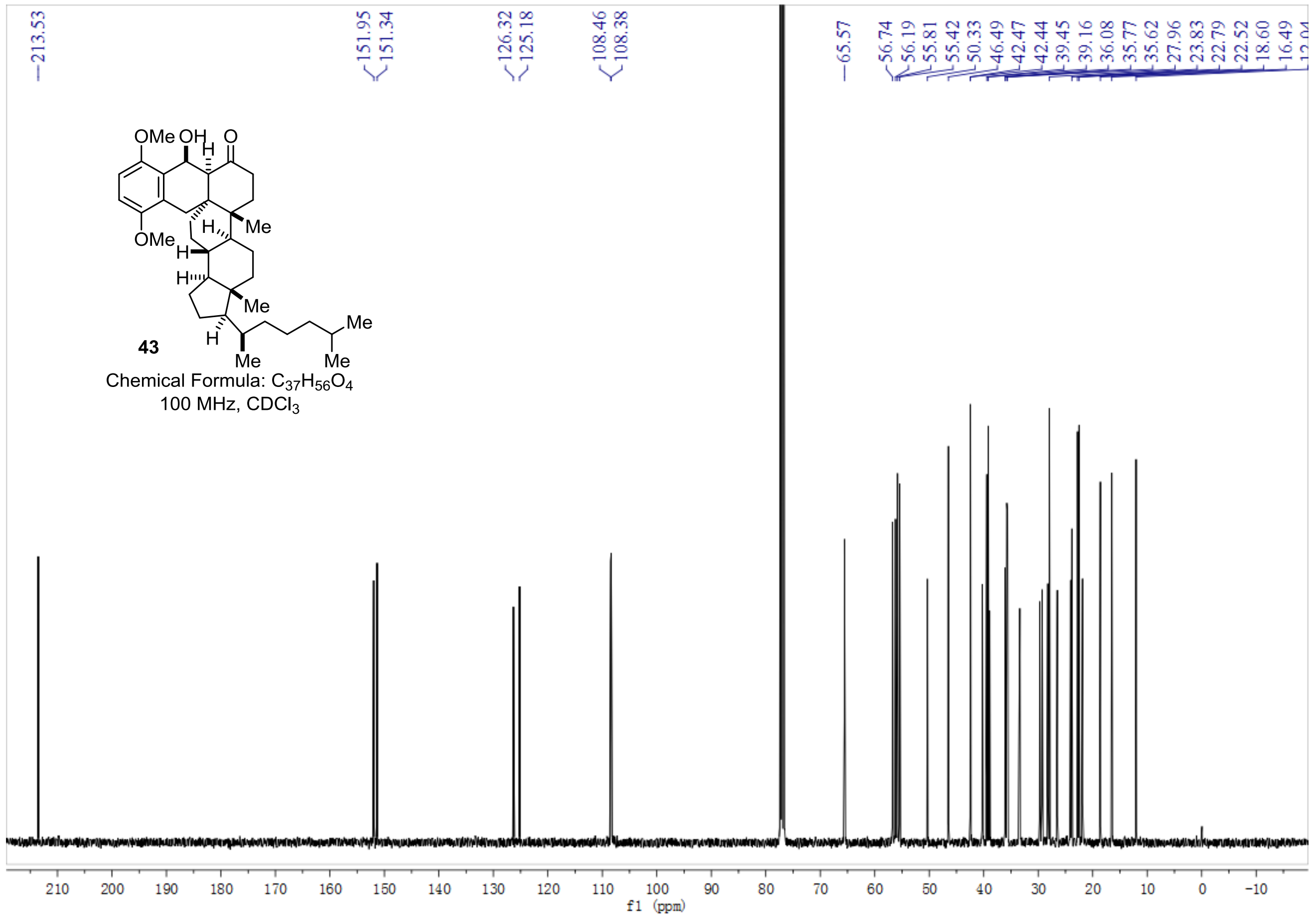

Supplementary Figure 32.^{1}H and ^{13}C NMR spectra for 43.

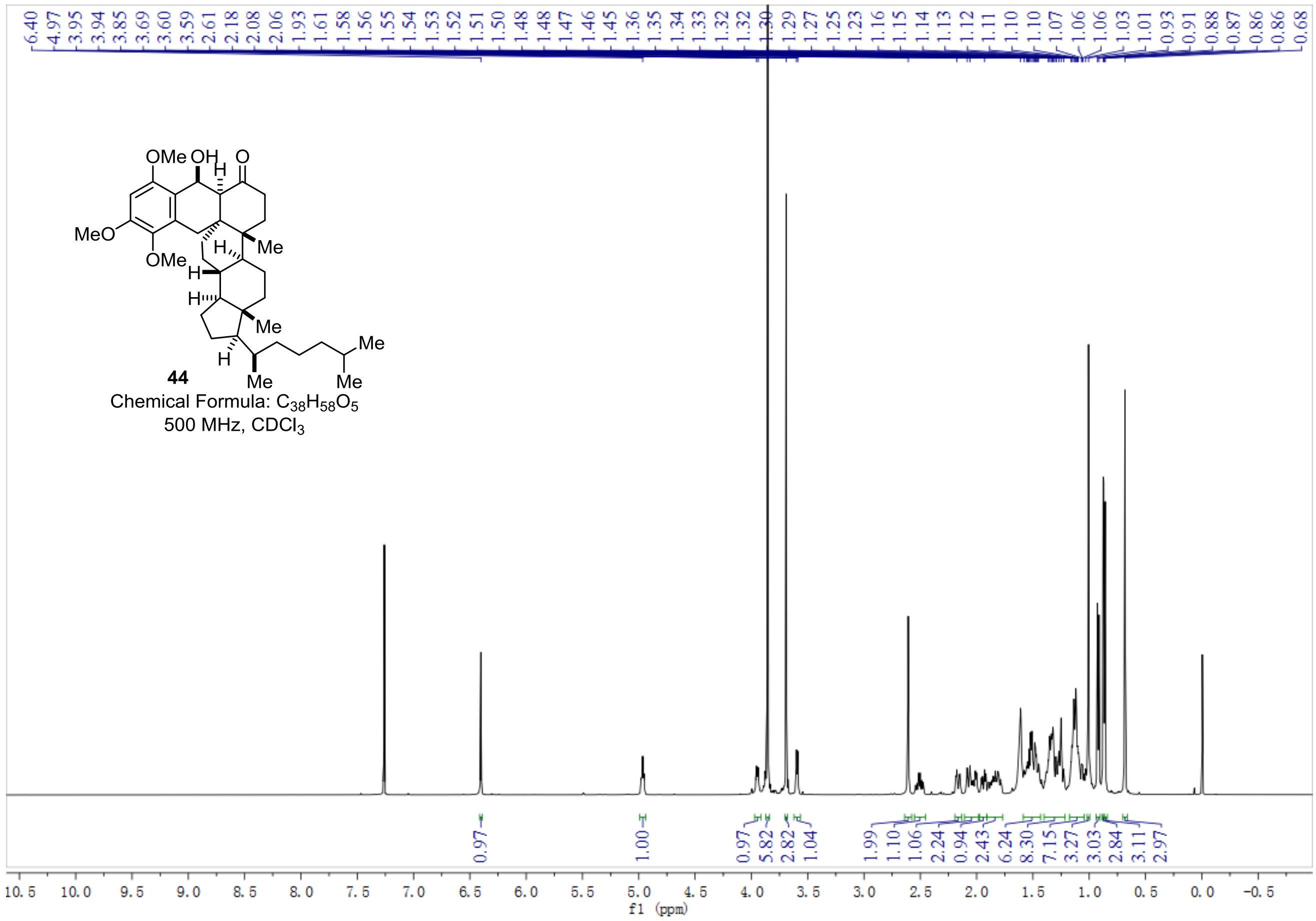

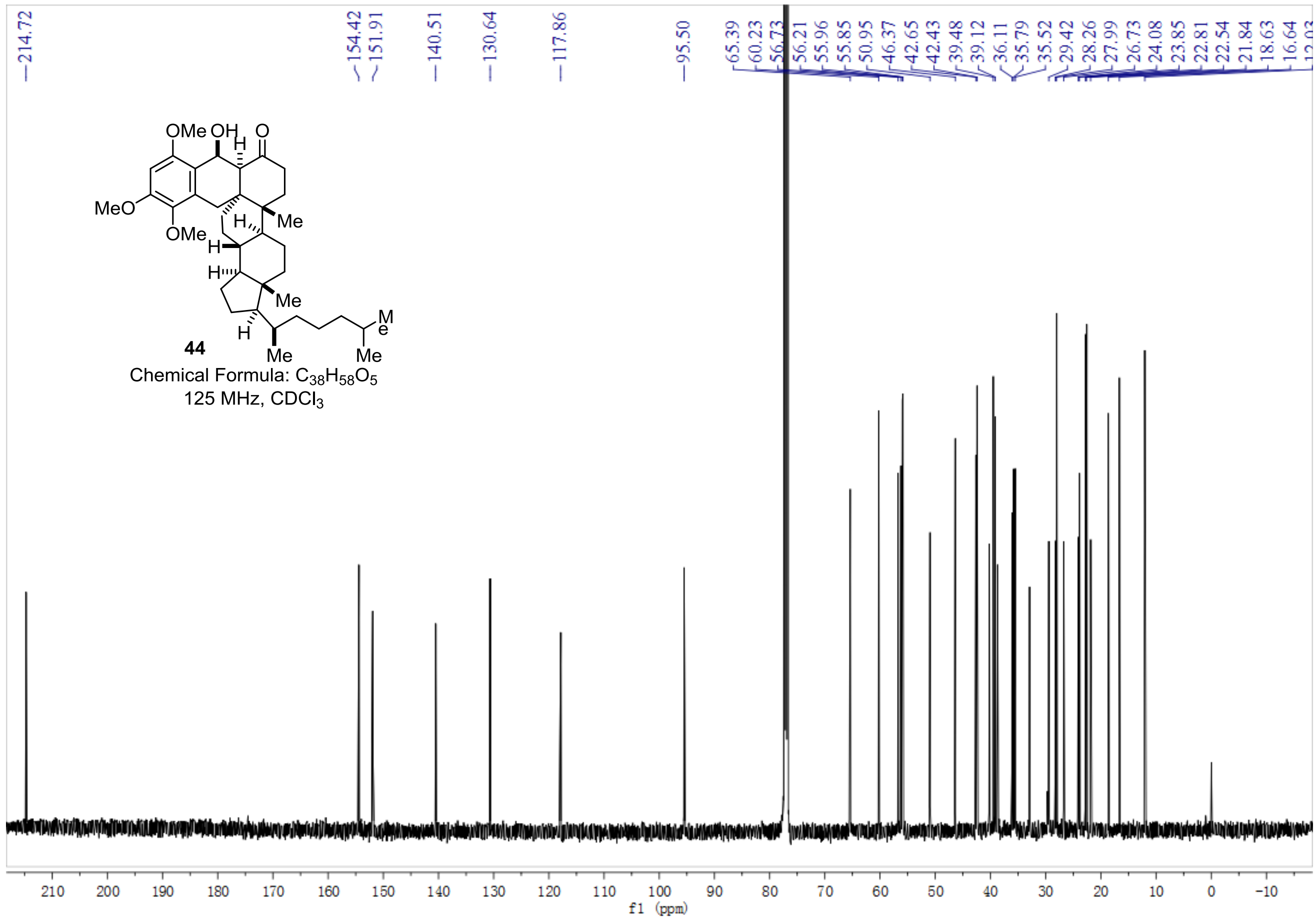

Supplementary Figure 33. ^{1}H and ^{13}C NMR spectra for 44.

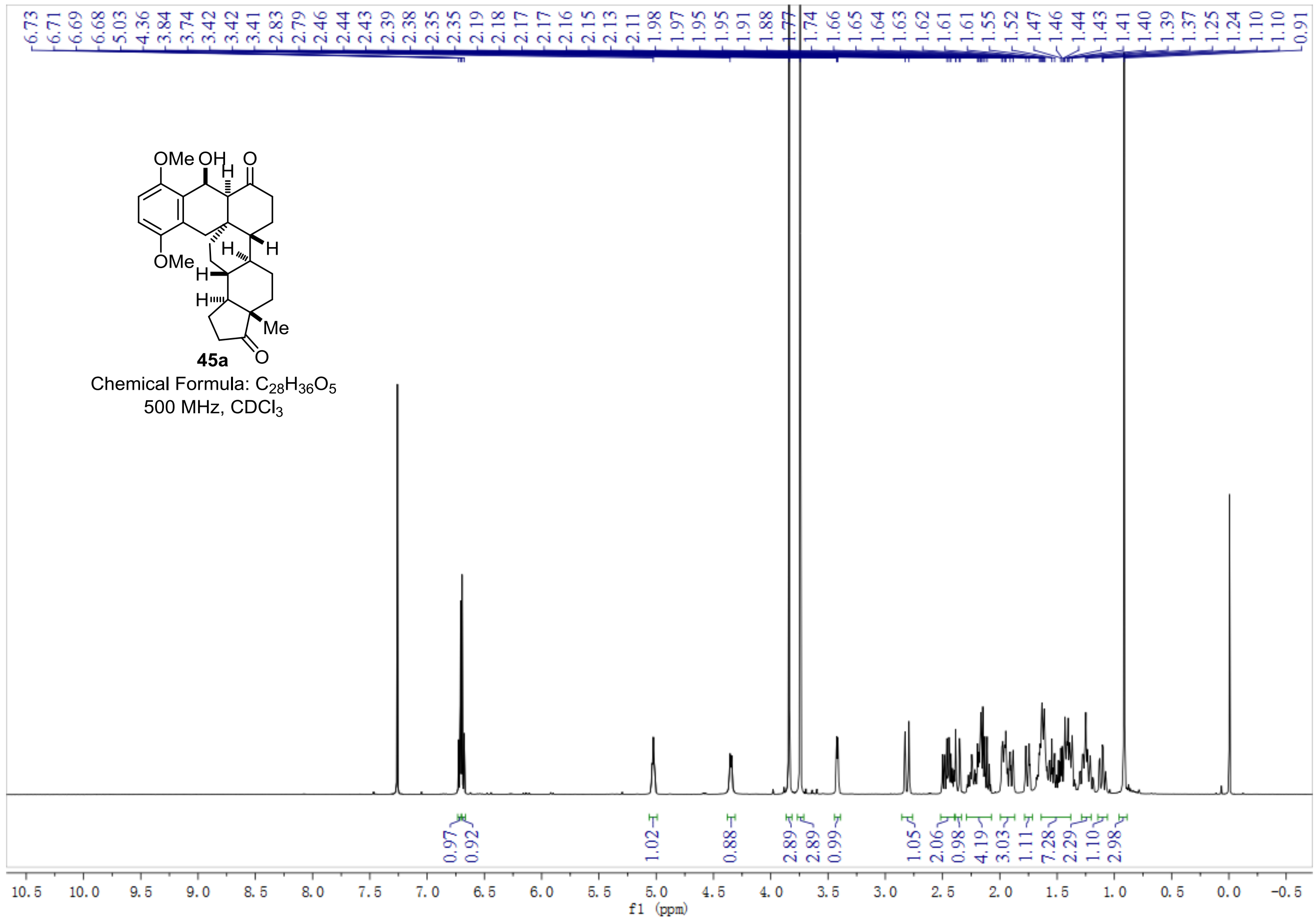

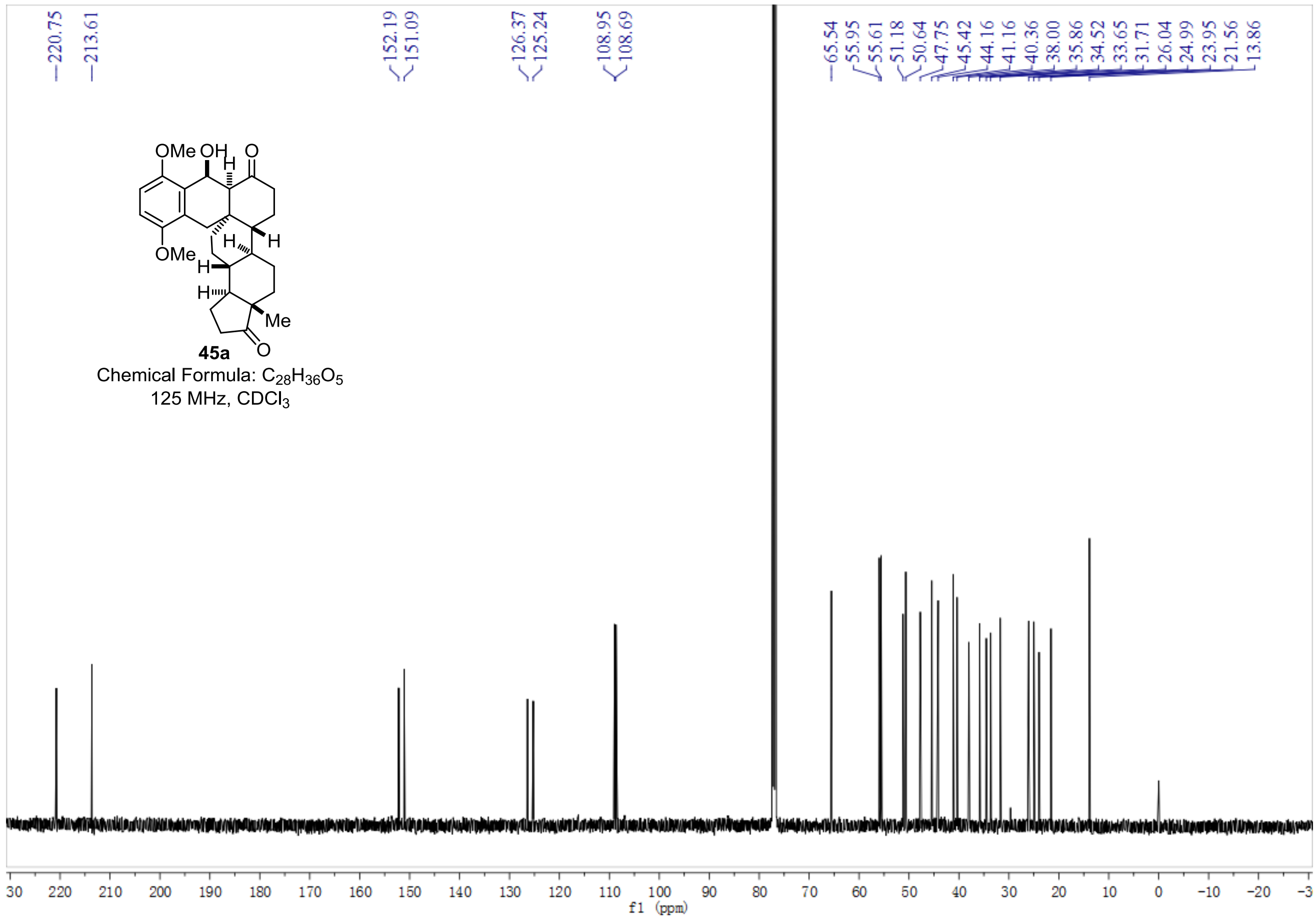

Supplementary Figure 34. ¹H and ¹³C NMR spectra for 45a.

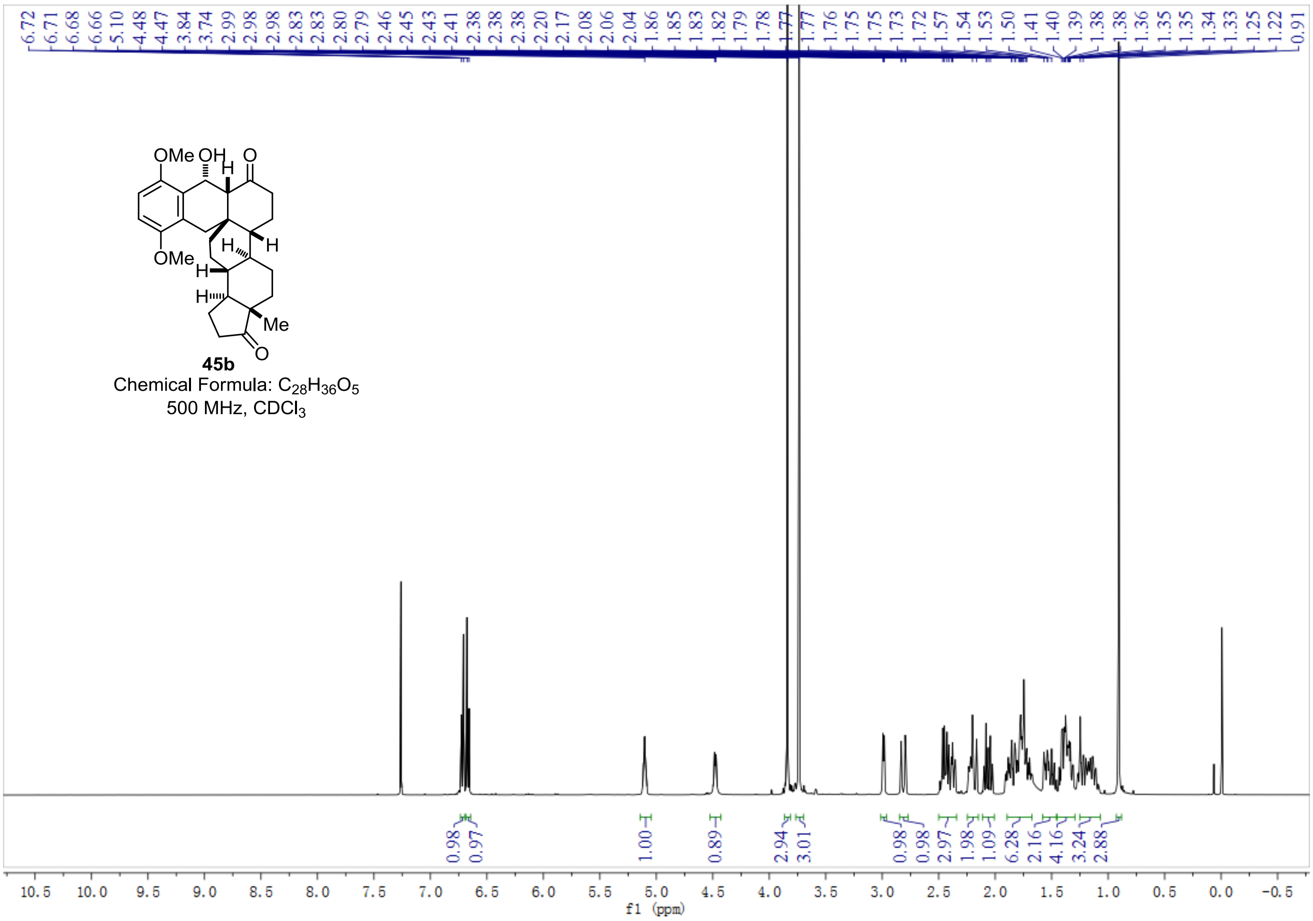

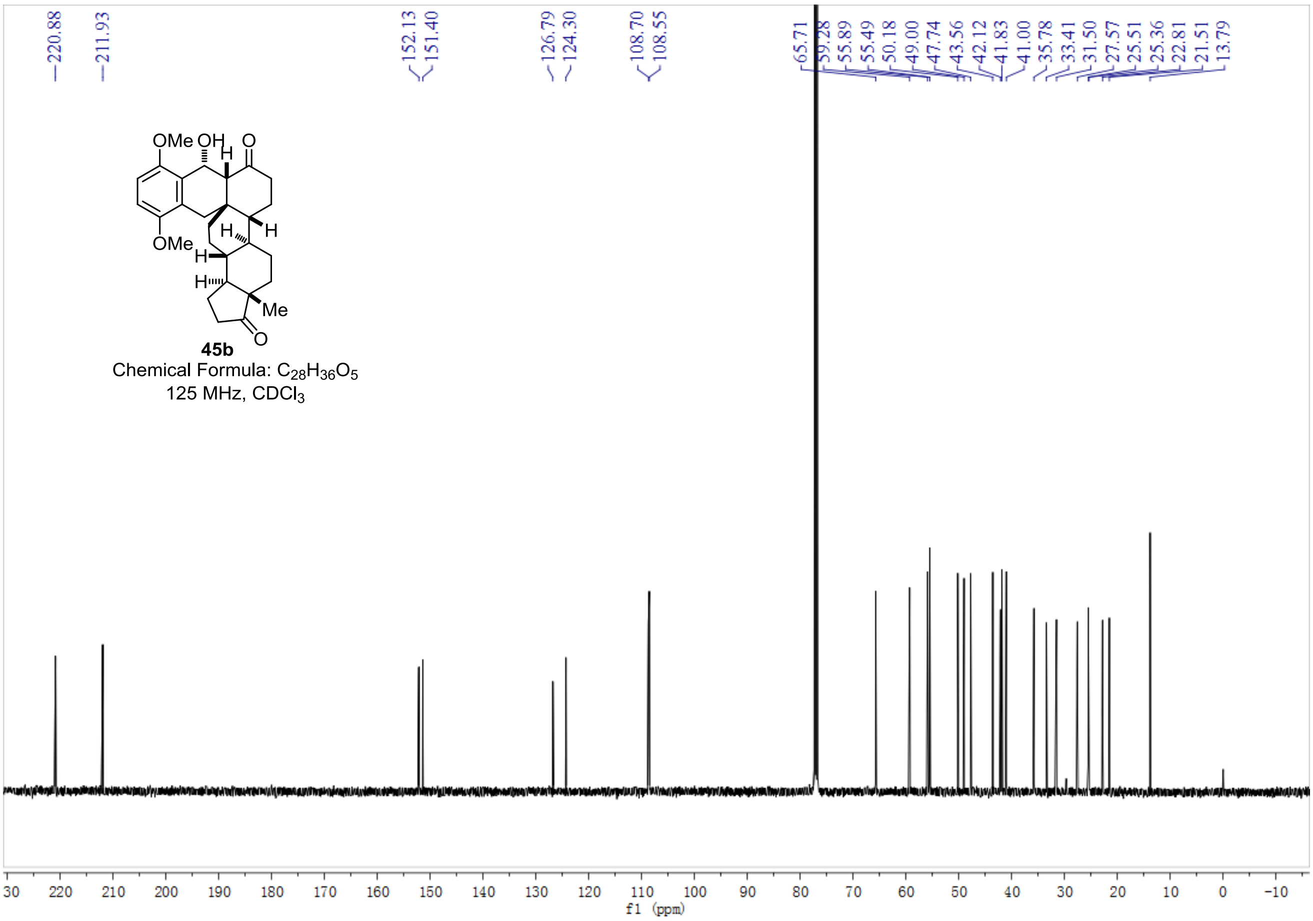

Supplementary Figure 35. ^{1}H **and** ^{13}C **NMR spectra for 45b.**

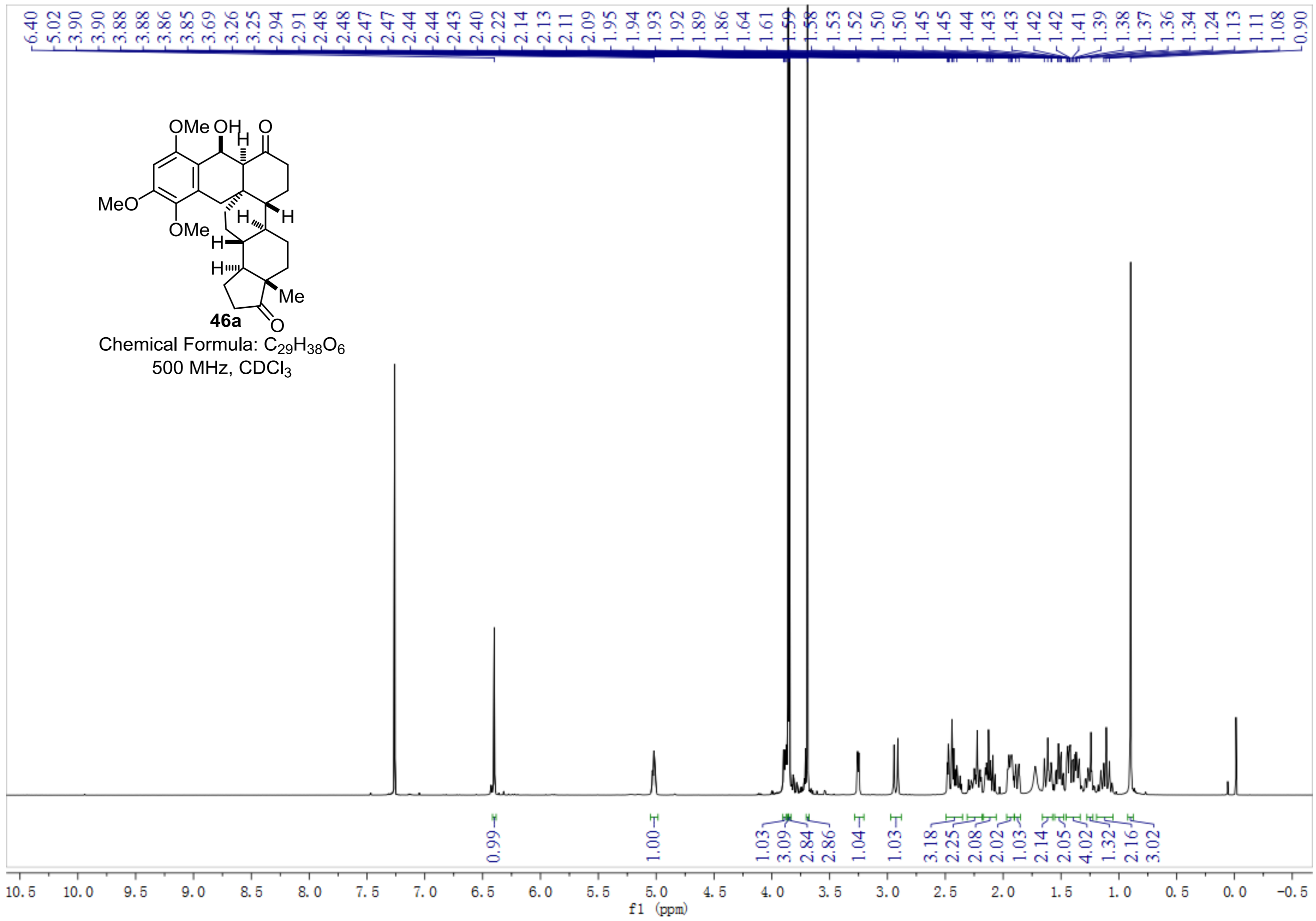

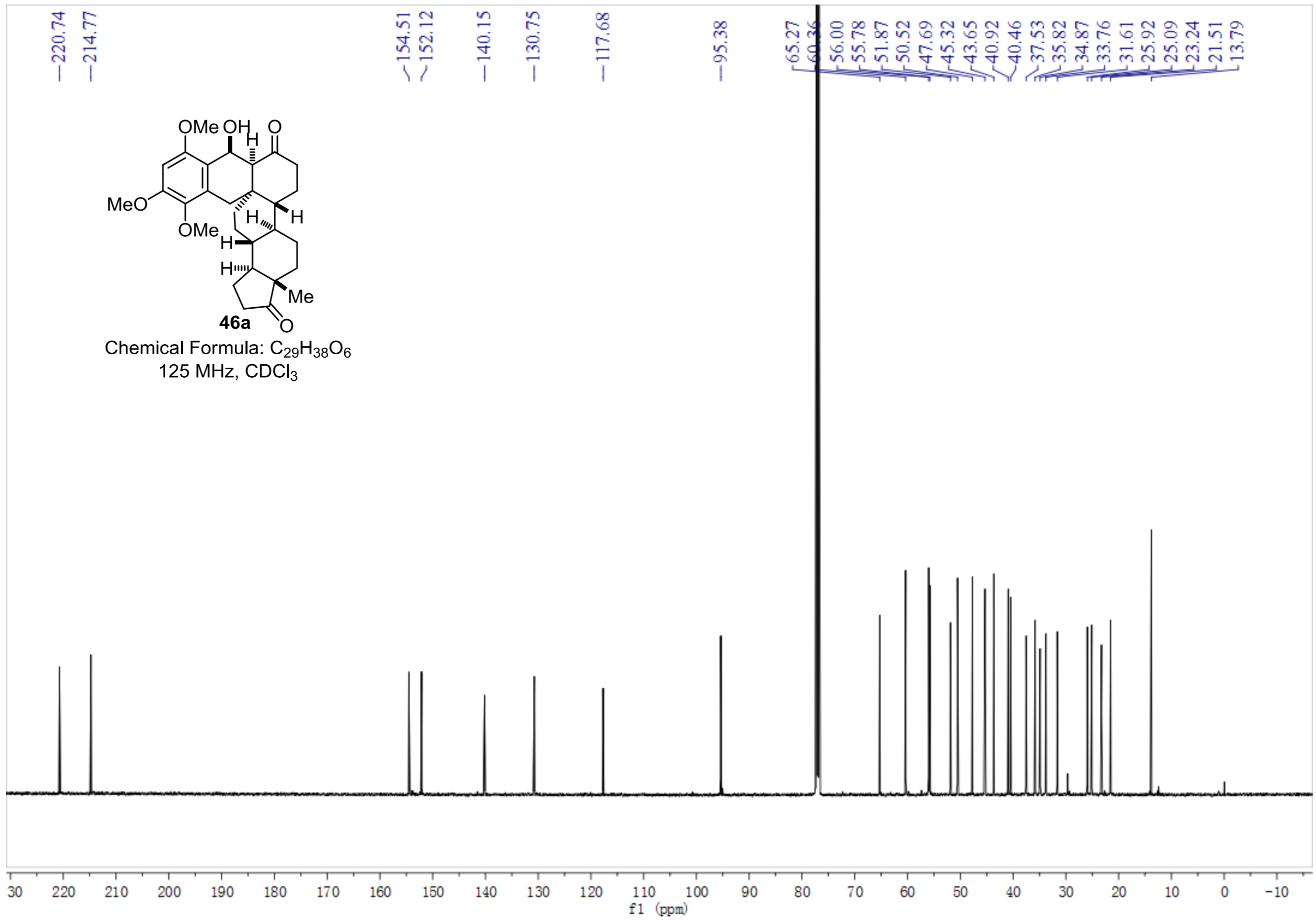

Supplementary Figure 36.^{1}H and ^{13}C NMR spectra for 46a.

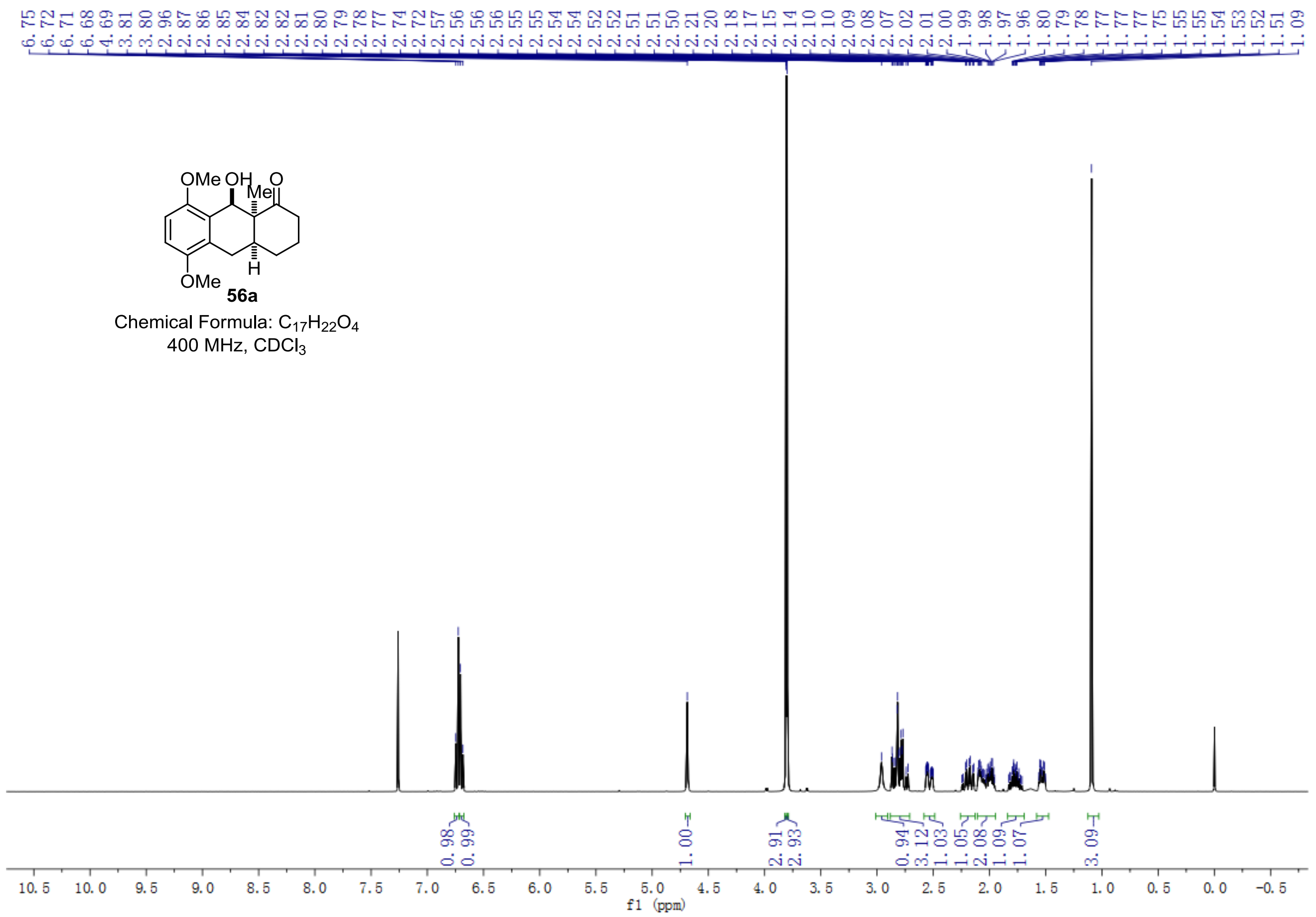

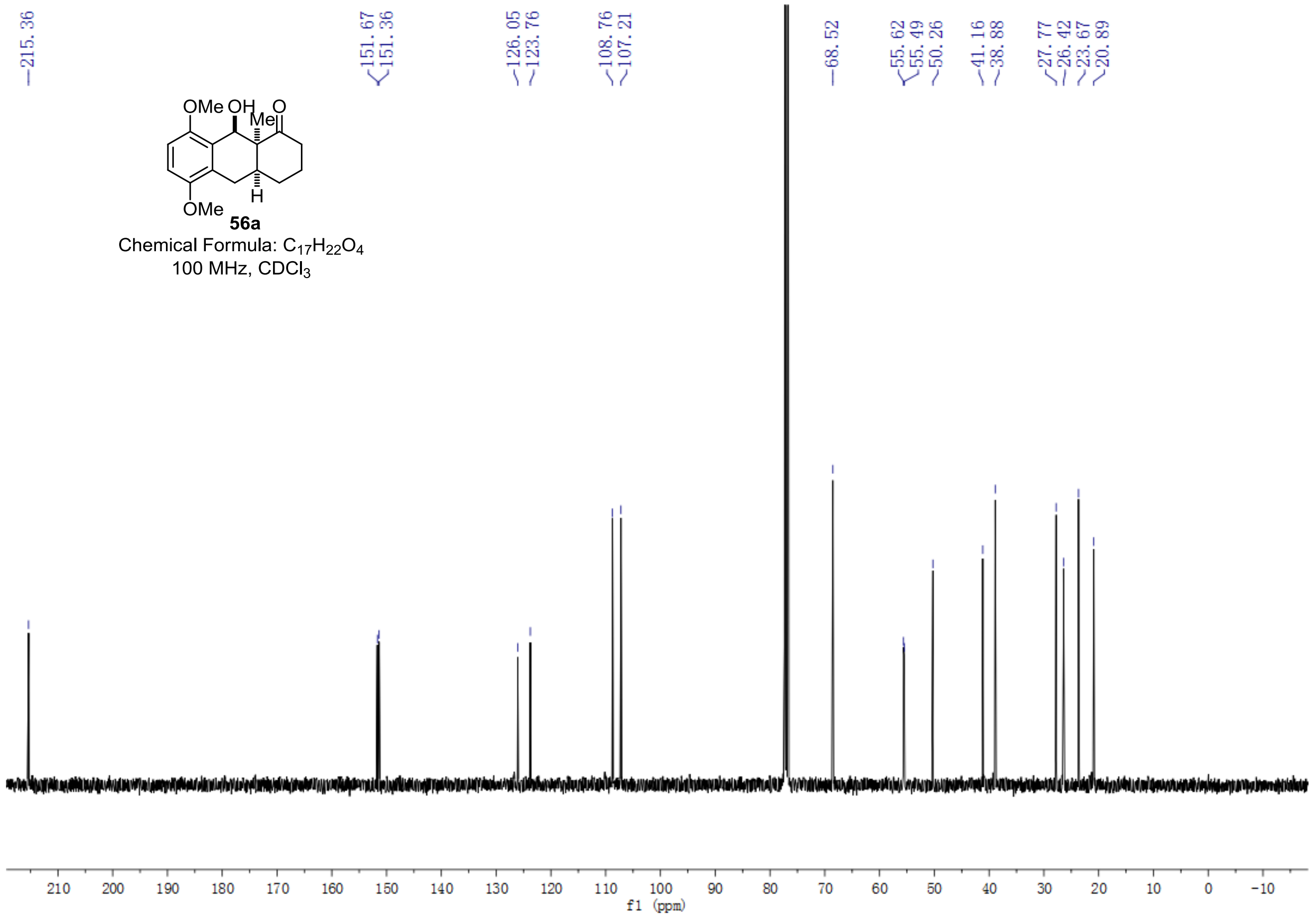

Supplementary Figure 37. ^{1}**H and** ^{13}**C NMR spectra for 56a.**

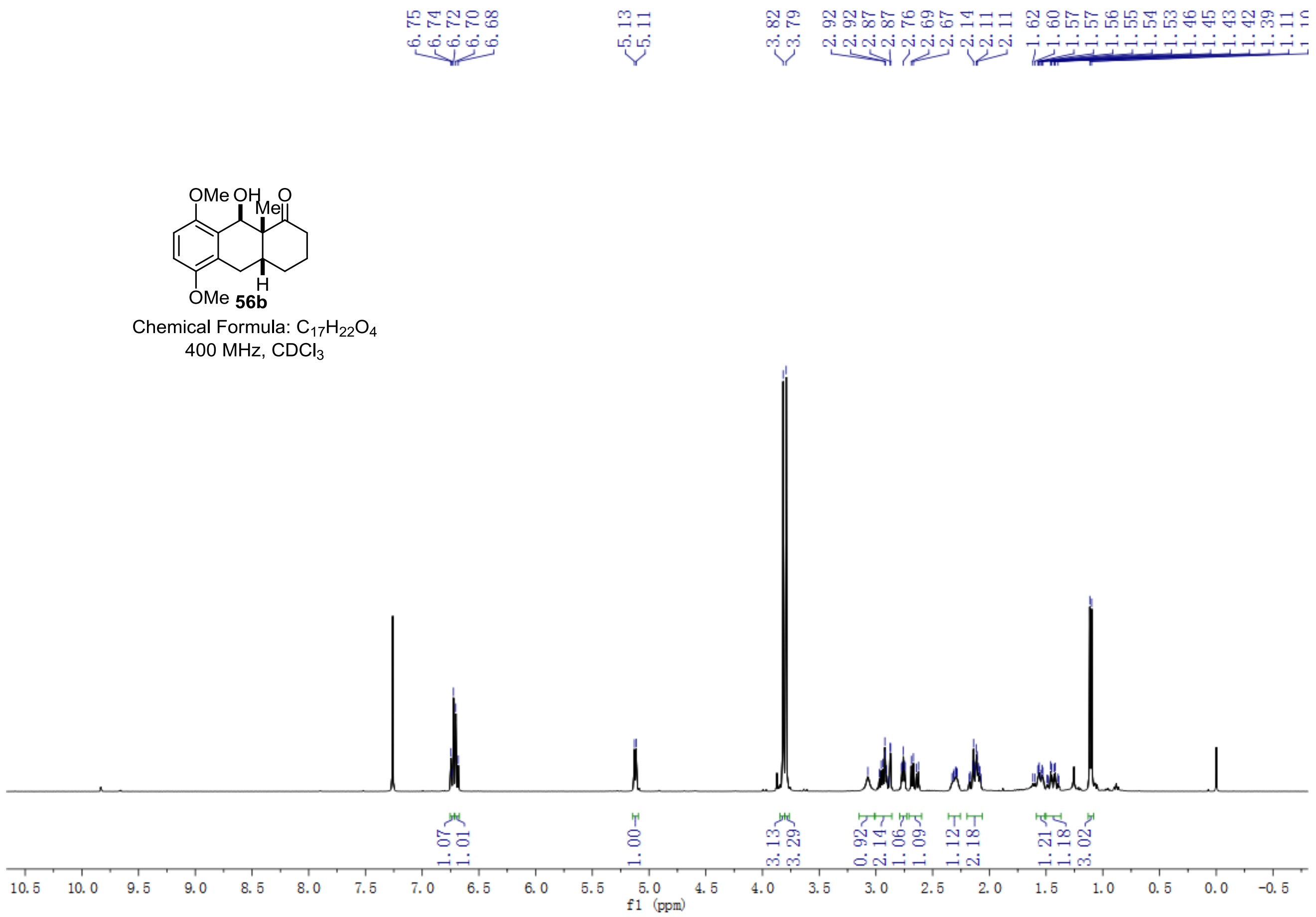

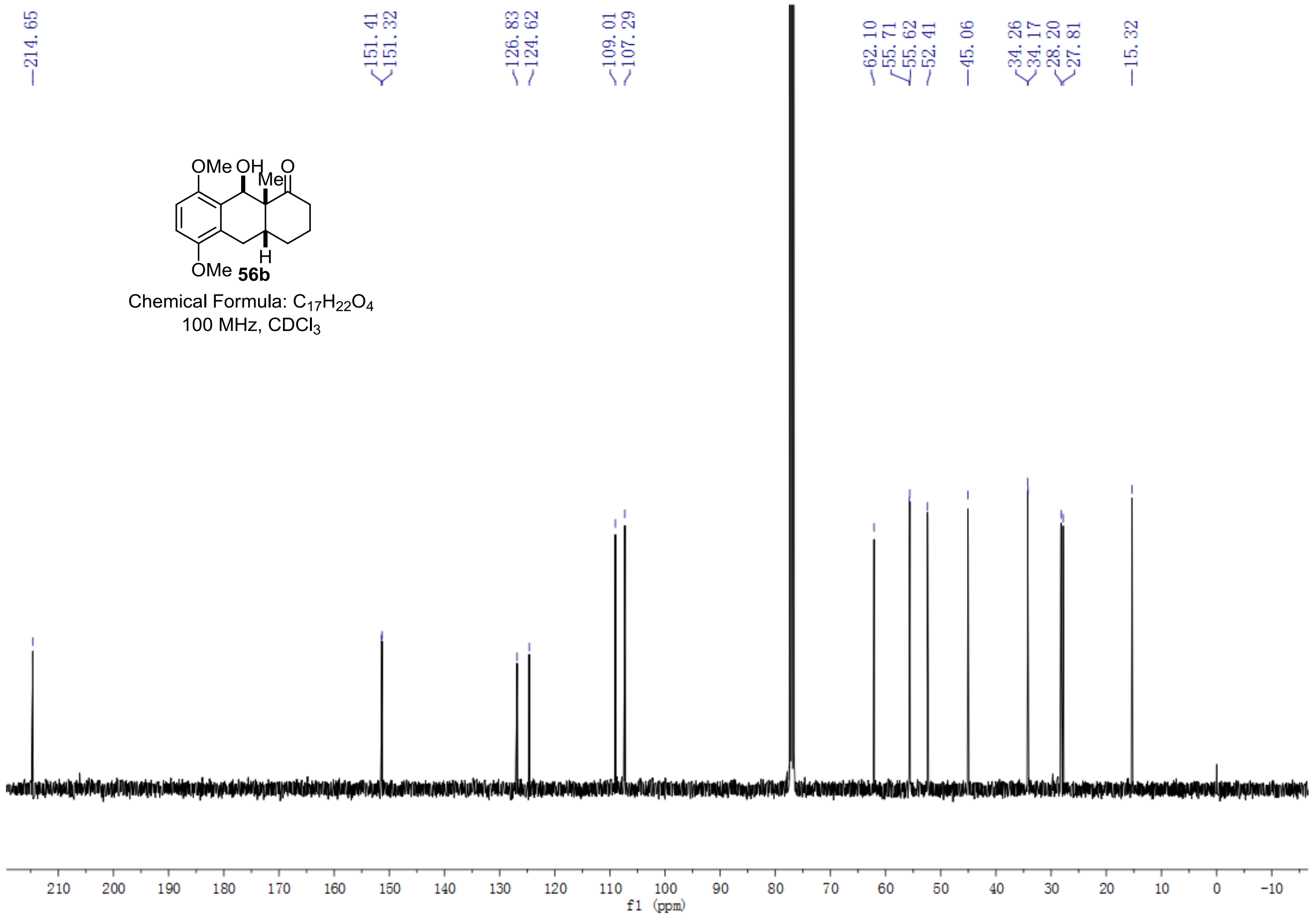

Supplementary Figure 38. ^{1}H **and** ^{13}C **NMR spectra for 56b.**

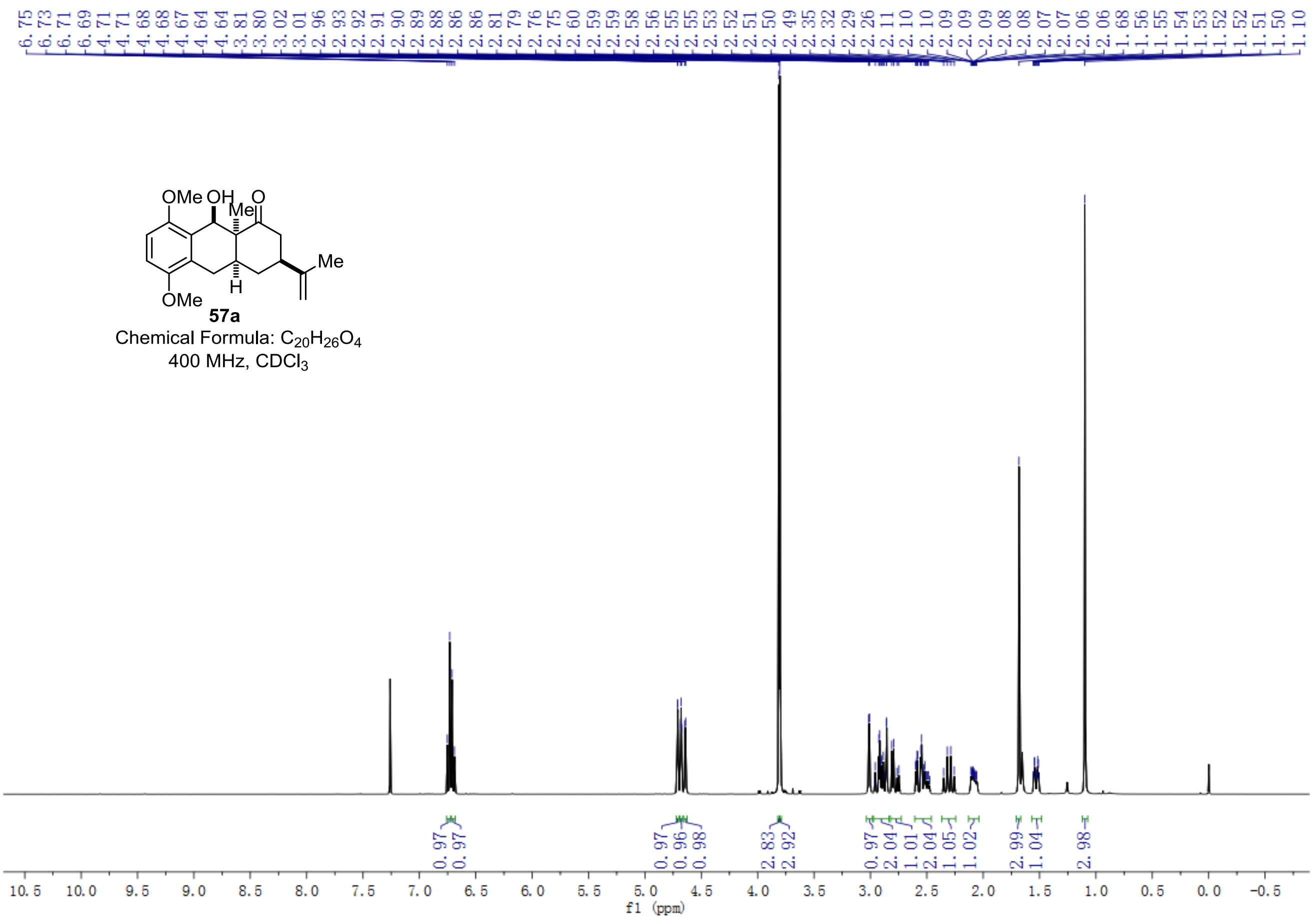

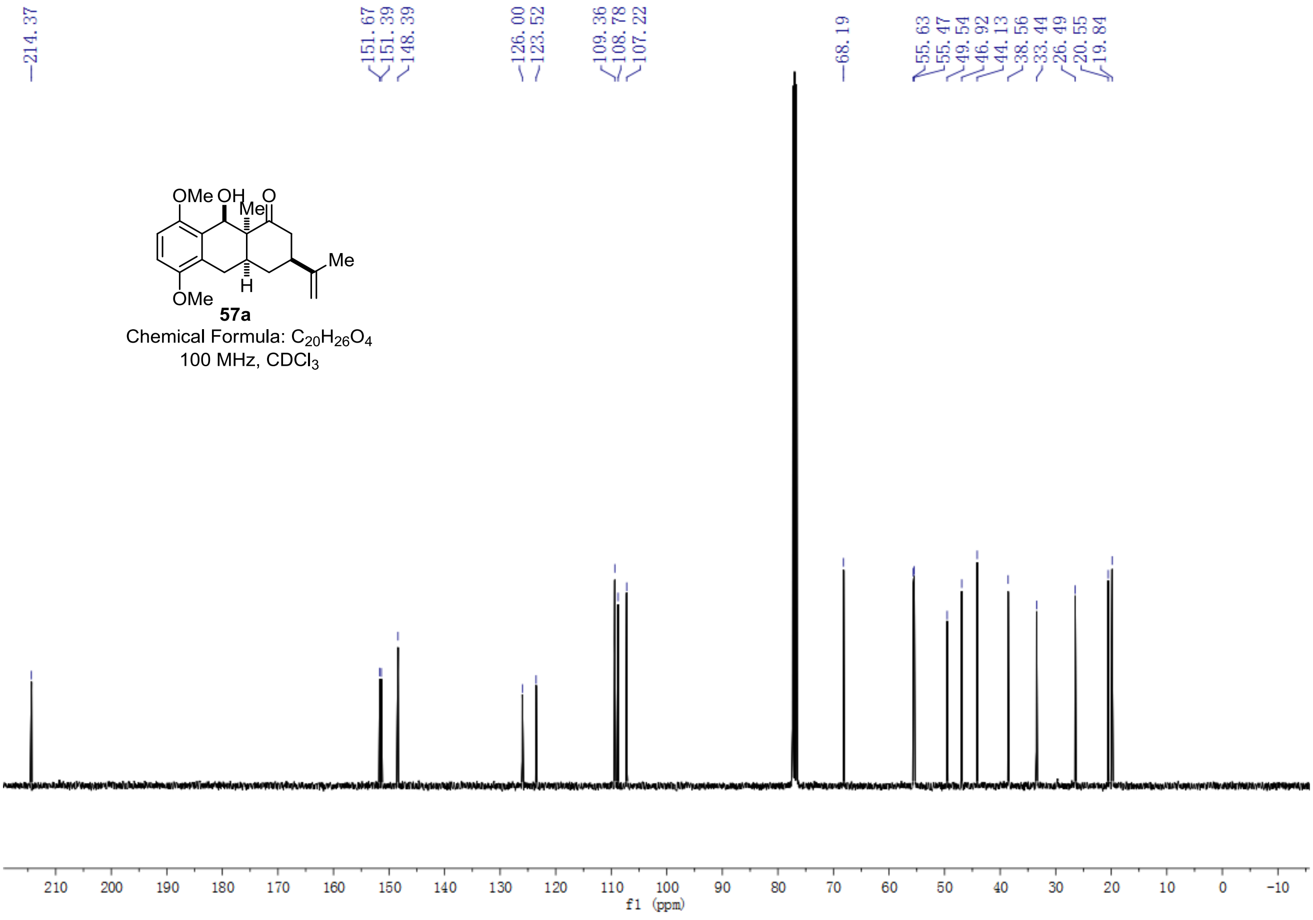

Supplementary Figure 39.^{1}H and ^{13}C NMR spectra for 57a.

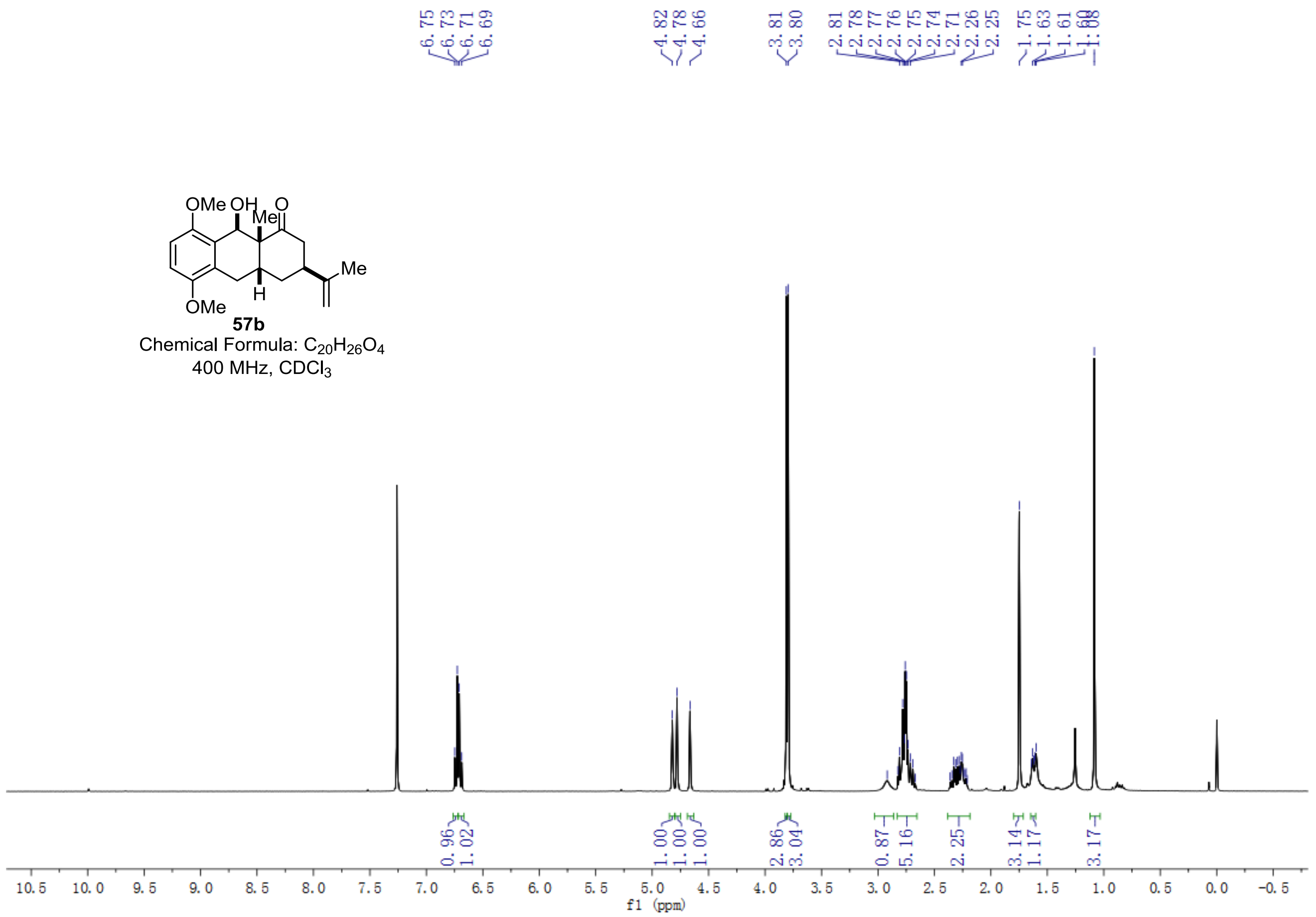

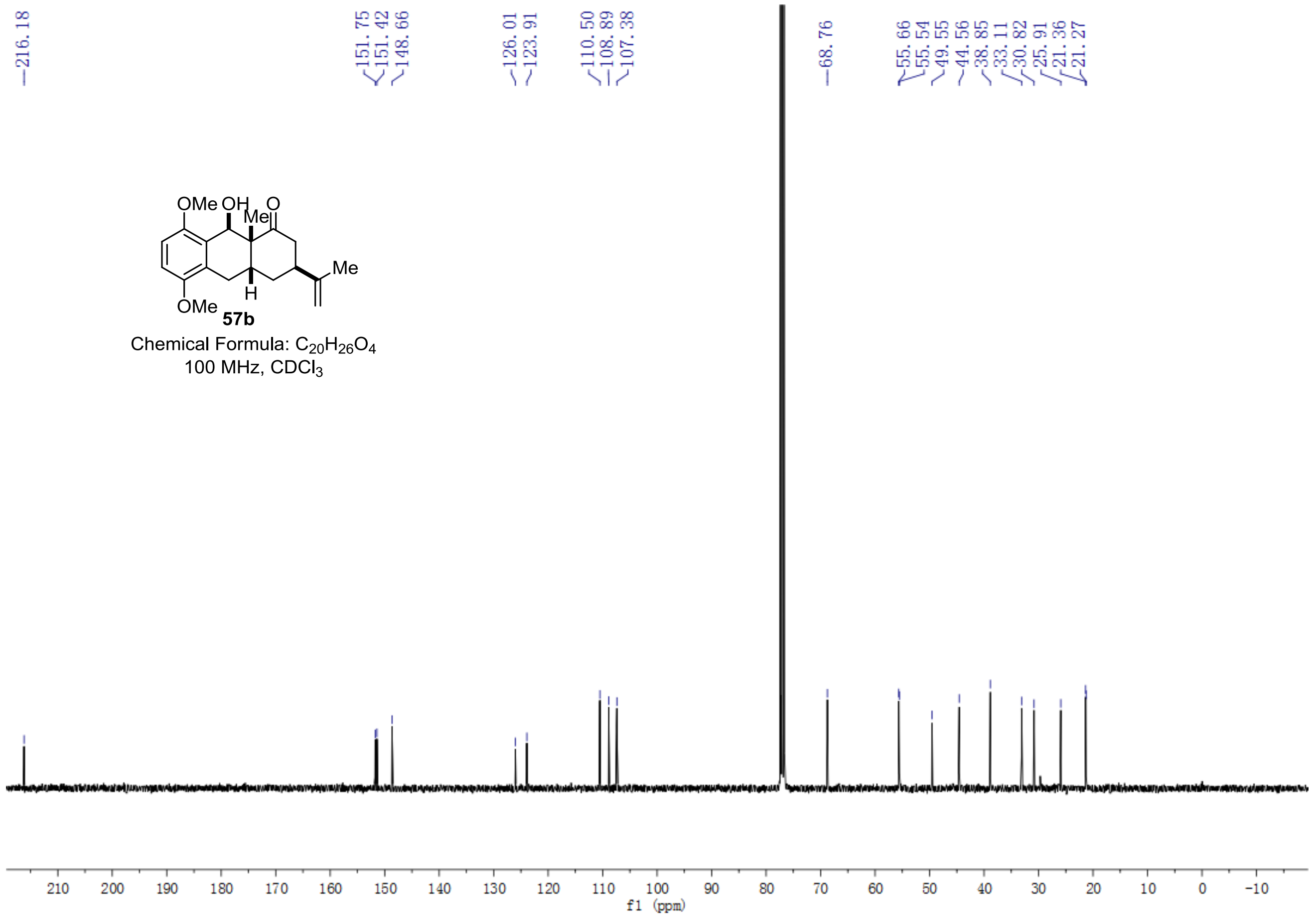

Supplementary Figure 40. ^{1}H and ^{13}C NMR spectra for 57b.

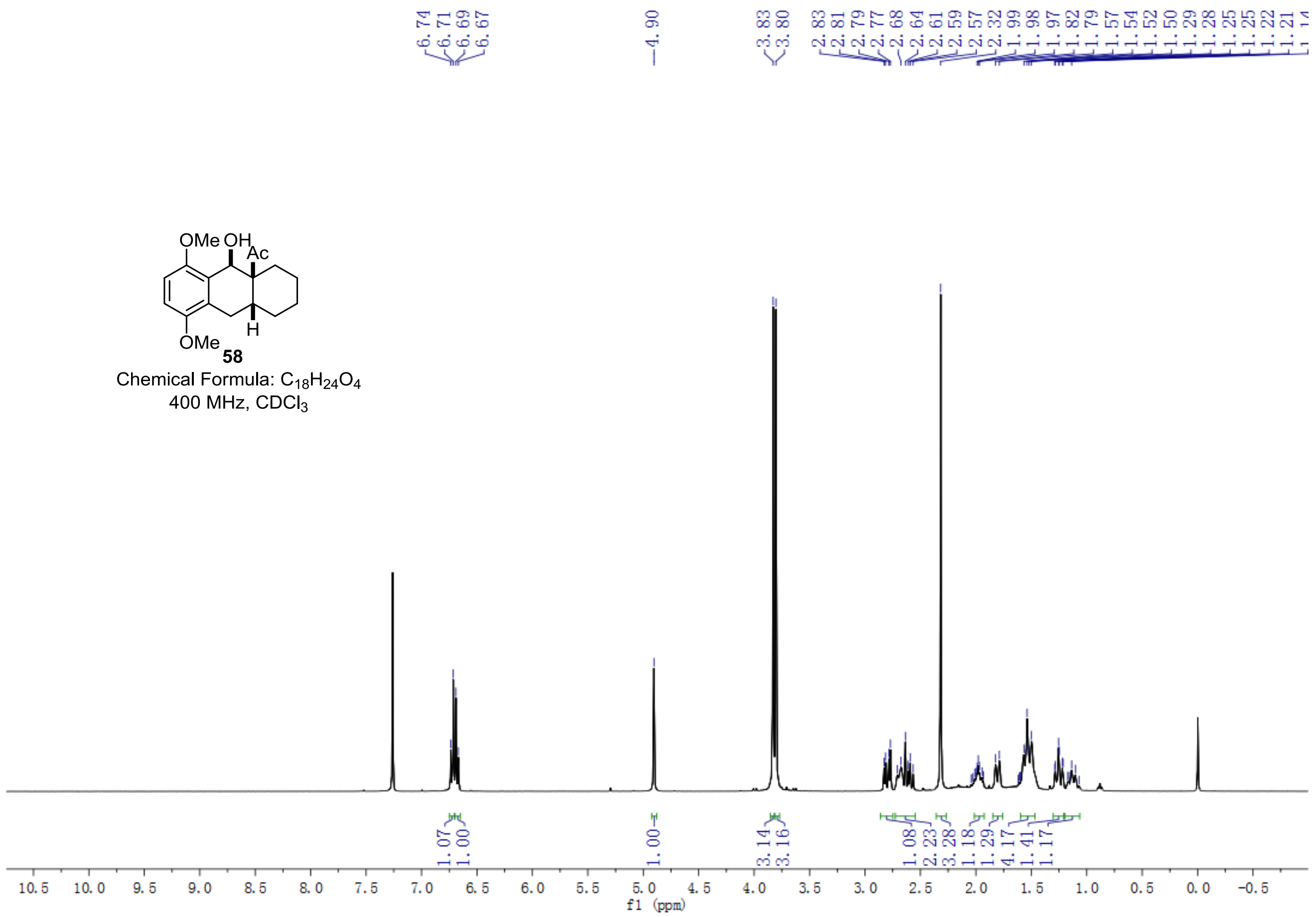

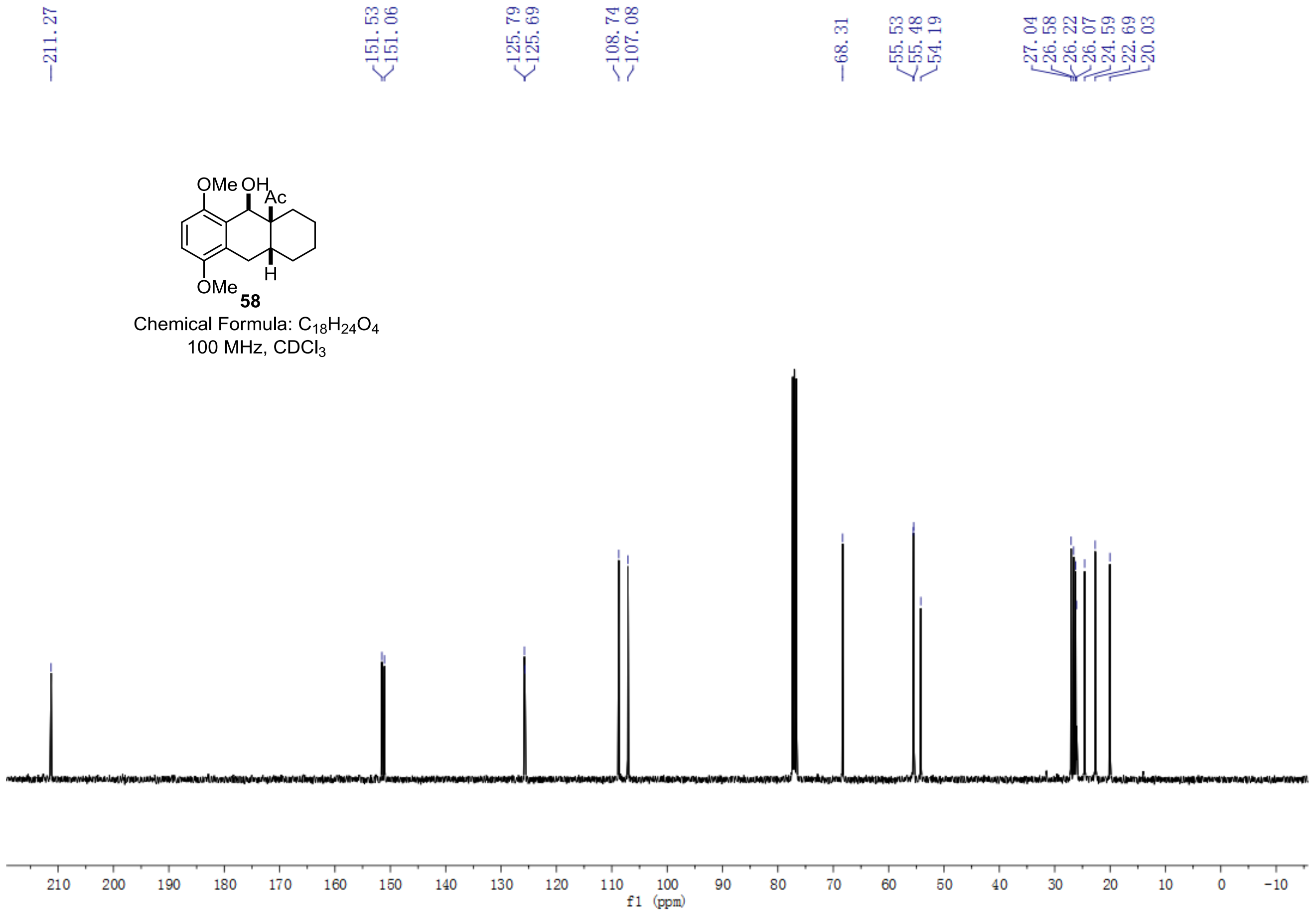

Supplementary Figure 41.^{1}H and ^{13}C NMR spectra for 58.

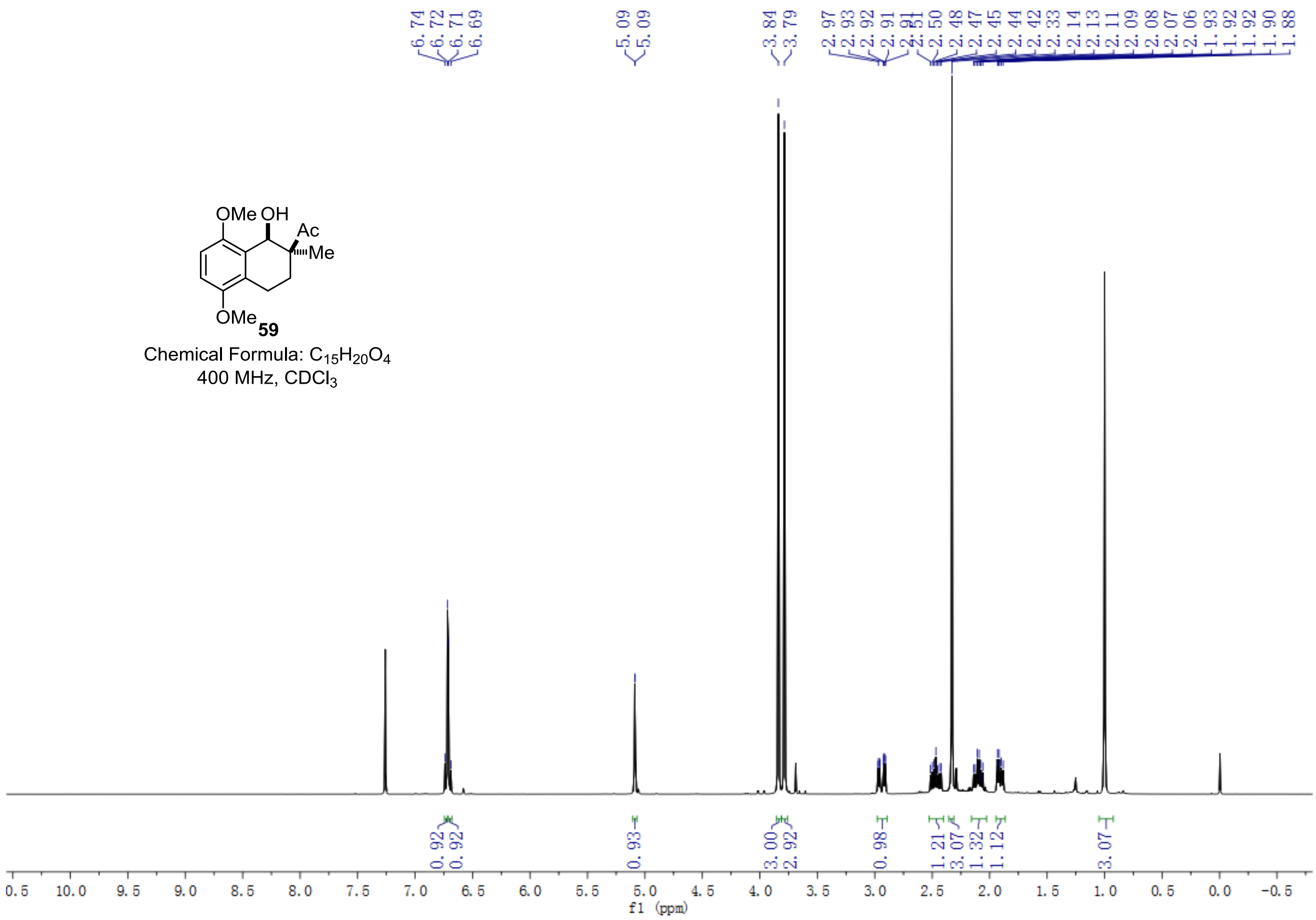

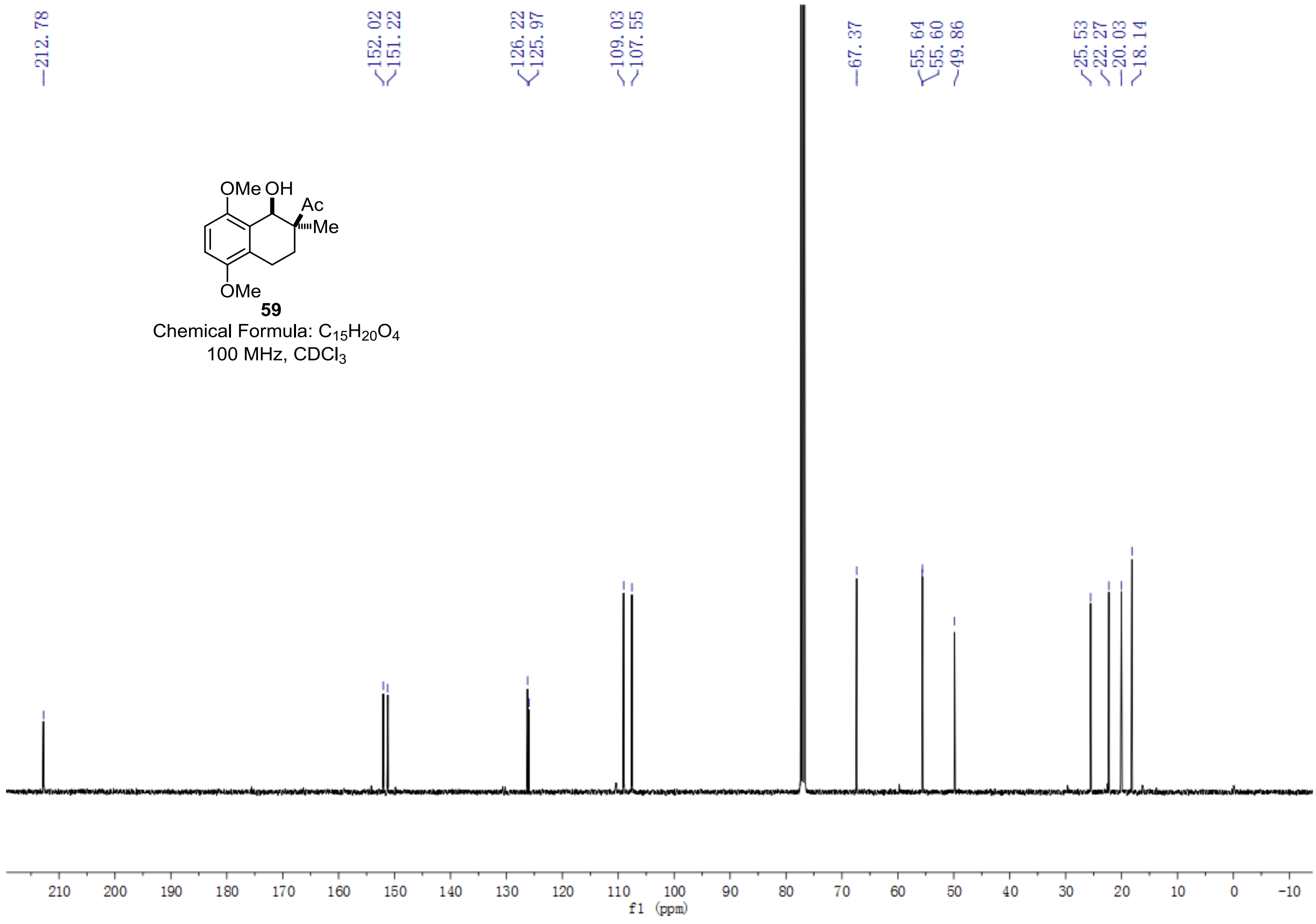

Supplementary Figure 42.^{1}H and ^{13}C NMR spectra for 59.

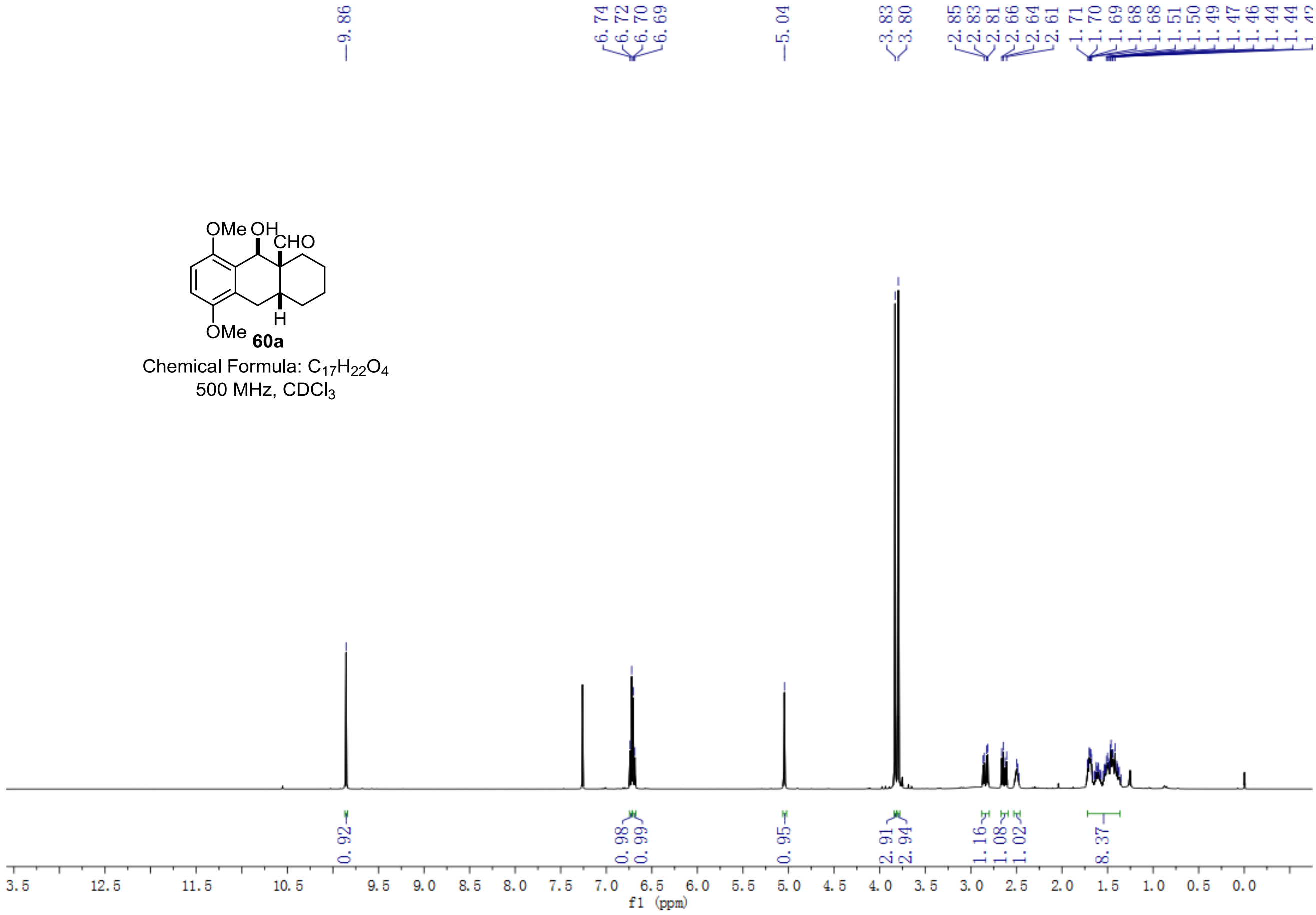

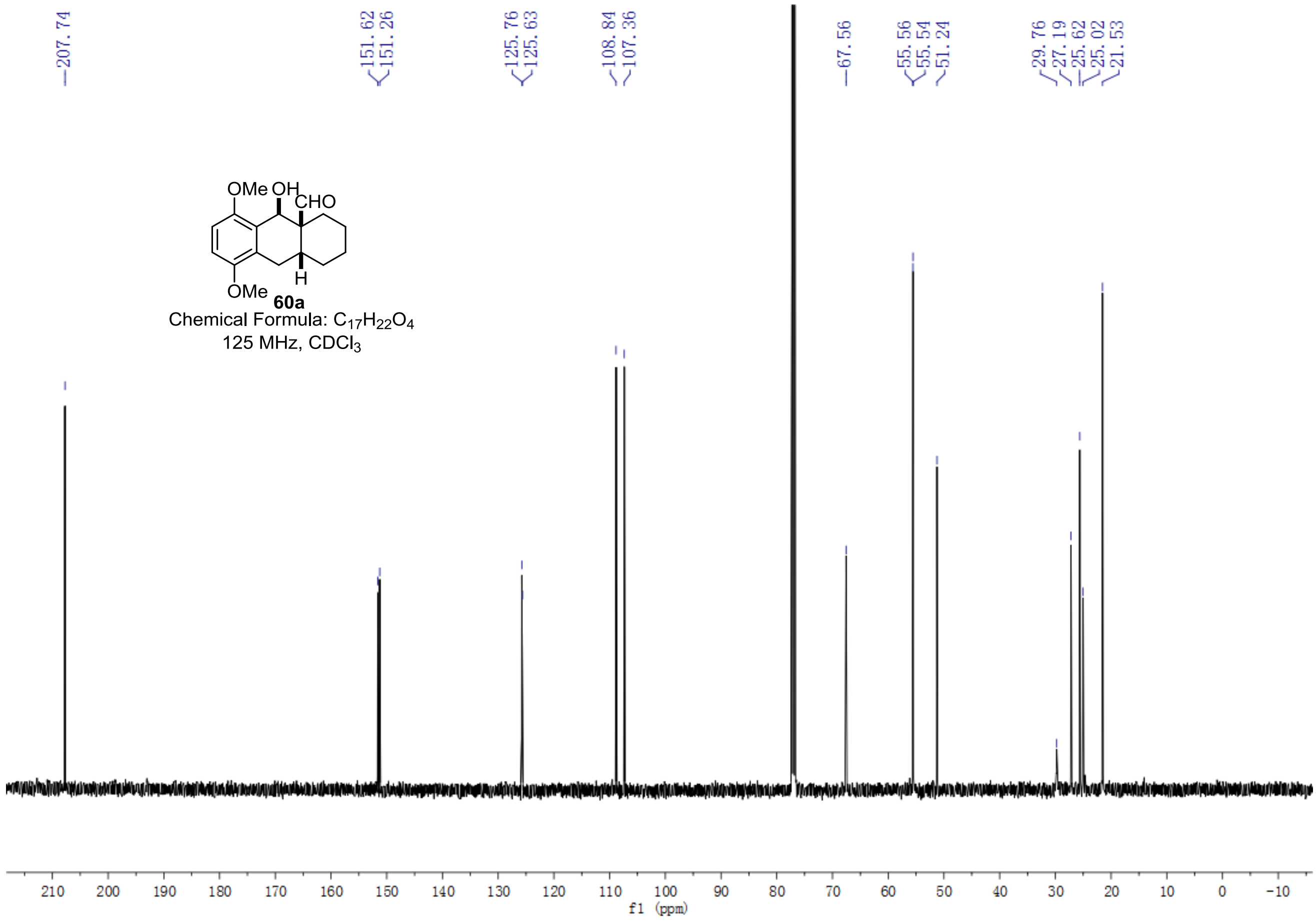

Supplementary Figure 43. ^{1}H **and** ^{13}C **NMR spectra for 60a.**

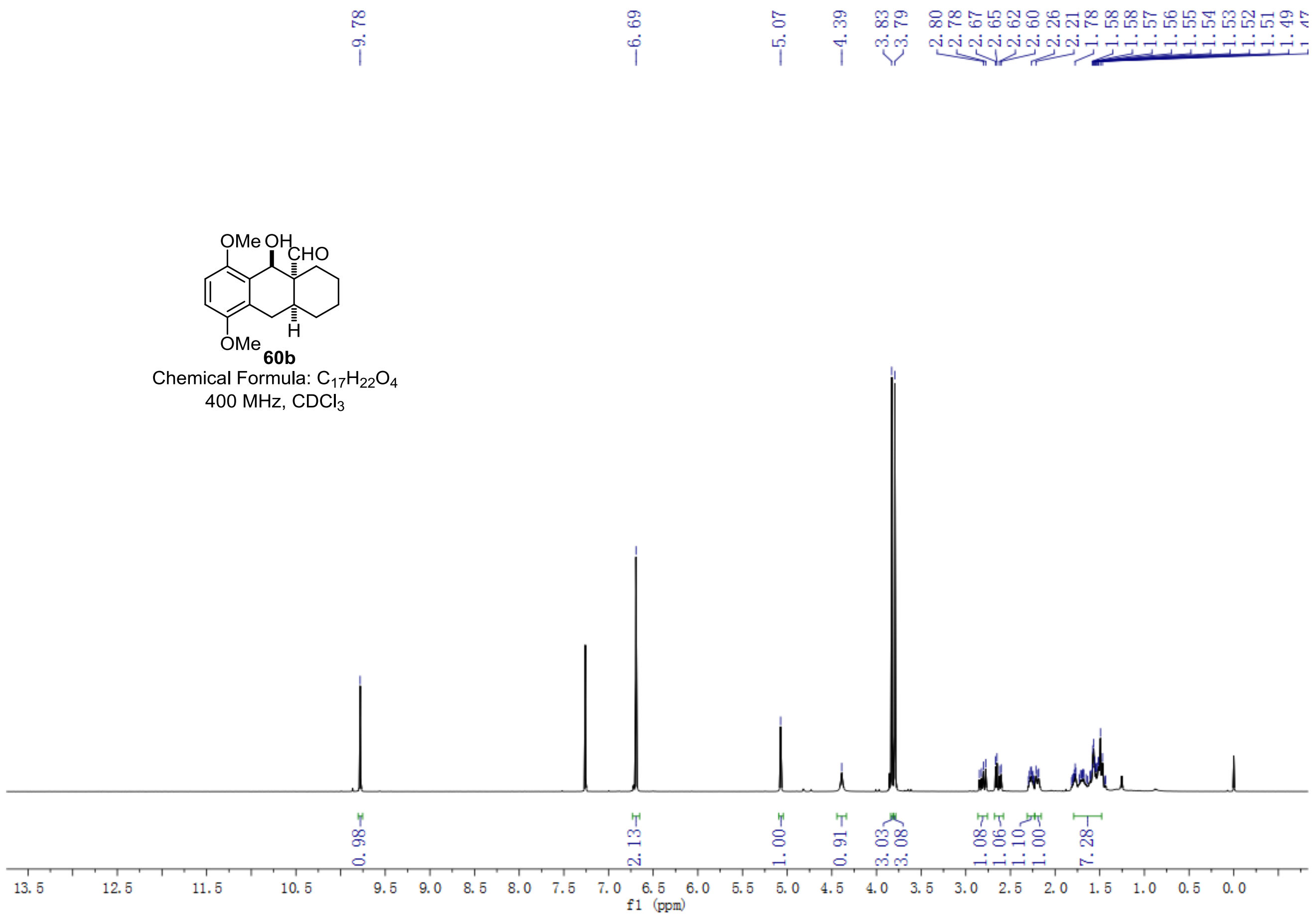

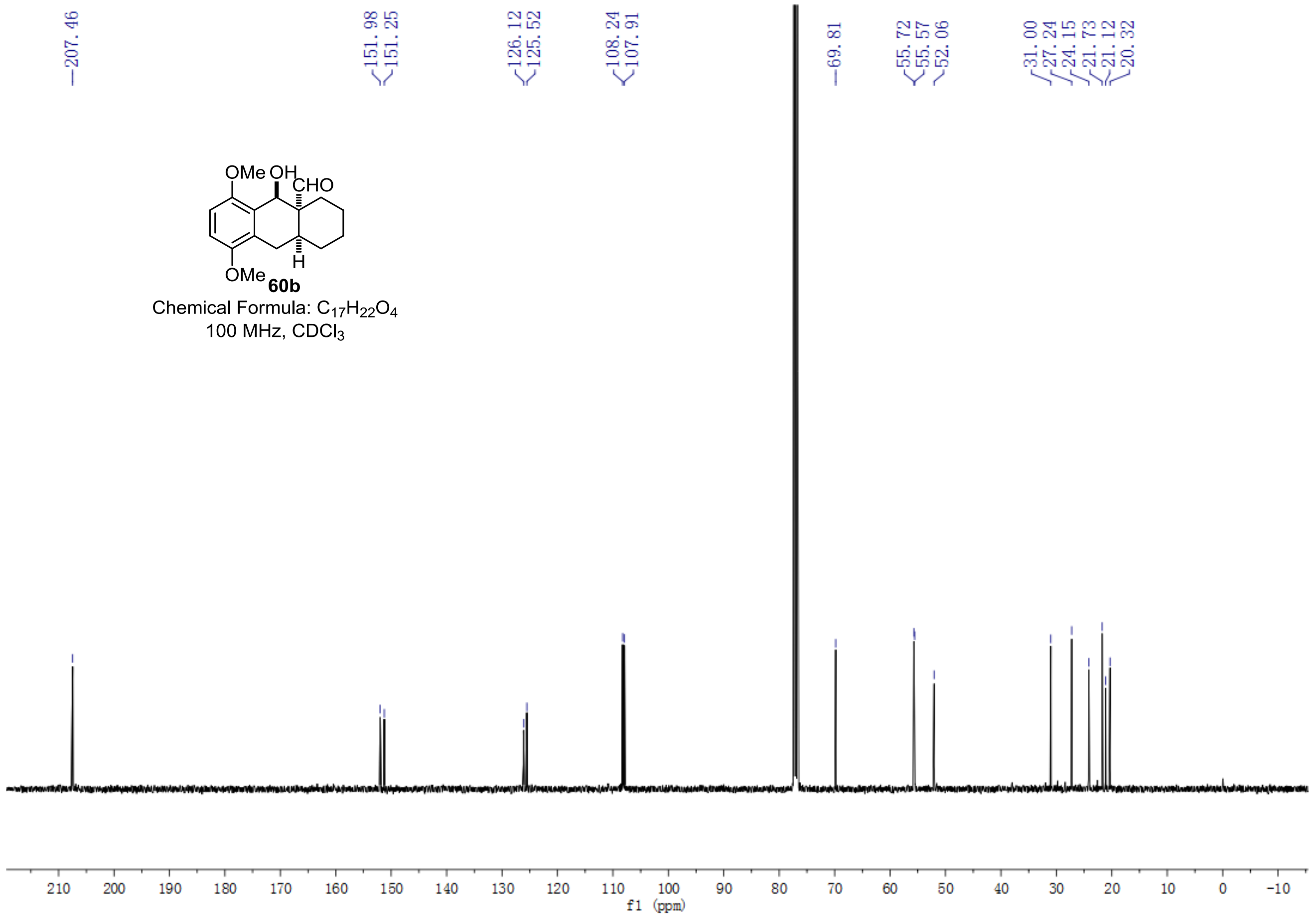

Supplementary Figure 44. ^{1}H and ^{13}C NMR spectra for 60b.

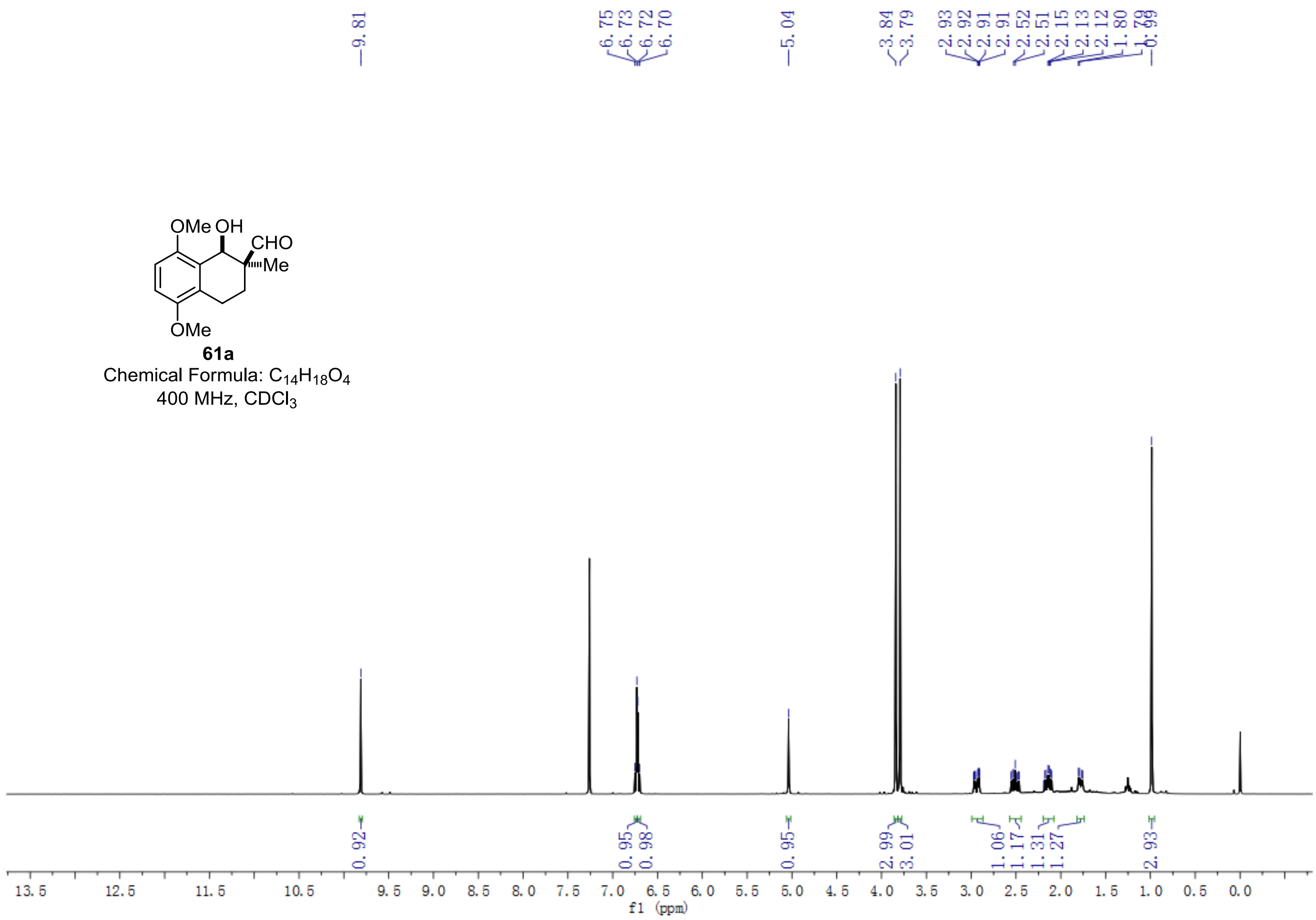

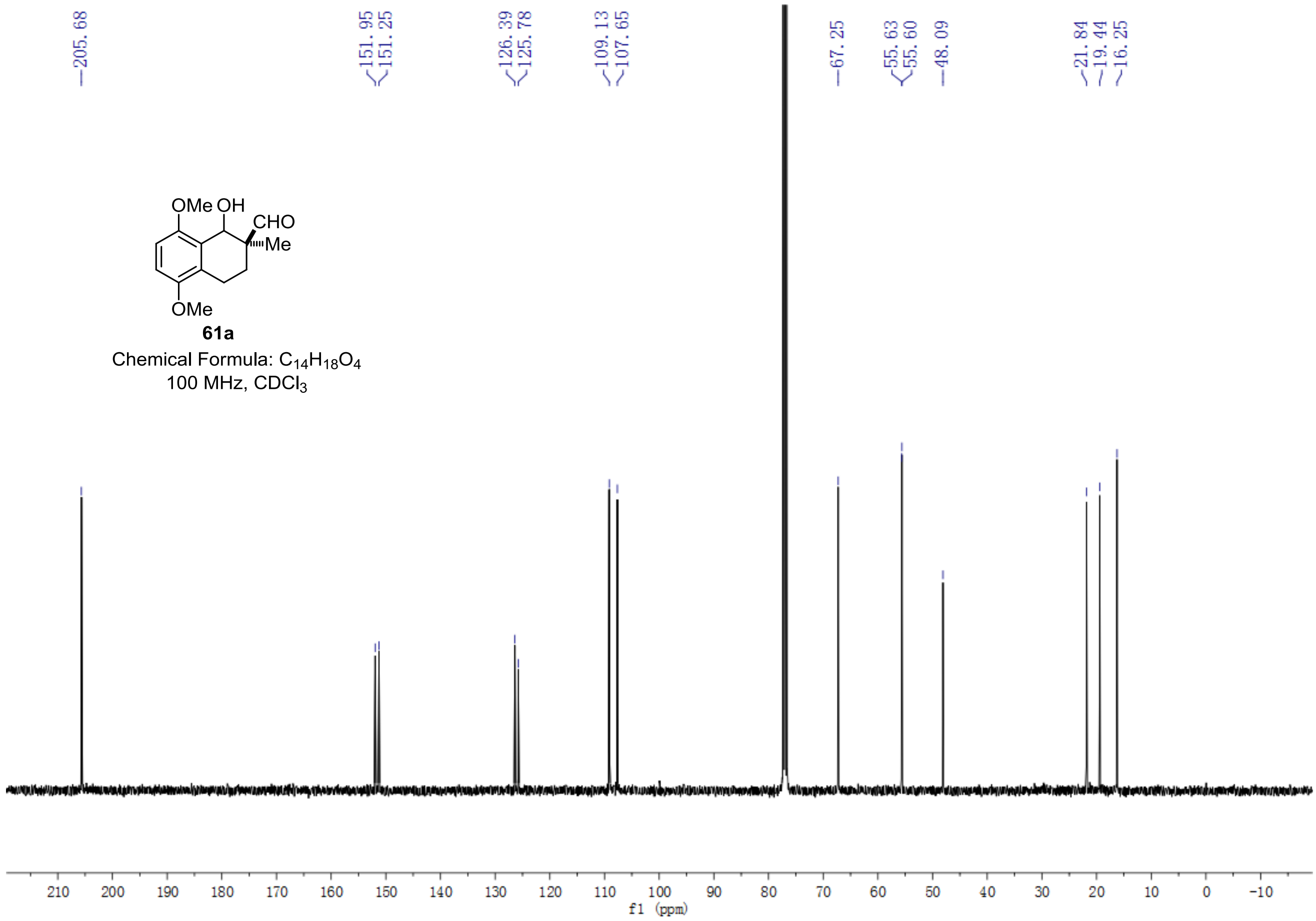

Supplementary Figure 45. ^{1}**H and** ^{13}**C NMR spectra for 61a.**

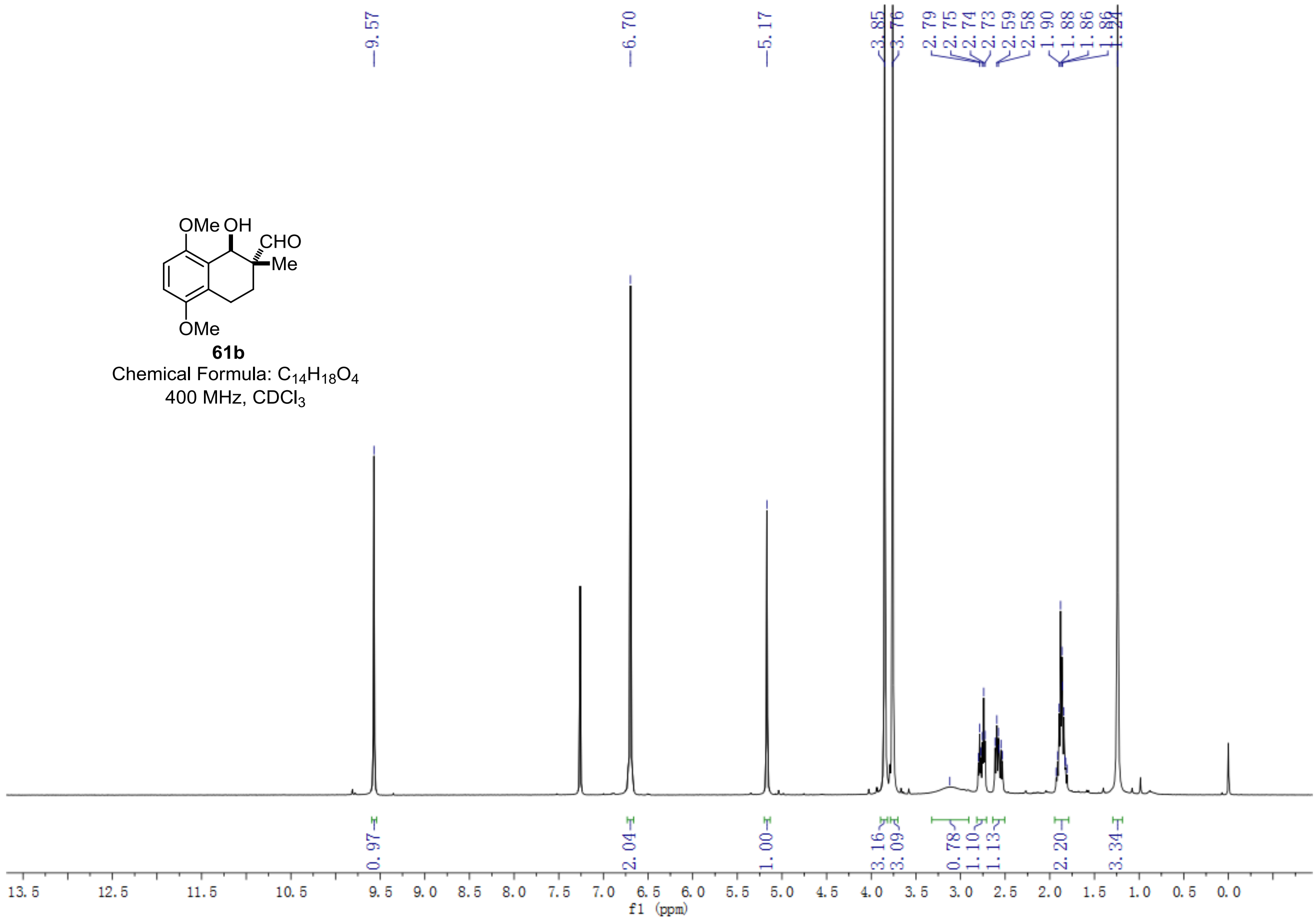

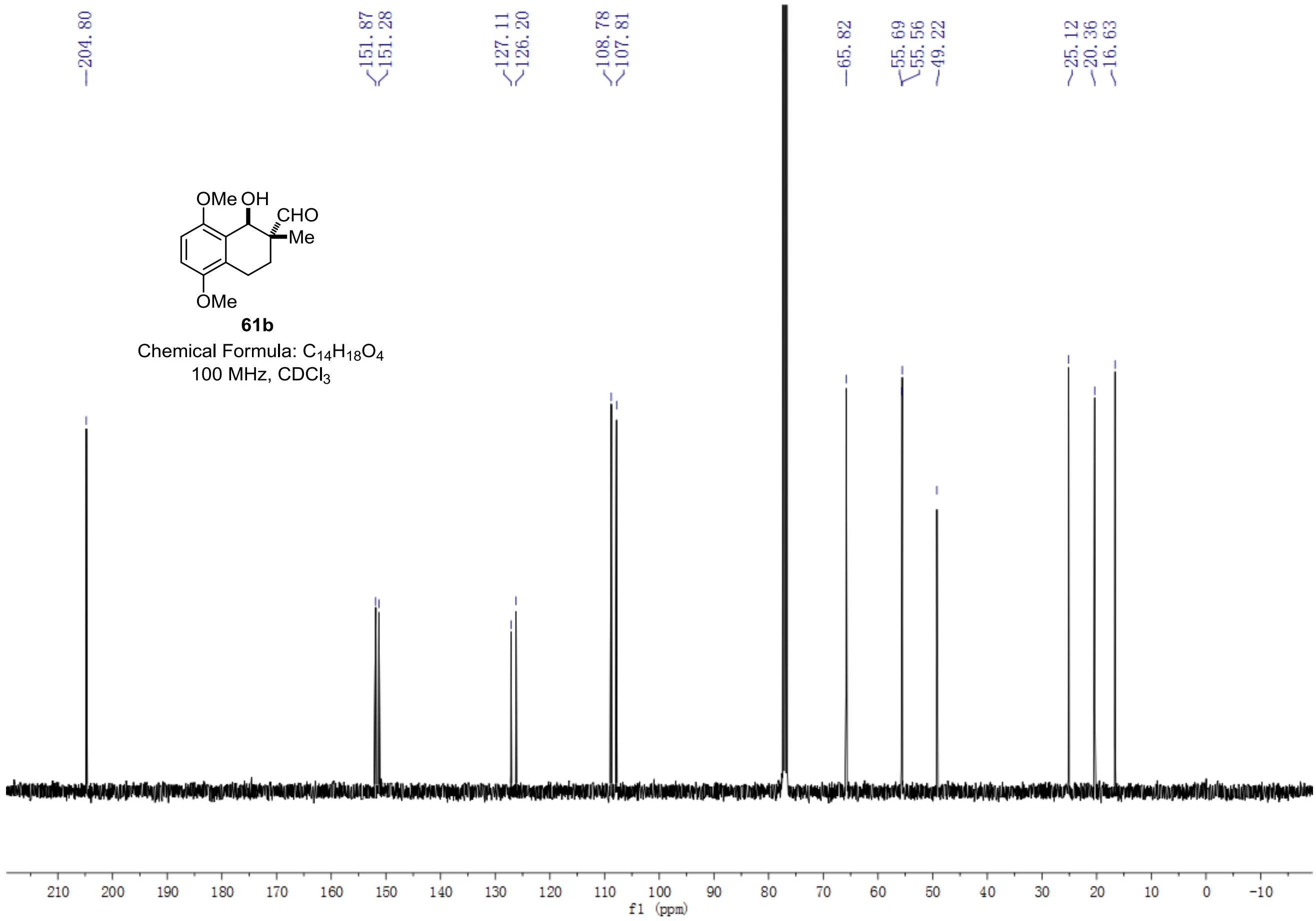

Supplementary Figure 46. ^{1}H and ^{13}C NMR spectra for 61b.

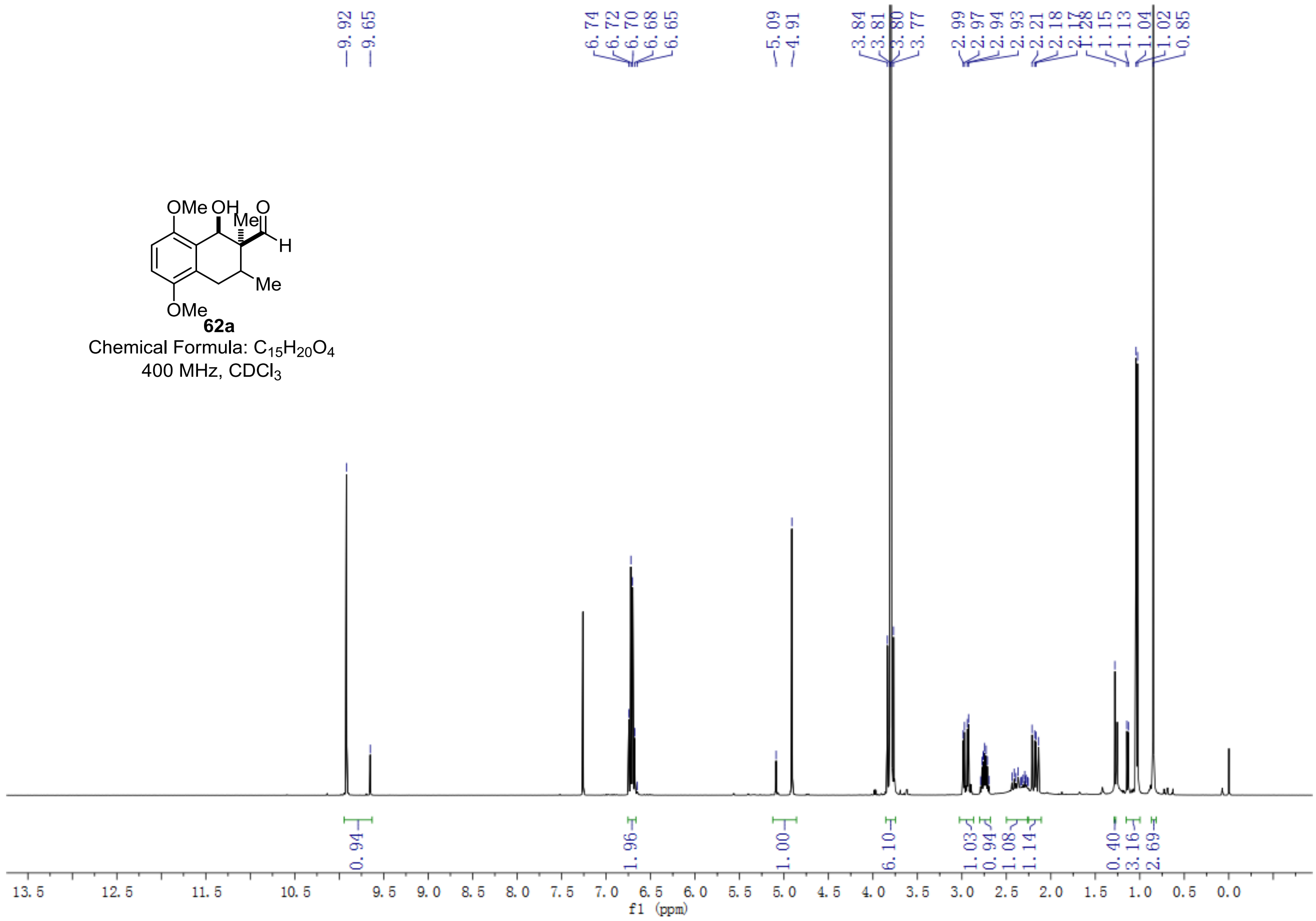

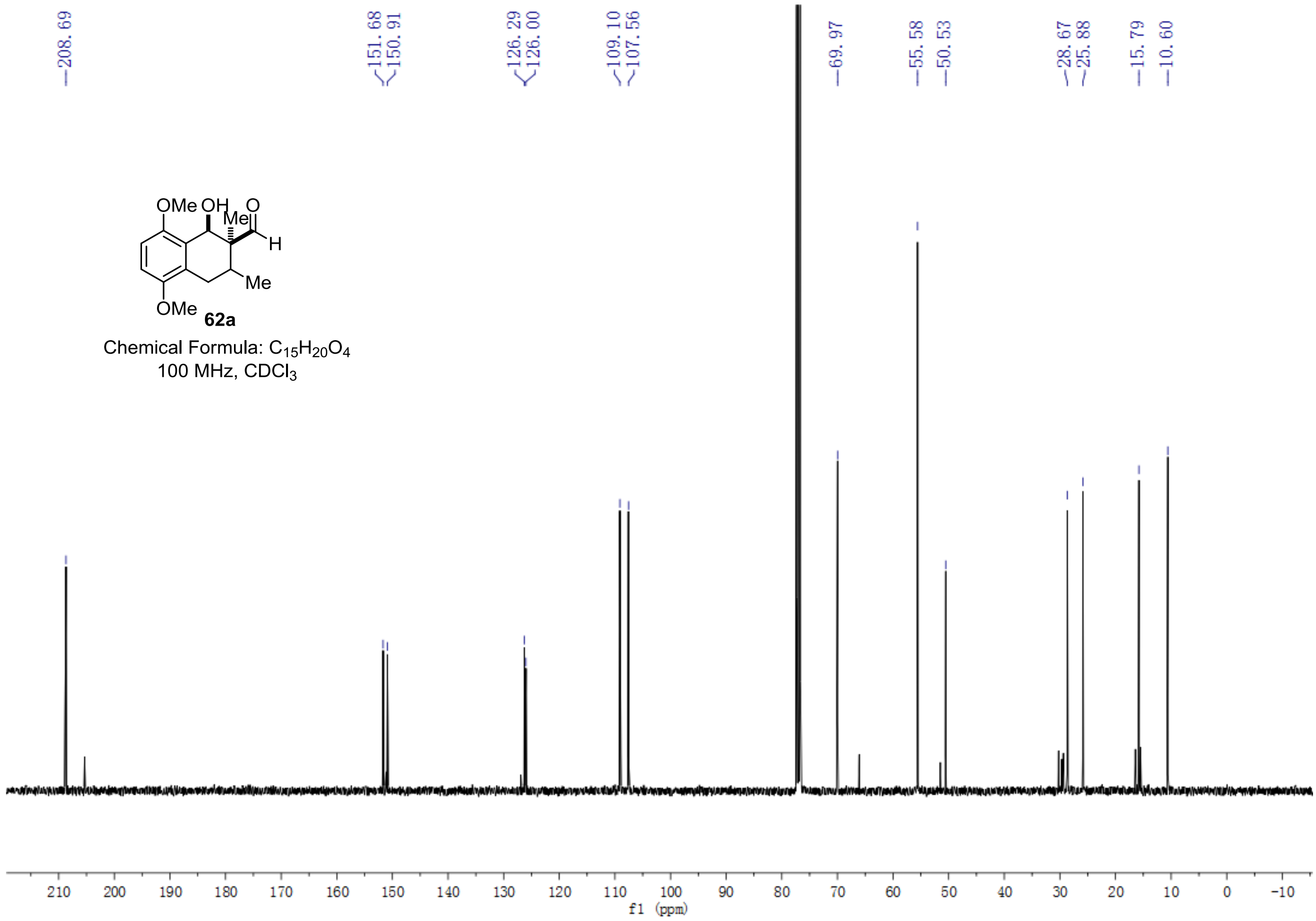

Supplementary Figure 47. ^{1}H and ^{13}C NMR spectra for 62a.

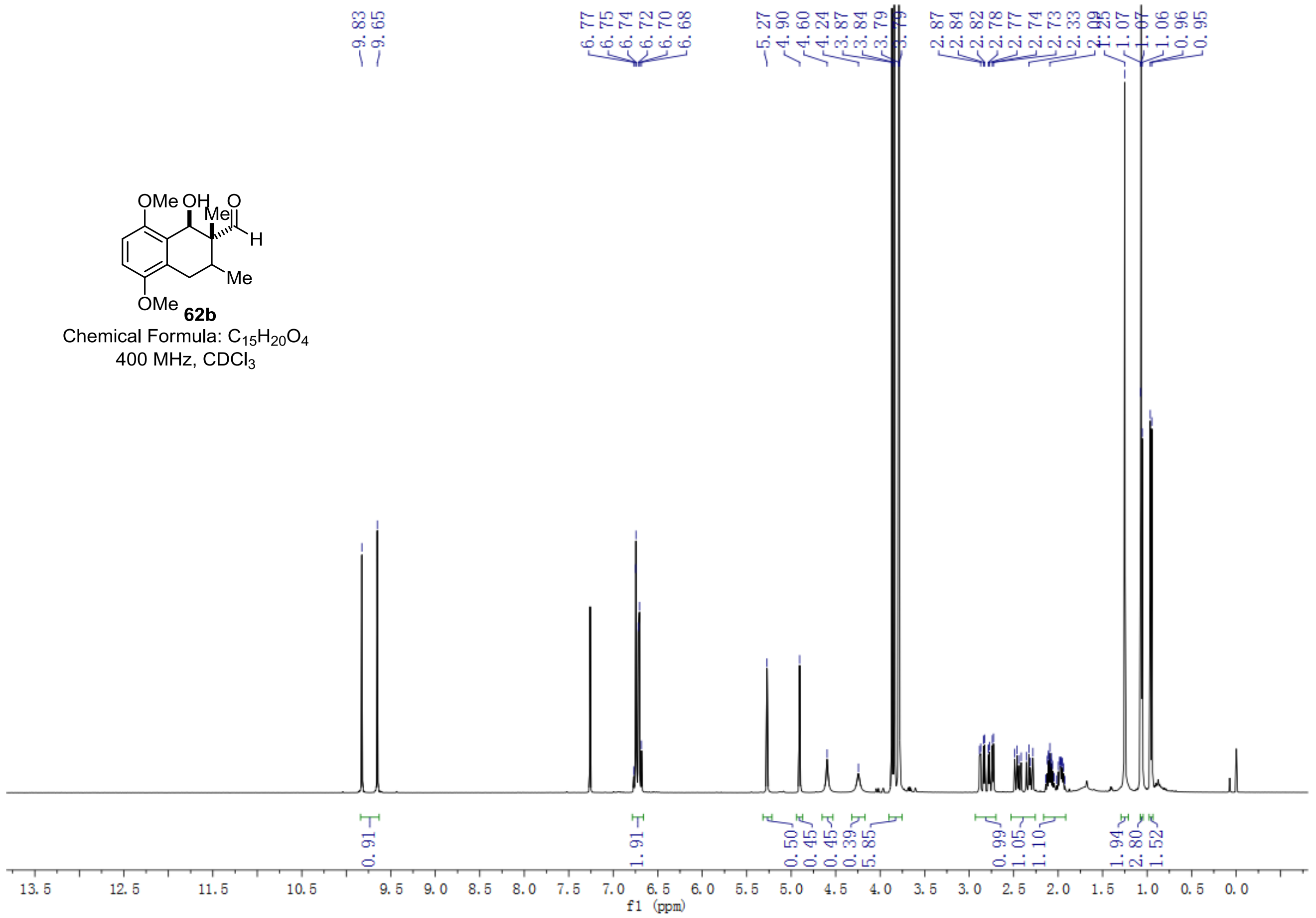

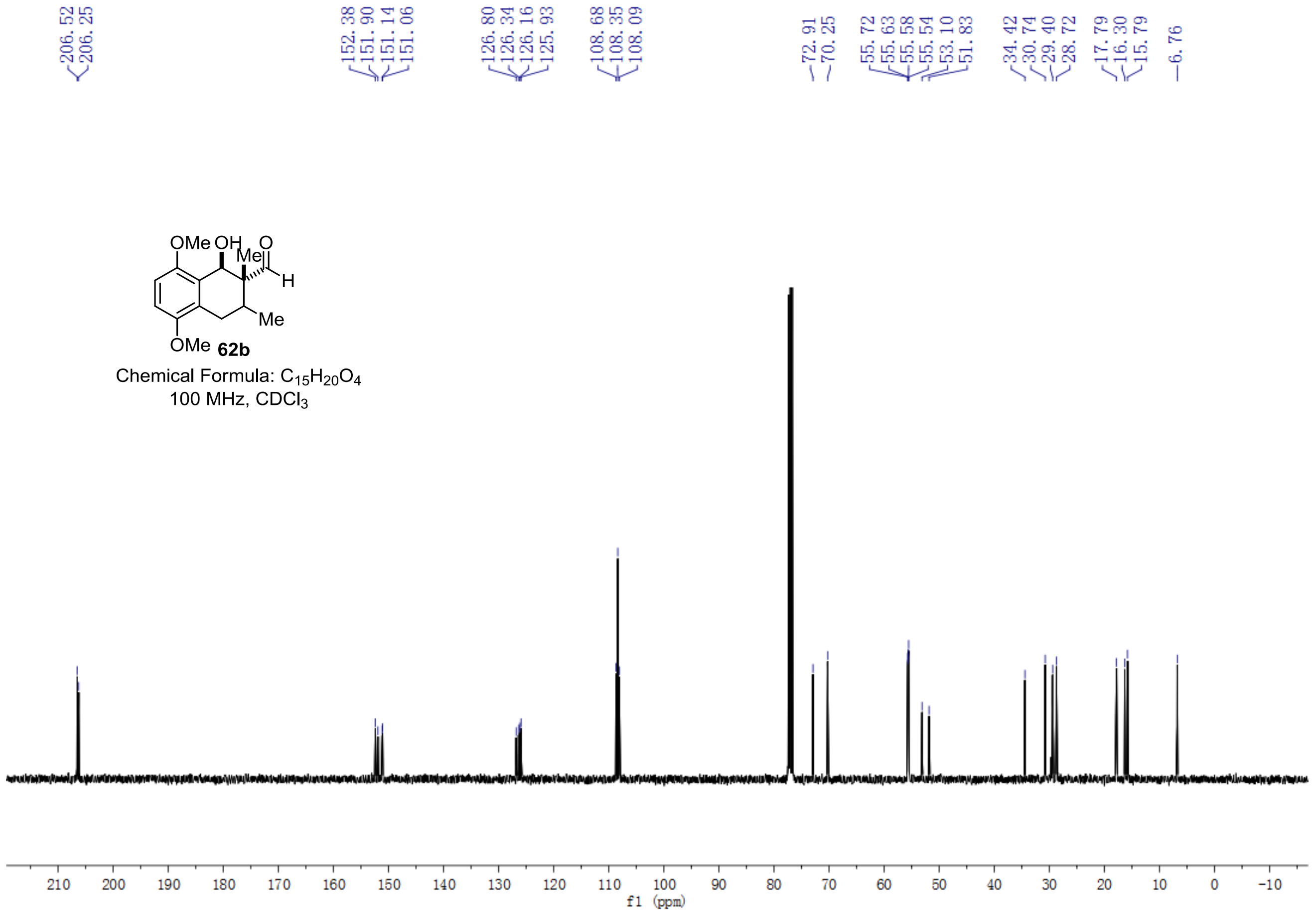

Supplementary Figure 48. ^1H **and** ^{13}C **NMR spectra for 62b.**

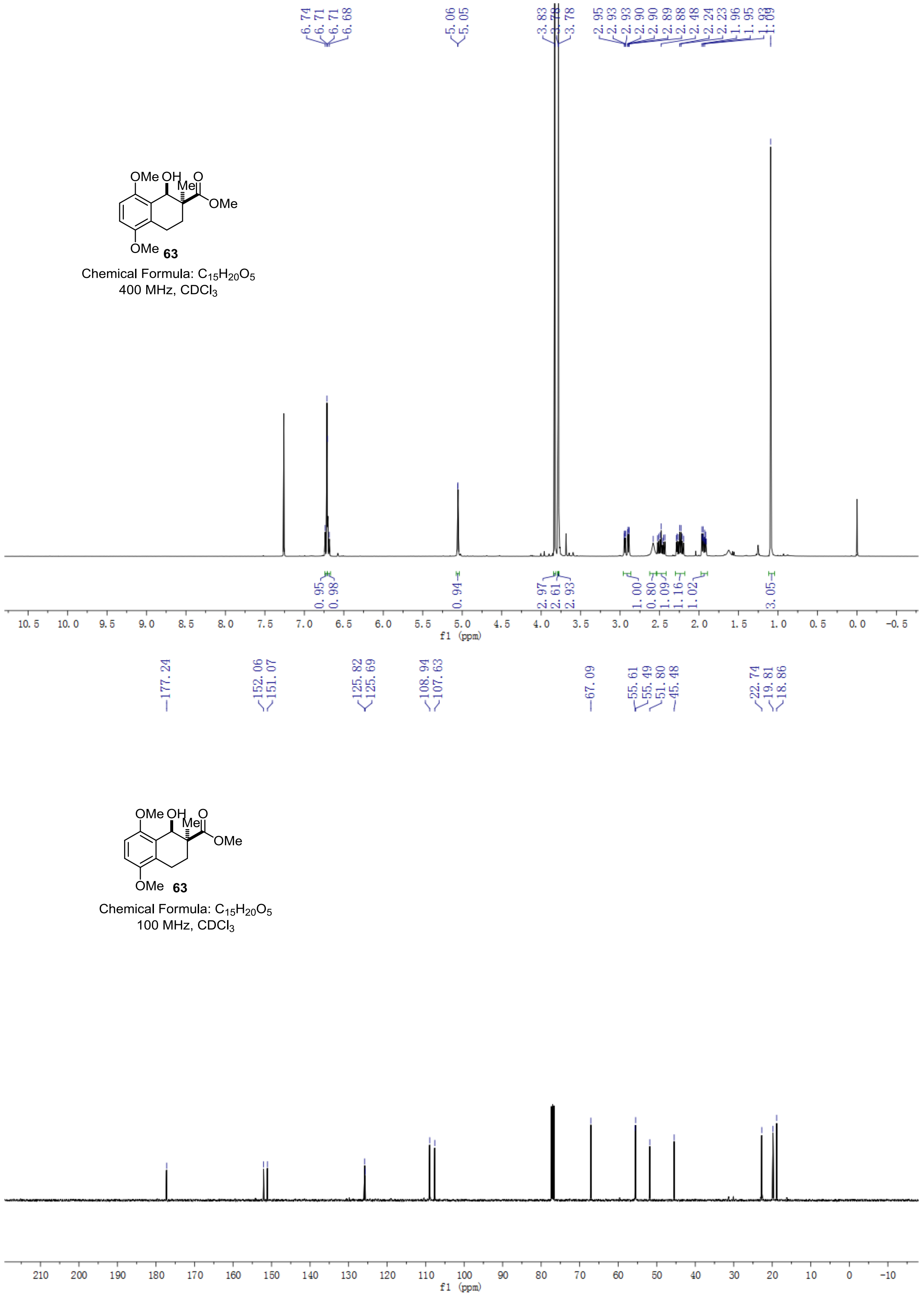

Supplementary Figure 49. ^{1}**H and** ^{13}**C NMR spectra for 63.**

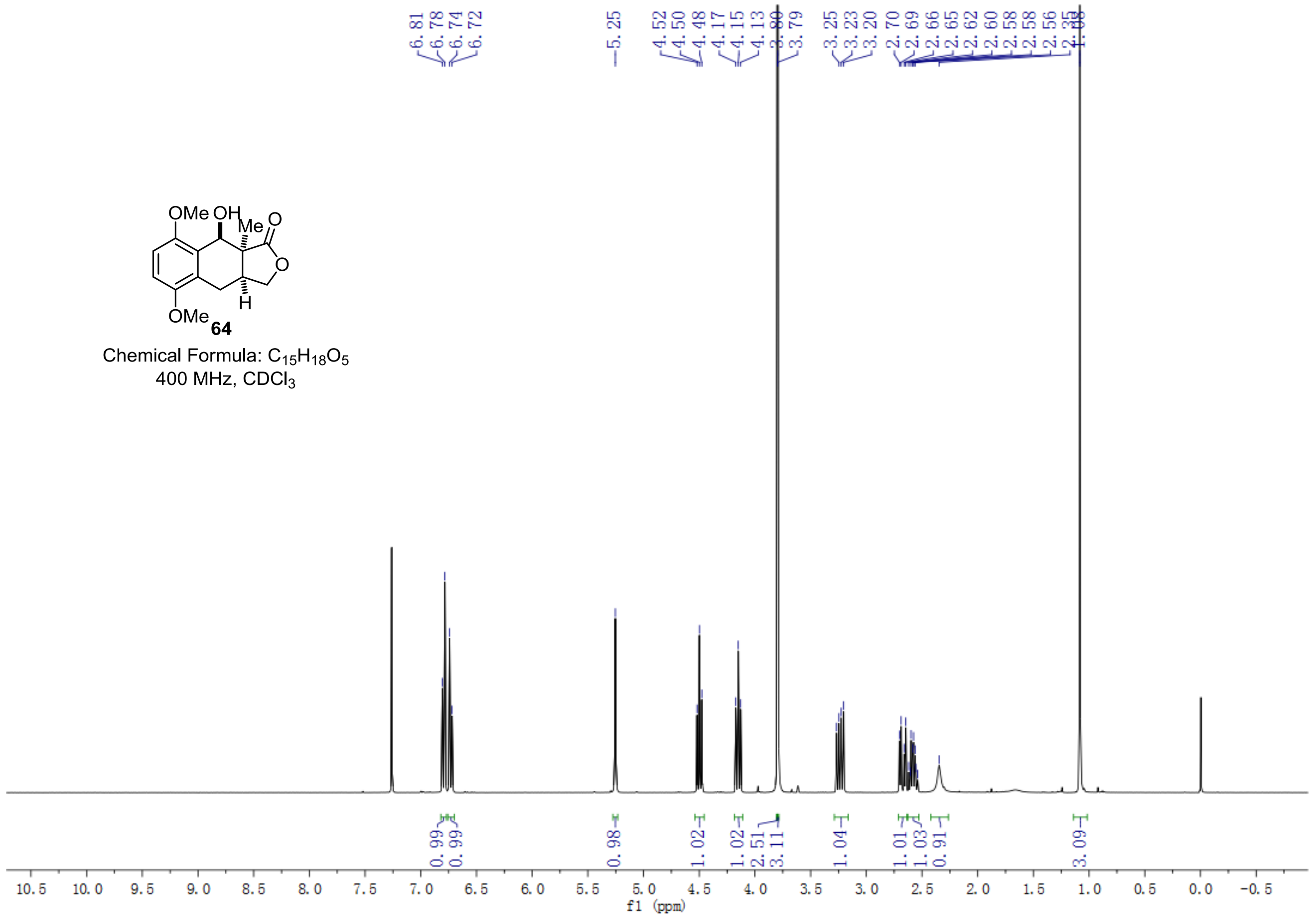

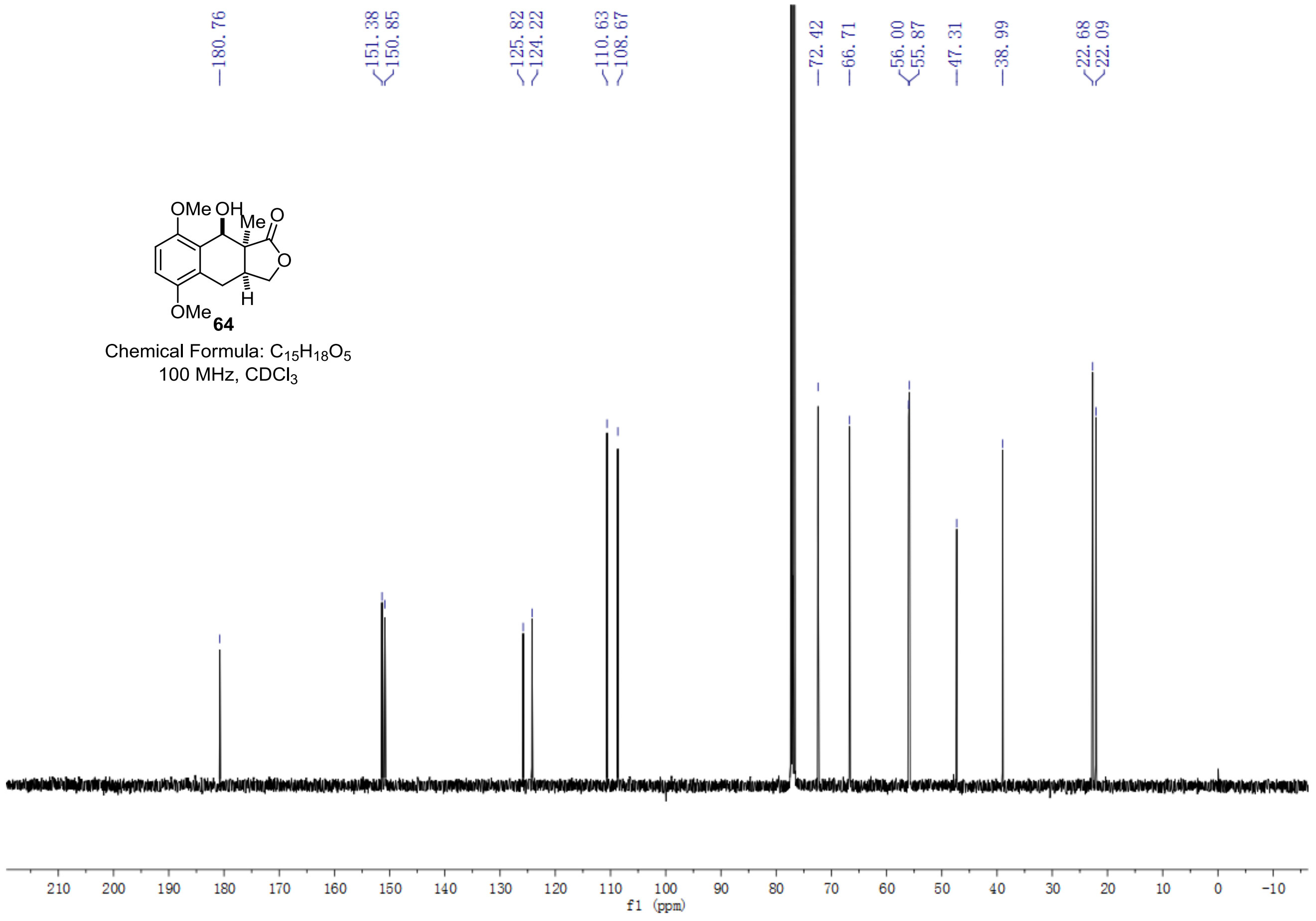

Supplementary Figure 50.^{1}H and ^{13}C NMR spectra for 64.

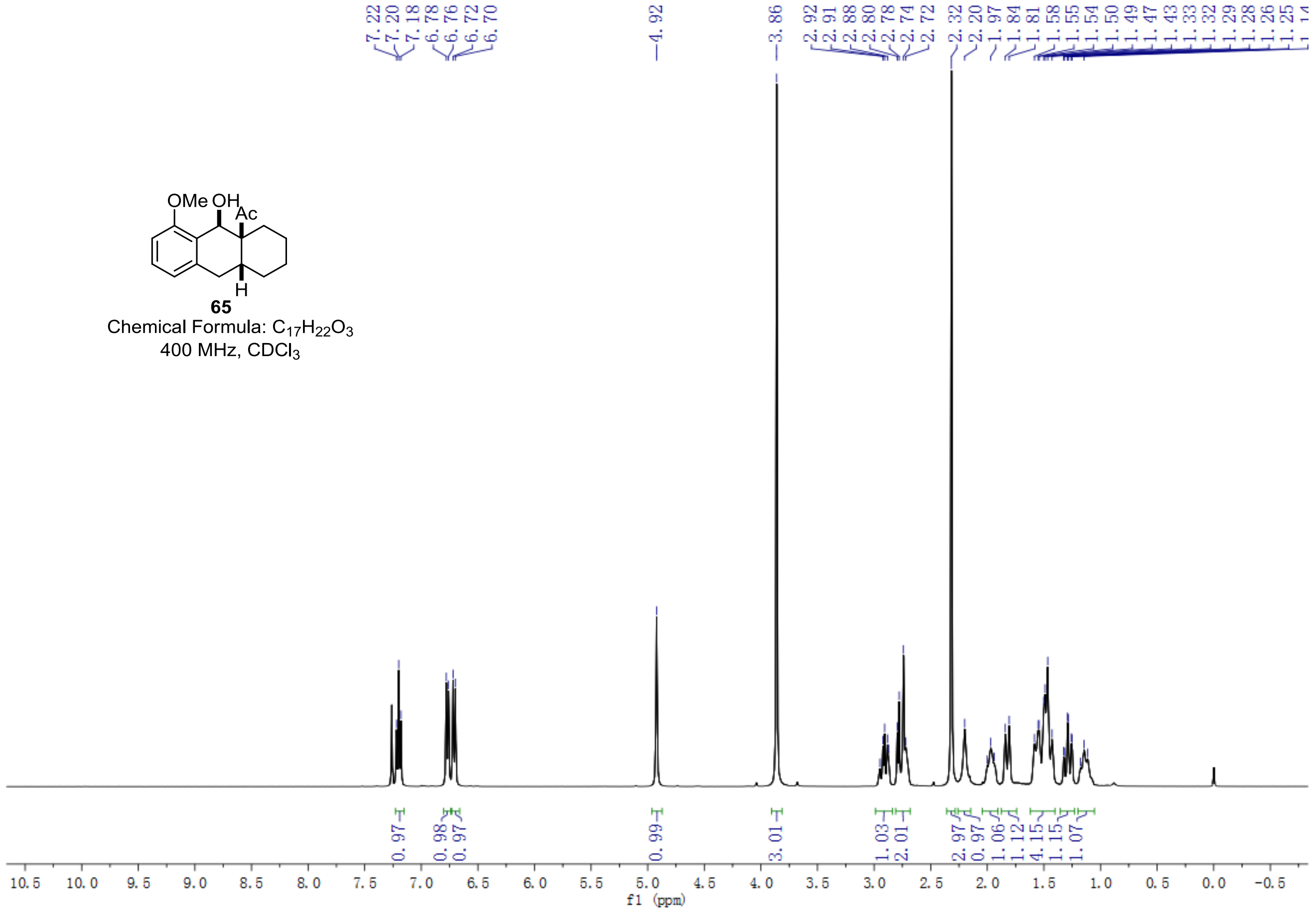

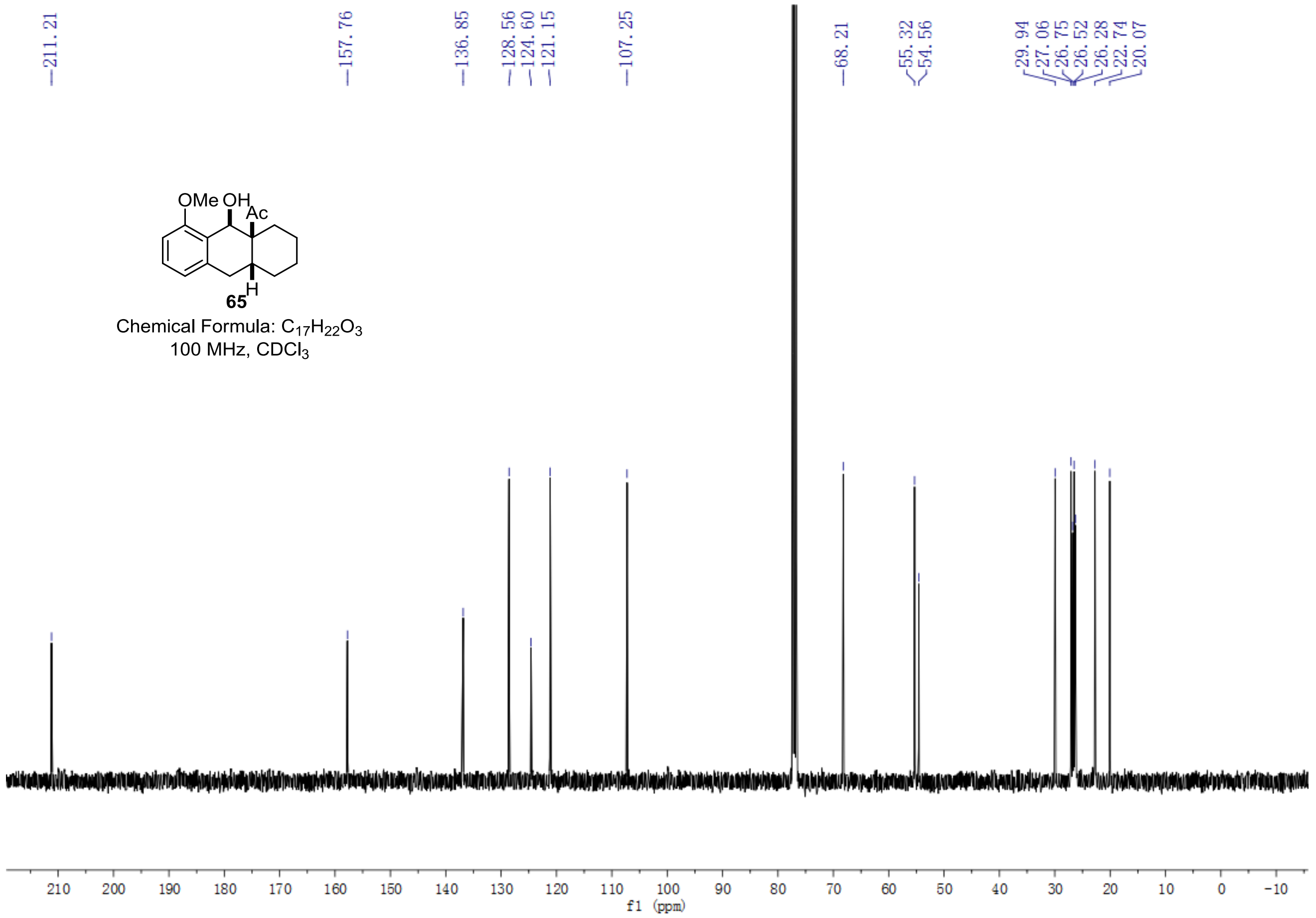

Supplementary Figure 51.^{1}H and ^{13}C NMR spectra for 65.

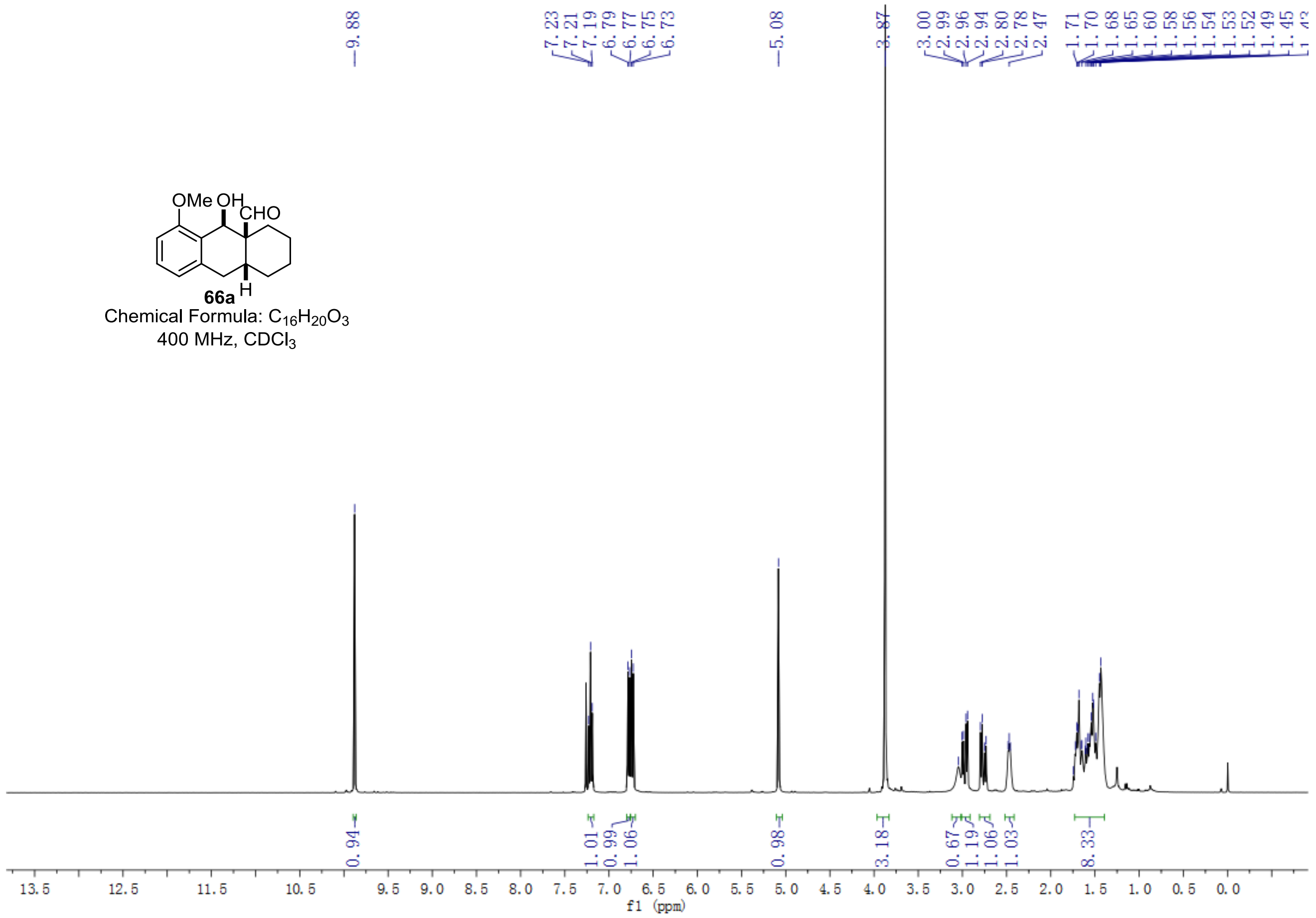

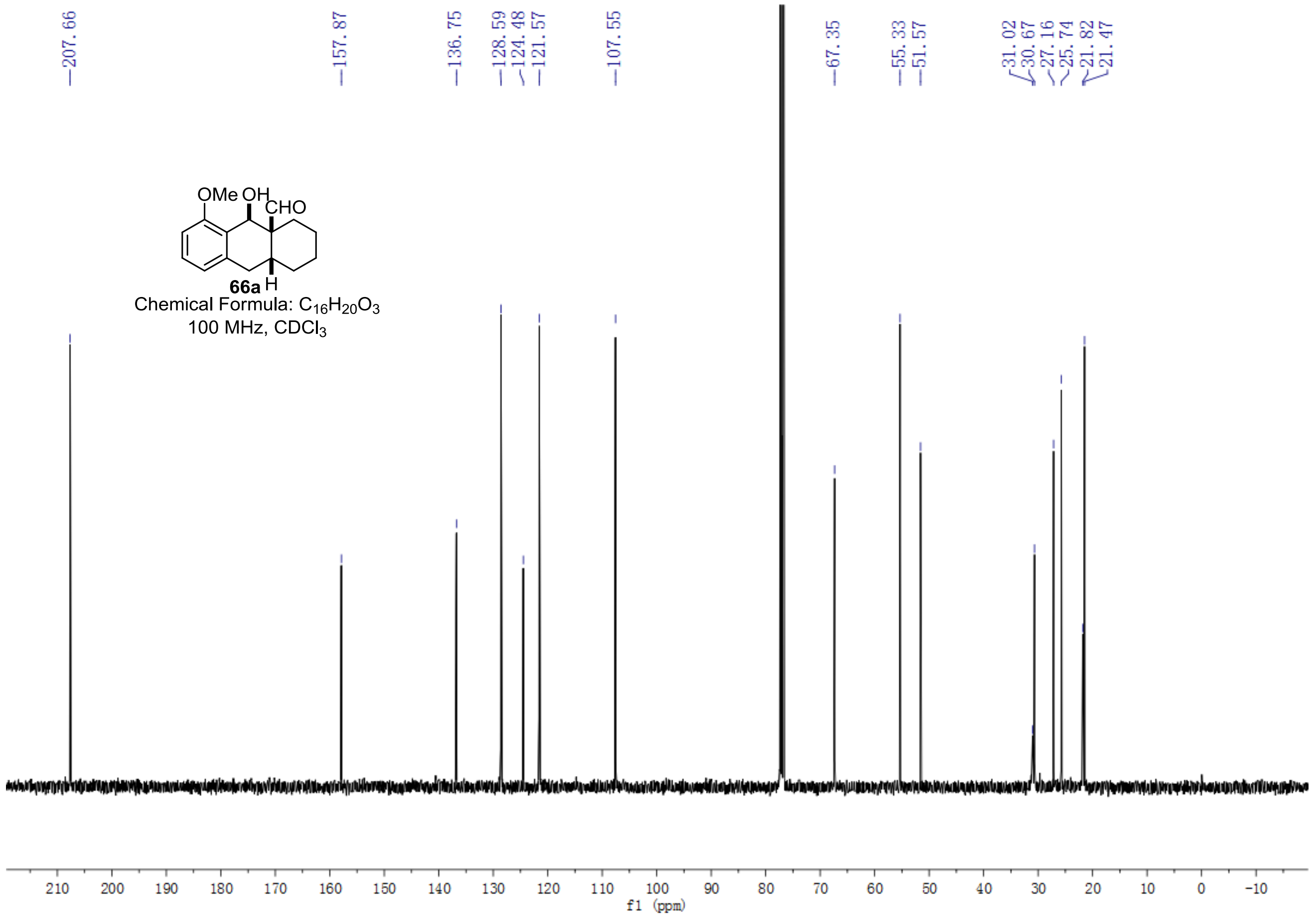

Supplementary Figure 52.[1]**H and** [13]**C NMR spectra for 66a.**

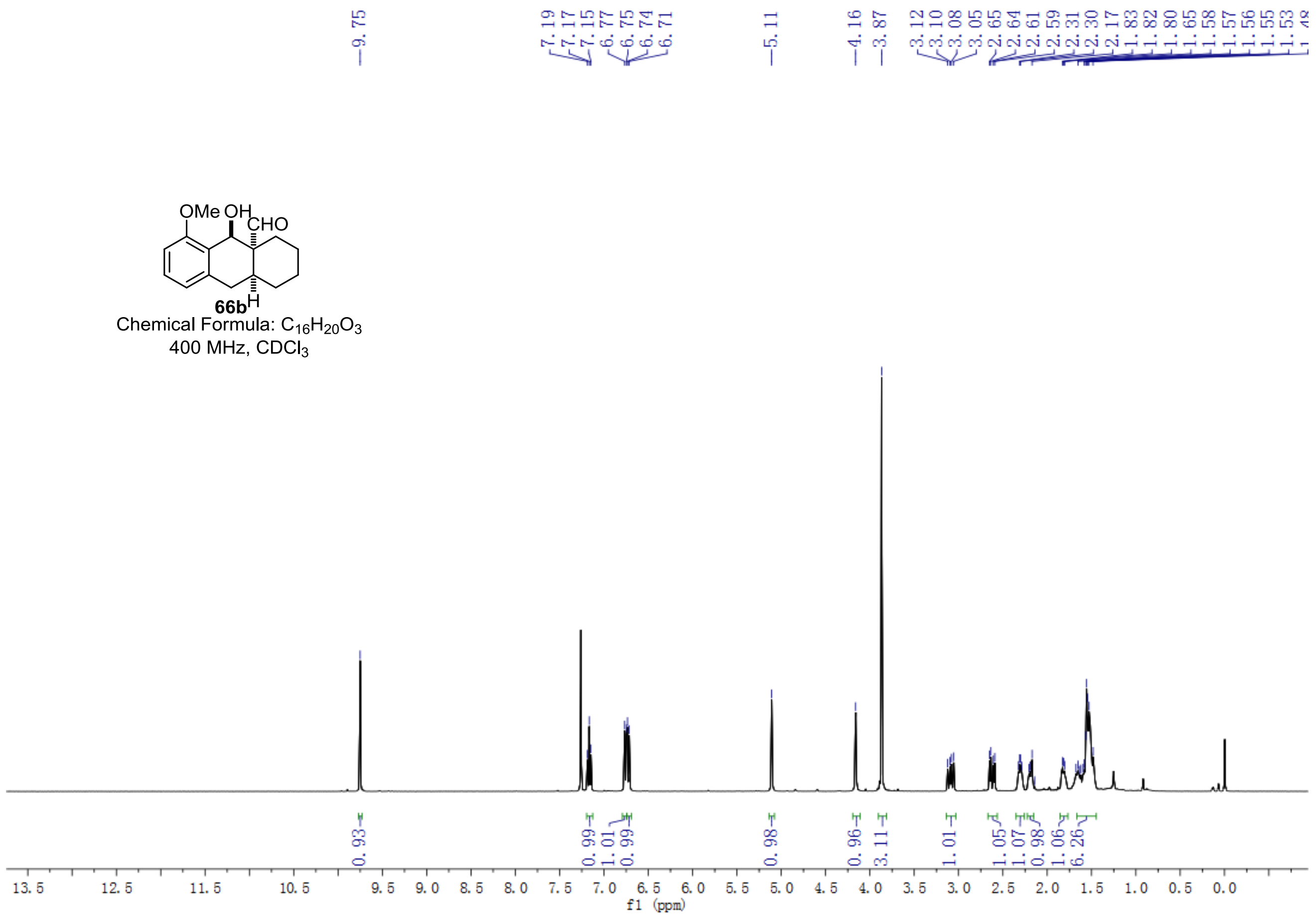

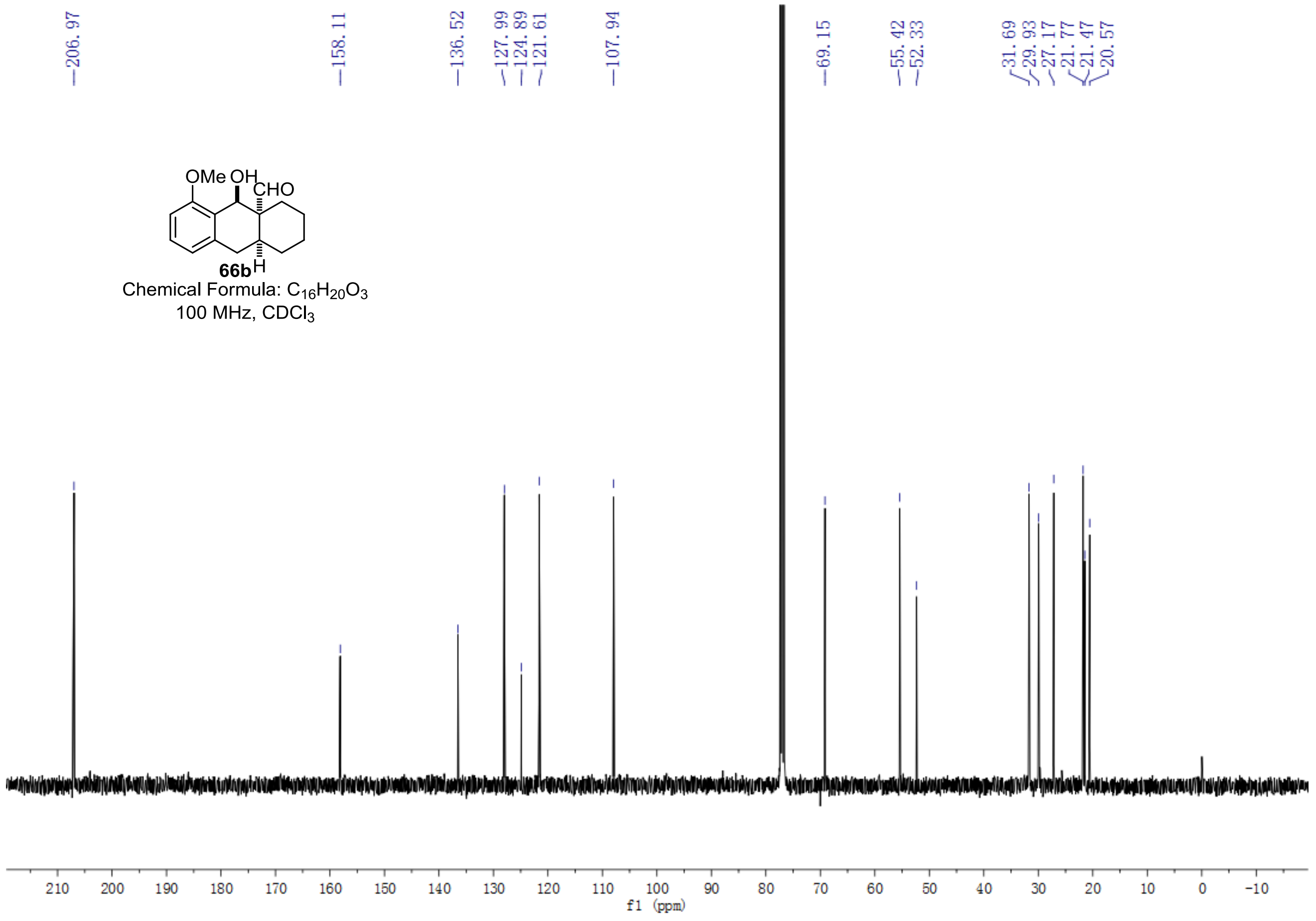

Supplementary Figure 53. ^{1}H **and** ^{13}C **NMR spectra for 66b.**

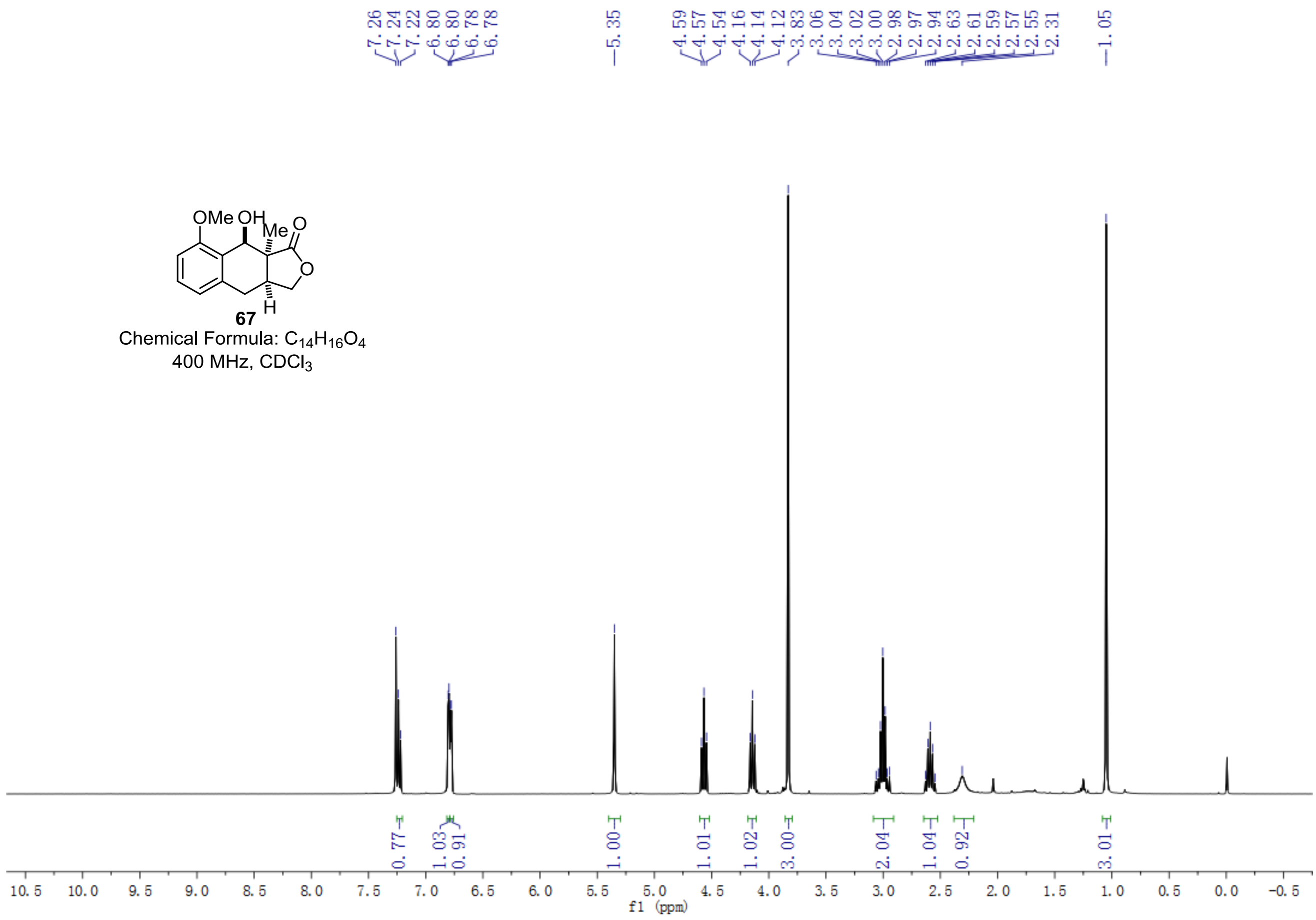

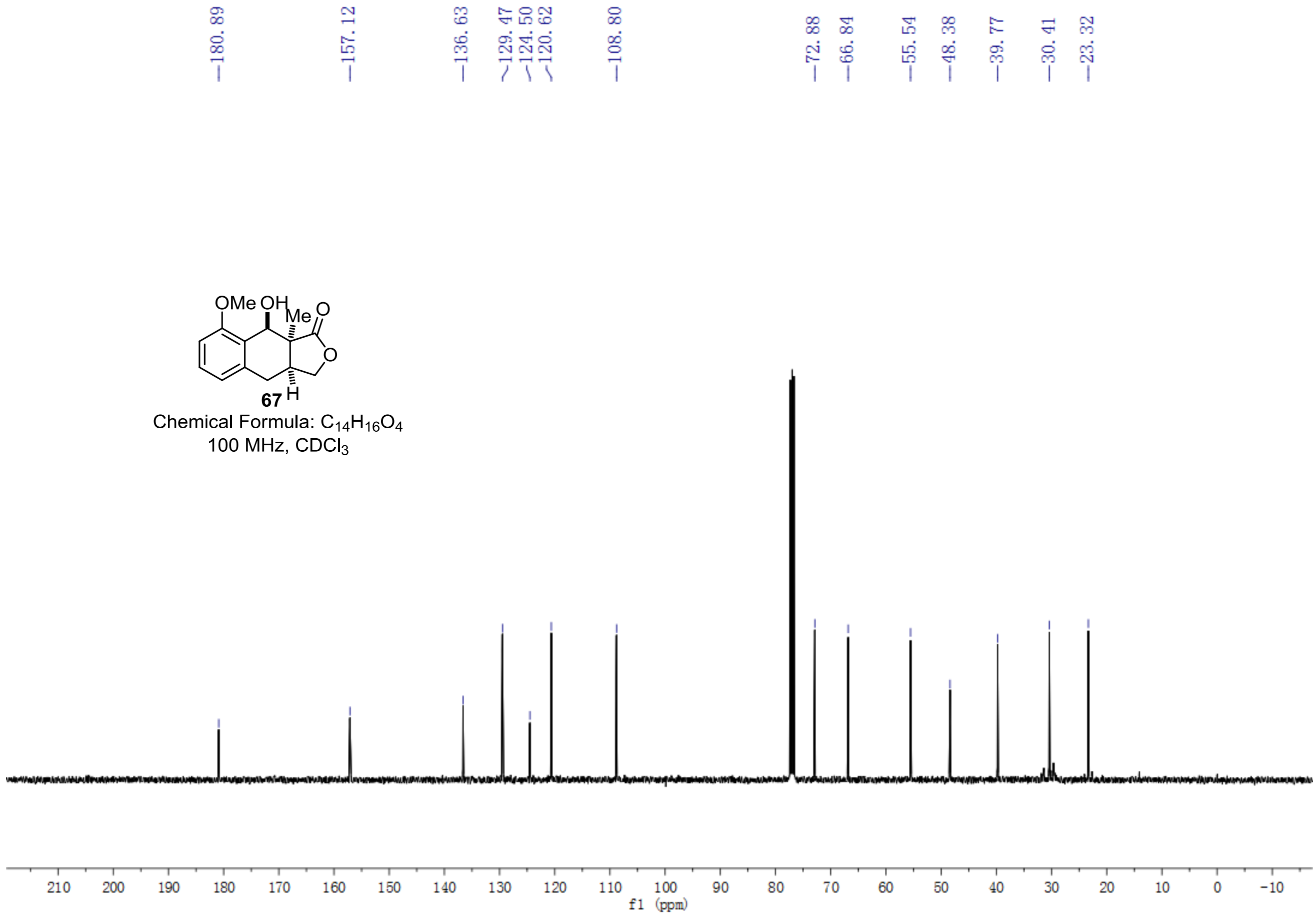

Supplementary Figure 54.^{1}H and ^{13}C NMR spectra for 67.

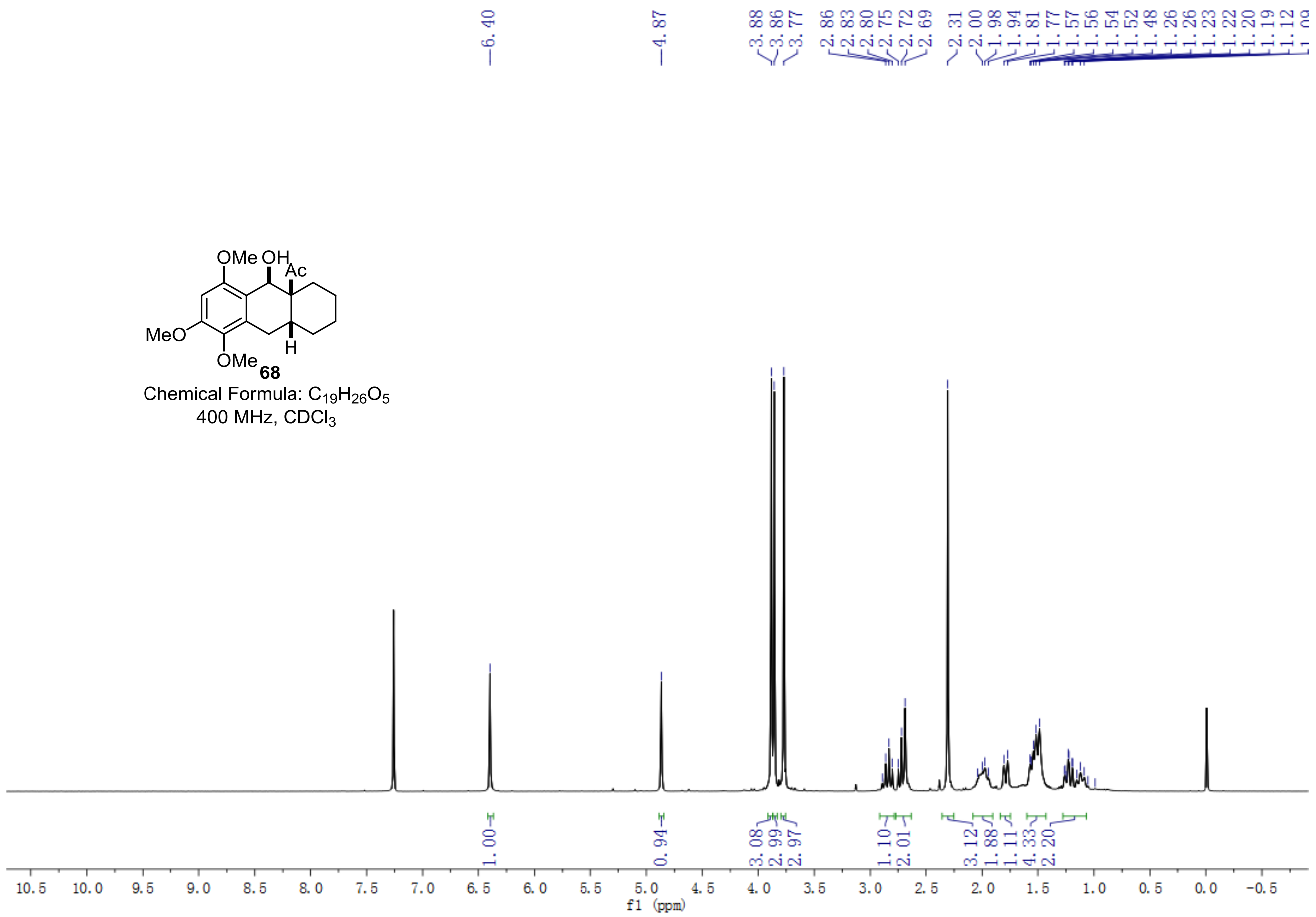

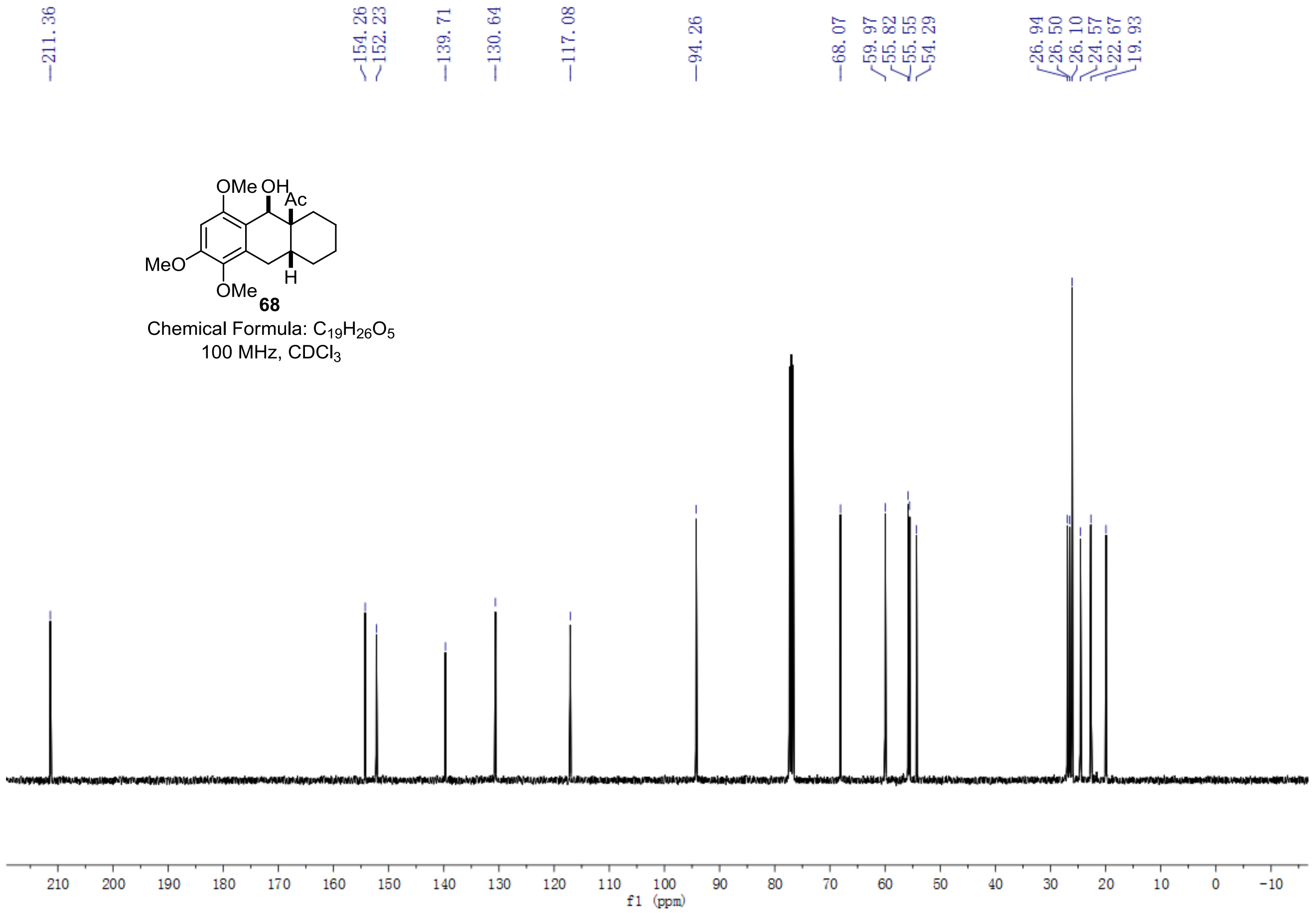

Supplementary Figure 55.^{1}H and ^{13}C NMR spectra for 68.

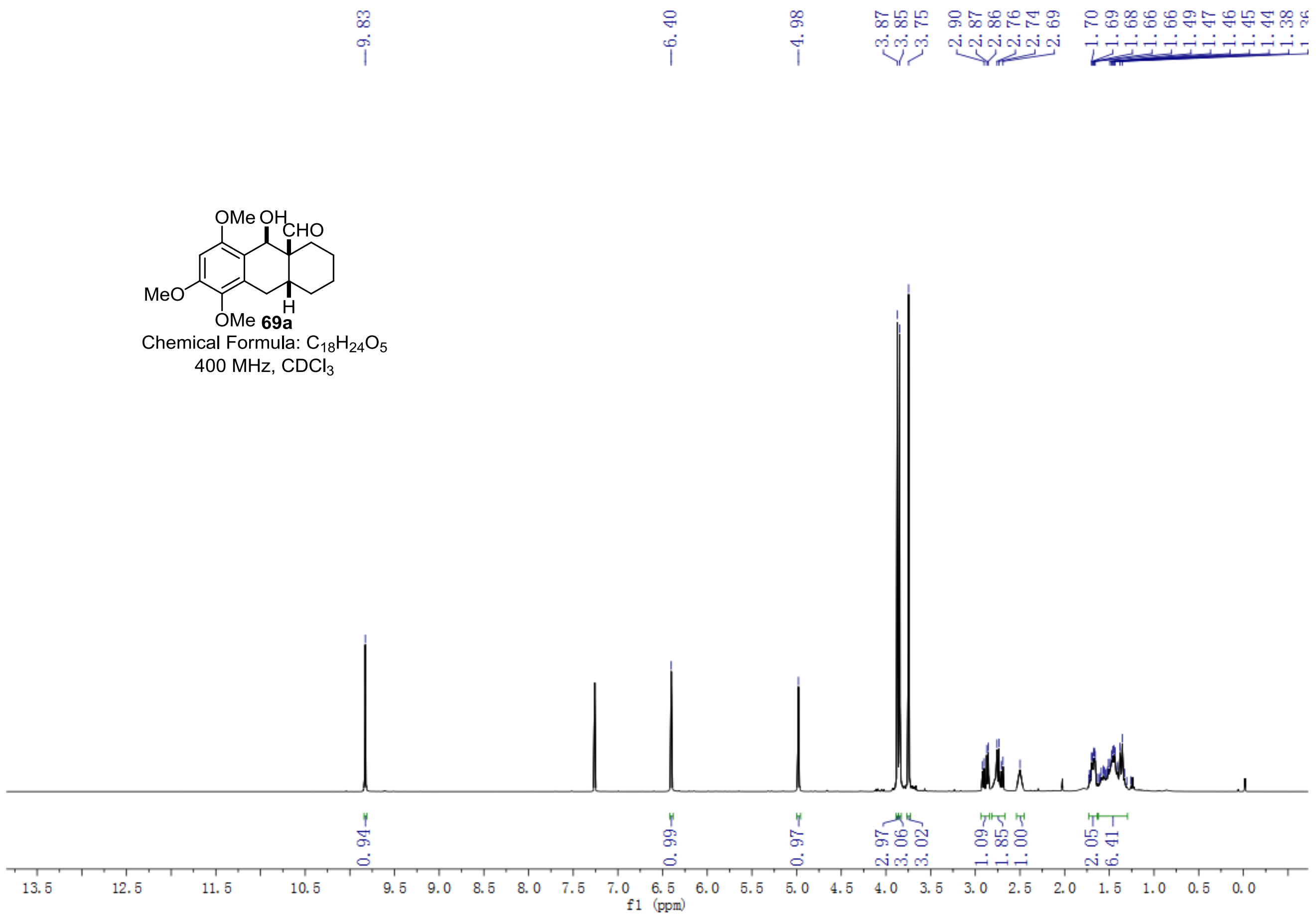

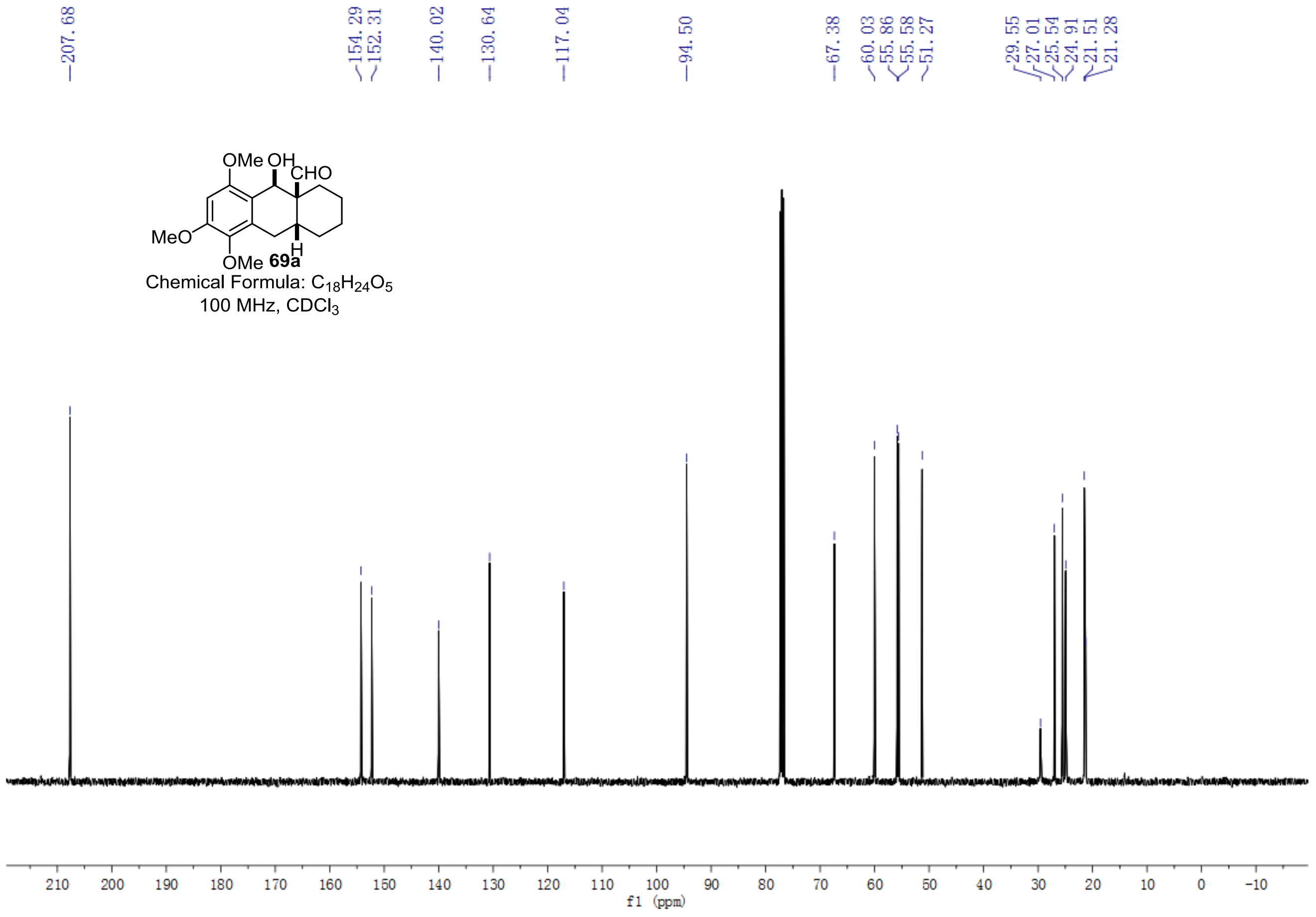

Supplementary Figure 56. ^{1}H and ^{13}C NMR spectra for 69a.

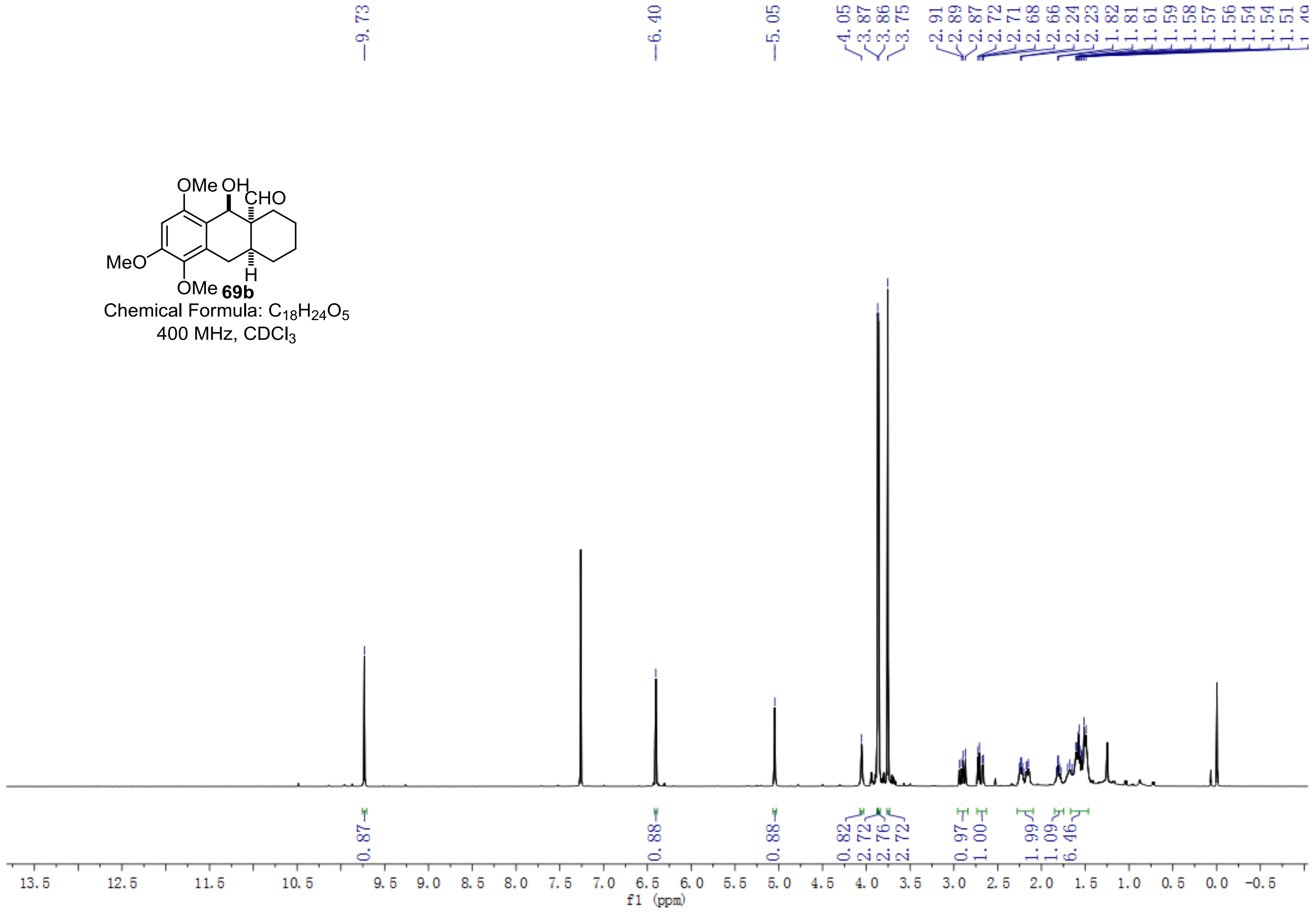

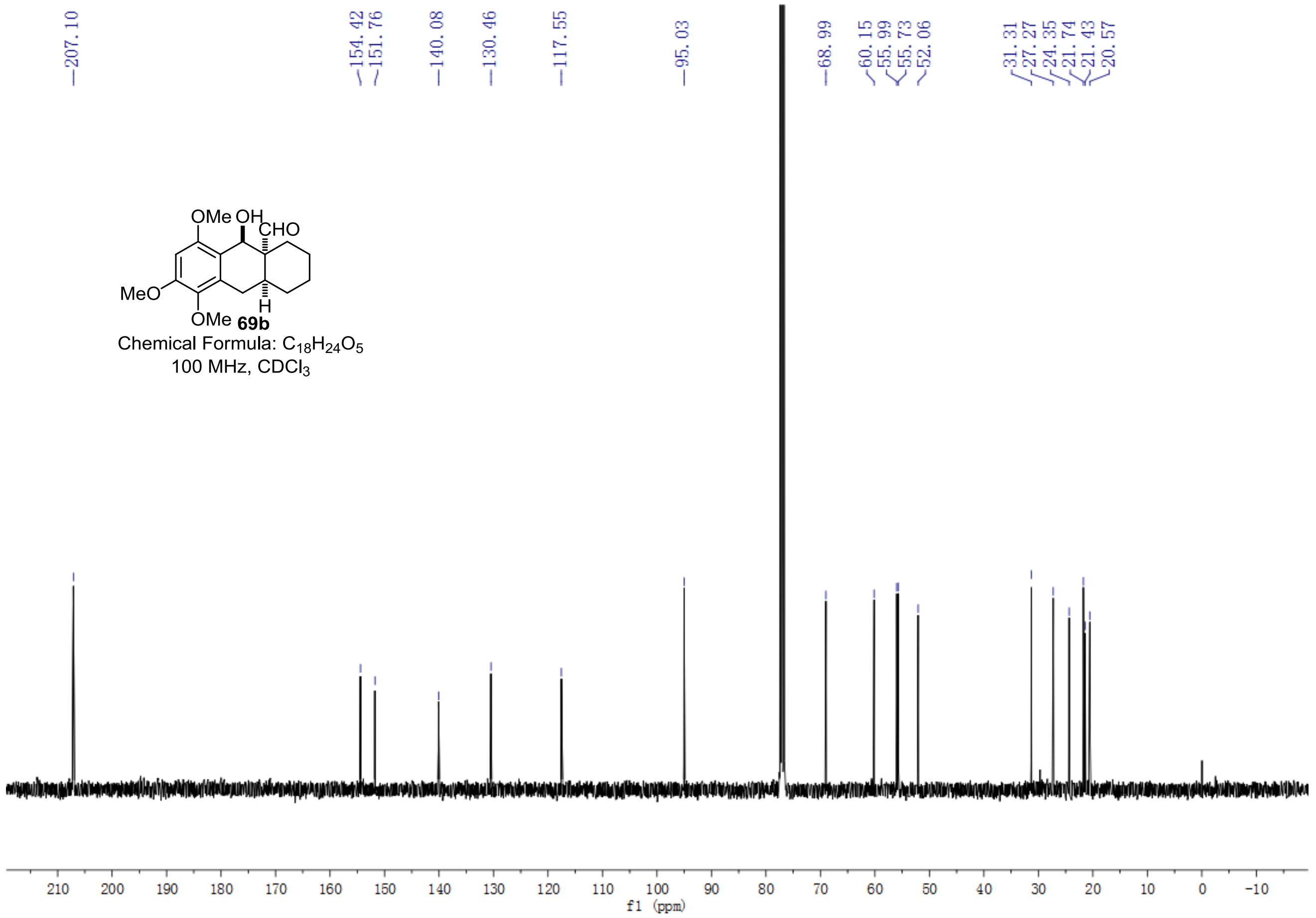

Supplementary Figure 57. ^{1}**H and** ^{13}**C NMR spectra for 69b.**

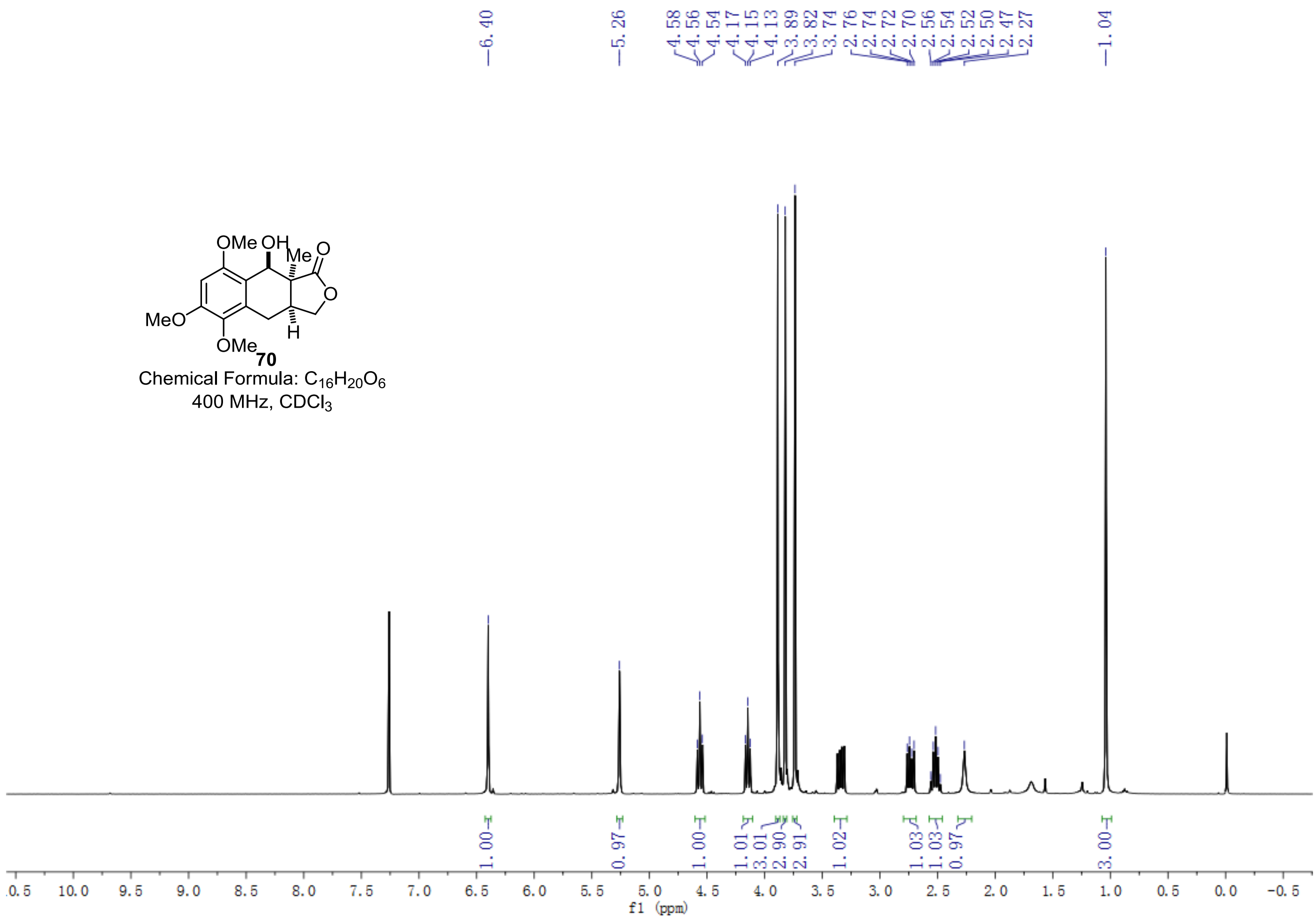

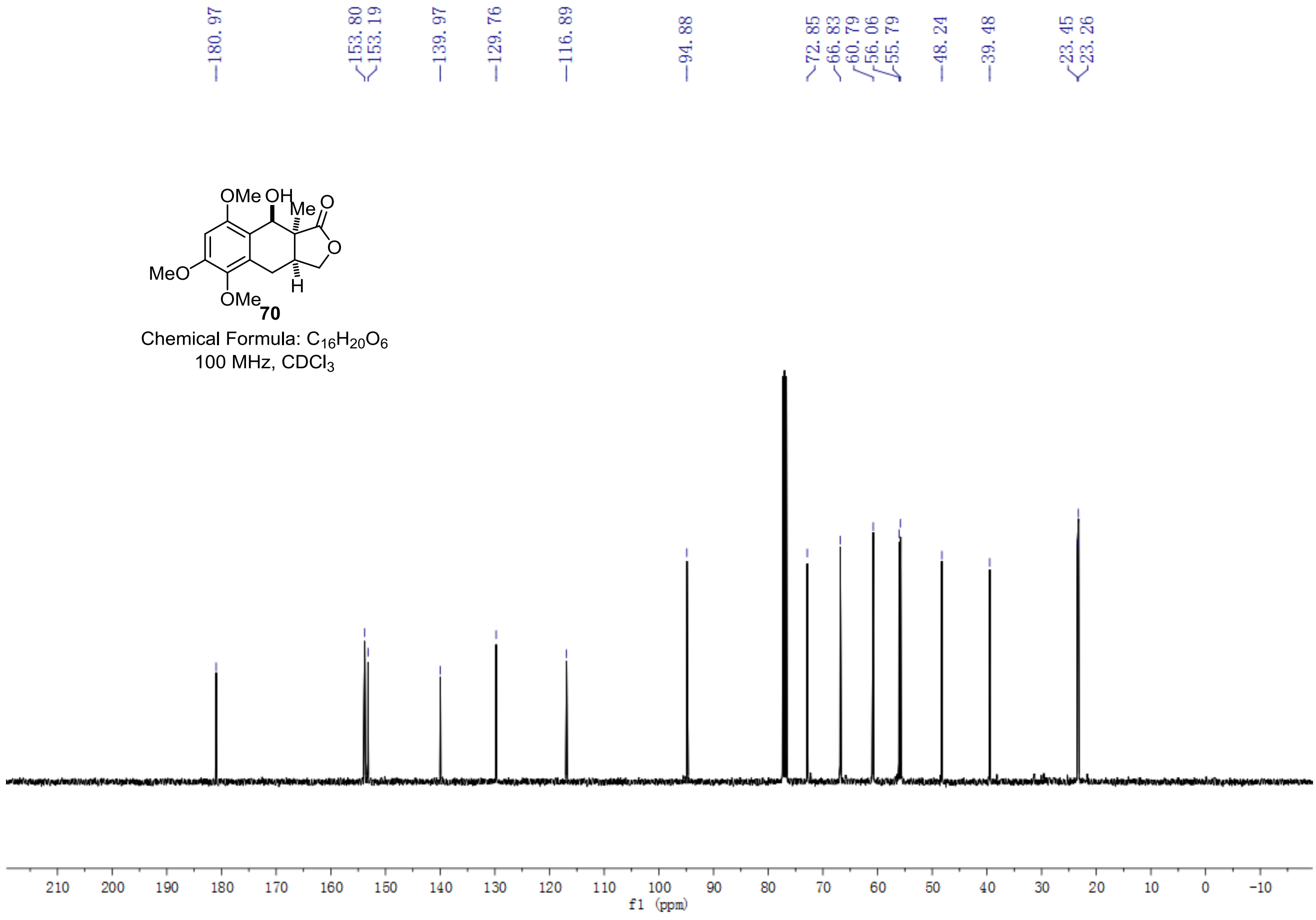

Supplementary Figure 58.^{1}**H and** ^{13}**C NMR spectra for 70.**

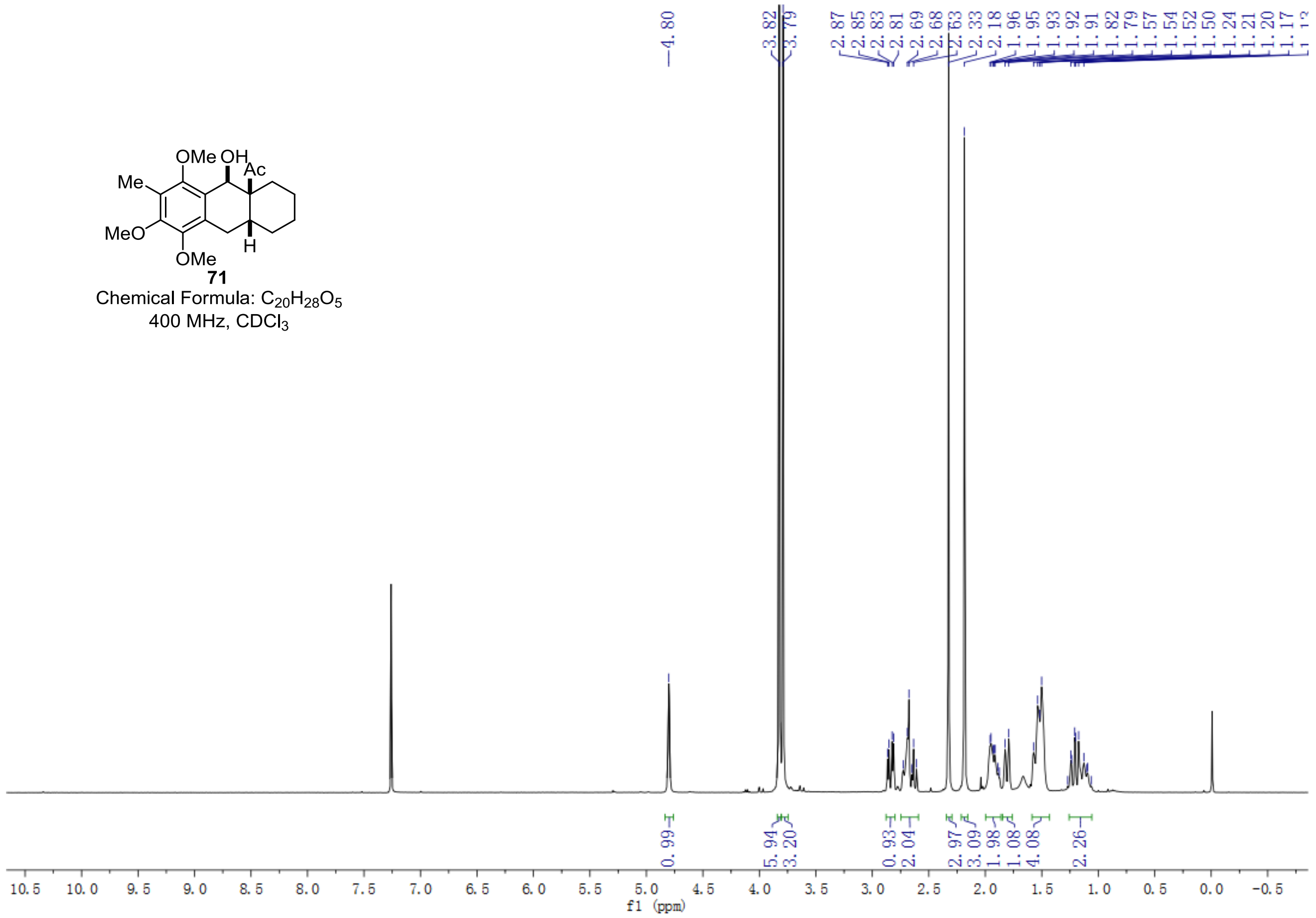

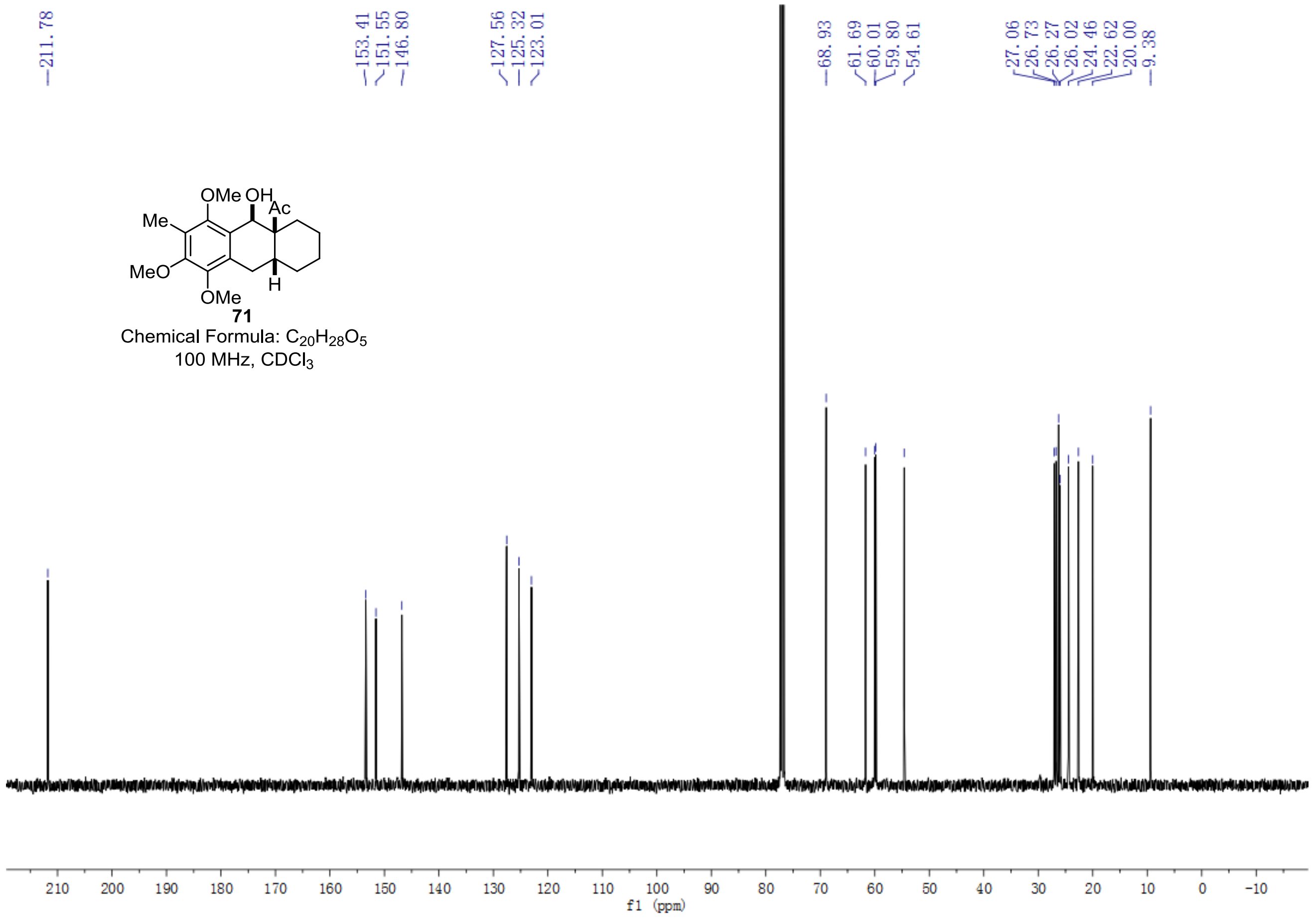

Supplementary Figure 59.^{1}H and ^{13}C NMR spectra for 71.

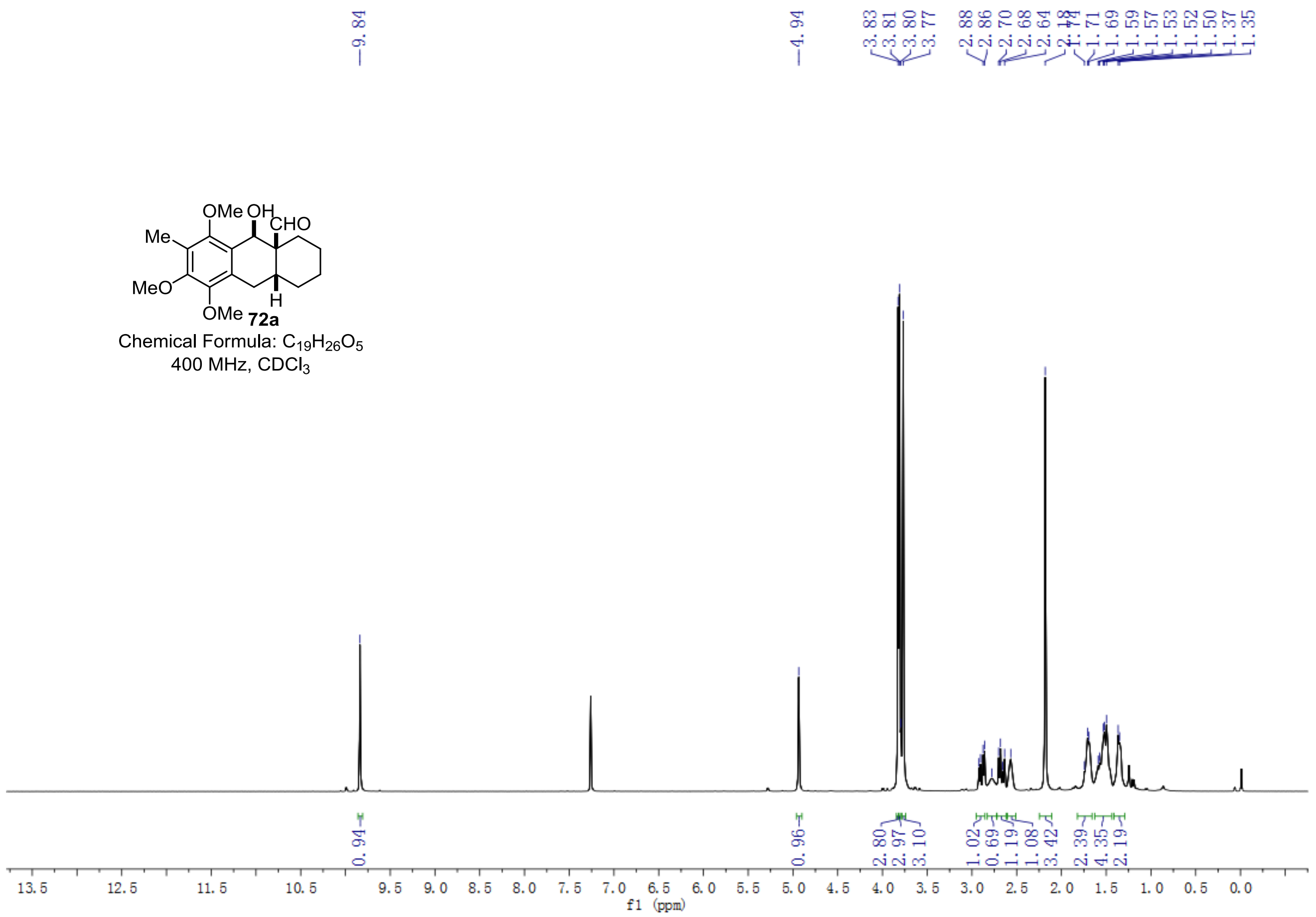

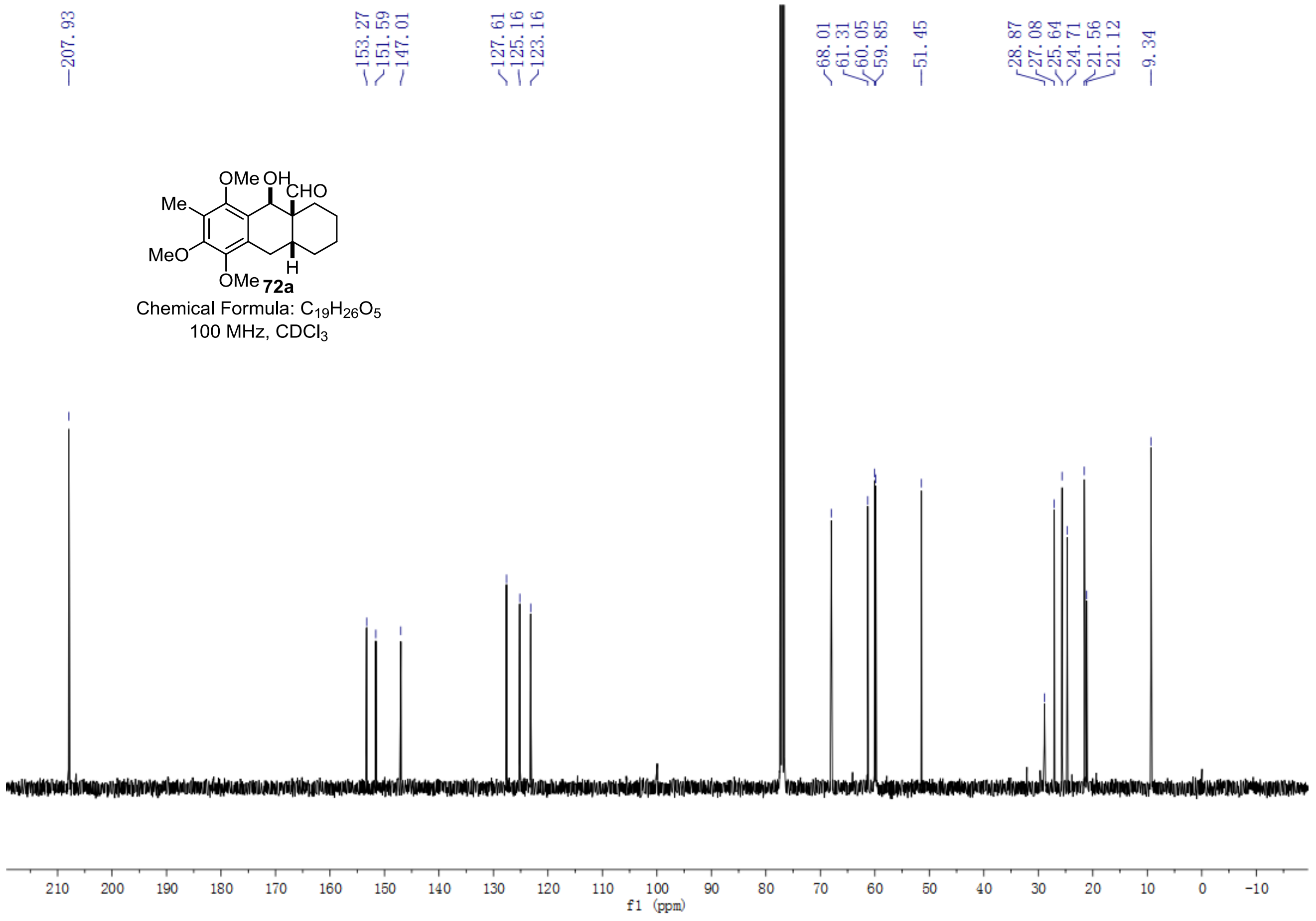

Supplementary Figure 60.^{1}H and ^{13}C NMR spectra for 72a.

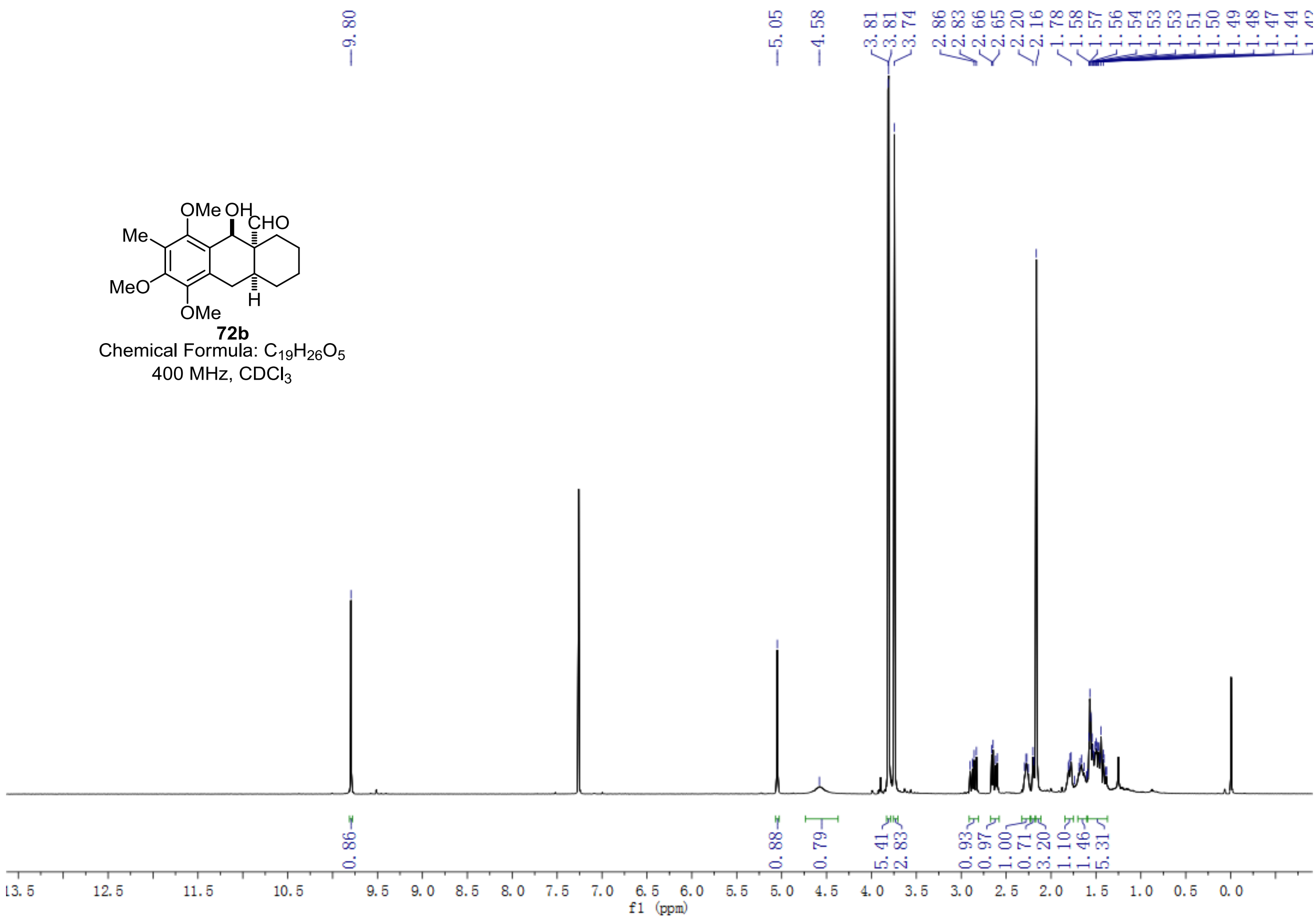

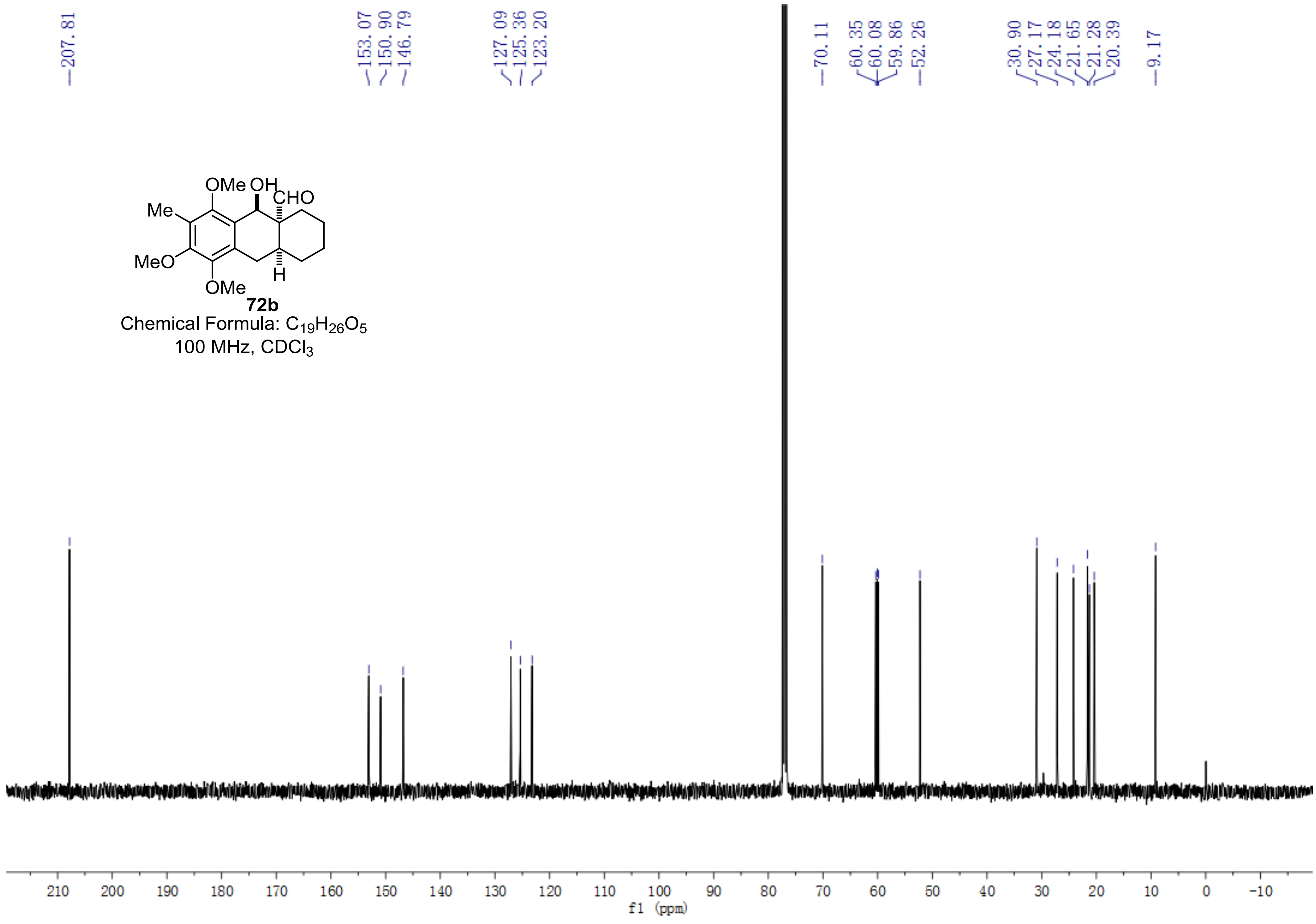

Supplementary Figure 61.^{1}H and ^{13}C NMR spectra for 72b.

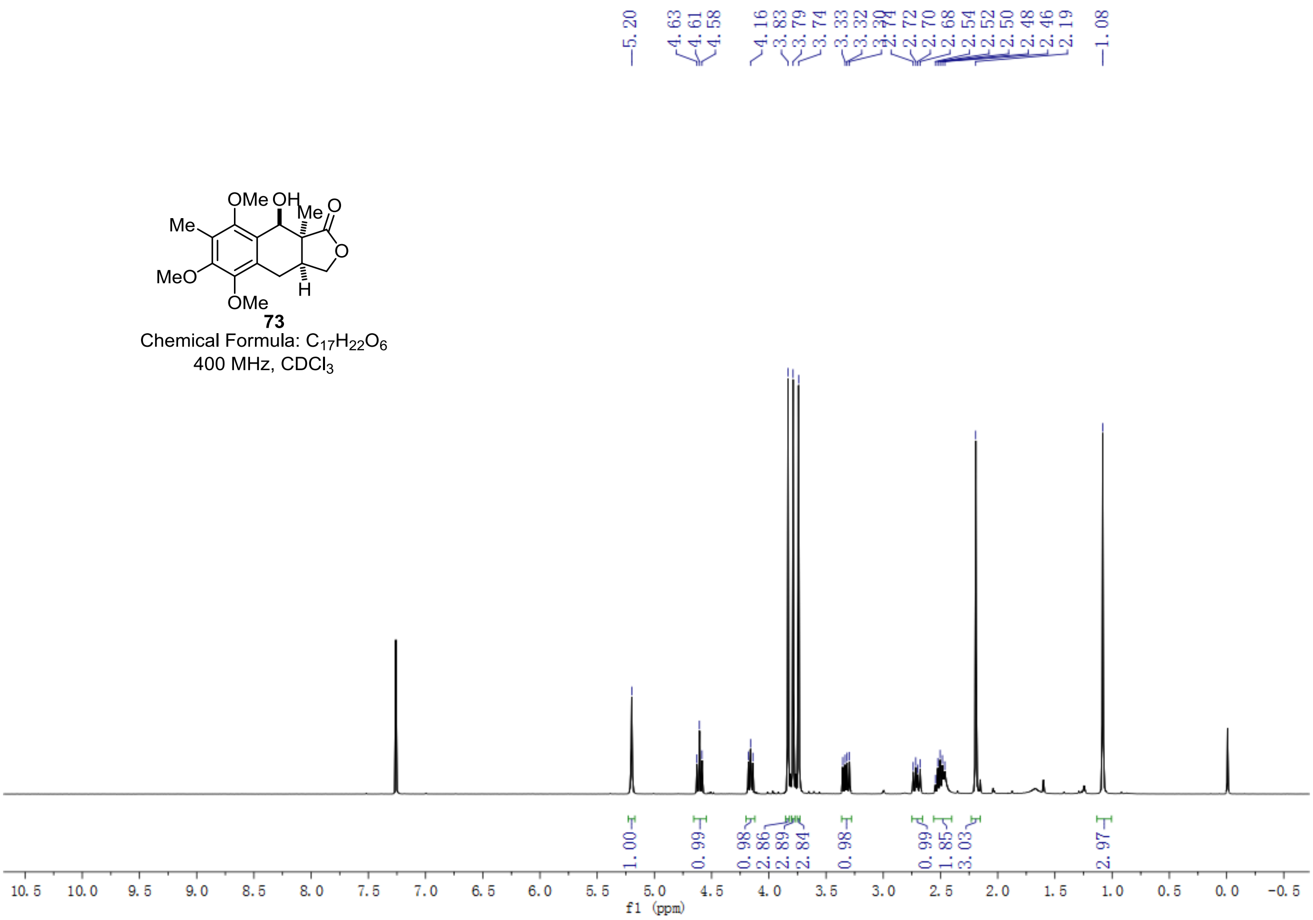

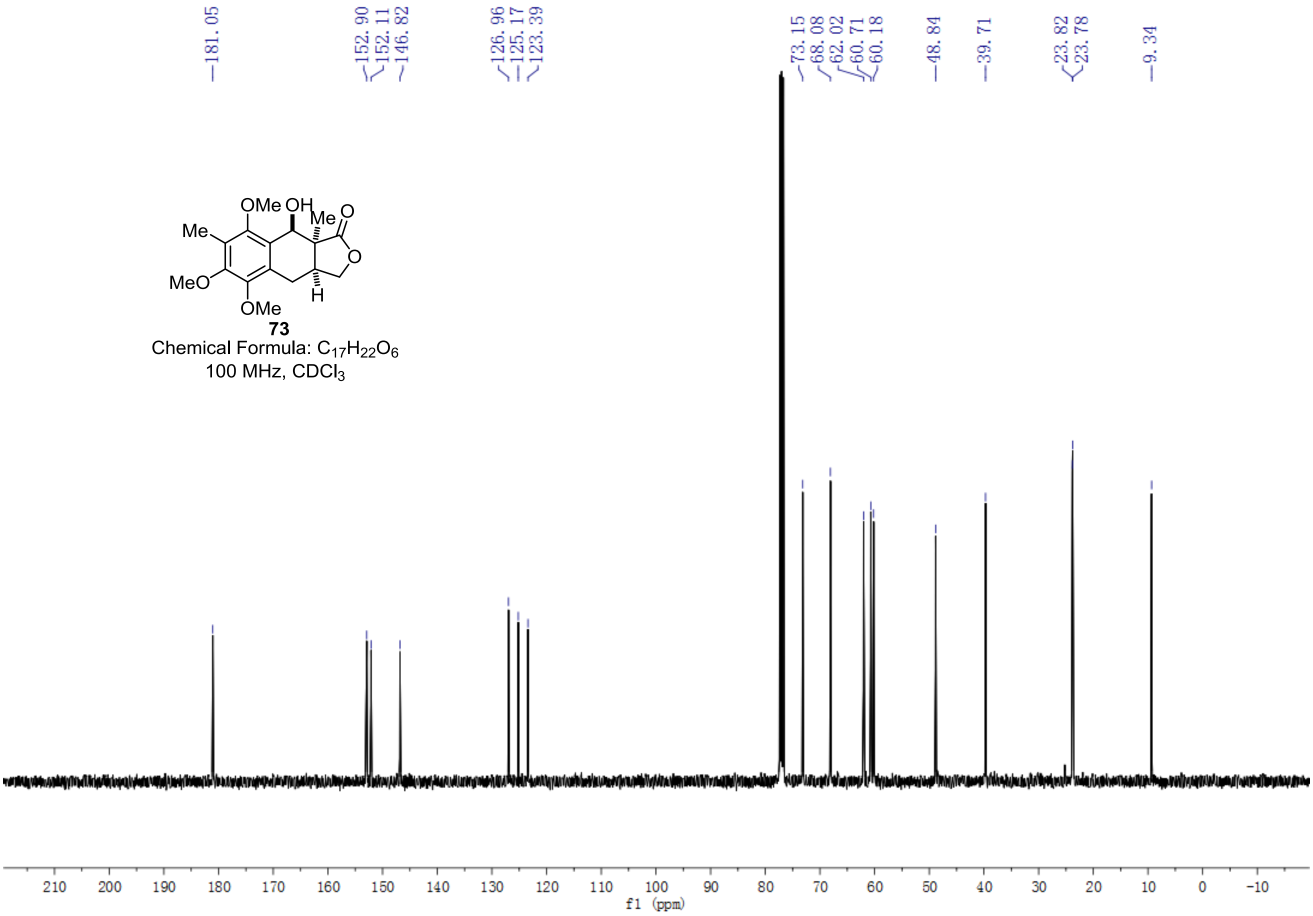

Supplementary Figure 62. ^{1}H **and** ^{13}C **NMR spectra for 73.**

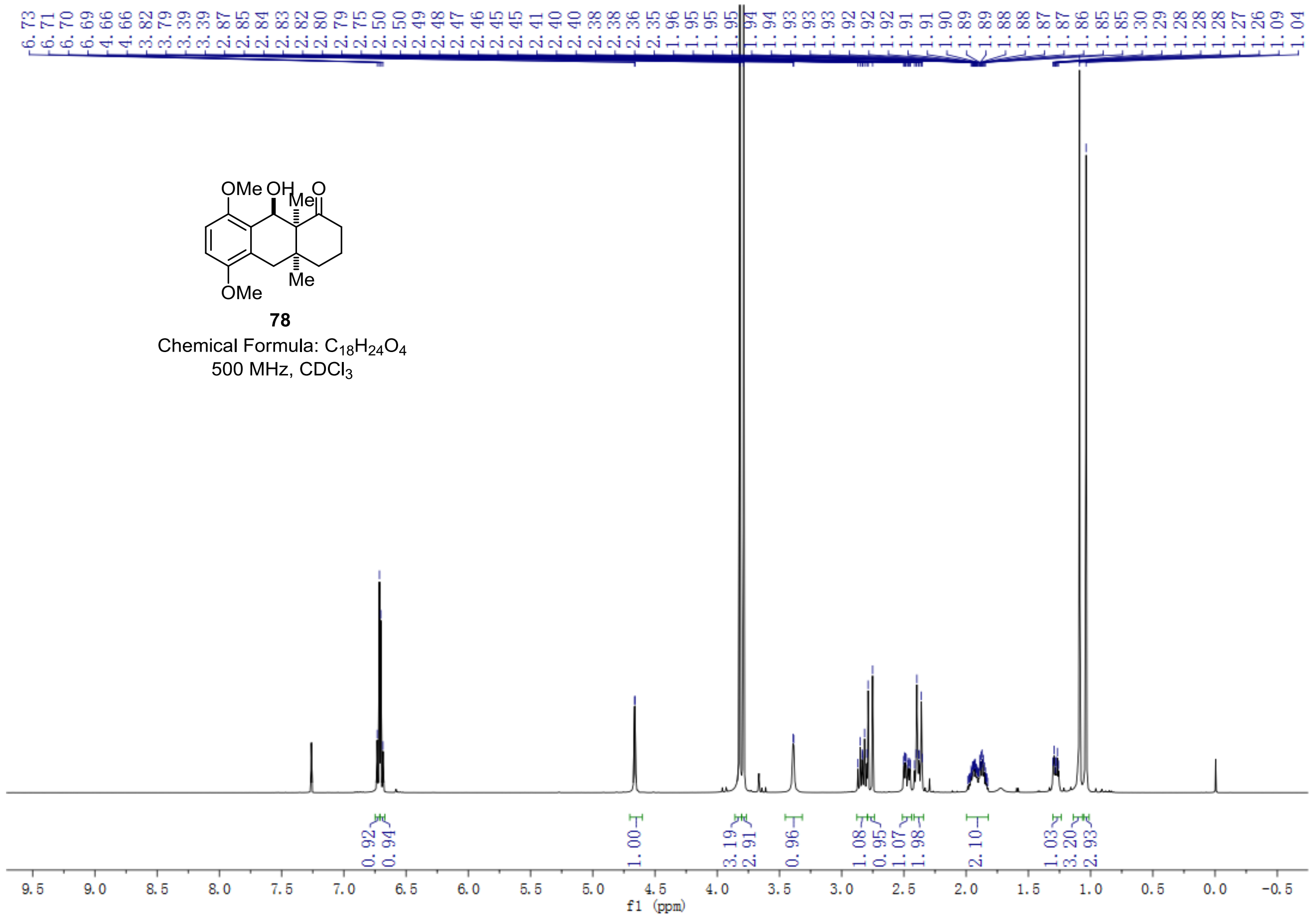

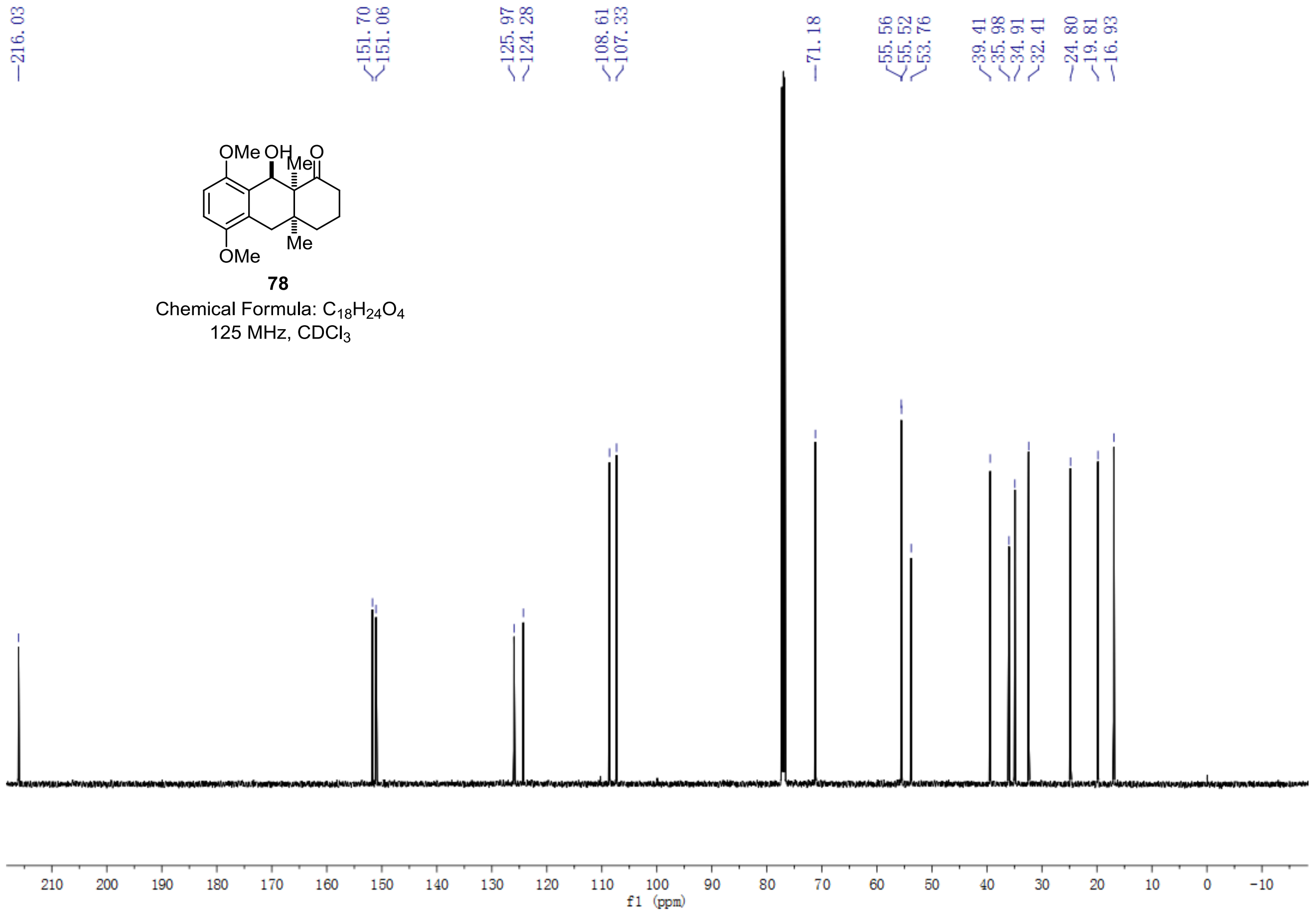

Supplementary Figure 63. ^{1}H and ^{13}C NMR spectra for 78.

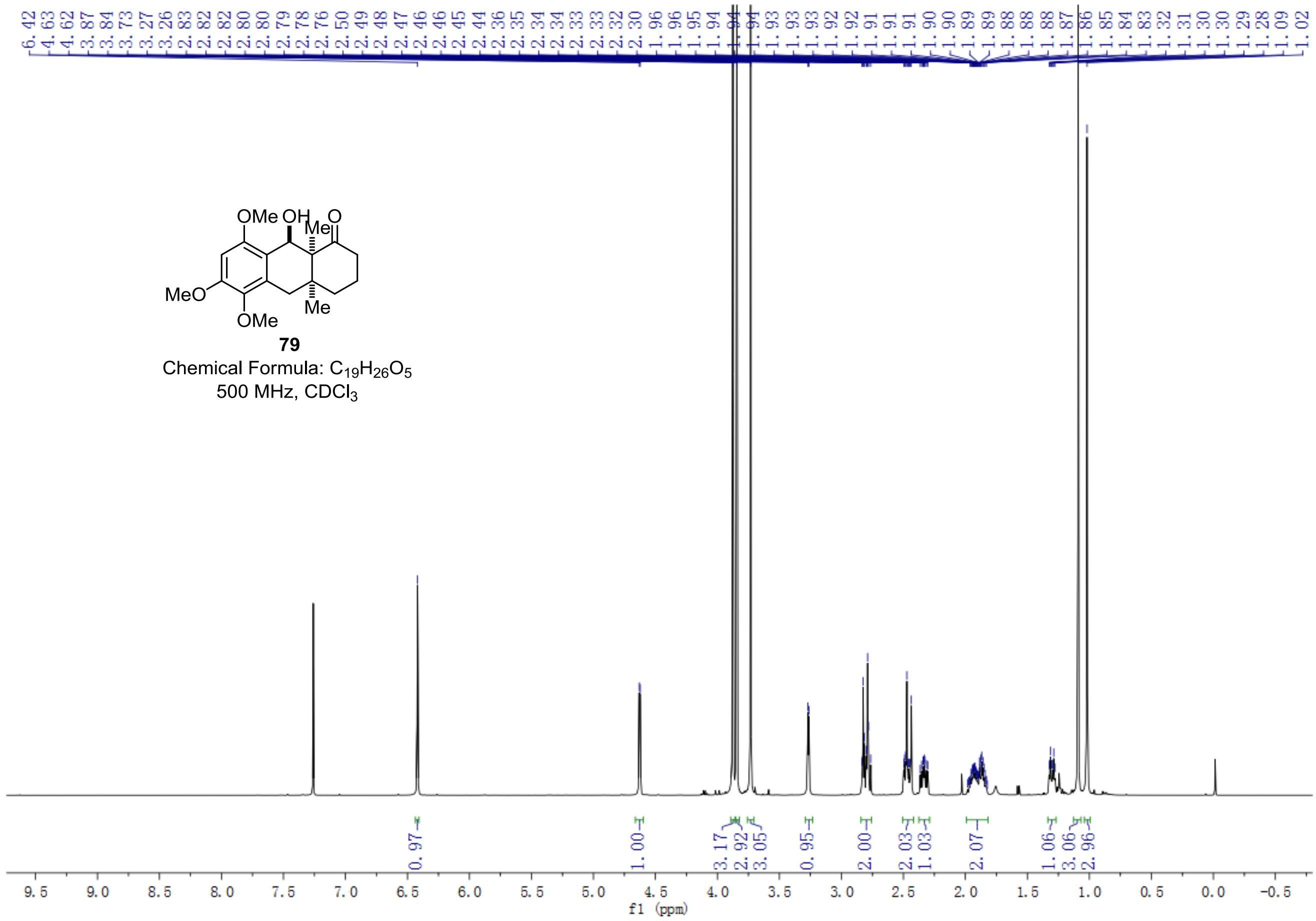

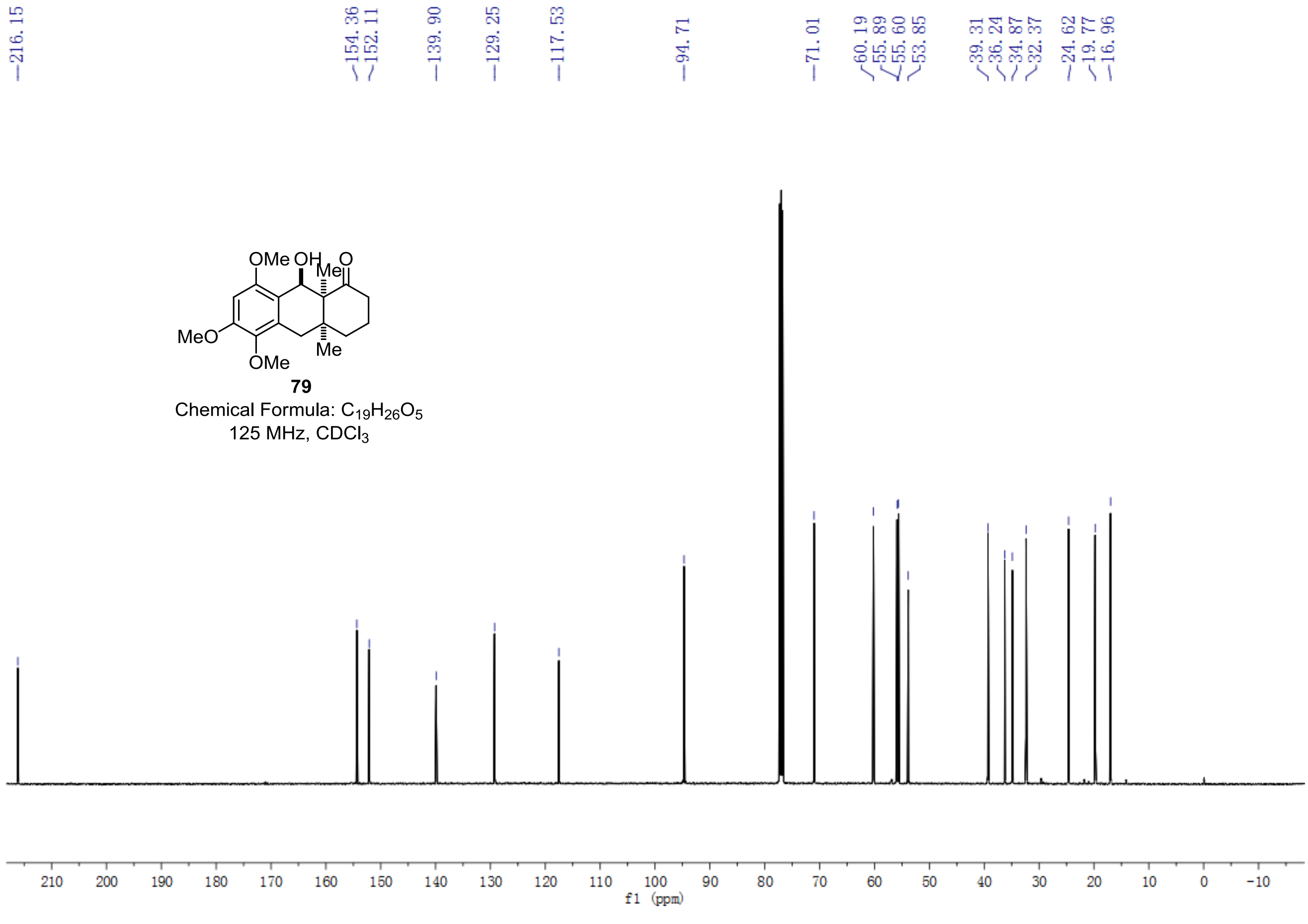

Supplementary Figure 64.^{1}**H and** ^{13}**C NMR spectra for 79.**

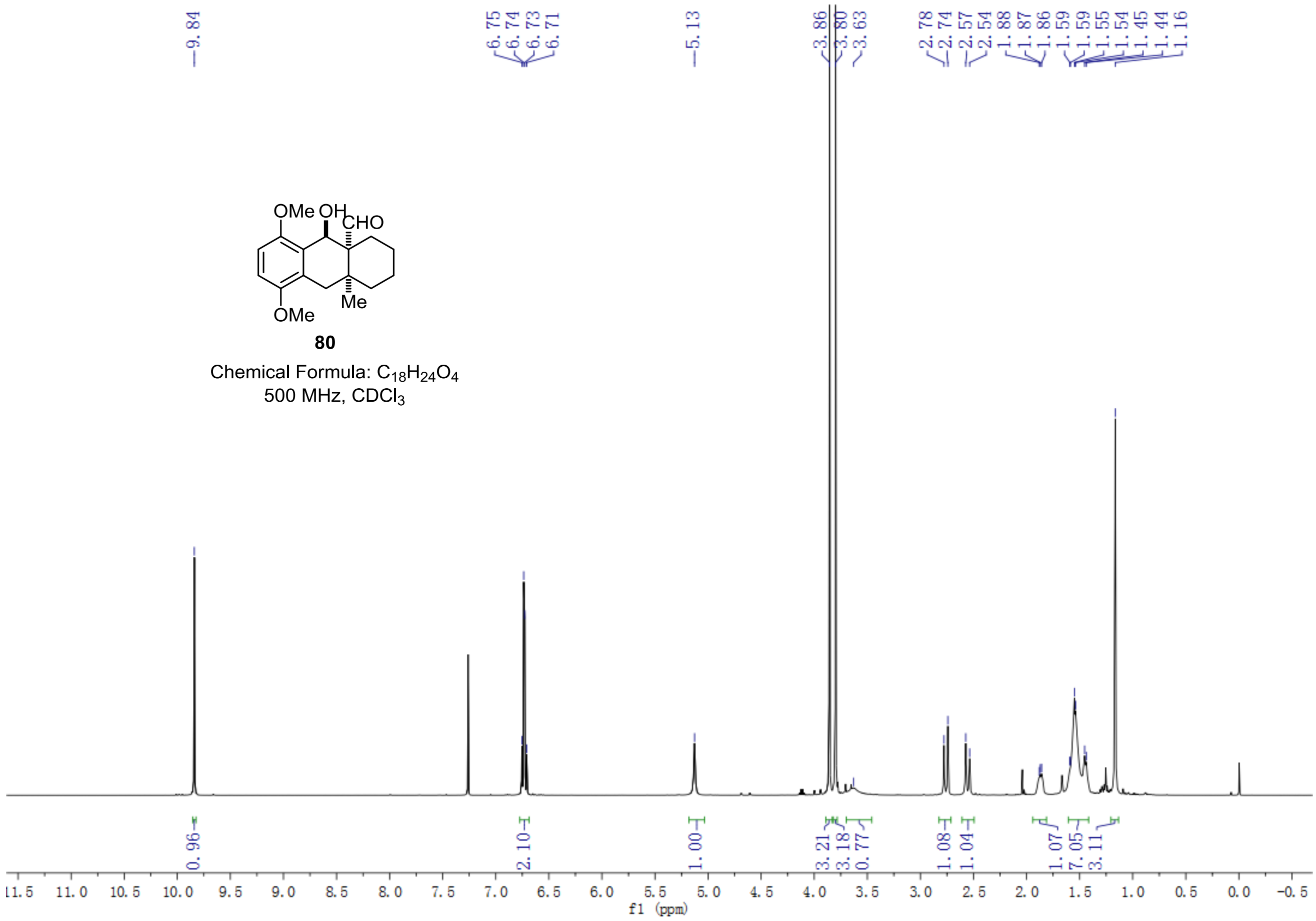

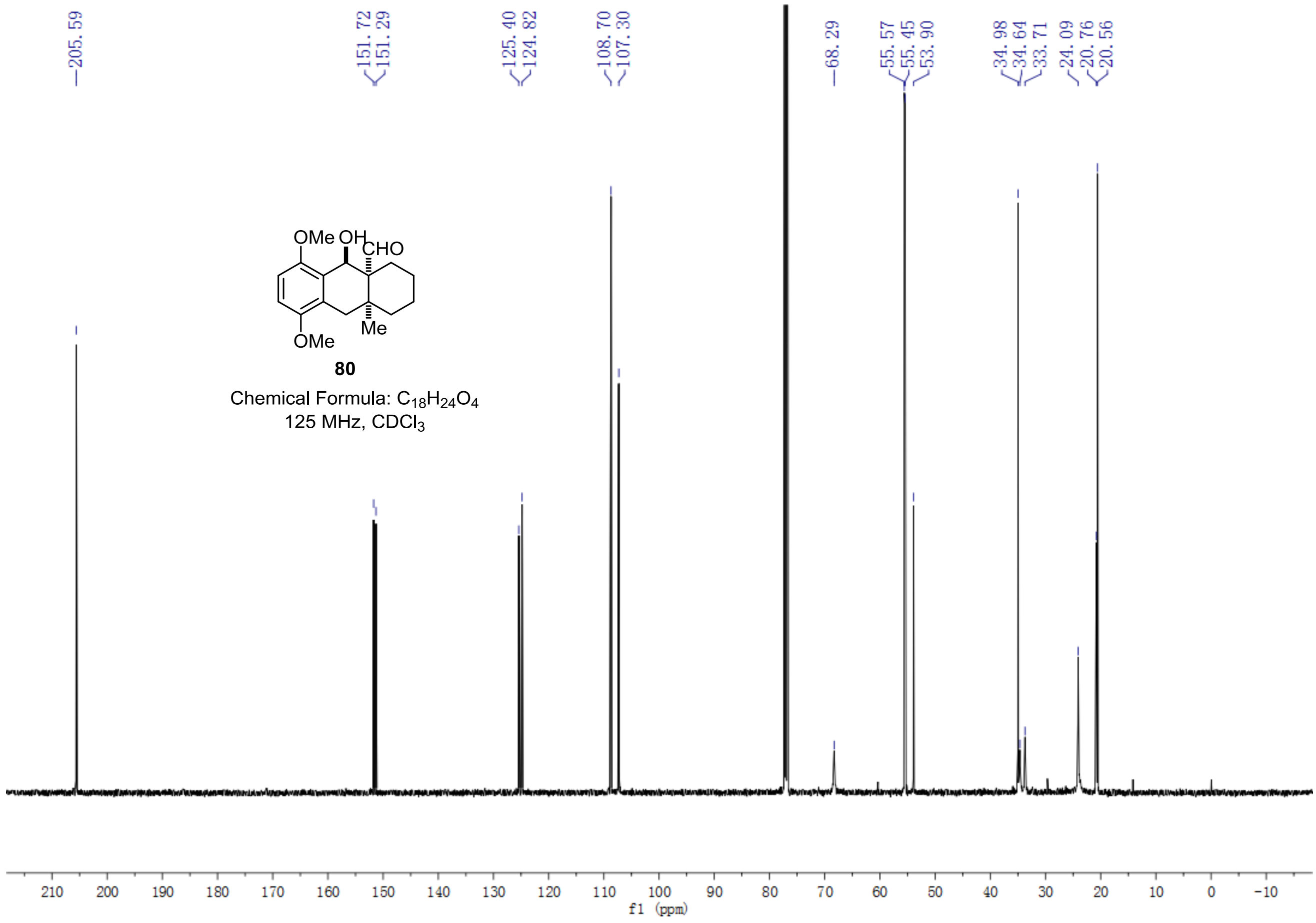

Supplementary Figure 65.^{1}H and ^{13}C NMR spectra for 80.

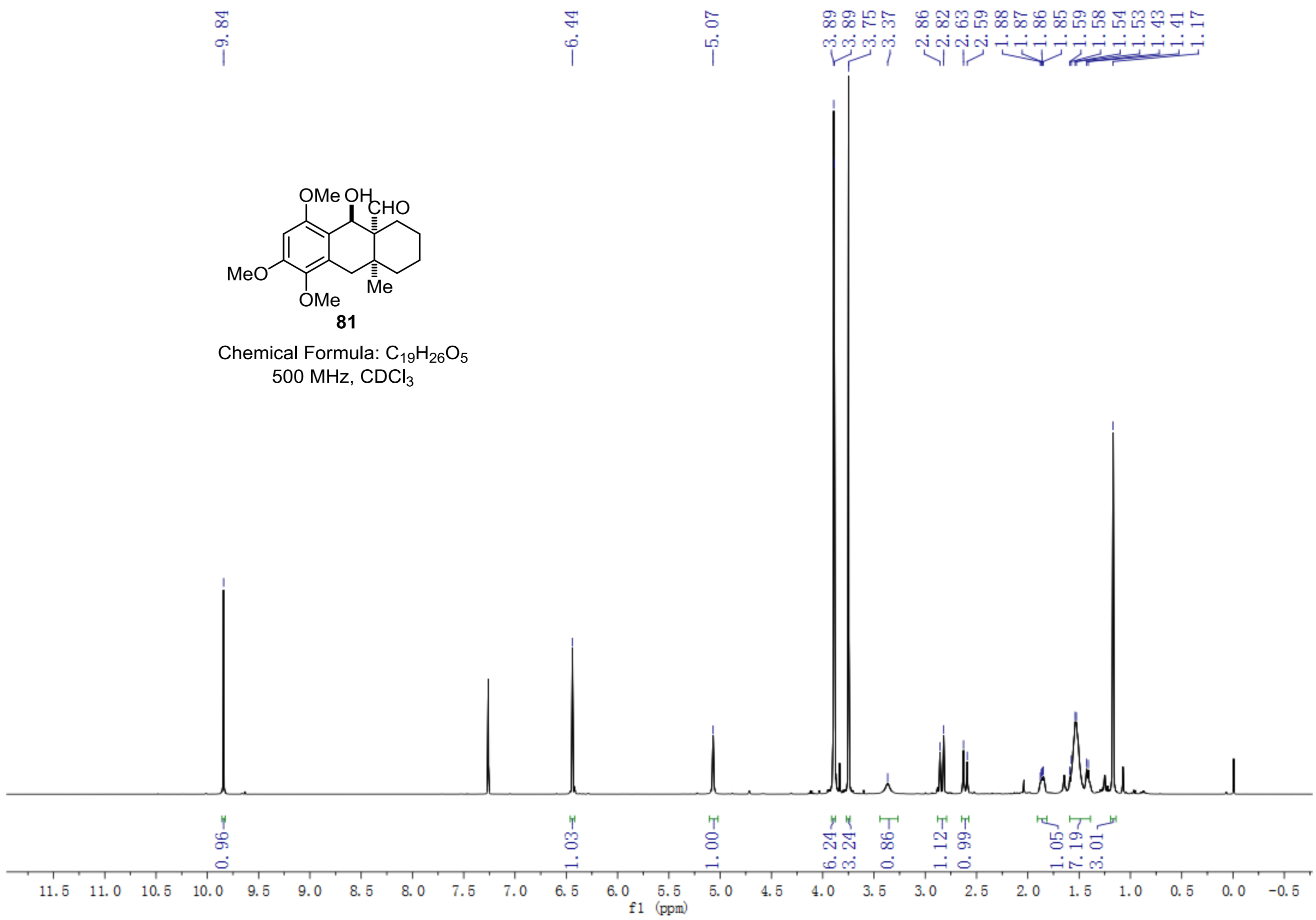

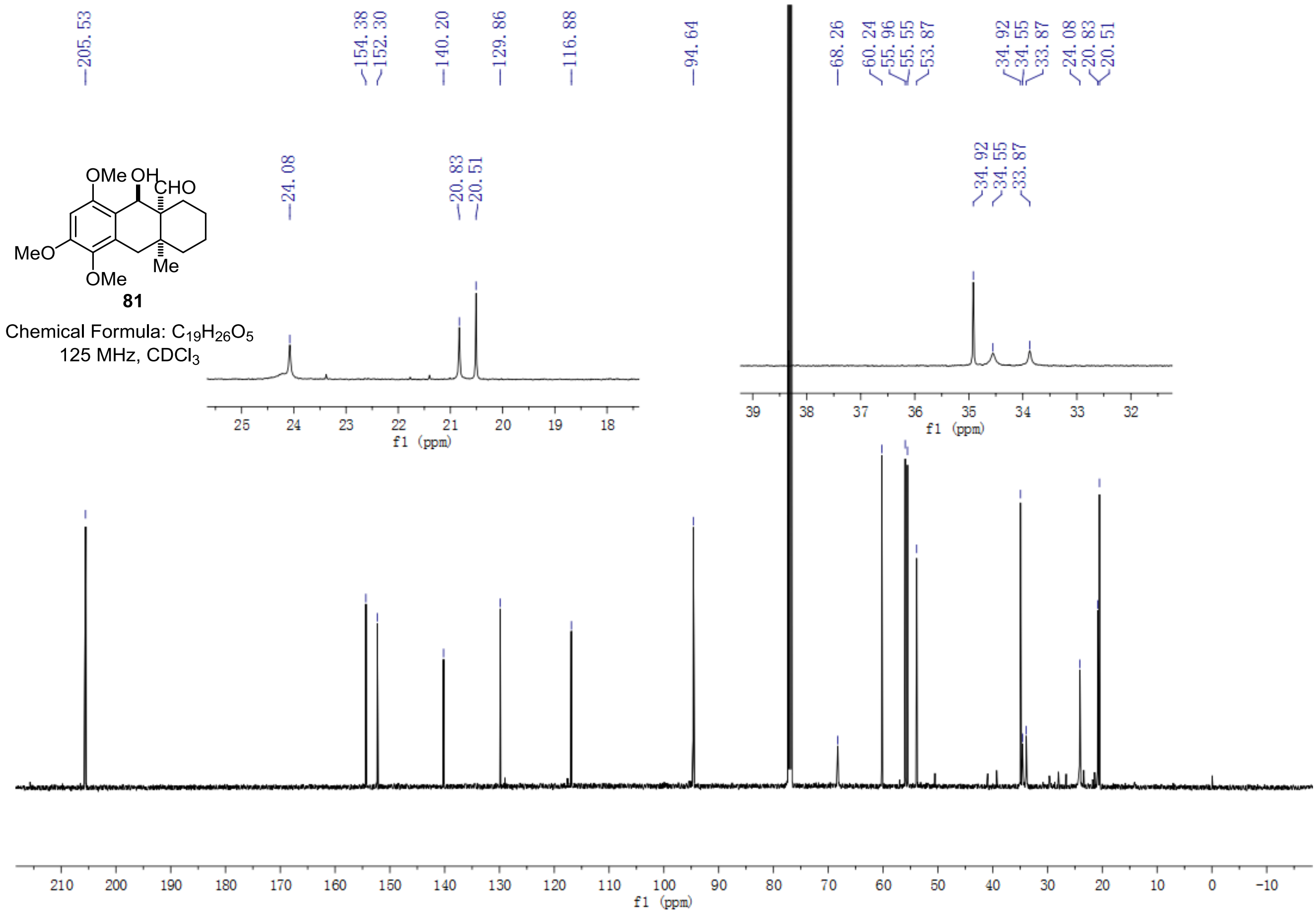

Supplementary Figure 66.[1]**H and** [13]**C NMR spectra for 81.**

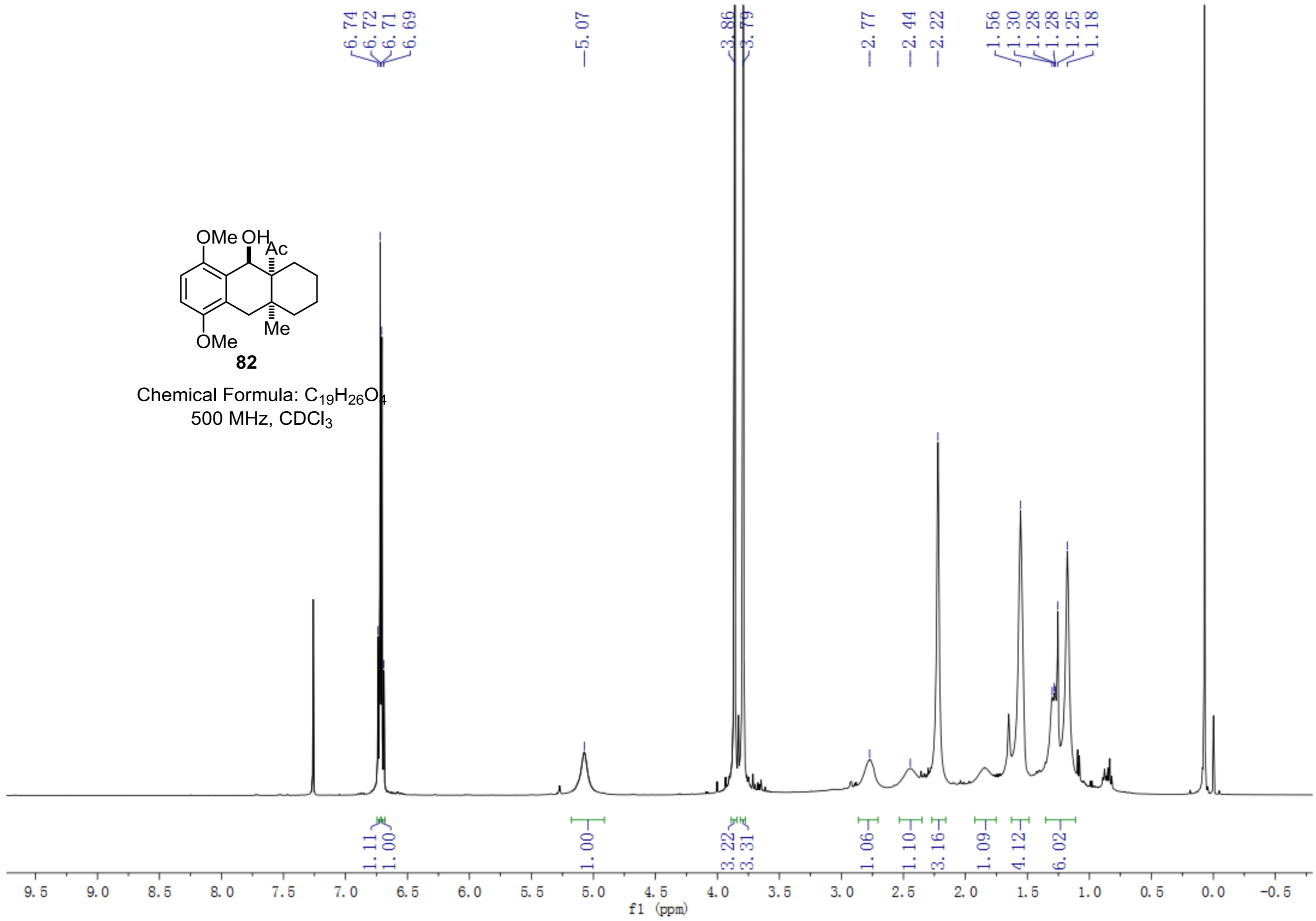

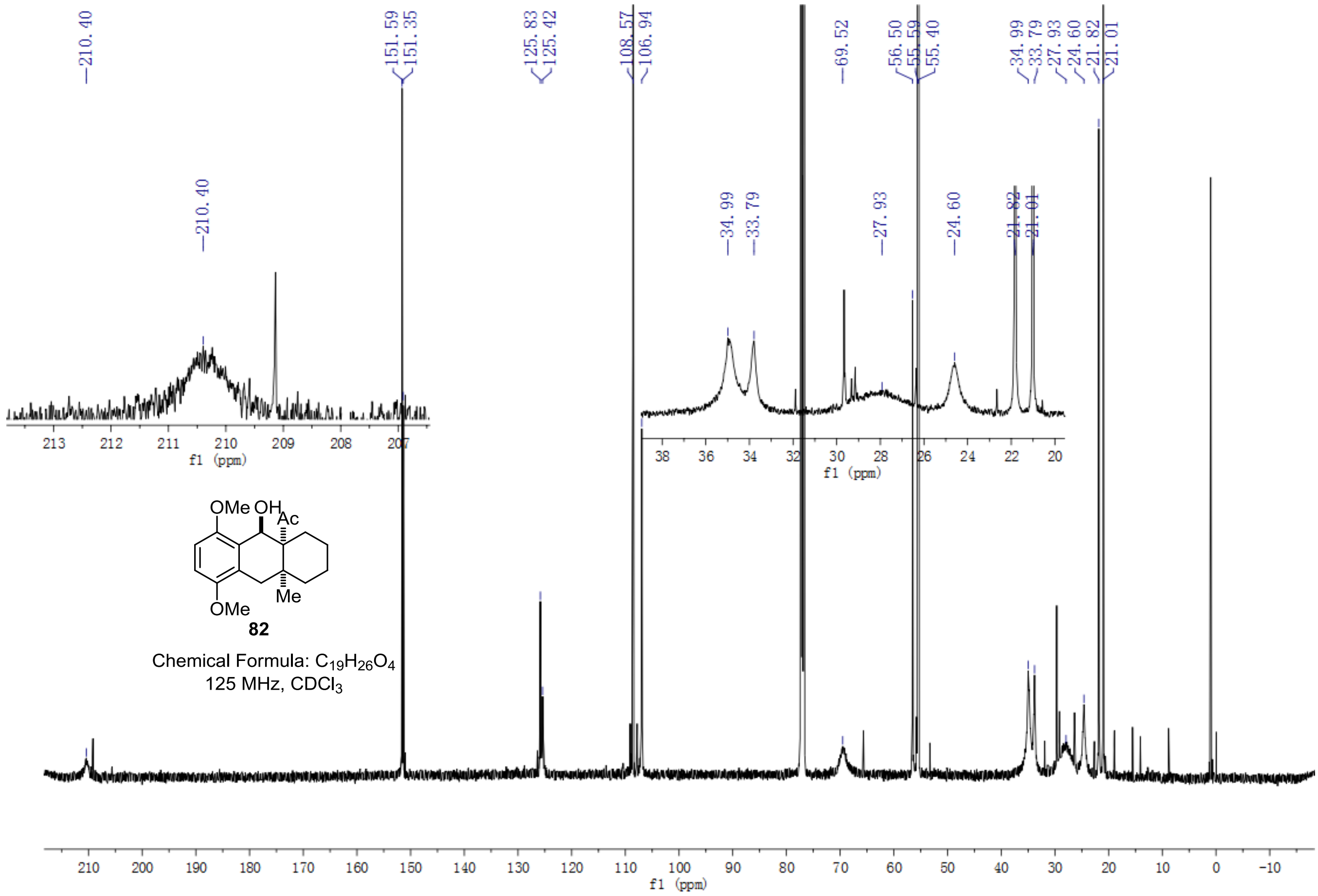

Supplementary Figure 67.^{1}H and ^{13}C NMR spectra for 82.

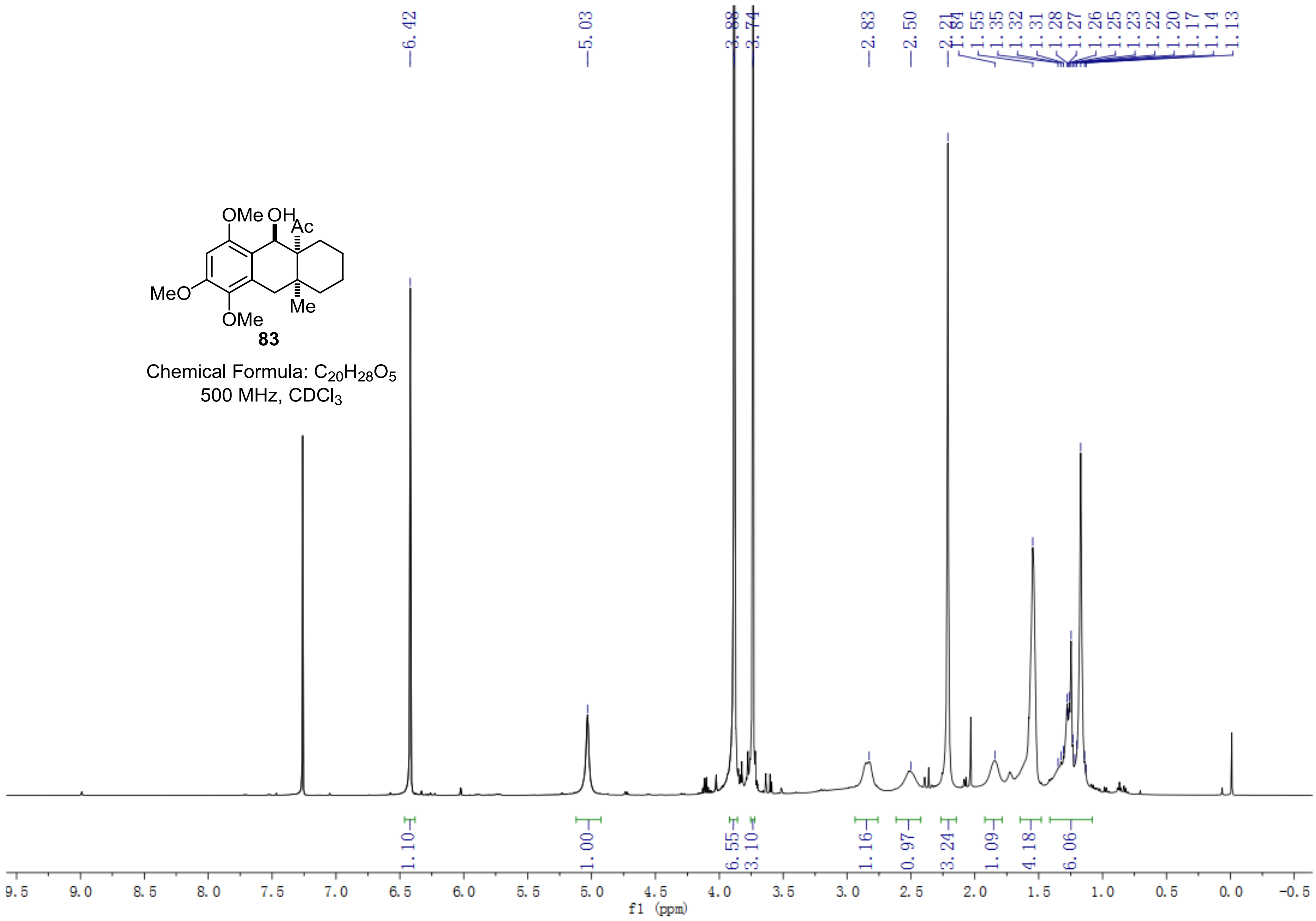

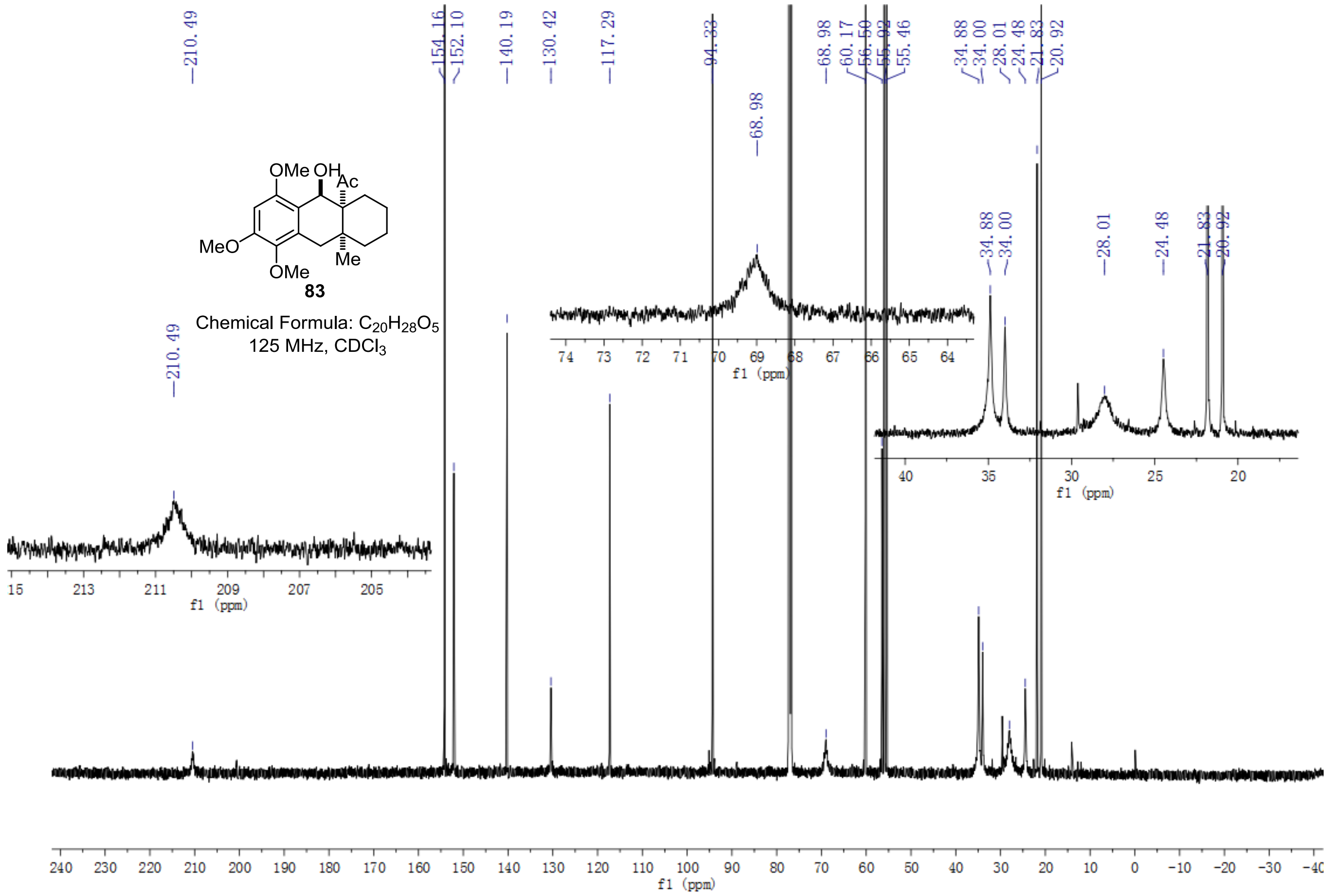

Supplementary Figure 68.^{1}H and ^{13}C NMR spectra for 83.

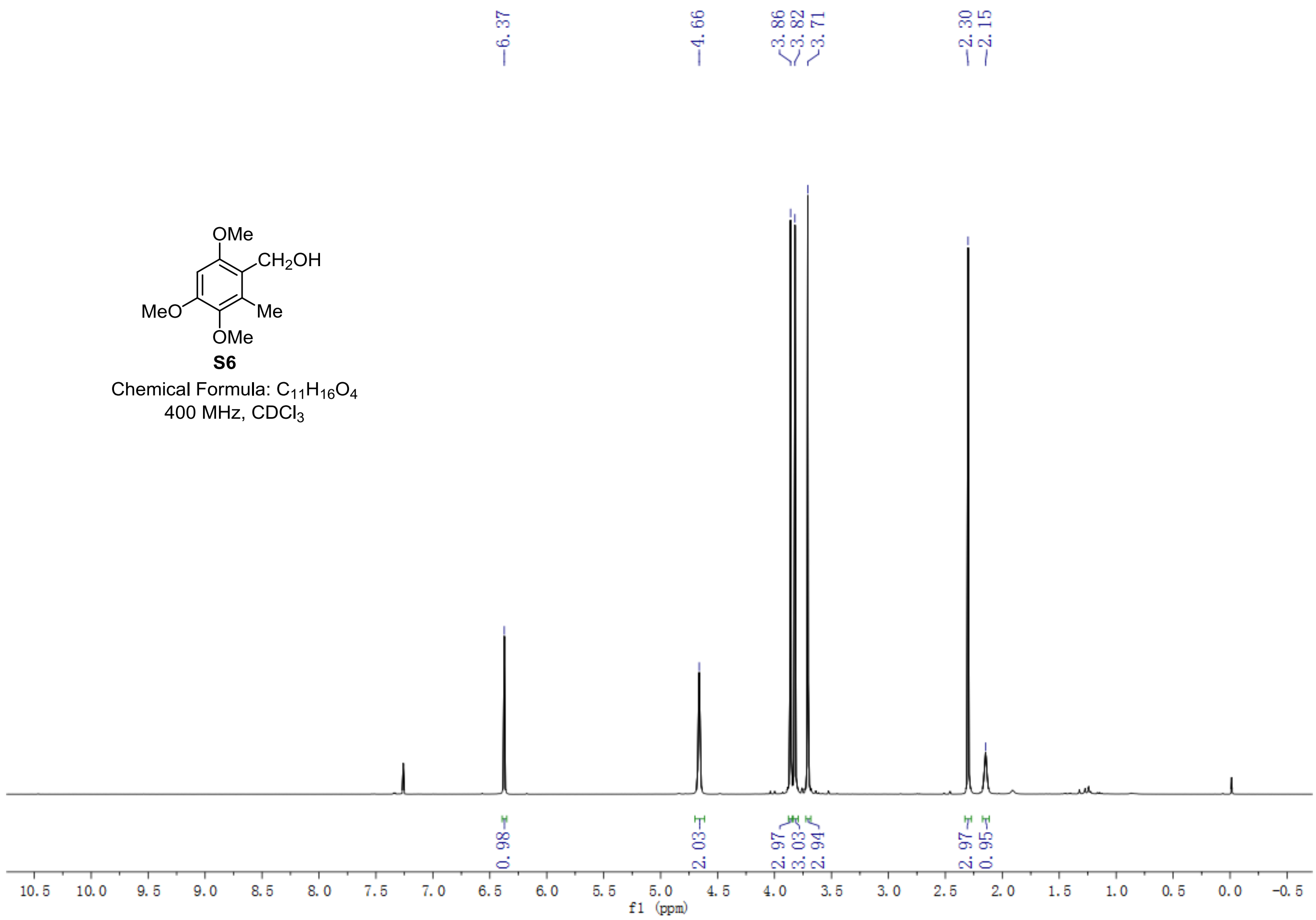

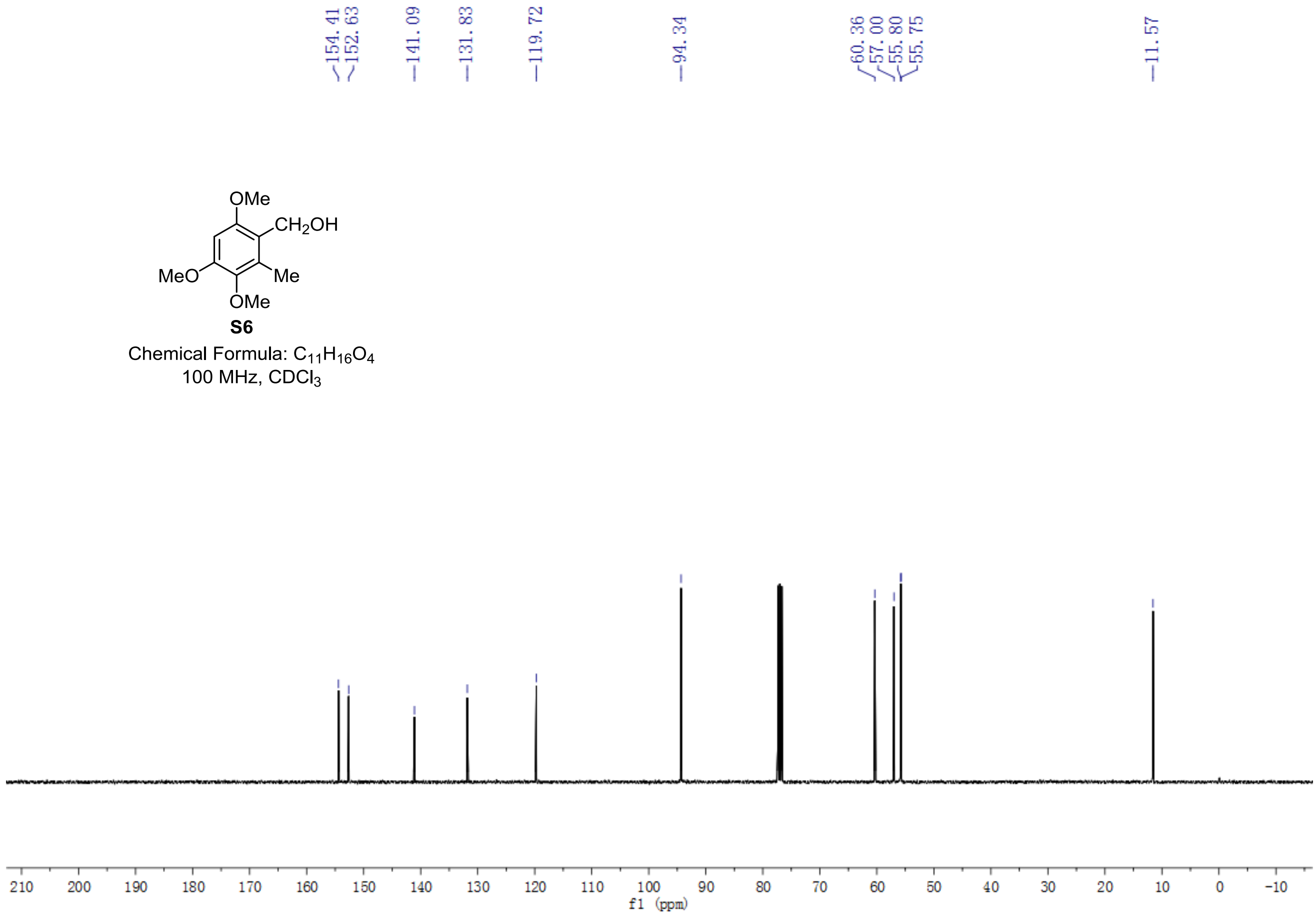

Supplementary Figure 69. ^{1}H **and** ^{13}C **NMR spectra for S6.**

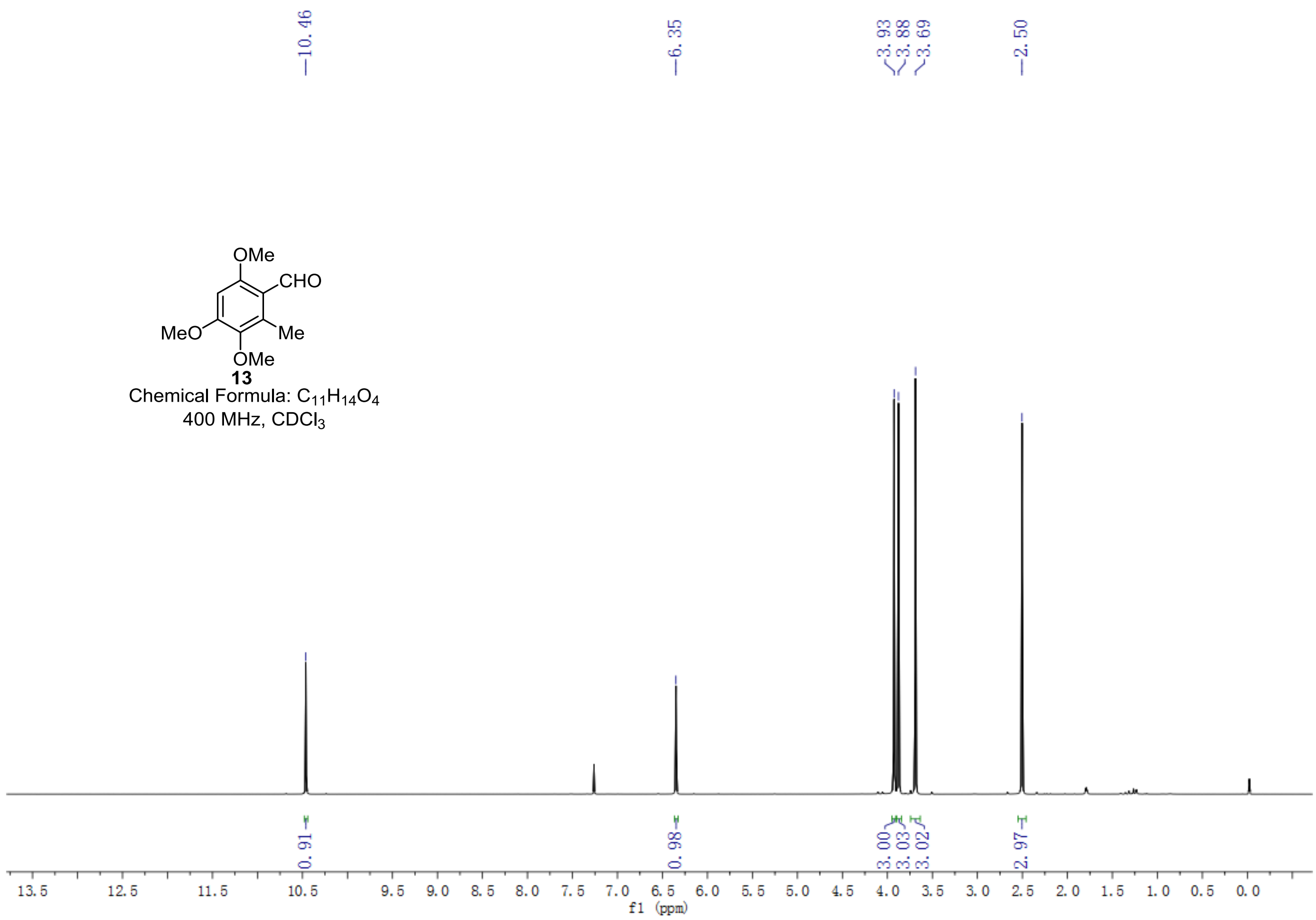

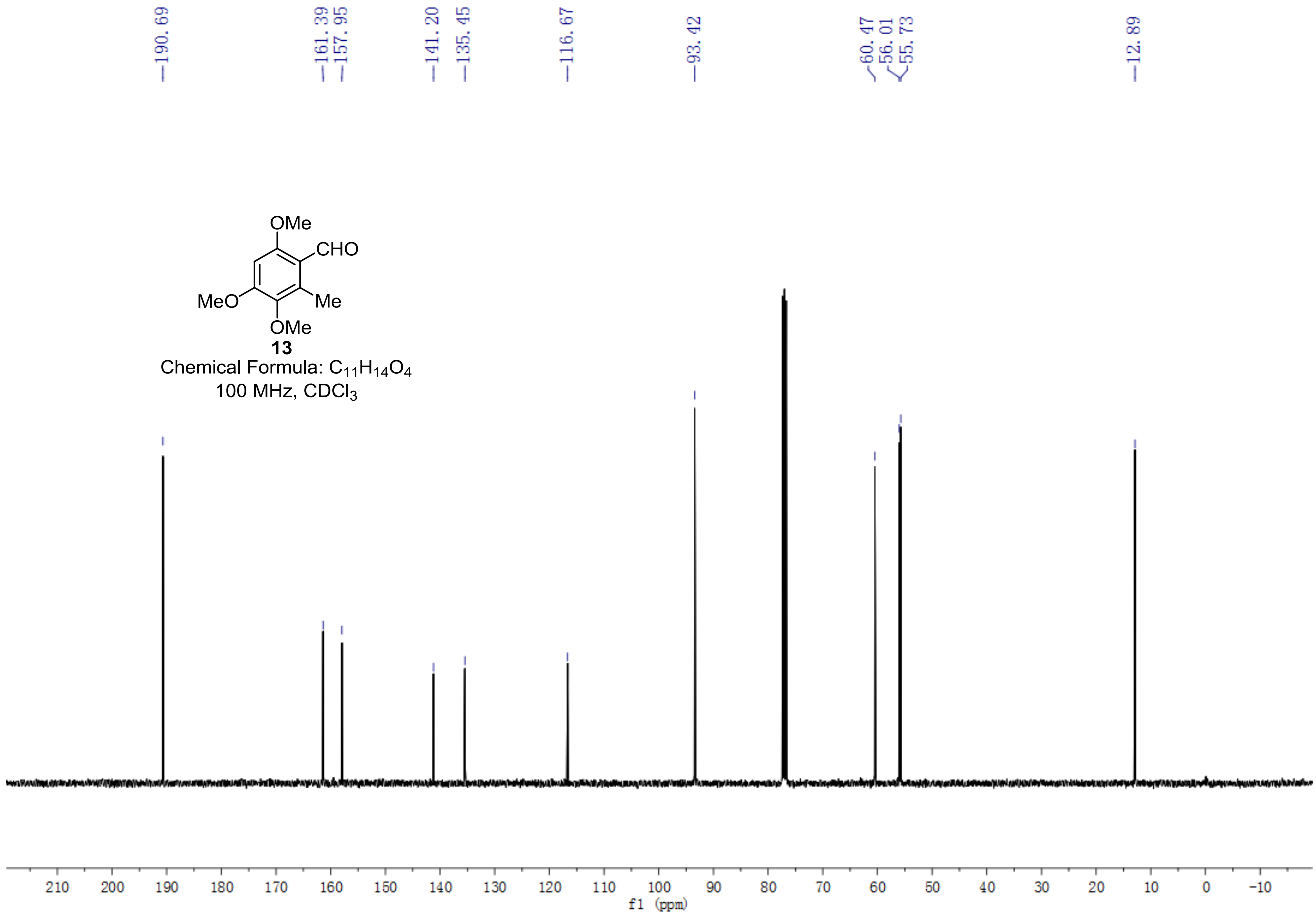

Supplementary Figure 70. ^{1}H **and** ^{13}C **NMR spectra for 13.**

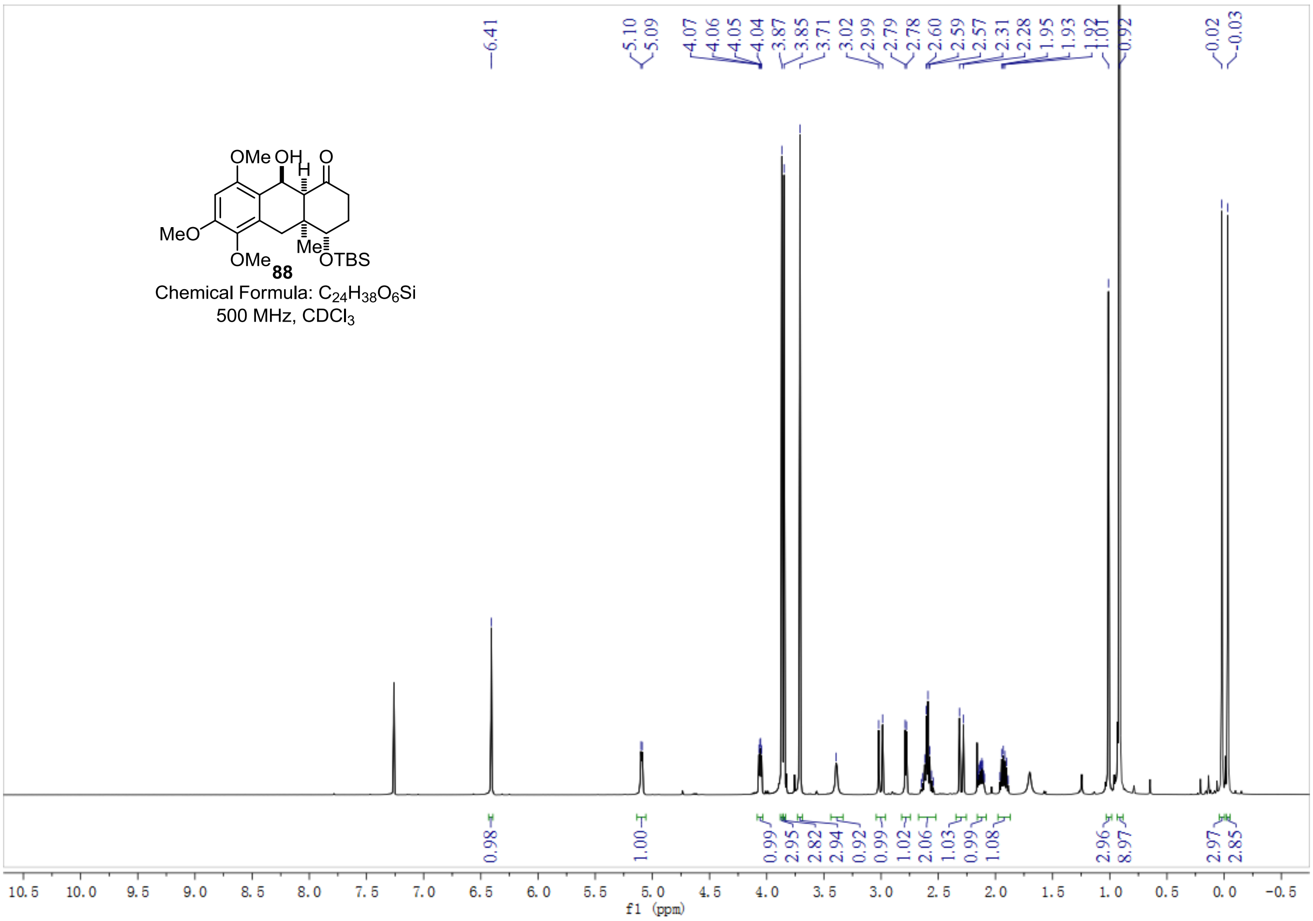

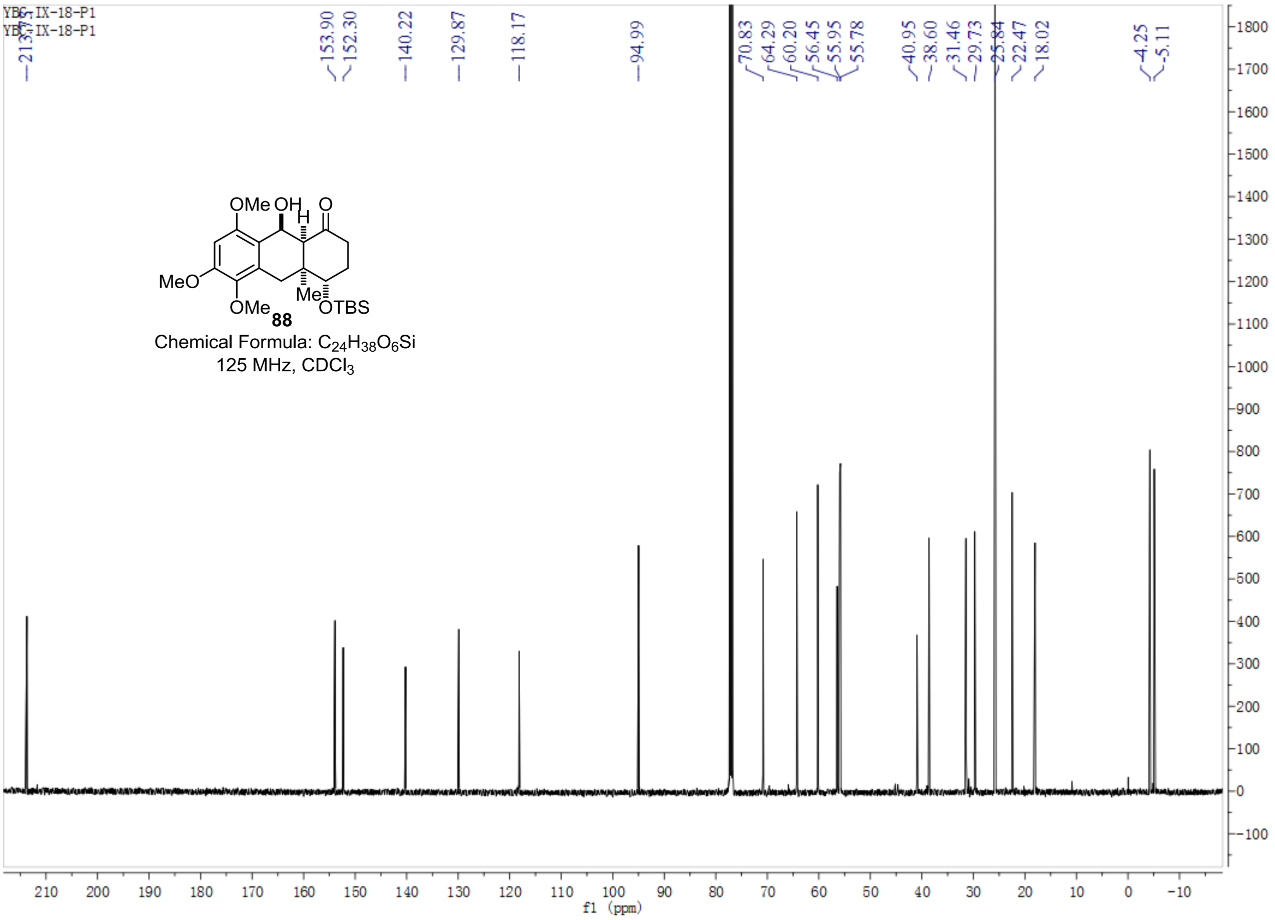

Supplementary Figure 71. ^{1}H and ^{13}C NMR spectra for 88.

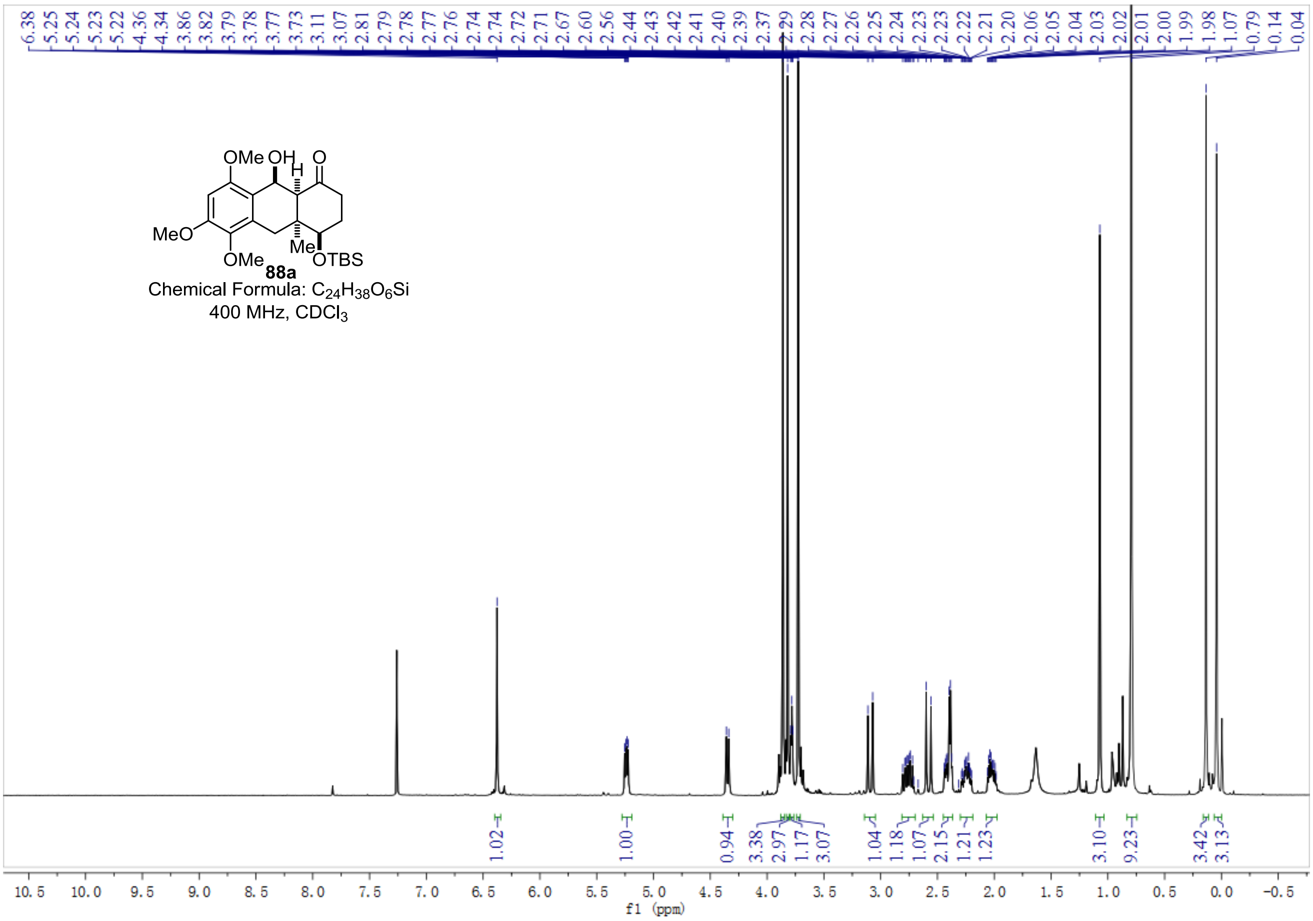

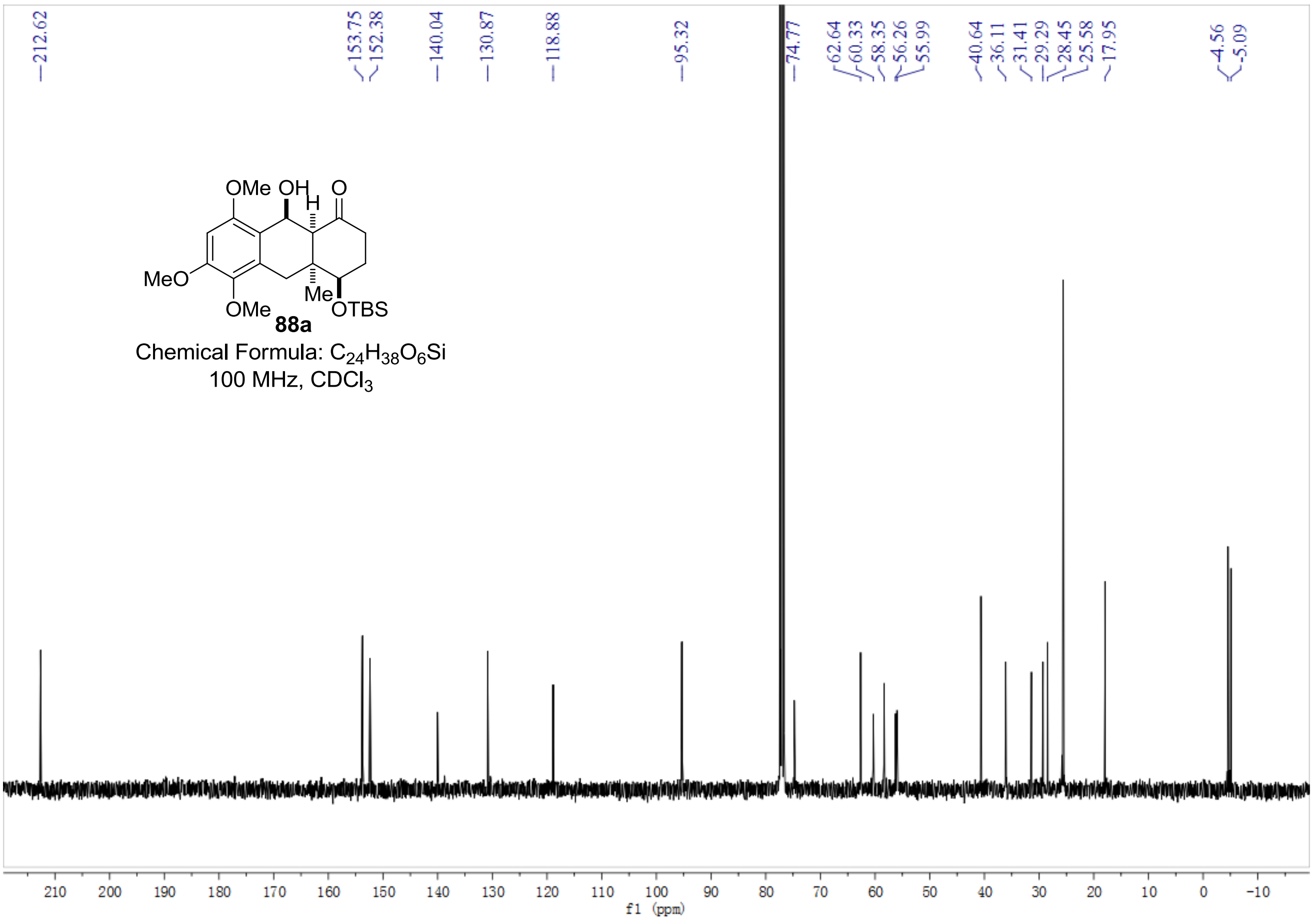

Supplementary Figure 72. ^{1}H **and** ^{13}C **NMR spectra for 88a.**

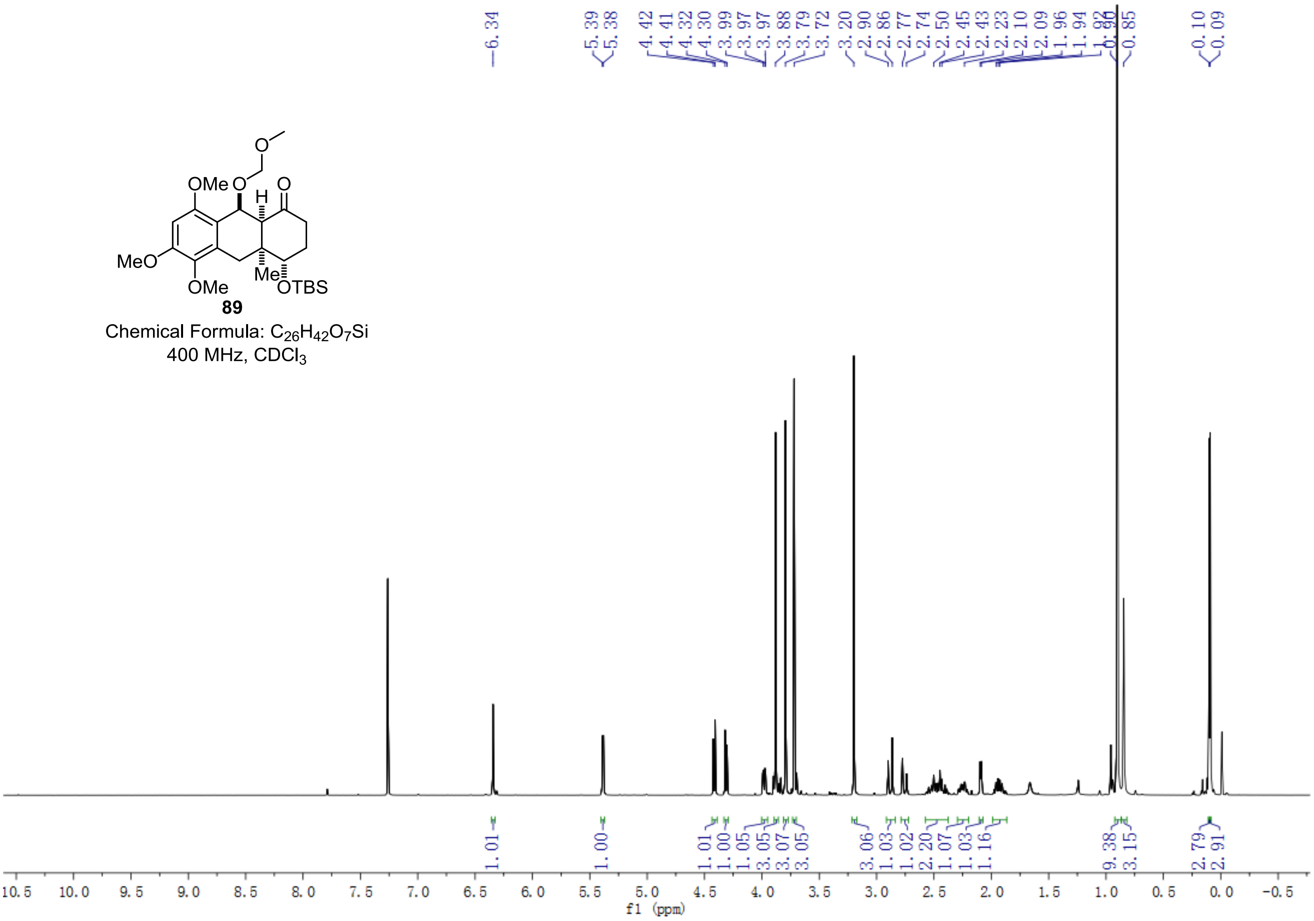

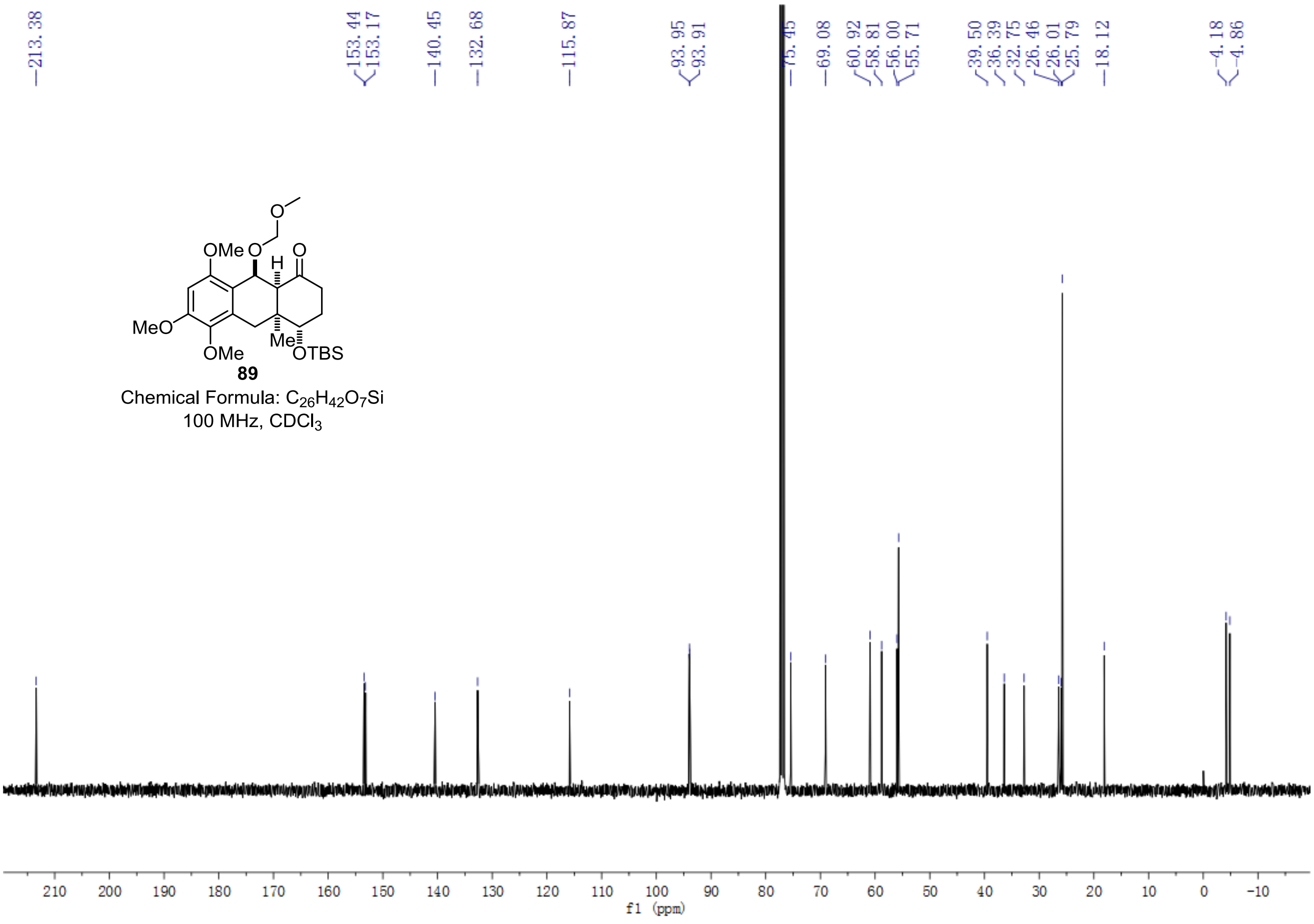

Supplementary Figure 73. ^{1}H and ^{13}C NMR spectra for 89.

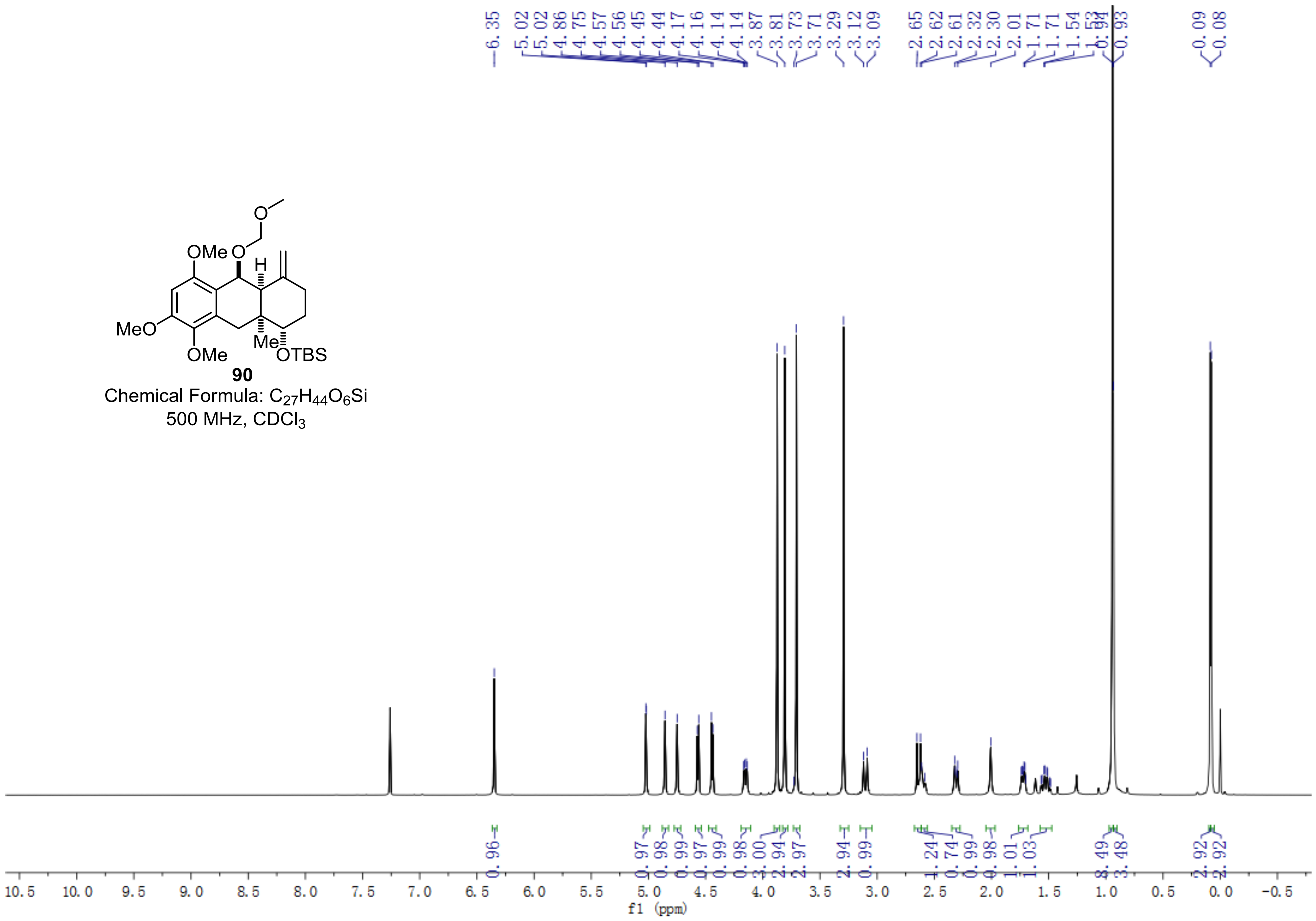

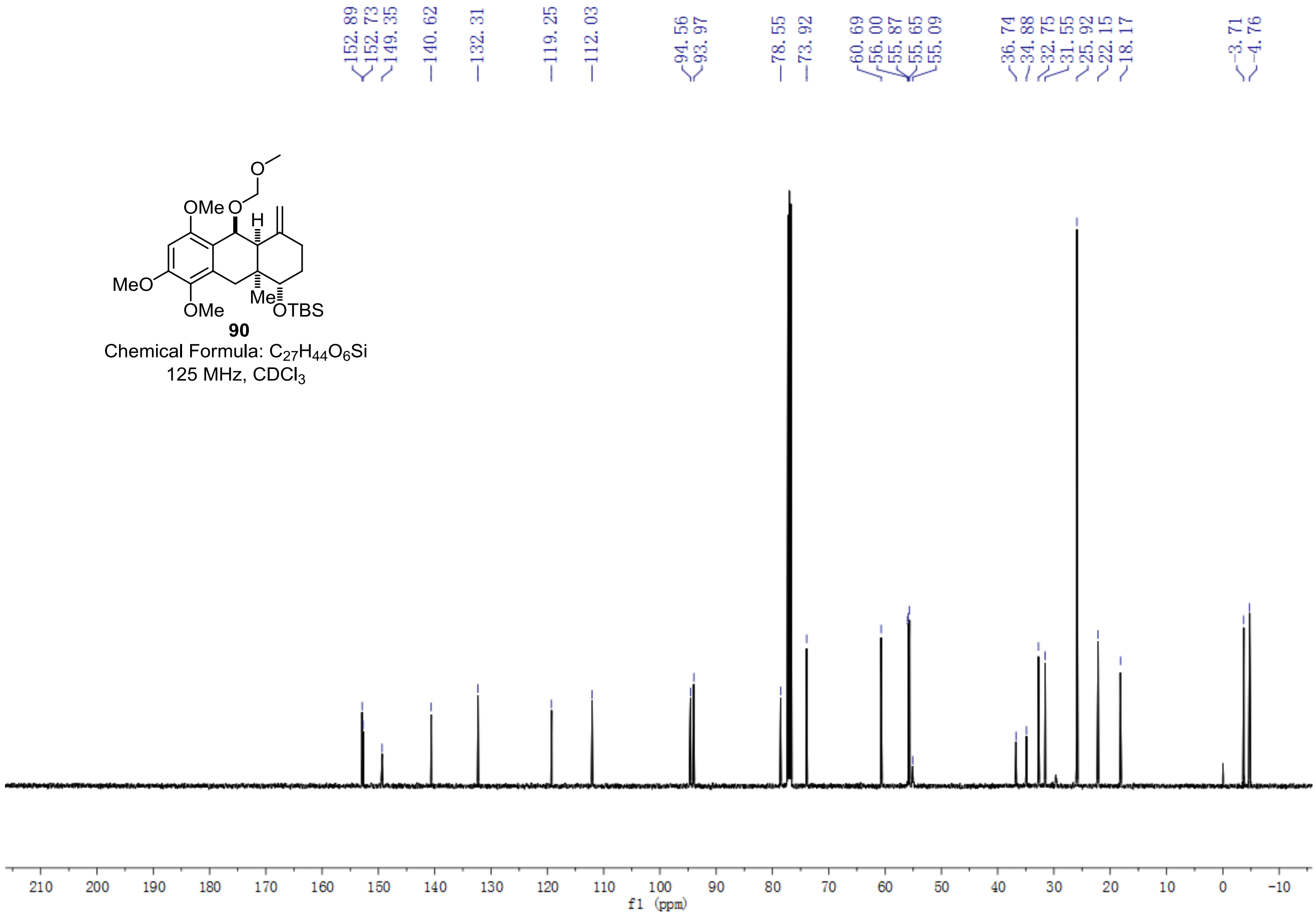

Supplementary Figure 74.^{1}H and ^{13}C NMR spectra for 90.

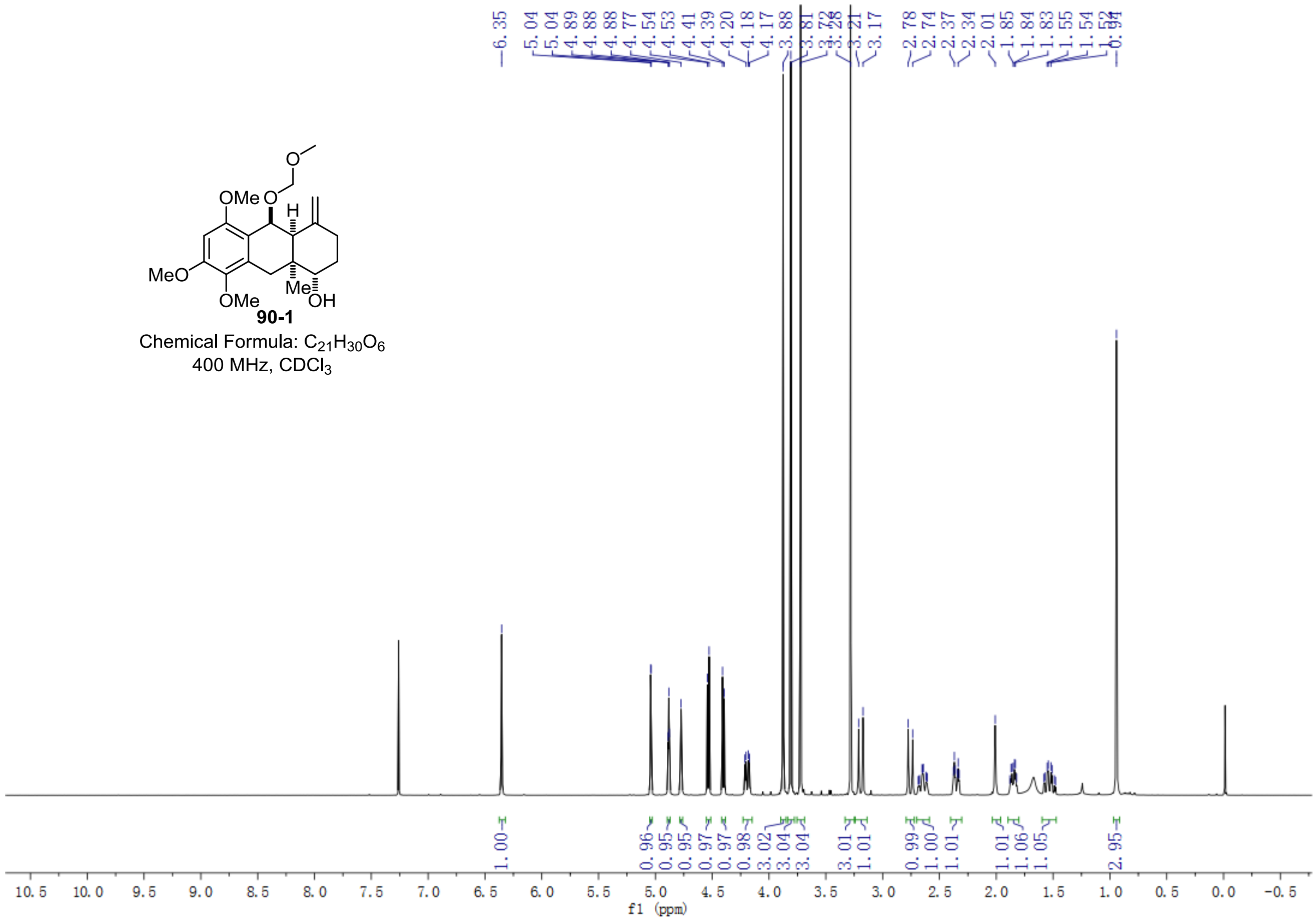

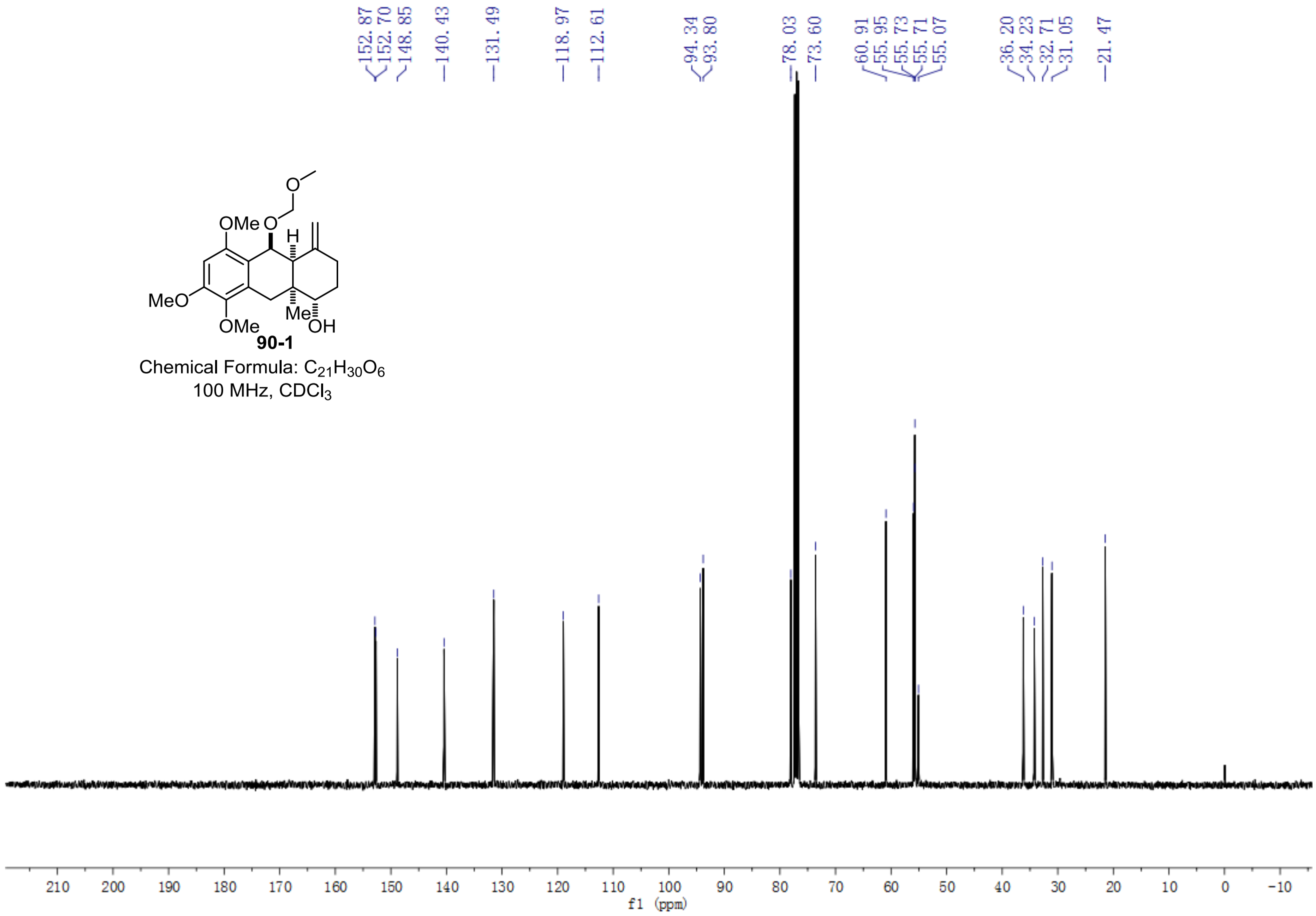

Supplementary Figure 75.^{1}H and ^{13}C NMR spectra for 90-1.

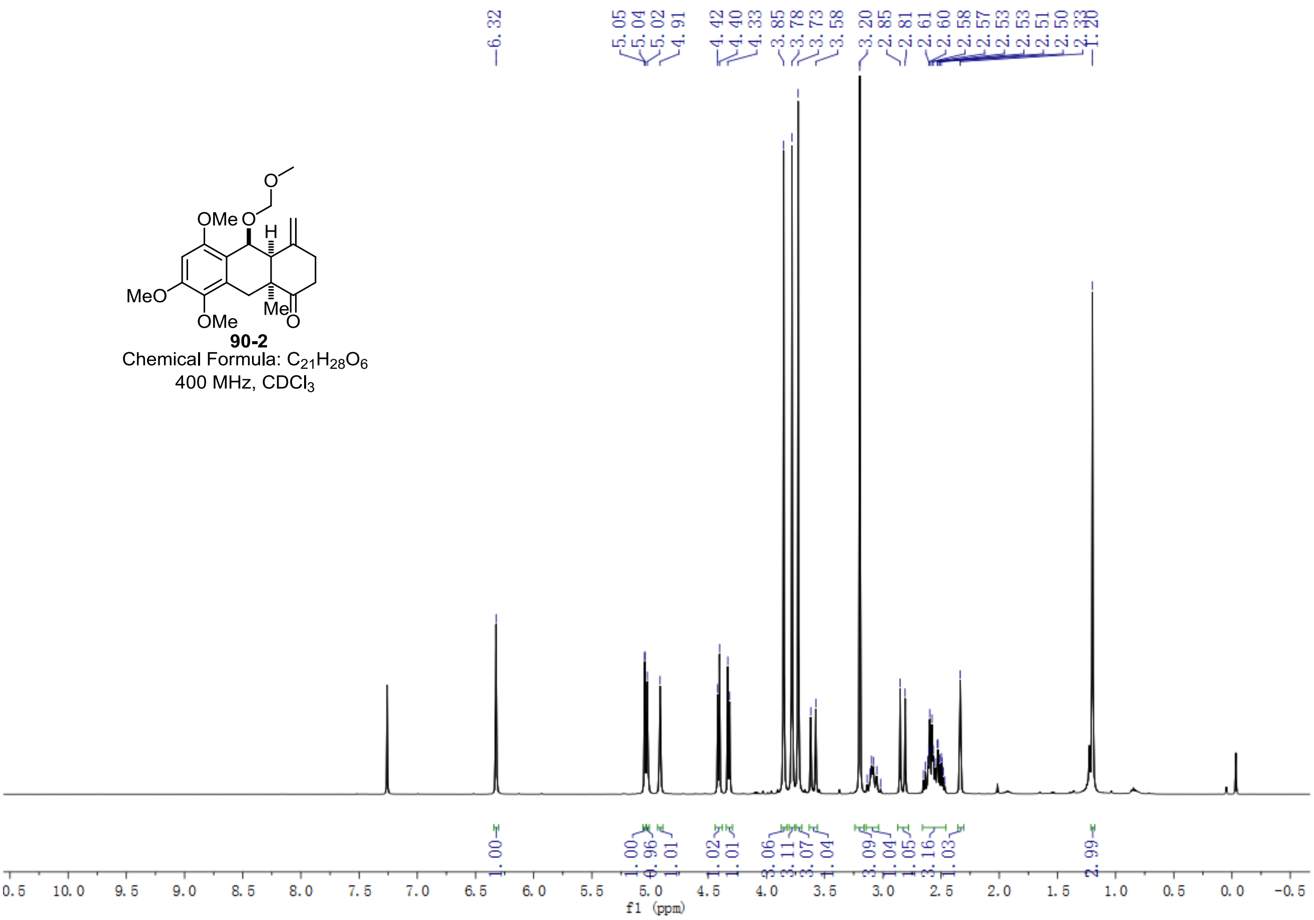

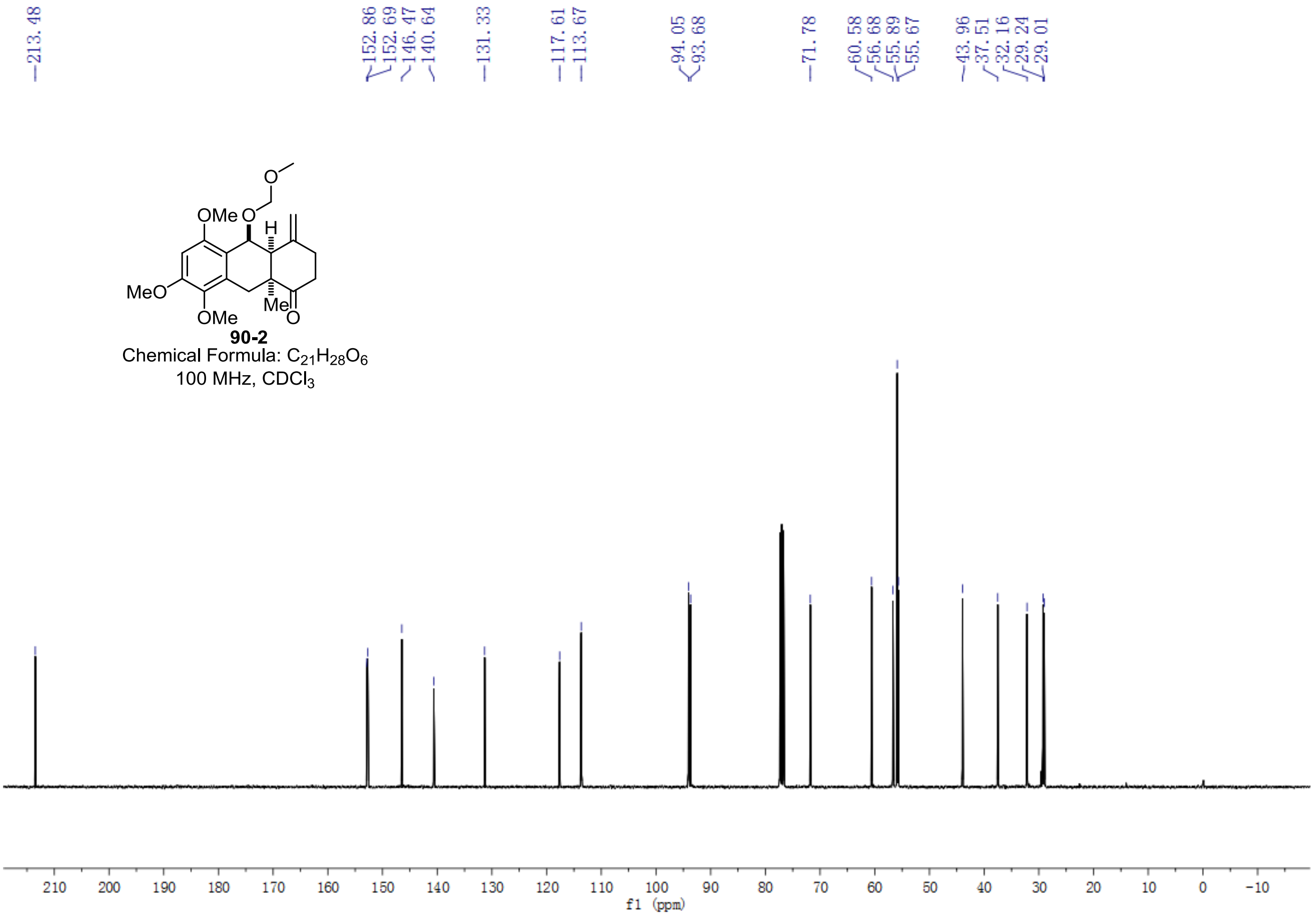

Supplementary Figure 76.^{1}H and ^{13}C NMR spectra for 90-2.

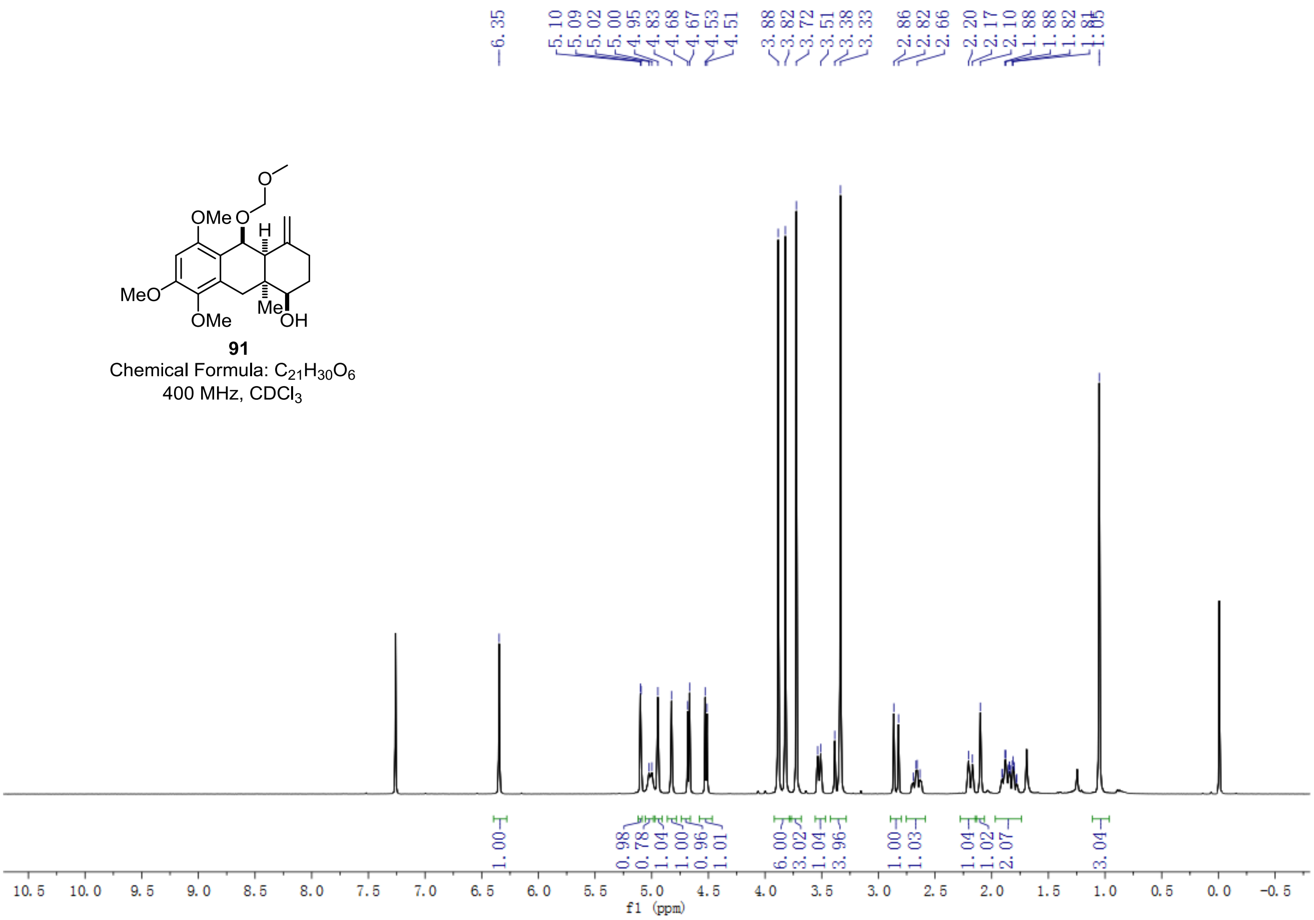

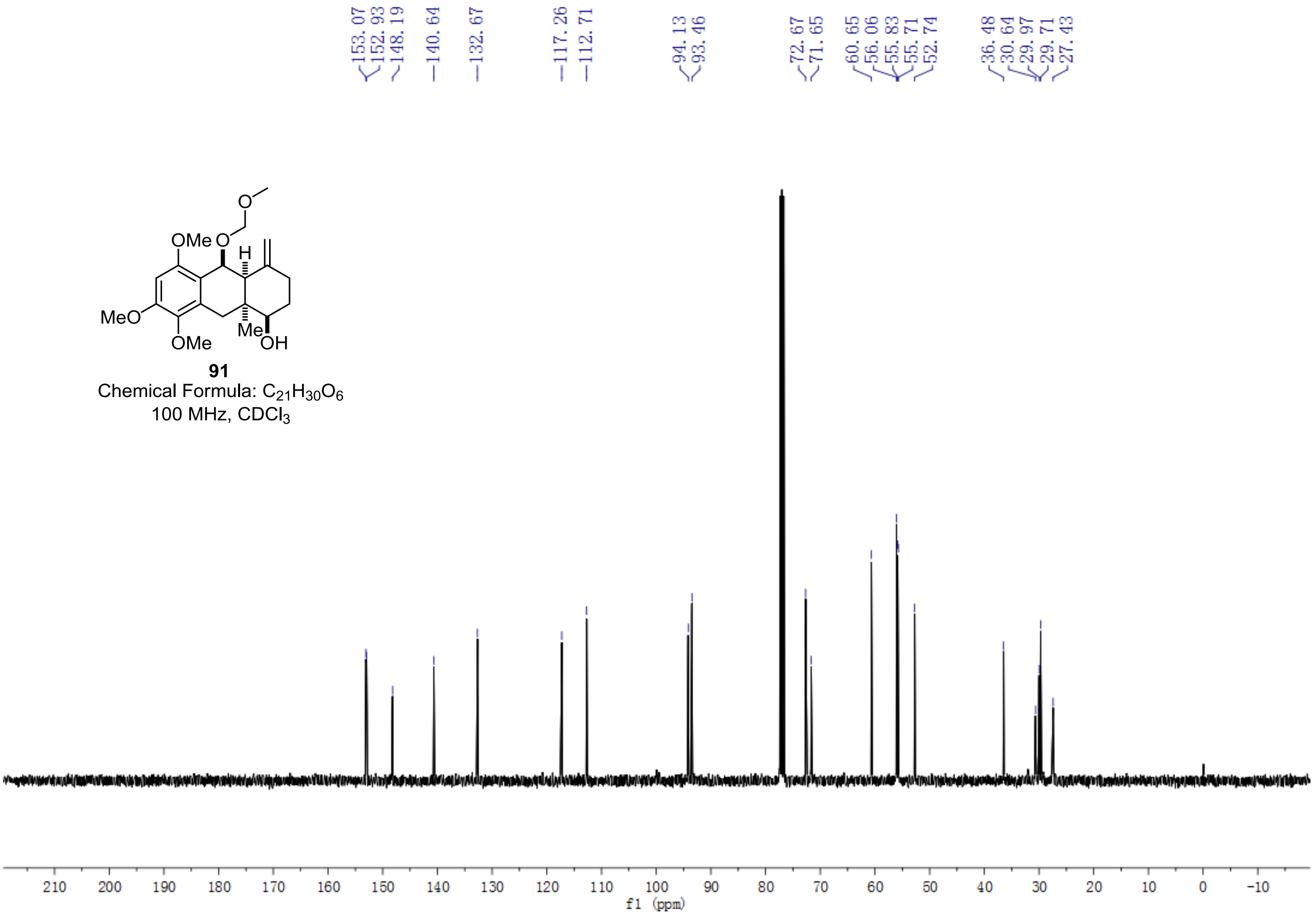

Supplementary Figure 77.[1]**H and** [13]**C NMR spectra for 91.**

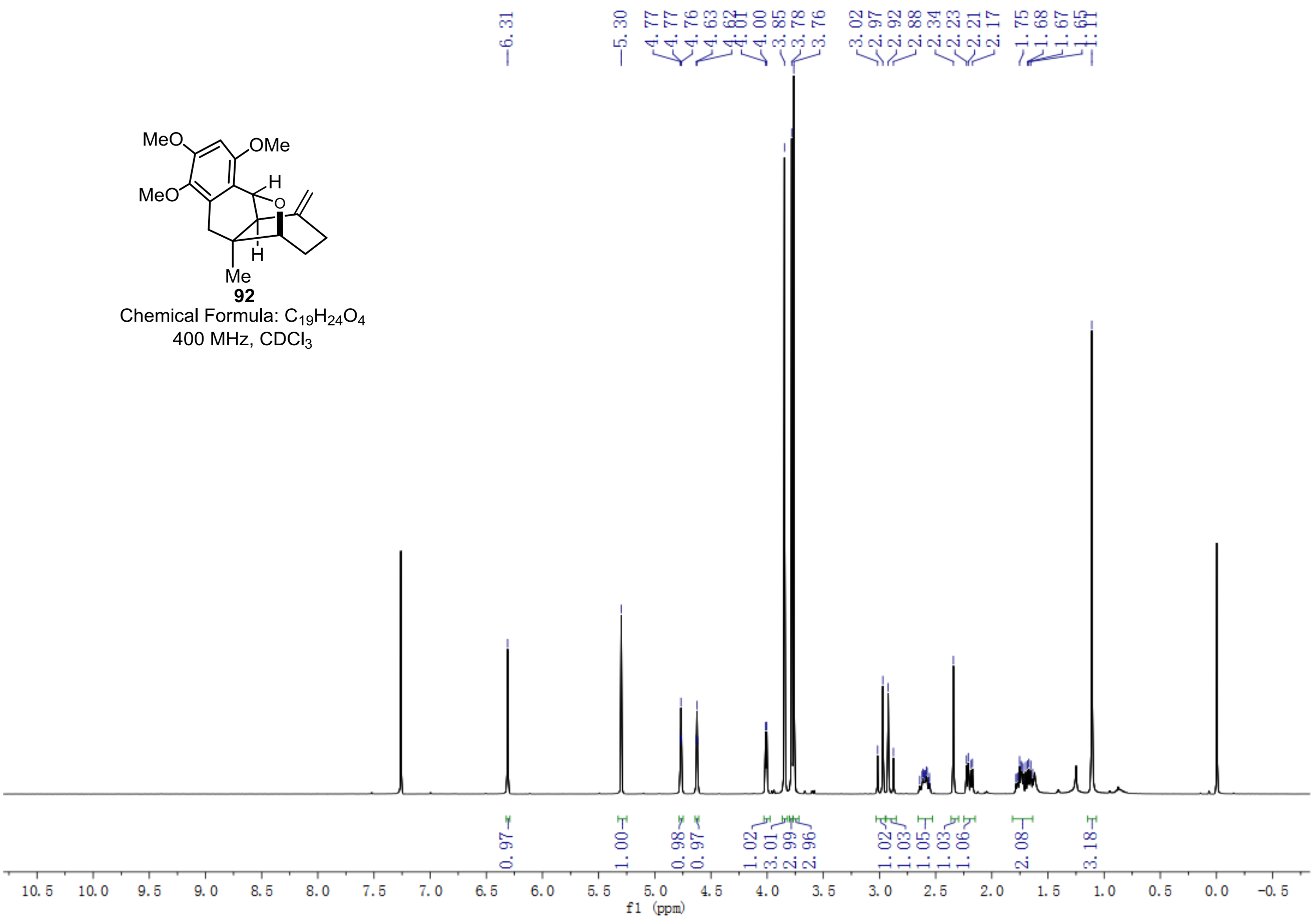

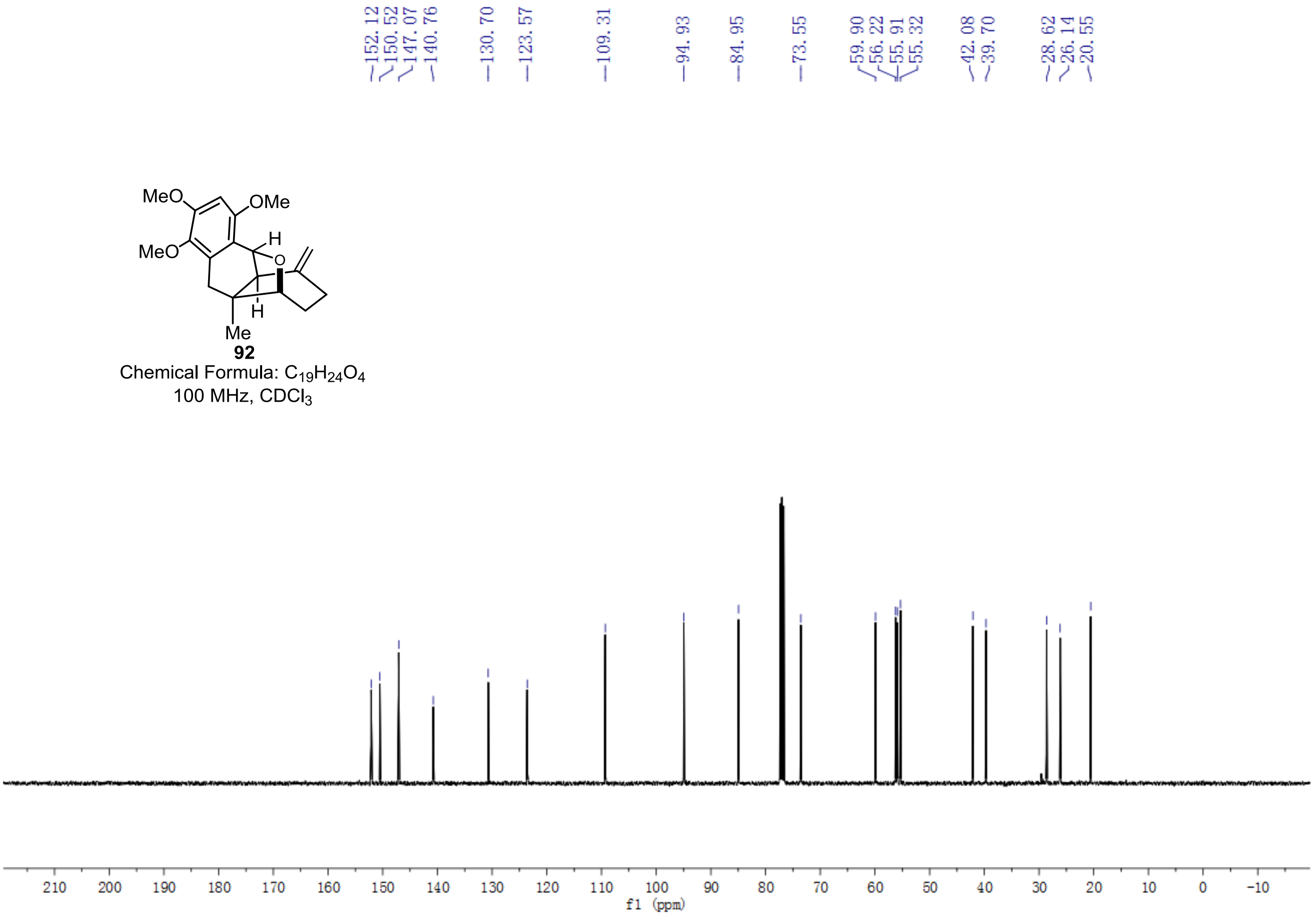

Supplementary Figure 78.^{1}H and ^{13}C NMR spectra for 92.

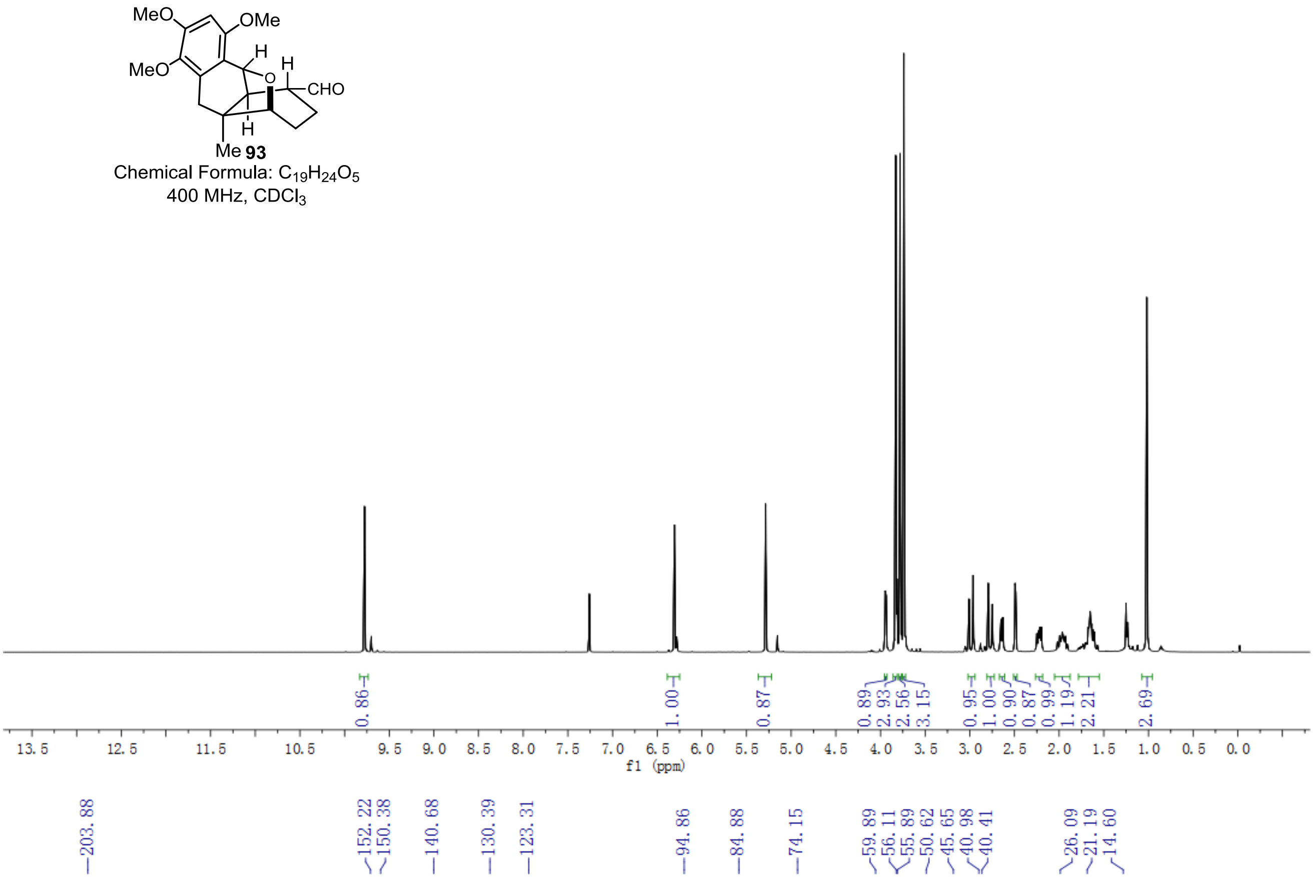

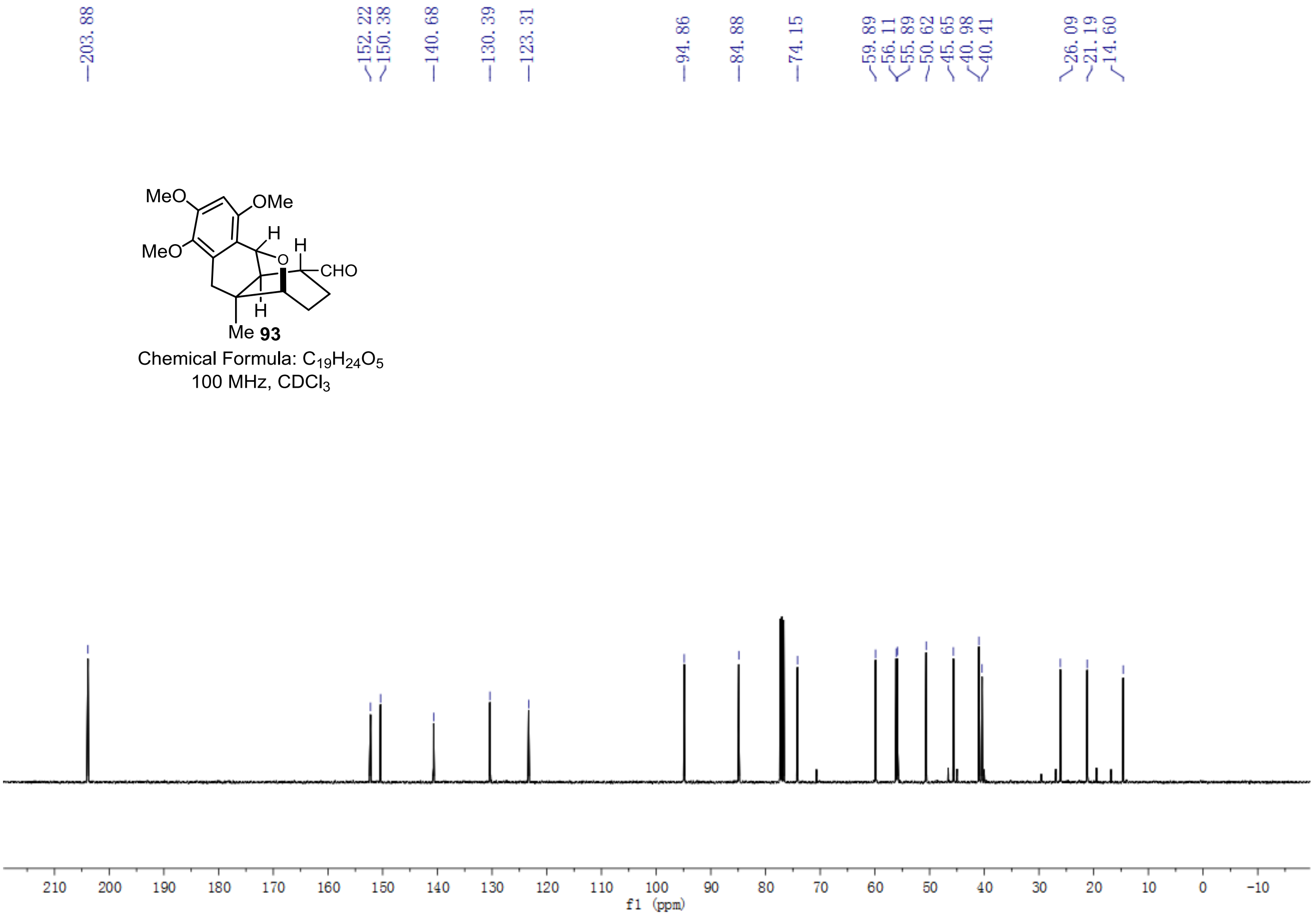

Supplementary Figure 79. ^{1}**H and** ^{13}**C NMR spectra for 93.**

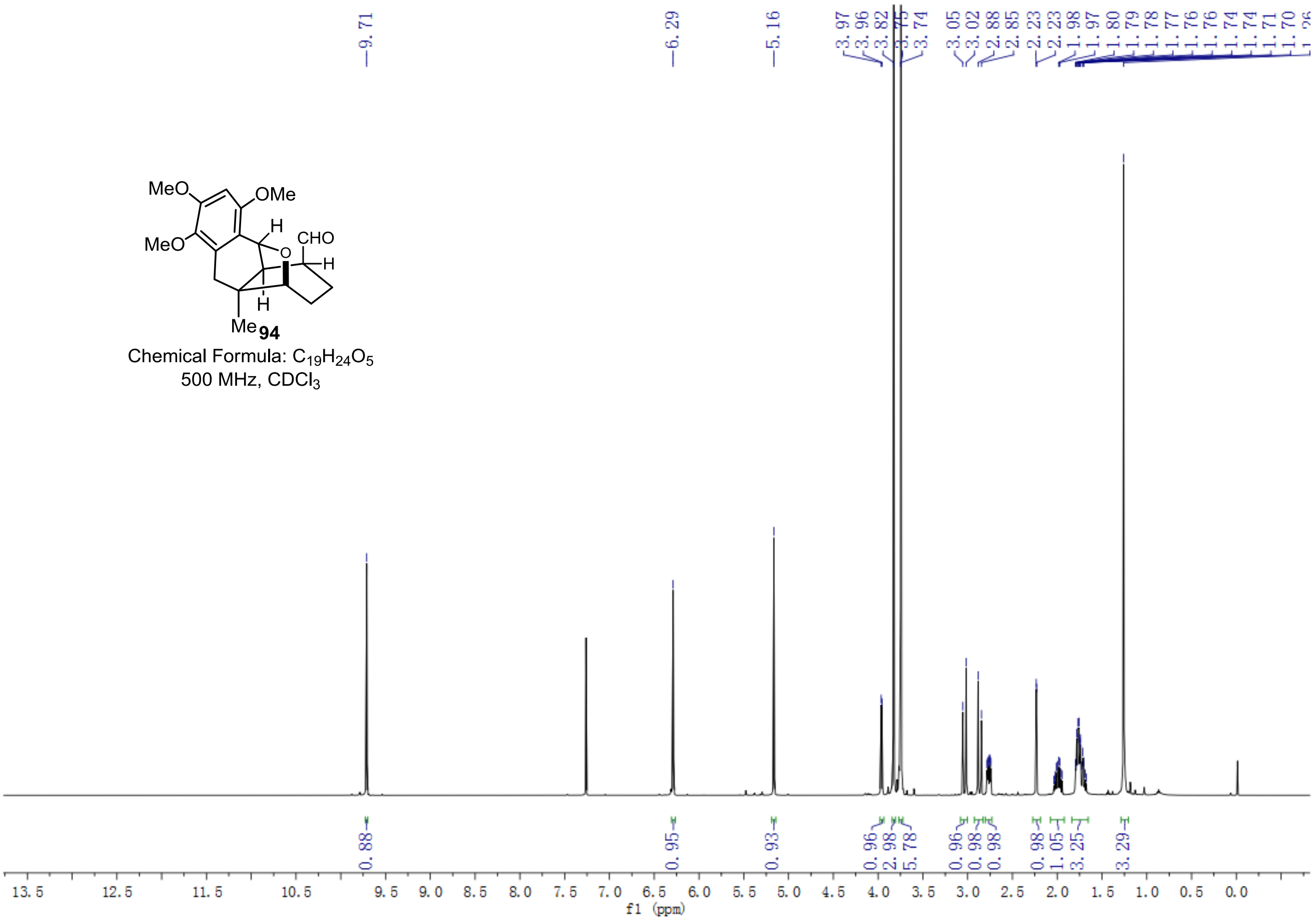

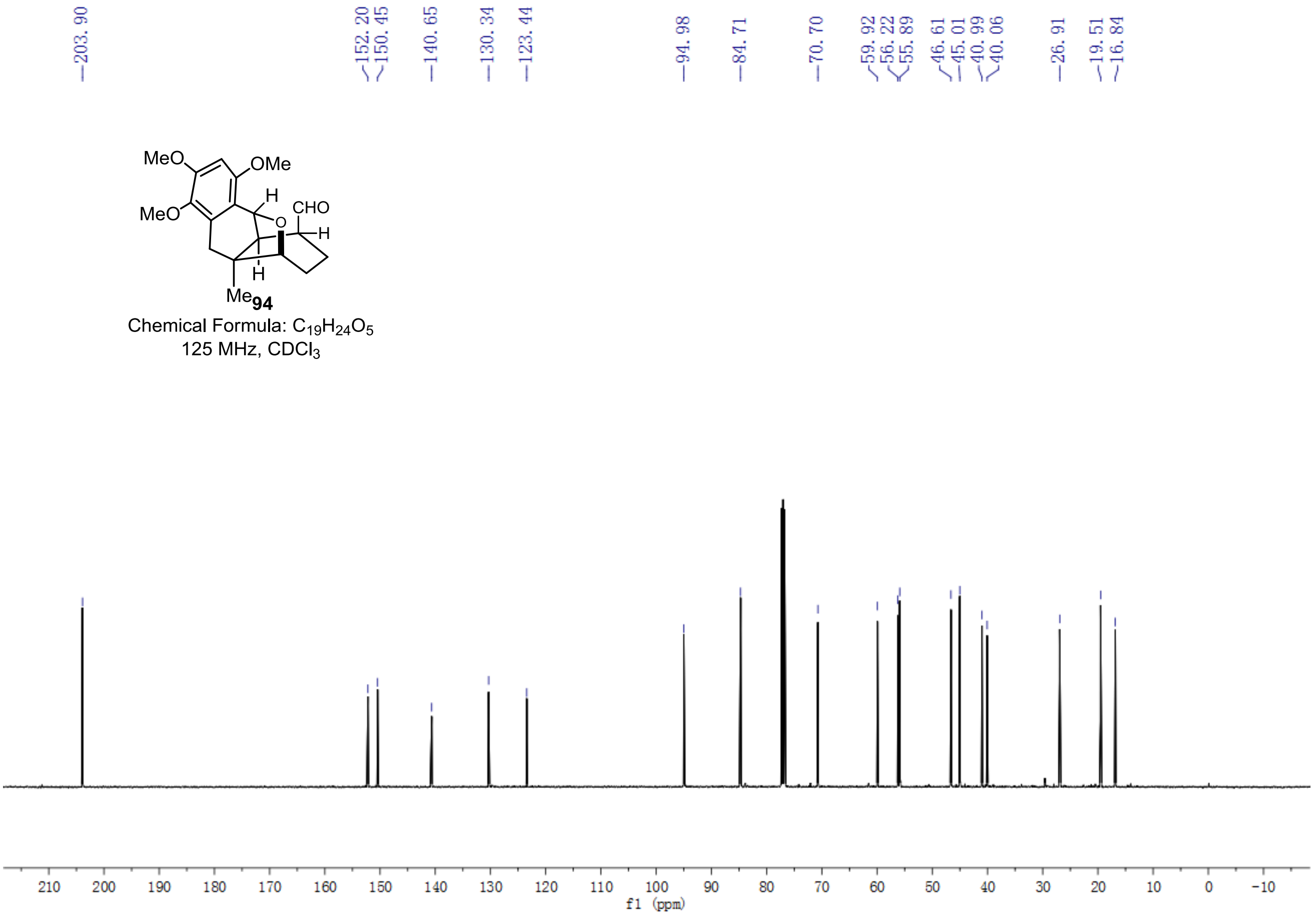

Supplementary Figure 80. ^{1}H **and** ^{13}C **NMR spectra for 94.**

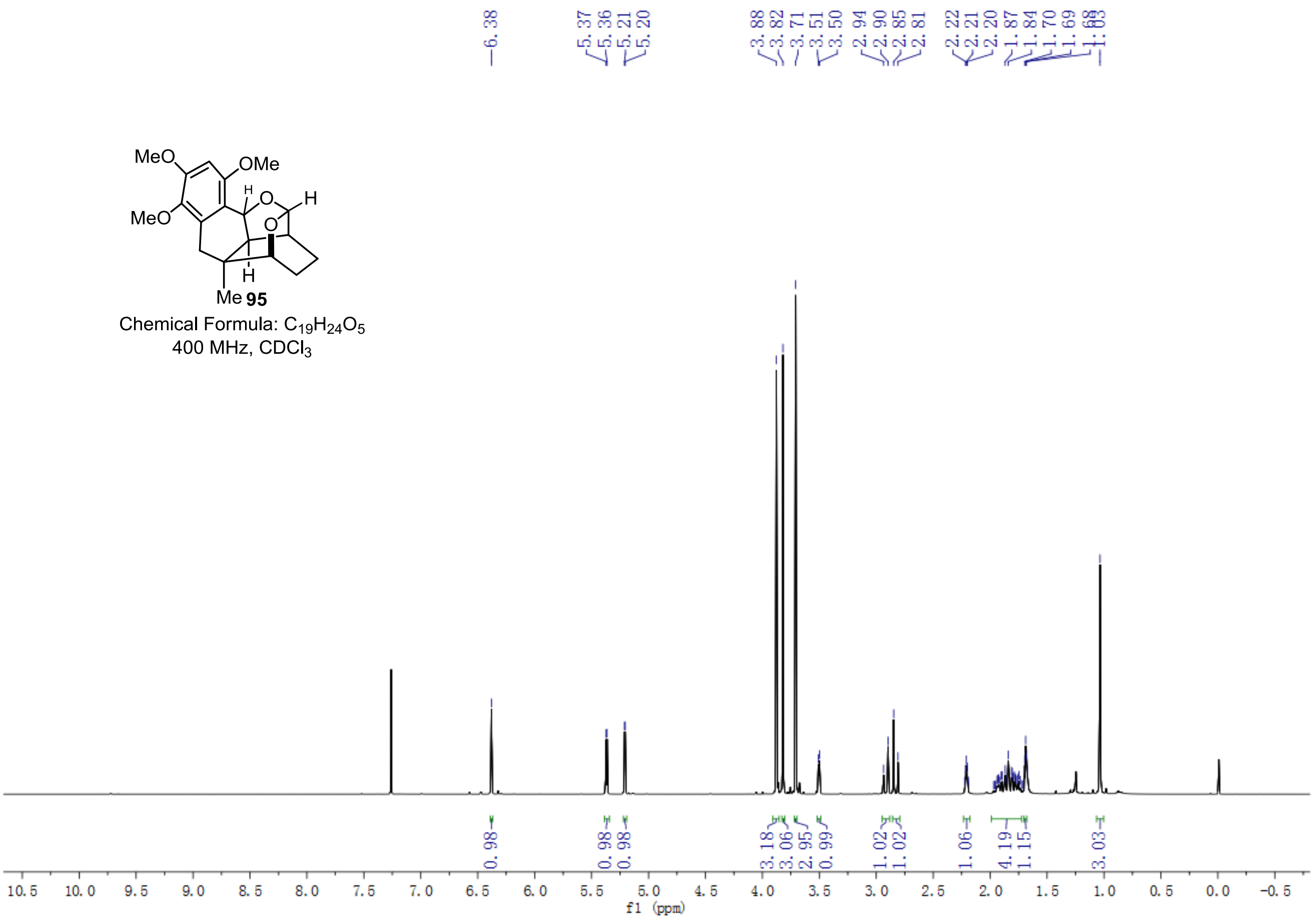

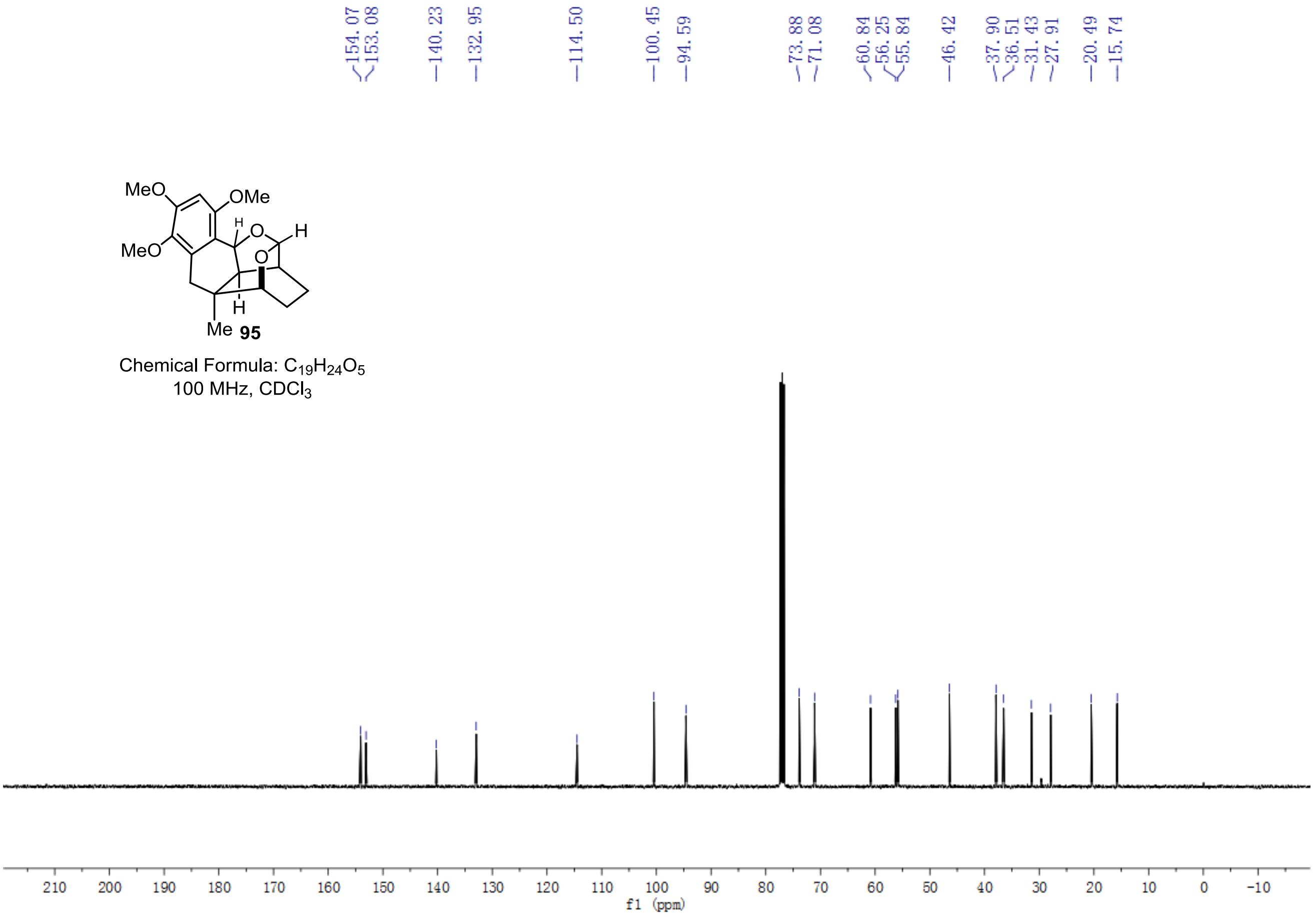

Supplementary Figure 81.^{1}H and ^{13}C NMR spectra for 95.

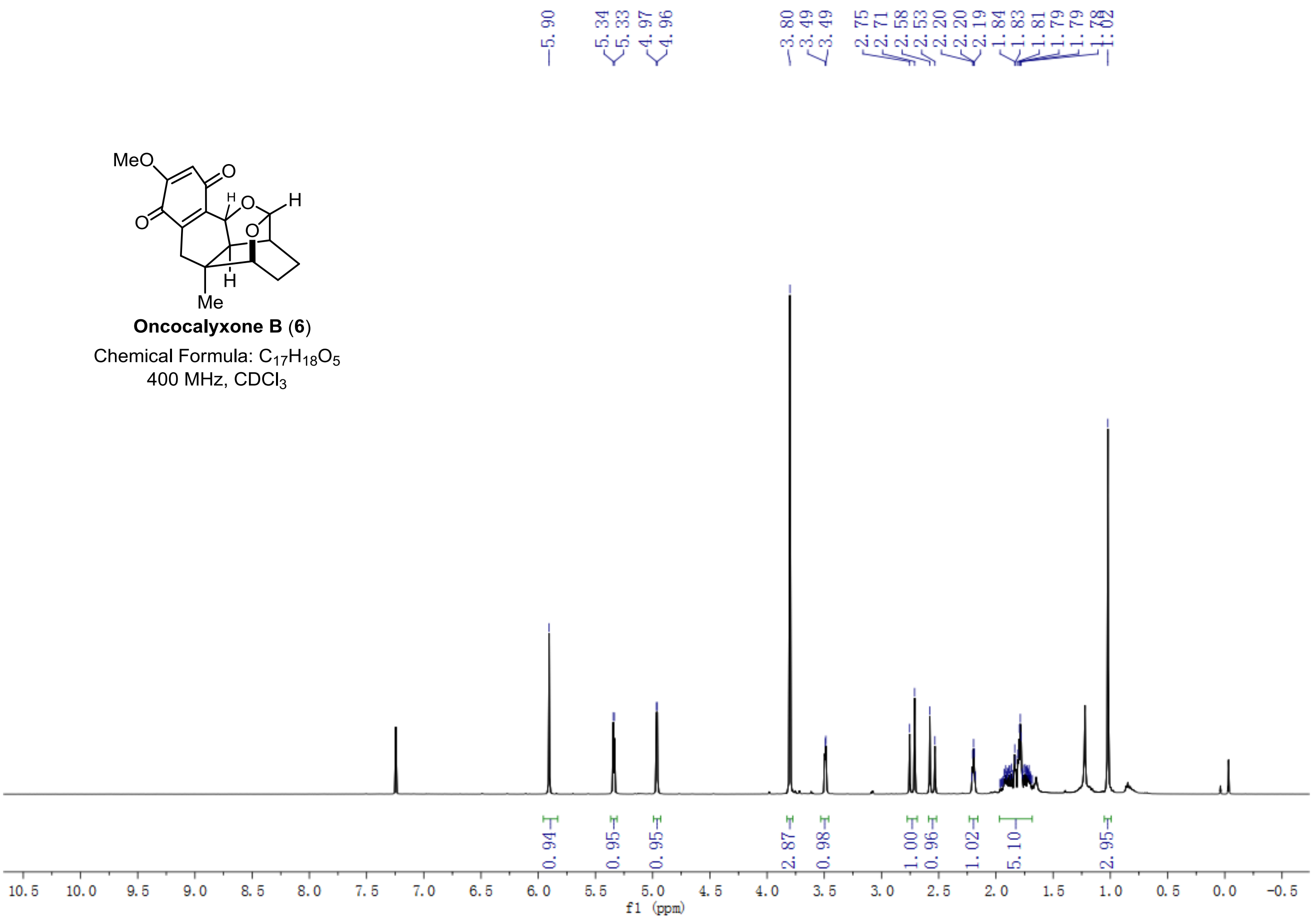

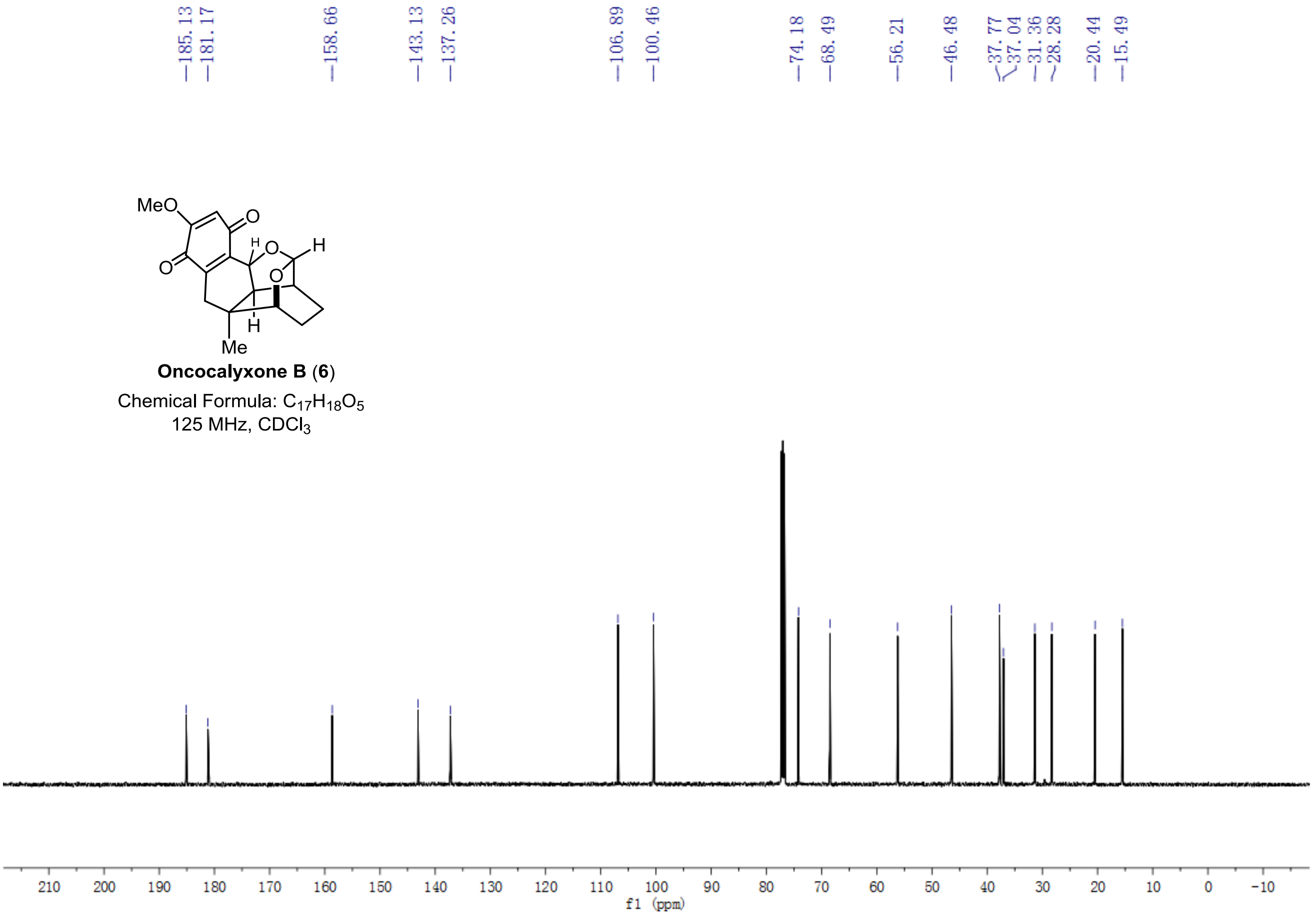

Supplementary Figure 82. ^{1}**H and** ^{13}**C NMR spectra for Oncocalyxone B (6).**

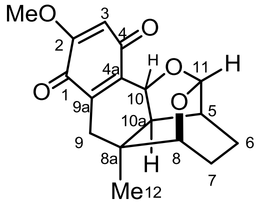

Oncocalyxone B

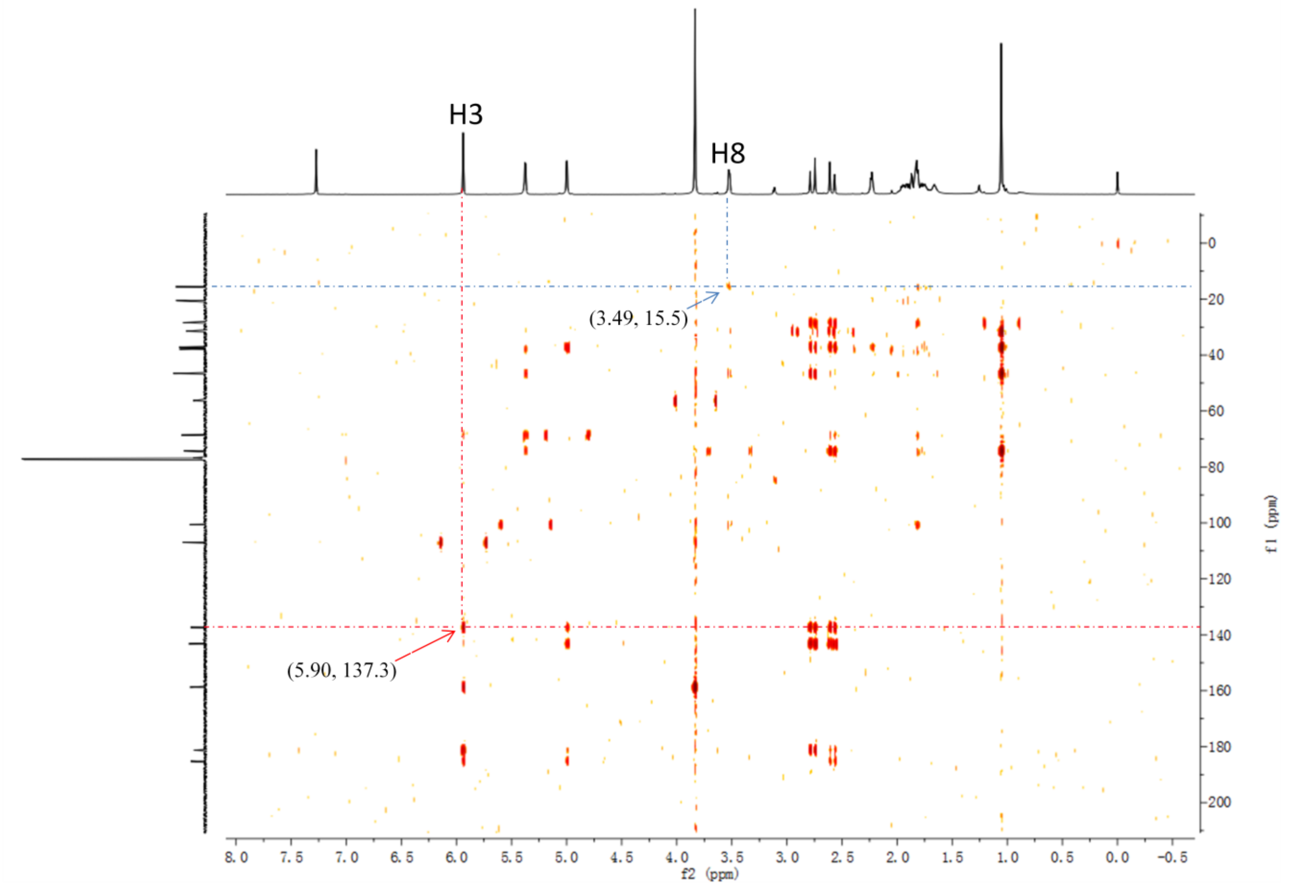

Supplementary Figure 83. HMBC spectrum for Oncocalyxone B (6).

Supplementary Table 1. Screening acids for PEDA reaction

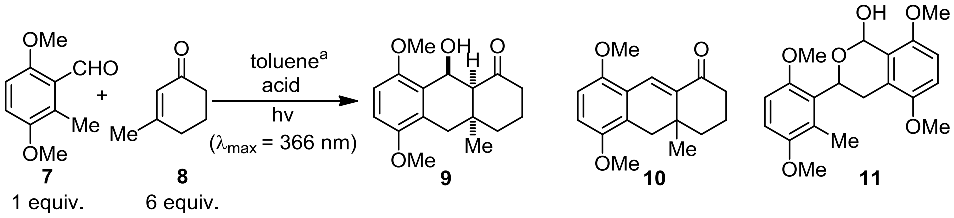

entry	acid (3.0 equiv.)	time	conversion(%)[b]	yield (**9**+**10**,%)[b]	yield(**11**,%)[b]	ratio(**9**/**10**)[b]
1	HCl	3.5 h	80	ND	1.3	--
2	CF_3SO_3H	3.5 h	98	ND	ND	--
3	PPTS	2 h	100	ND	5	--
4	TBSOTf	2 h	98	ND	ND	--
5	$SnCl_4$	3.5 h	44	ND	ND	--
6	$Cu(OTf)_2$	2 h	85	ND	ND	--
7	Et_2AlCl	2 h	99	ND	trace	--
8	$FeCl_3$	2 h	76	ND	ND	--
9	AgOTf	2 h	36	ND	ND	--
10	$AlCl_3$	2 h	74	ND	ND	--
11	$InBr_3$	3 h	76	ND	ND	--
12	$ZrCl_4$	2 h	55	ND	ND	--
13	$AgBF_4$	2 h	44	ND	ND	--
14	$Ti(OEt)_4$	2 h	96	20	11	**10**
15	$TiCl_2Cp_2$	3.5 h	78	ND	ND	--
16	$TiCl_4$	2 h	82	ND	ND	--
17	$TiCl_3$	100 min	92	ND	2.8	--
18	$Ti(On\text{-}Bu)_4$	1 h	100	10	5.9	**9**
19	$Ti(NEt_2)_4$	100 min	100	ND	ND	--
20[c]	$Ti(Ot\text{-}Bu)_4$	30 min	100	52	4	100:1

Reaction scale: aldehyde (0.1 mmol, 1.0 eq), unsaturated ketone (0.6 mmol, 6.0 eq), conc. = 0.02 mol/L. [a] All the photo reactions were run under degassed solvent. [b] Conversion, ratio and yields were determined by ^{1}H NMR spectroscopic crude analysis using CH_2Br_2 as an internal standard, unless noted. [c] Anhydrous and degassed dioxane was used as solvent. ND = not detected

Supplementary Table 2. Screening photo-sources for Titanium-promoted PEDA reaction

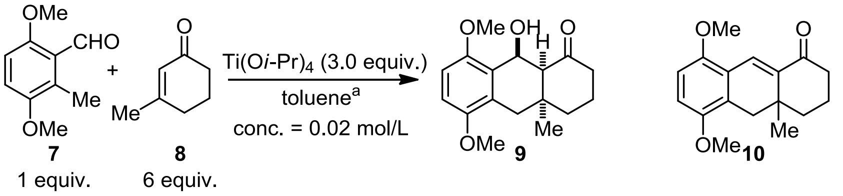

entry	photosource (λ_{max}/nm)	time	conversion(%)[b]	yield (**9**+**10**,%)[b]	ratio(**9**/**10**)[b]
1	254	1.5 h	98	19	3.4 : 1
2	300	45 min	96	55	4.8 : 1
3	366	30 min	100	56	14.3 : 1
4	419	4 h 50 min	66	22	1 : 3.6
5	575	4 h 50 min	62	27	1 : 7.1
6	visible light	18 h 40 min	56	11	1 : 2.4
7	high pressure lamp	35 min	100	51	20 : 1

Reaction scale: aldehyde (0.1 mmol, 1.0 eq), unsaturated ketone (0.6 mmol, 6.0 eq). [a] All the photo reactions were run under degassed solvent. [b] Conversion, ratio and yields were determined by ^{1}H NMR spectroscopic crude analysis using CH_2Br_2 as an internal standard, unless noted.

Supplementary Table 3. Screening solvents for Titanium-promoted PEDA reaction

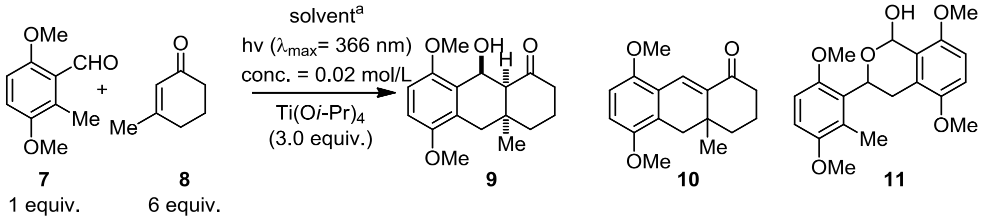

entry	solvent[a]	time	conversion(%)[b]	yield (**9+10**, %)[b]	yield(**11**,%)[b]	ratio(**9**/**10**)[b]
1	$ClCH_2CH_2Cl$	40 min	94	39	5.6	2.2 : 1
2	$CHCl_3$	2 h	90	7.5	1.8	**10**
3	CCl_4	1 h 35 min	97	trace	trace	--
4	THF	30 min	100	65	3	2 : 1
5	THF (dry)	30 min	100	55	3.7	33 : 1
6	DME	1 h 35 min	95	4	2.6	**10**
7	2-MeTHF	35 min	100	48	3	12.5 : 1
8	EtOAc	1.5 h	88	11	5.9	**10**
9	acetone	1 h 35 min	99	trace	2.6	--
10	MeOH	2 h	19	ND	trace	--
11	isopropanol	1 h 35 min	47	ND	ND	--
12	DMF	1 h 35 min	58	ND	ND	--
13	DMF (dry)	30 min	68	12	15	2: 1
14	DMSO (dry)	30 min	61	ND	14	--
15	ethylbenzene	30 min	100	65	2.6	1 : 1
16	m-xylene	30 min	100	62	2.9	1 : 1.2
17	p-xylene	1 h	77	37	2	3.6 : 1
18	xylene	30 min	100	59	3.3	1 : 1.2
19	mesitylene	30 min	100	58	2.8	1 : 1
20	fluorobenzene	30 min	100	37	5.1	1 : 1.4
21	benzotrifluoride	30 min	100	57	4.9	5.6 : 1
22	dioxane	2 h	94	11	trace	**9**
23	toluene/cyclohexane (2:1)	30 min	100	63	2.4	14.3 : 1
24	dioxane/cyclohexane (2:1)	30 min	100	68	1.3	25 : 1
25	toluene/dioxane (1:1)	30 min	100	62	1.8	20 : 1

Reaction scale: aldehyde (0.1 mmol, 1.0 eq), unsaturated ketone (0.6 mmol, 6.0 eq). [a] All the photo reactions were run under degassed solvent. [b] Conversion, ratio and yields were determined by ^1H NMR spectroscopic crude analysis using CH_2Br_2 as an internal standard, unless noted. ND = not detected

Supplementary Table 4. Screening additives for Titanium-promoted PEDA reaction

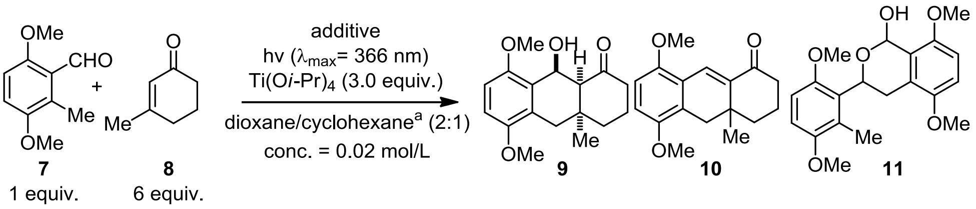

entry	additive	time	conversion(%)[b]	yield (**9+10**, %)[b]	yield(**11**,%)[b]	ratio(**9**/**10**)[b]
1	pyridine (6.0 equiv.)	30 min	100	57	2	6.7 : 1
2	NEt_3 (6.0 equiv.)	30 min	100	56	1	14.3 : 1
3	DIPEA (6.0 equiv.)	30 min	100	46	1.7	14.3 : 1
4	DIPA (6.0 equiv.)	30 min	100	63	2.3	16.7 : 1
5	DBU (6.0 equiv.)	30 min	81	ND	1.7	--
6	none	30 min	100	65	trace	2.5 : 1
7	DMAP (0.3 equiv.)	30 min	100	66	1.7	6.25 : 1
8	HMPA (0.3 equiv.)	30 min	100	63	2.2	6.7 : 1
9	PPh_3 (6.0 equiv.)	30 min	100	ND	ND	--
10	$Ph_3P{=}O$ (0.3 equiv.)	30 min	100	67	2.3	5.9 : 1
11	BINOL (6.0 equiv.)	1 h	66	ND	ND	--
12	DIPA (0.6 equiv.)	30 min	100	68.9	1.8	9.1 : 1

Reaction scale: aldehyde (0.1 mmol, 1.0 eq), unsaturated ketone (0.6 mmol, 6.0 eq). [a] All the photo reactions were run under degassed solvent. [b] Conversion, ratio and yields were determined by ^1H NMR spectroscopic crude analysis using CH_2Br_2 as an internal standard, unless noted. ND = not detected

Supplementary Table 5. Investigation the effect of the dosage of Ti(O*i*-Pr)$_4$ on the reaction yield

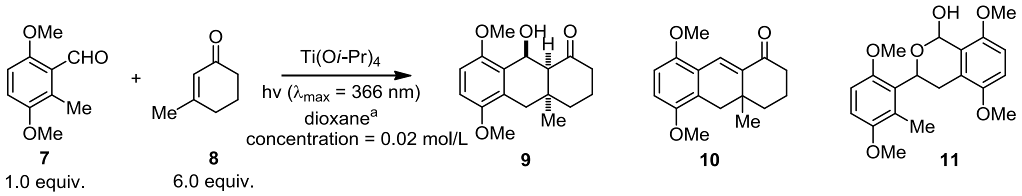

entry	Ti(O*i*-Pr)$_4$ (equiv.)	time	conversion(%)[b]	yield (**9**+**10**, %)[b]	yield(**11**,%)[b]	ratio(**9**/**10**)[b]
1	0.2	30 min	98	trace	trace	---
2	0.5	30 min	95	5.7	1.1	---
3	0.75	30 min	100	37	3.2	33:1
4	1.0	30 min	100	46	3.6	33:1
5	1.2	30 min	100	52	7.0	33:1
6	1.5	30 min	100	53	3.6	50:1
7	1.75	30 min	100	56	3.3	50:1
8	2.0	30 min	100	65.6	4.5	33:1
9	2.2	30 min	100	63.7	3.7	50:1
10	2.5	30 min	100	64	3.8	50:1
11	3.0	30 min	100	65	3.8	50:1
12	4.0	30 min	100	65	3.8	50:1

Reaction scale: aldehyde (0.1 mmol, 1.0 eq), unsaturated ketone (0.6 mmol, 6.0 eq).[a] All the photo reactions were run under dry and degassed solvent. [b] Conversion, ratio and yields were determined by ^{1}H NMR spectroscopic crude analysis using CH_2Br_2 as an internal standard, unless noted.

Supplementary Table 6. Investigation the effect of the dosage of dienophile (**8**) on the reaction yield

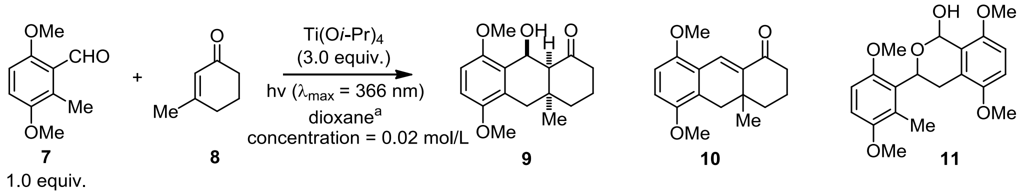

entry	**8** (equiv.)	time	conversion(%)[b]	yield (**9**+**10**, %)[b]	yield(**11**,%)[b]	ratio(**9**/**10**)[b]
1	1.0	30 min	100	50.7	7.4	33:1
2	2.0	30 min	100	62	6	33:1
3	3.0	30 min	100	60	4.6	33:1
4	4.0	30 min	100	60	2.9	50:1
5	5.0	30 min	100	62.6	3.7	50:1
6	6.0	30 min	100	63	3.1	50:1
7	7.0	30 min	100	61	3.7	50:1
8	8.0	30 min	100	62.8	3	33:1
9	9.0	30 min	100	63.6	3.7	33:1
10	10.0	30 min	100	64	2.5	50:1

Reaction scale: aldehyde (0.1 mmol, 1.0 eq). [a] All the photo reactions were run under dry and degassed solvent. [b] Conversion, ratio and yields were determined by ^{1}H NMR spectroscopic crude analysis using CH_2Br_2 as an internal standard, unless noted.

Supplementary Discussion

We believe that Ti(O*i*-Pr)$_4$ not only activates weakly reactive dienophiles, but also helps to stabilize the photo-enolized hydroxy-*o*-quinodimethane species based on our further mechanistic studies. Firstly, we

found that 2-methylbenzaldehyde substrates lacking a methoxy group *ortho* to the aldehyde showed no reactivity in this photoenolization/Diels–Alder reaction, such as the substrates **C1-4** in **Supplementary Figure 84**. This identifies the *ortho* methoxy group as crucial for the reaction, consistent with observations by Nicolaou and coworkers. We considered that the photoenolized hydroxy-*o*-quinodimethane and *ortho* methoxy group may be chelated by Ti(O*i*-Pr)$_4$, forming a relatively stable complex. The *ortho* methoxy may serve as a key neighboring group that helps to stabilize the short-lived photoenolized hydroxy-*o*-quinodimethane diene.

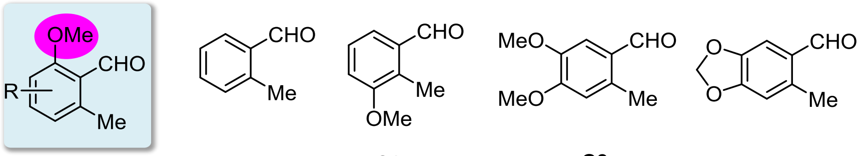

Supplementary Figure 84. Substrates lacking a methoxy group *ortho* to the aldehyde.

To gain further insights into the reaction mechanism, we carefully investigated the effects of dienophile and Ti(O*i*-Pr)$_4$ dosage on the reaction yield using the model reaction between **7** and **8** (**Supplementary Table 5 and 6**). We found that increasing the dosage and concentration of dienophile **8** had little effect on the reaction rate or yield (left curve, **Supplementary Figure 85**). In contrast, the reaction yield depended strongly on the dosage of Ti(O*i*-Pr)$_4$. Photolysis using Ti(O*i*-Pr)$_4$ dosages >2.0 equiv. gave stable and comparable yield (right curve, **Supplementary Figure 85**), while decreasing the dosage dramatically reduced the reaction yield. Using 50 mol % Ti(O*i*-Pr)$_4$ produced the cycloaddition product **9** in only 5.7% yield. These findings suggest that the reaction intermediates of diene and dienophile may interact with twice amounts of Ti(O*i*-Pr)$_4$ during the photoreaction.

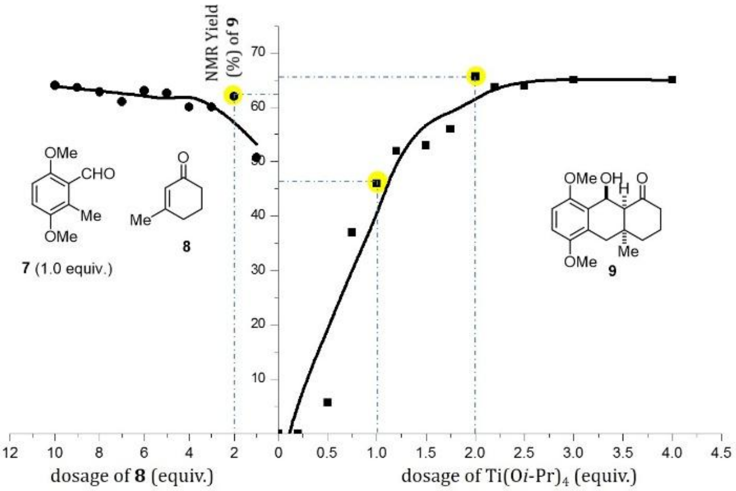

Supplementary Figure 85. Effects of dienophile and Ti(O*i*-Pr)$_4$ dosage on the reaction yield.

We also tried to study the process of this PEDA reaction using NMR spectrum (**Supplementary Figure 86**). A mixture of **7**, **8** and Ti(O*i*-Pr)$_4$ in toluene-d_8 was irradiated with UV light under the optimized conditions. We monitored the reaction using NMR every 5 minutes without quenching the reaction by saturated sodium bicarbonate. We found that the cycloaddition product **9** formed quickly during photolysis, and that it existed mainly as a Ti-chelated complex based on comparison with the NMR spectrum of purified **9**. This Ti-chelated complex could be transformed to product **9** by treatment with sat. $NaHCO_3$. This clearly

indicates that $Ti(Oi\text{-}Pr)_4$ plays a key role in this photoreaction, and that a chelation between $Ti(Oi\text{-}Pr)_4$ and diene/dienophile occurs during this process.

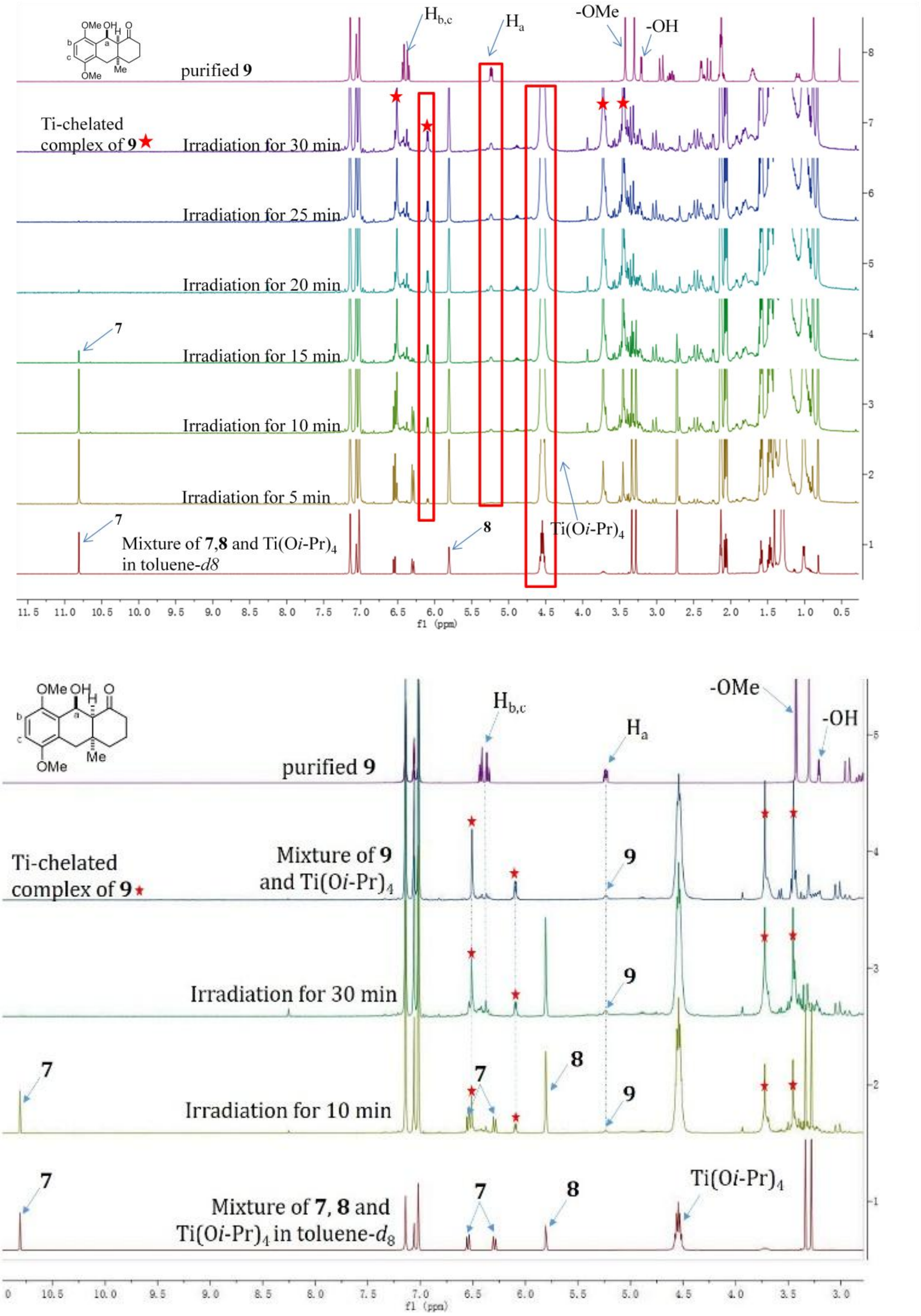

Supplementary Figure 86. Monitor the PEDA reaction with NMR spectrum.

Given these results and structural data on PEDA products, we propose plausible transition states for the reaction as shown in **Supplementary Figure 87**, which presents the formation of **9** as an example. We

consider that the hydroxy-*o*-quinodimethane species is effectively generated via photoenolization, after which the *Z*-dienol and *ortho* methoxy group may be chelated by Ti(O*i*-Pr)$_4$, forming a relatively stable complex. This complex may exist as a monomeric (**C5**) or dimeric titanium complex (**C6**). Although we cannot rule out the possibility of a monomeric form, we think the dimeric form **C6** is more likely, given the observed relationships between Ti dosage and reaction yield. The *ortho* methoxy may serve as a key neighboring group that helps to stabilize the short-lived photoenolized hydroxy-*o*-quinodimethane diene, which then interacts with a cyclic dienophile such as **8** to give a chelated intermediate **C7**. Then the activated enone reacts with the diene component from the *endo* direction, forming the Ti-chelated complex of **9**, which can be detected by NMR (Shown in **Supplementary Figure 86**). After dissociation, cycloaddition product **9** with three consecutive stereogenic centers is generated stereospecifically.

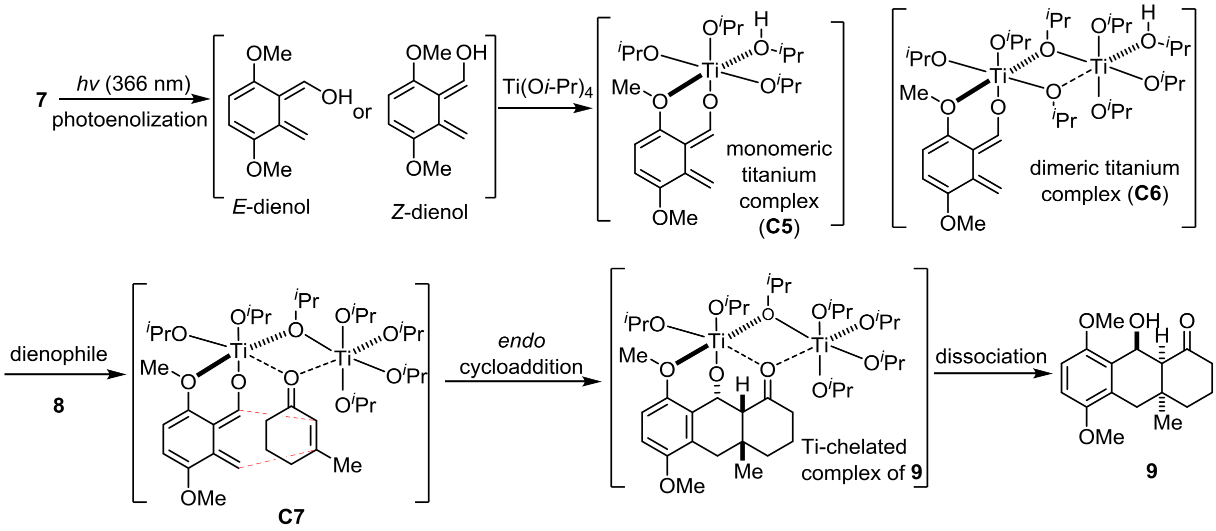

Supplementary Figure 87. Proposed plausible transition states in the PEDA reaction.

Supplementary Methods

General Experimental Procedures: All reactions were carried out under nitrogen unless noted. Anhydrous 1,4-dioxane, dichloromethane, acetonitrile, N,N-dimethylformamide, 1,2-dichloroethane were distilled from calcium hydride. Tetrahydrofuran was distilled from sodium-benzophenone ketyl, anhydrous toluene was distilled from sodium. All degassed solvents were obtained by bubbling N_2 over 40 min. Flash column chromatography was performed as described by Still[1], employing Qingdao Haiyang silica gel 60 (200–300 mesh). TLC analyses were performed on EMD 250 μm Silica Gel HSGF$_{254}$ plates and visualized by quenching of UV fluorescence (λ_{max}=254 nm), or by staining ceric ammonium molybdate, ammonium molybdate, phosphomolybdic acid, or potassium permanganate. The $[\alpha]_D$ was recorded using PolAAr 3005 High Accuracy Polarimeter. Infrared (IR) spectra were obtained using a Bruker tensor 27 infrared spectrometer. ^{1}H and ^{13}C NMR spectra were recorded on a Bruker-500, 400 spectrometer. Chemical shifts for ^{1}H and ^{13}C NMR spectra are reported in ppm (δ) relative to residue protium and carbon resonance in the solvent ($CDCl_3$: δ 7.26, 77.0 ppm) and the multiplicities are presented as follows: s = singlet, d = doublet, t = triplet, q = quartet, m = multiplet, br = broad. We found that the ^{13}C NMR spectrum of some pure compounds have splitting carbon peaks. This may be caused by the NMR equipment or their special

chemical structures. High-resolution mass spectra (HRMS) were acquired on Waters Micromass GCT Premier, and Mass spectra at Agilent 5975C. The photo reactor used for this photolysis is Rayonet RPR-200 (Southern New England Ultraviolet Company). The emission spectra of the 16 lamps for 366 nm in the Rayonet chamber reactor range from 300 to 400 nm with maximum emission wavelength at 366 nm. We use "λ_{max} = 366 nm" as a brief substitute.

(**Notice:** The size of quartz tube we chose for our photoreactions is as follows: 15 centimeters for length, 1.3 centimeters for inside diameter, 1.5 centimeters for outside diameter. As to quartz tube of larger size, the result of this photolysis is not very good.)

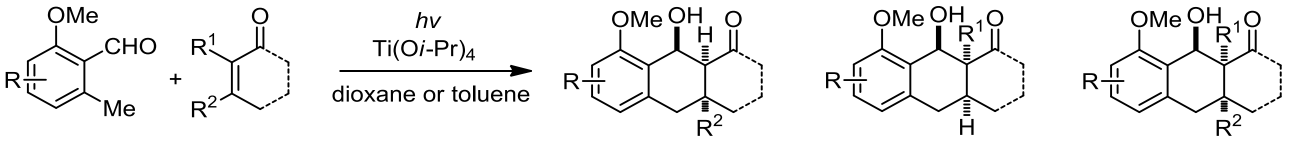

Supplementary Figure 88. General equation for Titanium(IV)-promoted PEDA reaction

General procedure A for Titanium(IV)-promoted photoenolization/Diels-Alder reaction:

To a solution of aromatic aldehyde (0.3 mmol, 1.0 equiv.) in anhydrous and degassed 1,4-dioxane (15 mL, 0.02 M) in quartz tube sealed with rubber plug was added dienophile (1.8 mmol, 6.0 equiv.) (if the dienophile was solid, it was added before the solvent) under N_2, then titanium(IV) isopropoxide (0.9 mmol, 3.0 equiv.) was added, after homogeneous mixing, the solution was photolyzed at rt in a Rayonet chamber reactor (16 lamps) at λ_{max} = 366 nm for certain time described below. Then the reaction mixture was poured into saturated sodium bicarbonate and stirred over 30 min, the above mixture was extracted three times with ethyl acetate, the combined organic phases were washed twice with brine and dried over anhydrous sodium sulfate. The dried solution was filtered and the filtrate was concentrated under vacuum. The residue was purified by silica gel column chromatography to give the corresponding product.

General procedure B for Titanium(IV)-promoted photoenolization/Diels-Alder reaction:

To a solution of aromatic aldehyde (0.3 mmol, 1.0 equiv.) in anhydrous and degassed toluene (15 mL, 0.02 M) in quartz tube sealed with rubber plug was added dienophile (0.45 mmol, 1.5 equiv.) (if the dienophile was solid, it was added before the solvent) under N_2, then titanium(IV) isopropoxide (0.36 mmol, 1.2 equiv.) was added, after homogeneous mixing, the solution was photolyzed at rt in a Rayonet chamber reactor (16 lamps) at λ_{max} = 366 nm for certain time described below. Then the reaction mixture was poured into saturated sodium bicarbonate and stirred over 30 min, the above mixture was extracted three times with ethyl acetate, the combined organic phases were washed twice with brine and dried over anhydrous sodium sulfate. The dried solution was filtered and the filtrate was concentrated under vacuum. The residue was purified by silica gel column chromatography to give the corresponding product.

General procedure C for Titanium(IV)-promoted photoenolization/Diels-Alder reaction:

To a solution of dienophile (0.3 mmol, 1.0 equiv.) and aromatic aldehyde (1.2 mmol, 4.0 equiv.) in anhydrous and degassed toluene (60 mL, concentration for aromatic aldehyde is 0.02 M) in quartz tube sealed with rubber plug was added titanium(IV) isopropoxide (0.9 mmol, 3.0 equiv.) under N_2, after homogeneous mixing, the solution was photolyzed at rt in a Rayonet chamber reactor (16 lamps) at λ_{max} = 366 nm for certain time described below. Then the reaction mixture was poured into saturated sodium bicarbonate and stirred over 30 min, the above mixture was extracted three times with ethyl acetate, the combined organic phases were washed twice with brine and dried over anhydrous sodium sulfate. The dried solution was filtered and the filtrate was concentrated under vacuum. The residue was purified by silica gel column chromatography to give the corresponding product. (Due to need for much solvent, four parallel reactions were conducted with four quartz tubes, 15 ml toluene for each quartz tube, then combined for workup.)

General procedure D for Titanium(IV)-promoted photoenolization/Diels-Alder reaction:

To a solution of dienophile (0.4 mmol, 1.0 equiv.) and aromatic aldehyde (1.6 mmol, 4.0 equiv.) in anhydrous and degassed dioxane (40 mL, concentration for dienophile is 0.01 M) in quartz tube sealed with rubber plug was added titanium(IV) isopropoxide (1.2 mmol, 3.0 equiv.) under N_2, after homogeneous mixing, the solution was photolyzed at rt in a Rayonet chamber reactor (16 lamps) at λ_{max} = 366 nm for certain time described below. Then the reaction mixture was poured into saturated sodium bicarbonate and stirred over 30 min, the above mixture was extracted three times with ethyl acetate, the combined organic phases were washed twice with brine and dried over anhydrous sodium sulfate. The dried solution was filtered and the filtrate was concentrated under vacuum. The residue was purified by silica gel column chromatography to give the corresponding product. (Due to need for much solvent, four parallel reactions were conducted with four quartz tubes, 10 ml dioxane for each quartz tube, then combined for workup. The gas inside was replaced into N_2 with oil pump under the low temperature (liquid nitrogen), because of the dienophile used here can be easily taken away by oil pump at rt.)

Compound **7** was prepared according to the following procedure.

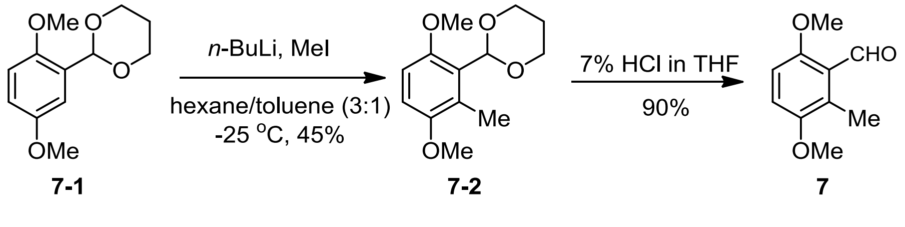

Supplementary Figure 89. Synthesis of aldehyde **7**.

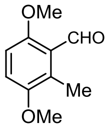

7

To a stirred solution of **7-1** (10.1 g, 45.04 mmol, 1.0 equiv.) in anhydrous toluene/hexane(1:3) (200 mL) was added *n*-BuLi (2.5 M solution in hexane, 36 mL, 90.08 mmol, 2.0 equiv.) dropwise at -25 ^{o}C under N_2, the resulting mixture was vigorously stirred at -25 ^{o}C for 11.5 h, then MeI (11.2 mL, 180.16 mmol, 4.0 equiv.) in anhydrous tetrahydrofuran (80 mL) was was added to the above mixture, after the addition, the reaction mixture was stirred over 5 h. Then the mixture was quenched with saturated sodium bicarbonate (100 mL), the solvent was removed under vacuum and the residue was extracted with ethyl acetate (3×100 mL), the combined organic layer was washed with brine (2×150 mL) and dried over anhydrous sodium sulfate. The dried solution was filtered and the filtrate was concentrated under vacuum. The residue was purified by silica gel column chromatography (2% to 5% ethyl acetate-petroleum ether) to give **7-2** as a colorless oil (4.86 g, 45%).

To a stirred solution of **7-2** (4.9 g, 20.4 mmol, 1.0 equiv.) in tetrahydrofuran (48 mL) was added concentrated hydrochloric acid (12 mL), the resulting mixture was stirred at rt for 1.5 h. Then the solvent was removed under vacuum. The residue was diluted with water (50 mL) and diethyl ether (50 mL), the above mixture was separated to obtain organic layer with a separatory funnel, the aqueous layer was extracted with diethyl ether (2×50 mL), the combined organic layer was washed sequentially with saturated sodium bicarbonate (2×60 mL) and brine (2×60 mL) and dried over anhydrous sodium sulfate. The dried solution was filtered and the filtrate was concentrated under vacuum. The residue was purified by silica gel column chromatography (0% to 5% ethyl acetate-petroleum ether) to give **7** as a white solid (3.3 g, 90%). ^{1}H NMR (400 MHz, $CDCl_3$) δ 10.61 (s, 1H), 7.02 (d, J = 9.0 Hz, 1H), 6.78 (d, J = 9.0 Hz, 1H), 3.85 (s, 3H), 3.80 (s, 3H), 2.46 (s, 3H). The spectroscopic data are identical in all respects to those previously reported.[2]

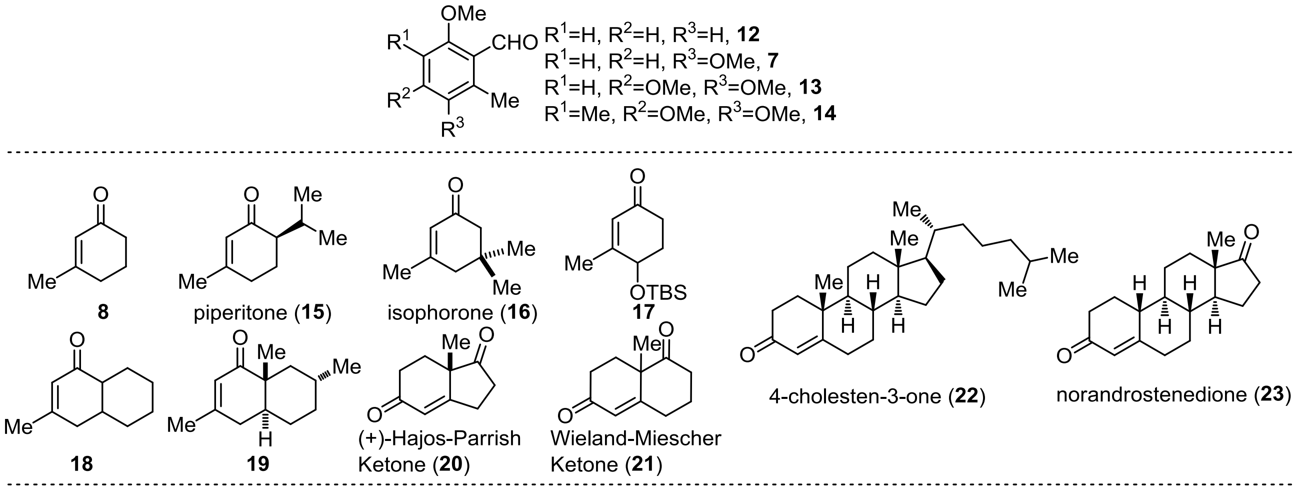

Supplementary Figure 90. Aromatic aldehyde and scope of the PEDA reaction involving *β*-substituted cyclohexenones as dienophiles.

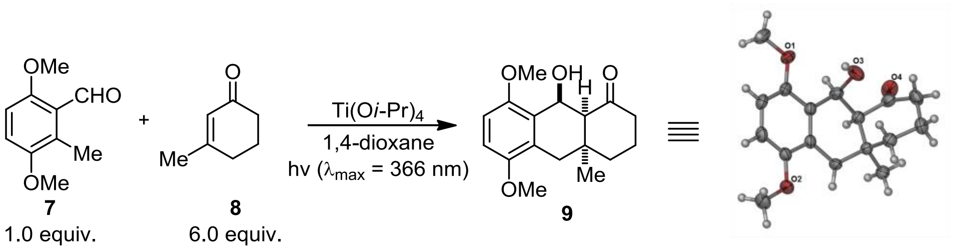

CCDC 1482902

Supplementary Figure 91. Titanium(IV)-promoted PEDA reaction for synthesis of *β*-hydroxyl ketone **9**.

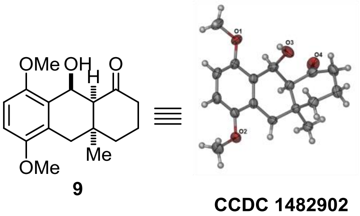

CCDC 1482902

***β*-hydroxy ketone 9.** To a solution of **7** (90 mg, 0.5 mmol, 1.0 equiv.) in anhydrous and degassed 1,4-dioxane (25 mL) in quartz tube sealed with rubber plug was added **8** (340 μL, 3.0 mmol, 6.0 equiv.) under N_2, then titanium(IV) isopropoxide (445 μL, 1.5 mmol, 3.0 equiv.) was added, after homogeneous mixing, the solution was photolyzed at rt in a Rayonet chamber reactor (16 lamps) at λ_{max} = 366 nm for 30 min. The reaction mixture was poured into saturated sodium bicarbonate and stirred over 30 min, the above mixture was extracted with ethyl acetate (3×15 mL), the combined organic phases were washed with brine (2×20 mL) and dried over anhydrous sodium sulfate. The dried solution was filtered and the filtrate was concentrated. The residue was purified by silica gel column chromatography (5% to 15% ethyl acetate-petroleum ether) to give **9** as a white solid (104.5 mg, 72%): R_f = 0.25 (30% ethyl acetate-petroleum ether); m.p. 125 – 127 °C; Recrystallization of **9** from dichlorome-thane/hexane (v/v, 1:3), CCDC 1482902. ^{1}H NMR (500 MHz, $CDCl_3$) δ 6.71 (s, 2H), 5.14 (dd, J = 6.1, 4.1 Hz, 1H), 3.83 (s, 3H), 3.77 (s, 3H), 3.72 (d, J = 4.2 Hz, 1H), 2.76 (d, J = 17.6 Hz, 1H), 2.68 (d, J = 6.2 Hz, 1H), 2.64 – 2.56 (m, 1H), 2.48 – 2.40 (m, 1H), 2.32 (d, J = 17.6 Hz, 1H), 2.02 – 1.93 (m, 3H), 1.56 – 1.47 (m, 1H), 1.06 (s, 3H) ppm; ^{13}C NMR (125 MHz, $CDCl_3$) δ 213.6, 151.6, 151.2, 126.4, 124.9, 108.9, 107.8, 64.6, 58.2, 55.7, 55.6, 41.1, 36.2, 34.2, 33.8, 27.6, 21.7 ppm; IR ν_{max} 3496, 2928, 1688, 1475, 1252, 1081, 1046, 970, 793, 712 cm^{-1}; HRMS–EI (*m*/*z*): [M]$^+$ calcd for $C_{17}H_{22}O_4$, 290.1518; found, 290.1515.

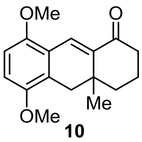

Unsaturated ketone 10: ^{1}H NMR (400 MHz, $CDCl_3$) δ 7.77 (s, 1H), 6.83 (d, J = 8.9 Hz, 1H), 6.67 (dd, J = 8.9, 0.7 Hz, 1H), 3.803 (s, 3H), 3.797 (s, 3H), 3.09 (d, J = 16.4 Hz, 1H), 2.67 – 2.57 (m, 1H), 2.50 (d, J = 16.4 Hz, 1H), 2.43 – 2.31 (m, 1H), 2.05 – 1.83 (m, 3H), 1.75 (td, J = 12.9, 4.2 Hz, 1H), 0.99 (s, 3H). ^{13}C NMR (100 MHz, $CDCl_3$) δ 199.9, 151.9, 150.8, 139.8, 126.7, 125.8, 122.2, 113.0, 108.6, 56.2, 55.8, 39.9, 37.9, 37.1, 33.9, 23.3, 18.7. HRMS–EI (*m*/*z*): [M]$^+$ calcd for $C_{17}H_{20}O_3$, 272.1412; found, 272.1411.

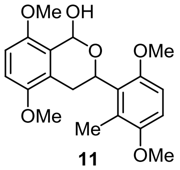
11

Hetero Diels-Alder product 11 mentioned in the article: R_f = 0.15 (20% ethyl acetate-petroleum ether); m.p. 155 – 157 ^oC; ^1H NMR (400 MHz, $CDCl_3$) δ 6.80 – 6.70 (m, 4H), 6.28 (d, J = 2.3 Hz, 1H), 6.03 (dd, J = 12.1, 4.0 Hz, 1H), 3.85 (s, 3H), 3.78 (s, 3H), 3.77 (s, 3H), 3.76 (s, 3H), 3.07 (dd, J = 17.8, 12.2 Hz, 1H), 3.02 (d, J = 3.2 Hz, 1H), 2.82 (dd, J = 17.7, 4.0 Hz, 1H), 2.36 (s, 3H) ppm; ^{13}C NMR (100 MHz, $CDCl_3$) δ 152.7, 151.9, 150.9, 150.8, 128.7, 128.1, 125.4, 124.8, 110.5, 109.6, 109.0, 108.1, 88.8, 63.2, 56.37 (d, J = 2.1 Hz), 56.23 (d, J = 2.2 Hz), 55.96 (d, J = 2.3 Hz), 55.68 (d, J = 2.0 Hz), 26.81, 12.70 ppm; IR ν_{max} 3459, 2933, 2834, 1602, 1481, 1252, 1080, 1021, 788, 716 cm^{-1}; HRMS–EI (m/z): $[M]^+$ calcd for $C_{20}H_{24}O_6$, 360.1573; found, 360.1579.

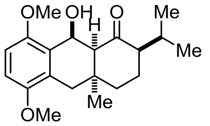
24

***β*-hydroxyl ketone 24** (61 mg) was prepared according to general procedure A from **7** (0.3 mmol) and **15** in 61% yield. The reaction time was 30 min under λ_{max} = 366 nm light. The products were isolated through silica gel column chromatography (3% to 6% ethyl acetate-petroleum ether) as a white solid: R_f = 0.59 (30% ethyl acetate-petroleum ether); m.p. 160 – 162 ^oC; $[\alpha]^{26}_D$ = +44.2 (c = 0.75, $CHCl_3$); ^1H NMR (400 MHz, $CDCl_3$) δ 6.71 (d, J = 8.9 Hz, 1H), 6.68 (d, J = 9.0 Hz, 1H), 5.14 (dd, *J* = 5.9, 3.5 Hz, 1H), 3.81 (s, 3H), 3.76 (s, 3H), 3.61 (d, *J* = 3.8 Hz, 1H), 2.77 (d, *J* = 17.6 Hz, 1H), 2.65 (d, *J* = 6.1 Hz, 1H), 2.50 – 2.30 (m, 3H), 2.01 – 1.87 (m, 2H), 1.86 – 1.75 (m, 1H), 1.61 – 1.51 (m, 1H), 1.01 (s, 3H), 0.92 (d, *J* = 2.9 Hz, 3H), 0.91 (d, *J* = 3.1 Hz, 3H) ppm; ^{13}C NMR (100 MHz, $CDCl_3$) δ 214.7, 151.5, 151.2, 126.6, 125.0, 109.0, 107.8, 64.2, 57.3, 55.9, 55.7, 55.6, 35.6, 34.4, 32.9, 27.7, 26.8, 22.2, 20.4, 18.7 ppm; IR ν_{max} 3536, 2954, 2930, 1684, 1473, 1255, 1084, 800, 709 cm^{-1}; HRMS–EI (*m/z*): $[M]^+$ calcd for $C_{20}H_{28}O_4$, 332.1988; found, 332.1984.

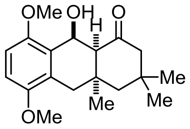
25

***β*-hydroxyl ketone 25** (73 mg) was prepared according to general procedure A from **7** (0.3 mmol) and **16** in 76% yield. The reaction time was 30 min under λ_{max} = 366 nm light. The products were isolated through silica gel column chromatography (6% to 10% ethyl acetate-petroleum ether) as a white solid: R_f = 0.18 (20% ethyl acetate-petroleum ether); m.p. 129 – 131 ^oC; ^1H NMR (400 MHz, $CDCl_3$) δ 6.72 (d, J = 8.9 Hz, 1H), 6.67 (d, J = 8.9 Hz, 1H), 5.34 (t, J = 3.8 Hz, 1H), 3.79 (s, 3H), 3.75 (s, 3H), 3.32 (d, J = 3.3 Hz, 1H), 2.79 (d, J = 16.4 Hz, 1H), 2.58 (d, J = 16.4 Hz, 1H), 2.48 (d, J = 5.1 Hz, 1H), 2.42 (d, J = 14.1 Hz, 1H), 2.33 (d, J = 14.1 Hz, 1H), 1.82 (d, J = 14.2 Hz, 1H), 1.62 (d, J = 14.2 Hz, 1H), 1.12 (s, 3H), 1.07 (s, 3H), 1.06 (s, 3H) ppm; ^{13}C NMR (100 MHz, $CDCl_3$) δ 214.0, 151.1, 150.9, 126.7, 126.1, 109.8, 108.2, 64.6, 57.5, 55.9, 55.7, 54.1, 50.5, 36.8, 35.0, 34.6, 32.7, 32.2, 30.9 ppm; IR ν_{max} 3534, 2935, 2875, 1692, 1480, 1259, 1078, 806, 716 cm^{-1}; HRMS–EI (*m/z*): $[M]^+$ calcd for $C_{19}H_{26}O_4$, 318.1831; found, 318.1829.

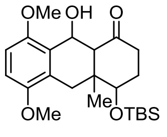

26

***β*-hydroxyl ketone 26** (82 mg, dr = 4:1) was prepared according to general procedure B from **7** (0.3 mmol) and **17**[3,4] in 64% yield. The reaction time was 30 min under λ_{max} = 366 nm light. The products were isolated through silica gel column chromatography (3% to 15% ethyl acetate-petroleum ether) as a white solid (**26a**, 64 mg, 50%) and a colorless oil (**26b**, 18mg, 14%)

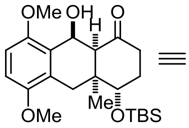

26a

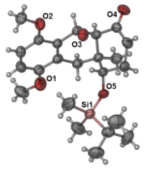

CCDC 1482898

***β*-hydroxyl ketone 26a:** R_f = 0.49 (20% ethyl acetate-petroleum ether); m.p. 114 – 116 ^{o}C; Recrystallization of **26a** from dichloromethane/hexane (v/v, 1:3), CCDC 1482898. ^{1}H NMR (400 MHz, $CDCl_3$) δ 6.70 (s, 2H), 5.10 (dd, J = 6.3, 3.6 Hz, 1H), 4.15 (dd, J = 8.3, 4.7 Hz, 1H), 3.83 (s, 3H), 3.76 (s, 3H), 3.63 (d, J = 3.4 Hz, 1H), 3.10 (d, J = 17.8 Hz, 1H), 2.80 (d, J = 6.3 Hz, 1H), 2.75 – 2.64 (m, 1H), 2.63 – 2.53 (m, 1H), 2.13 – 2.01 (m, 2H), 1.96 – 1.84 (m, 1H), 1.04 (s, 3H), 0.91 (s, 9H), -0.02 (s, 3H), -0.14 (s, 3H) ppm; ^{13}C NMR (100 MHz, $CDCl_3$) δ 213.1, 151.4, 151.2, 126.6, 124.5, 108.7, 107.6, 69.2, 64.1, 56.8, 55.6, 55.4, 39.6, 39.2, 31.3, 29.7, 25.8 (3C), 22.1, 18.0, -4.4, -5.6 ppm; IR ν_{max} 3527, 2926, 1698, 1460, 1254, 1078, 834, 774 cm^{-1}; HRMS–EI (*m/z*): [M]$^{+}$ calcd for $C_{23}H_{36}O_5Si$, 420.2332; found, 420.2327.

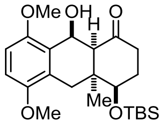

26b

***β*-hydroxyl ketone 26b:** R_f = 0.29 (20% ethyl acetate-petroleum ether); ^{1}H NMR (400 MHz, $CDCl_3$) δ 6.69 (d, J = 8.9 Hz, 1H), 6.66 (d, J = 8.9 Hz, 1H), 5.15 (dd, J = 10.1, 4.9 Hz, 1H), 4.78 (d, J = 10.1 Hz, 1H), 3.80 (s, 3H), 3.78 (d, J = 1.3 Hz, 1H), 3.77 (s, 3H), 2.96 (d, J = 18.0 Hz, 1H), 2.88 (ddd, J = 16.0, 11.3, 7.9 Hz, 1H), 2.54 (d, J = 4.8 Hz, 1H), 2.49 – 2.37 (m, 2H), 2.27 – 2.17 (m, 1H), 2.05 – 1.95 (m, 1H), 1.10 (s, 3H), 0.71 (s, 9H), 0.11 (s, 3H), -0.05 (s, 3H) ppm; ^{13}C NMR (100 MHz, $CDCl_3$) δ 212.3, 151.8, 150.4, 127.0, 125.5, 109.1, 108.2, 75.5, 62.8, 58.1, 56.3, 55.6, 39.4, 36.7, 32.5, 29.5, 29.1, 25.4 (3C), 17.8, -4.7, -5.5 ppm; IR ν_{max} 3404, 2932, 2855, 1703, 1599, 1473, 1254, 1056, 969, 833, 785, 725 cm^{-1}; HRMS–ESI (*m/z*): [M+Na]$^{+}$ calcd for $C_{23}H_{36}O_5NaSi$, 443.2230; found, 443.2223.

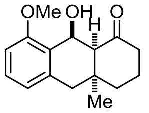

27

***β*-hydroxyl ketone 27** (31.3 mg) was prepared according to general procedure A from **12**[5] (0.3 mmol) and **8** in 40% yield. The reaction time was 30 min under λ_{max} = 366 nm light. The products were isolated through silica gel column chromatography (3% to 10% ethyl acetate-petroleum ether) as a colorless oil: R_f = 0.25 (20% ethyl acetate-petroleum ether); ^{1}H NMR (500 MHz, $CDCl_3$) δ 7.18 (t, J = 7.9 Hz, 1H), 6.75 (d, J = 8.2 Hz, 1H), 6.71 (d, J = 7.6 Hz, 1H), 5.20 (dd, J = 6.1, 4.1 Hz, 1H), 3.86 (s, 3H), 3.50 (d, J = 4.1 Hz, 1H), 2.85 (d, J = 16.5 Hz, 1H), 2.67 – 2.59 (m, 2H), 2.49 – 2.40 (m, 2H), 2.03 – 1.92 (m, 3H), 1.53 – 1.46 (m, 1H), 1.04 (s, 3H) ppm; ^{13}C NMR (125 MHz, $CDCl_3$) δ 213.9, 157.5, 136.2, 128.4, 125.3, 121.6, 108.2, 64.4, 58.7, 55.4, 40.92, 40.87, 36.2, 33.8, 27.5, 21.3 ppm; HRMS–EI (*m/z*): [M]$^{+}$ calcd for $C_{16}H_{20}O_3$, 260.1412; found, 260.1409.

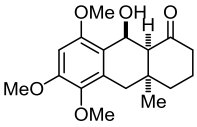

28

***β*-hydroxyl ketone 28** (63 mg) was prepared according to general procedure B from **13** (0.3 mmol) (synthesis of **13** see **Supplementary Figure 98**) and **8** in 66% yield. The reaction time was 30 min under λ_{max} = 366 nm light. The products were isolated through silica gel column chromatography (10% to 20% ethyl acetate-petroleum ether) as a colorless soil: R_f = 0.14 (20% ethyl acetate-petroleum ether); ^{1}H NMR (400 MHz, $CDCl_3$) δ 6.41 (s, 1H), 5.15 (dd, J = 5.8, 4.2 Hz, 1H), 3.87 (s, 3H), 3.85 (s, 3H), 3.72 (s, 3H), 3.40 (d, J = 4.3 Hz, 1H), 2.83 (d, J = 17.0 Hz, 1H), 2.64 – 2.54 (m, 2H), 2.50 – 2.37 (m, 2H), 2.01 – 1.91 (m, 3H), 1.57 – 1.47 (m, 1H), 1.04 (s, 3H) ppm; ^{13}C NMR (100 MHz, $CDCl_3$) δ 214.2, 154.0, 152.3, 140.3, 130.1, 118.1, 95.1, 64.4, 60.3, 58.5, 56.0, 55.8, 40.9, 36.1, 34.4, 34.2, 27.7, 21.3 ppm; IR ν_{max} 3442, 2928, 1692, 1597, 1460, 1324, 1231, 1202, 1087, 1047, 973, 809 cm^{-1}; HRMS–EI (*m*/*z*): $[M]^+$ calcd for $C_{18}H_{24}O_5$, 320.1624; found, 320.1628.

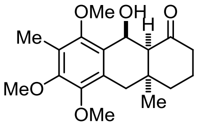

29

***β*-hydroxyl ketone 29** (67 mg) was prepared according to general procedure A from **14**[6] (0.3 mmol) and **8** in 67% yield. The reaction time was 80 min under λ_{max} = 366 nm light. The products were isolated through silica gel column chromatography (3% to 8% ethyl acetate-petroleum ether) as a colorless oil: R_f = 0.39 (30% ethyl acetate-petroleum ether); ^{1}H NMR (400 MHz, $CDCl_3$) δ 5.15 (t, J = 5.3 Hz, 1H), 3.82 (s, 3H), 3.76 (s, 6H), 3.59 (d, J = 5.2 Hz, 1H), 2.77 (d, J = 16.7 Hz, 1H), 2.59 (d, J = 5.8 Hz, 1H), 2.57 – 2.50 (m, 1H), 2.49 – 2.39 (m, 2H), 2.18 (s, 3H), 2.03 – 1.94 (m, 2H), 1.94 – 1.85 (m, 1H), 1.63 – 1.55 (m, 1H), 1.04 (s, 3H) ppm; ^{13}C NMR (100 MHz, $CDCl_3$) δ 214.6, 153.1, 151.4, 146.9, 127.2, 126.1, 123.3, 65.1, 61.0, 60.2, 60.1, 58.7, 40.9, 36.7, 35.4, 33.6, 27.9, 21.3, 9.3 ppm; IR ν_{max} 3482, 2935, 1694, 1461, 1112, 1067, 1013, 967, 732 cm^{-1}; HRMS–EI (*m*/*z*): $[M]^+$ calcd for $C_{19}H_{26}O_5$, 334.1780; found, 334.1779.

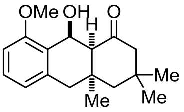

30

***β*-hydroxyl ketone 30** (58 mg) was prepared according to general procedure B from **12** (0.3 mmol) and **16** in 67% yield. The reaction time was 30 min under λ_{max} = 366 nm light. The products were isolated through silica gel column chromatography (5% to 10% ethyl acetate-petroleum ether) as a white solid: R_f = 0.35 (20% ethyl acetate-petroleum ether); m.p. 135 – 137 ^{o}C; ^{1}H NMR (400 MHz, $CDCl_3$) δ 7.19 (t, J = 7.9 Hz, 1H), 6.73 (dd, J = 14.9, 7.9 Hz, 2H), 5.48 (d, J = 4.4 Hz, 1H), 3.83 (s, 3H), 3.11 (d, J = 15.1 Hz, 1H), 2.94 (s, 1H), 2.51 – 2.30 (m, 4H), 1.79 (d, J = 14.2 Hz, 1H), 1.67 (d, J = 14.2 Hz, 1H), 1.14 (s, 3H), 1.09 (s, 3H), 1.08 (s, 3H) ppm; ^{13}C NMR (100 MHz, $CDCl_3$) δ 214.7, 156.5, 138.0, 128.7, 125.4, 121.2, 108.5, 64.4, 58.1, 55.4, 54.1, 51.2, 43.0, 37.3, 34.8, 32.4, 32.0, 31.4 ppm; IR ν_{max} 3499, 2945, 1686, 1591, 1470, 1266, 1091, 978, 787, 757, 729 cm^{-1}; HRMS–EI (*m*/*z*): $[M]^+$ calcd for $C_{18}H_{24}O_3$, 288.1725; found, 288.1728.

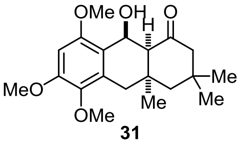

31

***β*-hydroxyl ketone 31** (76 mg) was prepared according to general procedure B from **13** (0.3 mmol) and **16** in 72% yield. The reaction time was 30 min under λ_{max} = 366 nm light. The products were isolated through silica gel column chromatography (7% to 15% ethyl acetate-petroleum ether) as a white solid: R_f = 0.39 (30% ethyl acetate-petroleum ether); ^{1}H NMR (400 MHz, $CDCl_3$) δ 6.35 (s, 1H), 5.41 (s, 1H), 3.85 (s, 3H), 3.79 (s, 3H), 3.69 (s, 3H), 2.87 (d, J = 3.0 Hz, 1H), 2.83 (d, J = 15.4 Hz, 1H), 2.74 (d, J = 15.3 Hz, 1H), 2.43 (d, J = 13.7 Hz, 1H), 2.30 (d, J = 13.8 Hz, 1H), 2.26 (d, J = 4.2 Hz, 1H), 1.78 (d, J = 14.2 Hz, 1H), 1.67 (d, J = 14.2 Hz, 1H), 1.11 (s, 3H), 1.06 (s, 3H), 1.05 (s, 3H) ppm; ^{13}C NMR (100 MHz, $CDCl_3$) δ 214.9, 152.8, 152.6, 140.2, 131.4, 118.1, 94.6, 64.2, 60.7, 58.0, 55.8, 55.7, 54.0, 51.4, 36.8, 36.0, 34.6, 32.3, 31.7, 31.5 ppm; IR ν_{max} 3509, 2953, 2923, 2855, 1684, 1599, 1492, 1461, 1232, 1086, 1039, 804 cm^{-1}; HRMS–EI (*m*/*z*): $[M]^+$ calcd for $C_{20}H_{28}O_5$, 348.1937; found, 348.1943.

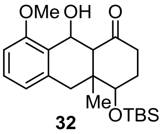

32

***β*-hydroxyl ketone 32** (74.6 mg, dr = 4.1:1:1) was prepared according to general procedure B from **12** (0.3 mmol) and **17** in 63% yield. The reaction time was 30 min under λ_{max} = 366 nm light. The products were isolated through silica gel column chromatography (3% to 8% ethyl acetate-petroleum ether) as a white solid (**32a**, 55.8mg, 47%), a colorless oil (**32b**, 10 mg, 8.5%) and a colorless oil (**32c**, 8.8 mg, 7.5%)

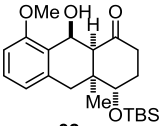

32a

***β*-hydroxyl ketone 32a:** R_f = 0.60 (20% ethyl acetate-petroleum ether); m.p. 120 – 122 °C; ^{1}H NMR (400 MHz, $CDCl_3$) δ 7.19 (t, J = 7.9 Hz, 1H), 6.75 (d, J = 8.2 Hz, 1H), 6.70 (d, J = 7.7 Hz, 1H), 5.15 (d, J = 6.3 Hz, 1H), 4.18 (dd, J = 8.7, 4.8 Hz, 1H), 3.87 (s, 3H), 3.44 (s, 1H), 3.08 (d, J = 16.7 Hz, 1H), 2.78 – 2.67 (m, 2H), 2.63 – 2.52 (m, 1H), 2.26 (d, J = 16.7 Hz, 1H), 2.11 – 2.01 (m, 1H), 1.96 – 1.84 (m, 1H), 1.02 (s, 3H), 0.90 (s, 9H), -0.02 (s, 3H), -0.12 (s, 3H) ppm; ^{13}C NMR (100 MHz, $CDCl_3$) δ 213.1, 157.3, 135.6, 128.5, 125.4, 121.8, 108.0, 69.2, 63.9, 57.5, 55.4, 39.8, 39.3, 38.0, 29.5, 25.8 (3C), 21.8, 18.0, -4.2, -5.0 ppm; IR ν_{max} 3537, 2949, 2854, 1697, 1592, 1465, 1248, 1086, 831, 772 cm^{-1}; HRMS–EI (*m*/*z*): $[M]^+$ calcd for $C_{22}H_{34}O_4Si$, 390.2226; found, 390.2224.

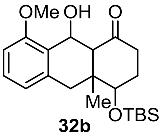

32b

***β*-hydroxyl ketone 32b:** R_f = 0.43 (20% ethyl acetate-petroleum ether); ^{1}H NMR (400 MHz, $CDCl_3$) δ 7.15 (t, J = 7.9 Hz, 1H), 6.75 – 6.69 (m, 2H), 5.50 (s, 1H), 3.98 (dd, J = 10.6, 4.8 Hz, 1H), 3.90 (s, 3H), 3.27 (s, 1H), 2.78 (s, 1H), 2.54 (d, J = 4.8 Hz, 2H), 2.51 – 2.38 (m, 2H), 2.16 – 2.07 (m, 1H), 2.01 – 1.97 (m, 1H), 1.21 (s, 3H), 0.90 (s, 9H), 0.11 (s, 3H), 0.09 (s, 3H) ppm; ^{13}C NMR (100 MHz, $CDCl_3$) δ 209.4, 157.7, 135.6, 128.0, 125.2, 122.2, 107.8, 76.2, 62.6, 58.2, 55.4 (d, J = 2.1 Hz), 41.9, 38.4, 32.2, 31.1, 25.9 (3C), 24.7, 18.1, -4.1, -4.9 ppm; IR ν_{max} 3575, 2926, 2854, 1706, 1590,

1460, 1250, 1076, 832, 769 cm^{-1}; HRMS–ESI (*m*/*z*): [M+Na]$^+$ calcd for $C_{22}H_{34}O_4NaSi$, 413.2124; found, 413.2117.

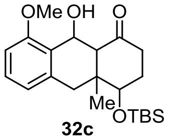
32c

***β*-hydroxyl ketone 32c:** R$_f$ = 0.38 (20% ethyl acetate-petroleum ether); ^{1}H NMR (400 MHz, $CDCl_3$) δ 7.15 (t, J = 7.9 Hz, 1H), 6.69 (dd, J = 14.6, 7.9 Hz, 2H), 5.27 (dd, J = 8.6, 4.6 Hz, 1H), 4.44 (d, J = 8.7 Hz, 1H), 3.83 (s, 3H), 3.80 – 3.77 (m, 1H), 3.16 (d, J = 16.5 Hz, 1H), 2.82 – 2.73 (m, 1H), 2.49 (d, J = 4.5 Hz, 1H), 2.48 – 2.38 (m, 2H), 2.28 – 2.18 (m, 1H), 2.06 – 2.97 (m, 1H), 1.07 (s, 3H), 0.78 (s, 9H), 0.13 (s, 3H), 0.02 (s, 3H) ppm; ^{13}C NMR (100 MHz, $CDCl_3$) δ 212.3, 157.4, 137.1, 128.4, 126.0, 120.8, 108.4, 74.9, 62.9, 58.4, 55.7 (d, J = 2.1 Hz), 40.9, 37.8, 36.4, 28.9, 28.8, 25.6 (3C), 17.9, -4.6, -5.1 ppm; IR ν_{max} 3525, 2930, 2856, 1685, 1593, 1467, 1258, 1049, 973, 835, 773 cm^{-1}; HRMS–ESI (*m*/*z*): [M+Na]$^+$ calcd for $C_{22}H_{34}O_4NaSi$, 413.2124; found, 413.2117.

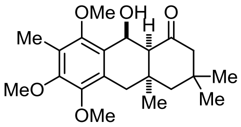
33

***β*-hydroxyl ketone 33** (92 mg) was prepared according to general procedure B from **14** (0.3 mmol) and **16** in 84% yield. The reaction time was 30 min under λ_{max} = 366 nm light. The products were isolated through silica gel column chromatography (2% to 10% ethyl acetate-petroleum ether) as a white solid: R$_f$ = 0.62 (30% ethyl acetate-petroleum ether); m.p. 113 – 115 ^{o}C; ^{1}H NMR (400 MHz, $CDCl_3$) δ 5.46 (d, J = 3.4 Hz, 1H), 3.82 (s, 3H), 3.77 (s, 3H), 3.76 (s, 3H), 2.93 (s, 1H), 2.82 (d, J = 14.9 Hz, 1H), 2.76 (d, J = 14.9 Hz, 1H), 2.52 (d, J = 13.3 Hz, 1H), 2.28 (d, J = 13.3 Hz, 1H), 2.22 (d, J = 3.9 Hz, 1H), 2.18 (s, 3H), 1.73 (s, 2H), 1.16 (s, 3H), 1.12 (s, 3H), 1.07 (s, 3H) ppm; ^{13}C NMR (100 MHz, $CDCl_3$) δ 215.4, 152.1, 151.8, 147.1, 128.7, 126.3, 123.1, 64.7, 61.9, 60.6, 60.2, 58.4, 53.7, 51.9, 37.5, 36.1, 35.0, 32.4, 32.1, 31.5, 9.3 ppm; IR ν_{max} 3426, 2953, 1678, 1460, 1401, 1248, 1116, 1049, 1013, 735 cm^{-1}; HRMS–EI (*m*/*z*): [M]$^+$ calcd for $C_{21}H_{30}O_5$, 362.2093; found, 362.2096.

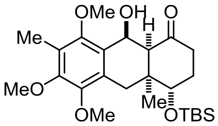
34a

***β*-hydroxyl ketone 34** (112.3 mg, dr = 11.4:1) was prepared according to general procedure B from **14** (0.3 mmol) and **17** in 82% yield. The reaction time was 30 min under λ_{max} = 366 nm light. The products were isolated through silica gel column chromatography (3% to 8% ethyl acetate-petroleum ether) to give a mixture of **34** (112.3 mg) as a white solid. Major isomer **34a**: R$_f$ = 0.52 (10% ethyl acetate-petroleum ether); ^{1}H NMR (400 MHz, $CDCl_3$) δ 5.09 (d, J = 5.4 Hz, 1H), 3.96 (dd, J = 5.5, 4.8, 1H), 3.80 (s, 3H), 3.75 (s, 6H), 3.59 (s, 1H), 2.88 (d, J = 16.8 Hz, 1H), 2.84 (d, J = 5.5 Hz, 1H), 2.71 – 2.59 (m, 1H), 2.57 – 2.47 (m, 1H), 2.32 (d, J = 16.7 Hz, 1H), 2.18 (s, 3H), 2.17 – 2.07 (m, 1H), 2.00 – 1.89 (m, 1H), 1.02 (s, 3H), 0.92 (s, 9H), 0.04 (s, 3H), -0.01 (s, 3H). ppm; ^{13}C NMR (100 MHz, $CDCl_3$) δ 214.2, 152.9, 151.4, 146.9, 126.8, 126.3, 123.3, 71.5, 64.9, 61.0, 60.09, 60.06, 56.3, 41.5, 38.3, 31.3, 29.6, 25.8 (3C), 22.5, 18.0, 9.4, -4.2, -5.1 ppm; IR ν_{max} 3398, 2933, 2857, 1683, 1464, 1253, 1102, 1075, 834, 771 cm^{-1}; HRMS–EI (*m*/*z*): [M]$^+$ calcd for $C_{25}H_{40}O_6Si$, 464.2594; found, 464.2592.

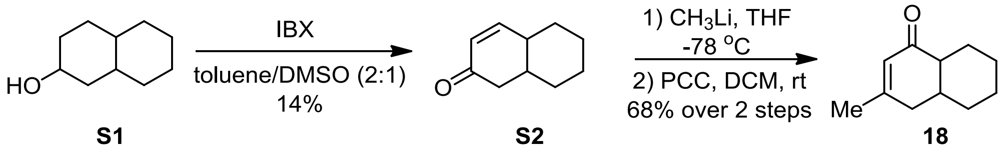

Supplementary Figure 92. Synthesis of enone **18**.

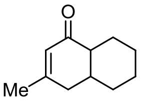

18

Enone 18: To a stirred solution of decalin-2-ol (mixture of isomers) **S1** (CAS: 825-51-4, TCI) (5.0 g, 32.4 mmol, 1.0 equiv.) in toluene/DMSO (2/1) (75 mL) was added 2-iodoxybenzoic acid (22.6 g, 81.0 mmol, 2.5 equiv.), the resulting mixture was stirred at 85 °C over 6 h. After cooling to rt, the reaction mixture was quenched with saturated sodium bicarbonate, the mixture was extracted with ethyl acetate (3×80 mL), the combined organic layer was washed with brine (2×80 mL) and dried over anhydrous sodium sulfate. The dried solution was filtered and the filtrate was concentrated under vacuum. The residue was purified by silica gel column chromatography (0.5% to 1% ethyl acetate-petroleum ether) to give **S2** as a light yellow oil (680 mg, 14%).

To a stirred solution of **S2** (205 mg, 1.33 mmol, 1.0 qeuiv.) in anhydrous tetrahydrofuran (10 mL) was added methyllithium (1.3 M solution in diethyl ether, 1.53 mL, 2.0 mmol, 1.5 equiv.) dropwise at -78 °C under N_2, the resulting mixture was stirred at -78 °C until TLC showed **S2** was consumed completely. Then the reaction was quenched with saturated ammonium chloride (10 mL), the mixture was extracted with ethyl acetate (3×10 mL), the combined organic layer was washed with brine (2×15 mL) and dried over anhydrous sodium sulfate. The dried solution was filtered and the filtrate was concentrated under vacuum. The residue was drained completely with oil pump and dissolved in dichloromethane (10 mL), then pyridinium chlorochromate (1.14 g, 5.3 mmol, 4.0 equiv.) was added to the above mixture, the resulting mixture was stirred at rt until TLC showed no material was remaining. The reaction mixture was filtered through a short pad of column chromatography and rinsed with ethyl acetate, the filtrate was concentrated under vacuum. The residue was purified by silica gel column chromatography (5% to 7.5% ethyl acetate-petroleum ether) to give **18** as a light yellow oil (148 mg, 68% over 2 steps, *trans*:*cis* = 1:1.4). R_f = 0.35 (10% ethyl acetate-petroleum ether); ^{1}H NMR (400 MHz, $CDCl_3$) δ 5.81 (s, 1H), 2.40 – 2.04 (m, 4H), 1.93 (s, 3H, major), 1.91 (s, 3H,major), 1.87 – 1.65 (m, 3H), 1.60 – 1.32 (m, 4H), 1.30 – 1.00 (m, 1H) ppm; ^{13}C NMR (100 MHz, $CDCl_3$) δ 202.0, 201.2, 160.6, 160.3, 126.2, 125.5, 50.0, 46.7, 39.9, 39.0, 34.8, 33.6, 28.8, 25.7, 25.5, 25.0, 24.4, 24.1, 23.7, 23.3 ppm; IR ν_{max} 2922, 2854, 1663, 1438, 1378, 1248, 1209, 873 cm^{-1}; HRMS–DART (*m*/*z*): $[M+H]^+$ calcd for $C_{11}H_{17}O$, 165.1279; found, 165.1274.

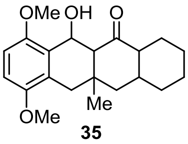

35

***β*-hydroxyl ketone 35** (51.3 mg, dr = 1:1.1:1) was prepared according to general procedure C from **7** (1.2 mmol) and **18** (0.3 mmol) in 49% yield. The reaction time was 30 min under λ_{max} = 366 nm light. The products were isolated by silica gel column chromatography (3% to 8% ethyl acetate-petroleum ether) for the first time and then **35a** was purified by preparative thin layer

chromatography (10% ethyl acetate-hexane) to give a white powder (15.5 mg, 15%), **35bc** was purified by preparative thin layer chromatography (15% acetone-hexane) to give an inseparable mixture as colorless oils (35.8 mg, 34%).

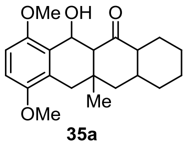

35a

***β*-hydroxyl ketone 35a:** R_f = 0.52 (20% ethyl acetate-petroleum ether); m.p. 155 – 157 °C; ^{1}H NMR (400 MHz, $CDCl_3$) δ 6.74 (d, J = 8.9 Hz, 1H), 6.69 (d, J = 8.9 Hz, 1H), 5.19 (dd, J = 6.1, 2.3 Hz, 1H), 3.81 (s, 3H), 3.79 (s, 3H), 3.04 (d, J = 2.2 Hz, 1H), 2.88 (d, J = 17.6 Hz, 1H), 2.61 (td, J = 11.1, 2.9 Hz, 1H), 2.43 (d, J = 17.7 Hz, 1H), 2.40 (dd, J = 6.3, 1.9 Hz, 1H), 2.22 – 2.15 (m, 1H), 1.98 (t, J = 12.7 Hz, 1H), 1.86 – 1.77 (m, 1H), 1.74 – 1.58 (m, 3H), 1.33 – 1.20 (m, 3H), 1.20 – 1.09 (m, 2H), 1.06 (s, 3H) ppm; ^{13}C NMR (100 MHz, $CDCl_3$) δ 214.0, 151.2, 151.0, 126.9, 124.9, 109.5, 107.5, 63.4, 58.7, 55.8 (d, J = 2.0 Hz), 55.6 (d, J = 2.0 Hz), 55.4, 41.4, 39.1, 36.8, 34.4, 33.0, 29.9, 26.3, 26.2, 25.6 ppm; IR ν_{max} 3527, 2919, 2849, 1689, 1480, 1258, 1079, 805, 715 cm^{-1}; HRMS–ESI (*m*/*z*): $[M+Na]^+$ calcd for $C_{21}H_{28}O_4Na$, 367.1885; found, 367.1879.

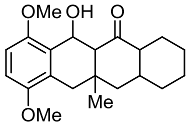

35b, 35c

***β*-hydroxyl ketone 35b, 35c:** R_f = 0.20 (20% ethyl acetate-petroleum ether); ^{1}H NMR (400 MHz, $CDCl_3$) δ 6.73 – 6.63 (m, 2H), 5.18 – 5.09 (m, 1H), 4.53 (d, J = 5.5 Hz, 1H, major), 4.44 (d, J = 6.4 Hz, 1H, minor), 3.84 (s, 3H, major), 3.83 (s, 3H, minor), 3.724 (s, 3H, major), 3.720 (s, 3H, minor), 3.20 (dd, J = 6.0, 1.9 Hz, 1H, minor), 3.05 (d, J = 6.1 Hz, 1H, major), 2.63 – 2.28 (m, 3H), 2.23 – 2.05 (m, 1H), 1.85 – 1.66 (m, 4H), 1.66 – 1.60 (m, 1H), 1.59 – 1.50 (m, 2H), 1.38 – 1.14 (m, 3H), 1.04 (s, 3H, minor), 1.03 (s, 3H, major). ^{13}C NMR (100 MHz, $CDCl_3$) δ 215.8, 212.5, 152.3, 152.1, 151.2 (2C), 126.7, 126.5, 125.3, 125.1, 108.8, 108.63, 108.59, 108.3, 66.1, 66.0, 57.9, 56.0 (d, J = 2.2 Hz), 55.9 (d, J = 2.2 Hz), 55.52, 55.5 (d, J = 2.0 Hz), 55.4 (d, J = 2.0 Hz), 53.8, 52.8, 46.5, 41.6, 40.0, 39.1, 39.0, 34.2, 32.4, 30.9, 30.5, 29.7, 27.4, 27.1, 25.7, 25.4, 25.2, 25.1, 24.6, 20.6 ppm; IR ν_{max} 3378, 2924, 2846, 1684, 1479, 1251, 1082, 1042, 791, 725 cm^{-1}; HRMS–ESI (*m*/*z*): $[M+Na]^+$ calcd for $C_{21}H_{28}O_4Na$, 367.1885; found, 367.1879.

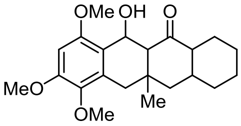

36

***β*-hydroxyl ketone 36** (58.7 mg, dr = 1.0:1.25:1.1) was prepared according to general procedure C from **13** (1.2 mmol) and **18** (0.3 mmol) in 52% yield. The reaction time was 1 h under λ_{max} = 366 nm light. The products were isolated through silica gel column chromatography (5% to 16% ethyl acetate-petroleum ether) for the first time and then **36a** was purified by preparative thin layer chromatography (10% acetone-hexane) to give a white solid (17.8 mg, 15.8%), **36b** was purified by preparative thin layer chromatography (25% ethyl acetate-hexane) to give a sandy beige oil (21.0 mg, 18.5%), **36c** was purified by preparative thin layer chromatography (25% ethyl acetate-hexane) to give a sandy beige oil (19.9 mg, 17.5%).

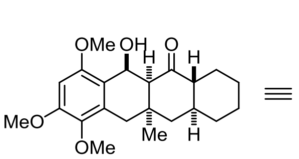

36a

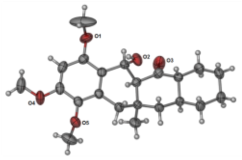

CCDC 1502442

***β*-hydroxyl ketone 36a:** R_f = 0.68 (40% ethyl acetate-petroleum ether); m.p. 108 – 110 °C; Recrystallization of **36a** from dichloromethane, CCDC 1502442. ^{1}H NMR (400 MHz, $CDCl_3$) δ 6.39 (s, 1H), 5.22 (dd, J = 5.4, 1.7 Hz, 1H), 3.88 (s, 3H), 3.82 (s, 3H), 3.73 (s, 3H), 2.89 (d, J = 16.7 Hz, 1H), 2.65 (s, 1H), 2.60 (d, J = 16.7 Hz, 1H), 2.48 (td, J = 11.2, 3.0 Hz, 1H), 2.27 (dd, J = 5.4, 1.7 Hz, 1H), 2.20 – 2.13 (m, 1H), 1.93 (t, J = 12.7 Hz, 1H), 1.85 – 1.78 (m, 1H), 1.73 – 1.55 (m, 3H), 1.40 (ddd, J = 12.9, 3.1, 1.9 Hz, 1H), 1.27 – 1.19 (m, 2H), 1.18 – 1.07 (m, 2H), 1.04 (s, 3H) ppm; ^{13}C NMR (100 MHz, $CDCl_3$) δ 214.2, 153.2, 152.7, 140.2, 130.1, 118.9, 94.6, 64.0, 60.5 (d, J = 2.0 Hz), 59.2, 56.0 (d, J = 2.2 Hz), 55.7 (d, J = 2.1 Hz), 55.4, 42.9, 38.5, 37.0, 34.4, 33.2, 30.2, 26.14, 26.06, 25.6 ppm; IR ν_{max} 3450, 2921, 2853, 1689, 1598, 1486, 1453, 1326, 1232, 1201, 1071, 983, 830 cm^{-1}; HRMS–ESI (*m*/*z*): $[M+Na]^+$ calcd for $C_{22}H_{30}O_5Na$, 397.1991; found, 397.1988.

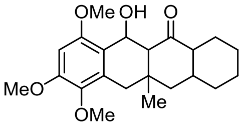

36b

***β*-hydroxyl ketone 36b:** R_f = 0.44 (40% ethyl acetate-petroleum ether); ^{1}H NMR (400 MHz, $CDCl_3$) δ 6.41 (s, 1H), 5.12 (t, J = 6.4 Hz, 1H), 4.05 (d, J = 7.3 Hz, 1H), 3.86 (s, 6H), 3.68 (s, 3H), 3.10 (dd, J = 6.0, 1.8 Hz, 1H), 2.68 (d, J = 16.8 Hz, 1H), 2.51 – 2.37 (m, 3H), 2.17 (t, J = 13.6 Hz, 1H), 1.85 – 1.75 (m, 2H), 1.75 – 1.66 (m, 2H), 1.62 – 1.50 (m, 3H), 1.36 (dd, J = 13.9, 3.9 Hz, 1H), 1.27 – 1.18 (m, 1H), 1.00 (s, 3H) ppm; ^{13}C NMR (100 MHz, $CDCl_3$) δ 217.0, 154.7, 151.9, 140.3, 130.7, 118.2, 95.8, 65.8, 60.3 (d, J = 1.8 Hz), 56.1 (d, J = 2.0 Hz), 55.8 (d, J = 2.0 Hz), 53.5, 53.4, 39.1, 38.9, 32.1, 30.8, 29.8, 27.6, 25.4, 25.2, 20.7 ppm; IR ν_{max} 3459, 2925, 2852, 1687, 1598, 1489, 1459, 1328, 1233, 1203, 1084, 911, 729 cm^{-1}; HRMS–DART (*m*/*z*): $[M+H]^+$ calcd for $C_{22}H_{31}O_5$, 375.2171; found, 375.2169.

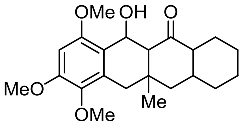

36c

***β*-hydroxyl ketone 36c:** R_f = 0.38 (40% ethyl acetate-petroleum ether); ^{1}H NMR (400 MHz, $CDCl_3$) δ 6.42 (s, 1H), 5.06 (t, J = 6.4 Hz, 1H), 4.28 (d, J = 7.1 Hz, 1H), 3.87 (s, 3H), 3.85 (s, 3H), 3.69 (s, 3H), 3.04 (d, J = 6.3 Hz, 1H), 2.55 (d, J = 17.2 Hz, 1H), 2.42 (dd, J = 17.1, 1.8 Hz, 1H), 2.13 – 2.05 (m, 1H), 1.81 – 1.70 (m, 5H), 1.68 – 1.61 (m, 2H), 1.36 – 1.14 (m, 4H), 1.00 (s, 3H).ppm; ^{13}C NMR (100 MHz, $CDCl_3$) δ 213.5, 154.8, 151.7, 140.2, 130.4, 118.1, 95.9, 65.8, 60.2 (d, J = 1.8 Hz), 57.9, 56.1 (d, J = 1.9 Hz), 55.9 (d, J = 1.9 Hz), 55.5, 46.5, 41.4, 40.3, 34.2, 30.9, 26.7, 25.7, 25.1, 24.6 ppm; IR ν_{max} 3546, 2924, 2849, 1709, 1597, 1486, 1449, 1322, 1233, 1203, 1087, 1028, 828, 803, 730 cm^{-1}; HRMS–DART (*m*/*z*): $[M+H]^+$ calcd for $C_{22}H_{31}O_5$, 375.2171; found, 375.2170.

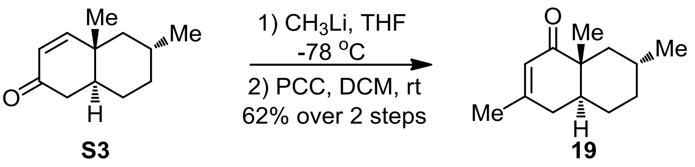

Supplementary Figure 93. Synthesis of enone **19**.

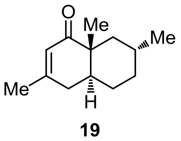

Enone 19: To a stirred solution of **S3**[7] (300 mg, 1.68 mmol, 1.0 qeuiv.) in anhydrous tetrahydrofuran (15 mL) was added methyllithium (1.3 M solution in diethyl ether, 1.9 mL, 2.52 mmol, 1.5 equiv.) dropwise at -78 ^{o}C under N_2, the resulting mixture was stirred at -78 ^{o}C until TLC showed **S3** was consumed completely. Then the reaction was quenched with saturated ammonium chloride (10 mL), the mixture was extracted with ethyl acetate (3×10 mL), the combined organic layer was washed with brine (2×15 mL) and dried over anhydrous sodium sulfate. The dried solution was filtered and the filtrate was concentrated under vacuum. The residue was drained completely with oil pump and dissolved in dichloromethane (10 mL), then pyridinium chlorochromate (1.45 g, 6.72 mmol, 4.0 equiv.) was added to the above mixture, the resulting mixture was stirred at rt until TLC showed no material was remaining. The reaction mixture was filtered through a short pad of column chromatography and rinsed with ethyl acetate, the filtrate was concentrated under vacuum. The residue was purified by silica gel column chromatography (0% to 5% ethyl acetate-petroleum ether) to give **19** as a light yellow oil (203 mg, 62% over 2 steps, dr = 3.2:1). R_f = 0.7 (10% ethyl acetate-petroleum ether); ^{1}H NMR (500 MHz, $CDCl_3$) δ5.81 (s, 1H, minor), 5.74 (s, 1H, major), 2.14 – 1.97 (m, 2H), 1.93 (s, 3H, minor), 1.90 (s, 3H, major), 1.82 – 1.72 (m, 1H), 1.71 – 1.55 (s, 3H), 1.53 – 1.38 (m, 2H), 1.20 (s, 3H, minor), 1.19 – 1.03 (m, 1H), 0.98 (s, 3H, major), 0.96 – 0.81 (m, 1H), 0.90 (d, J = 6.5 Hz, 3H, major), 0.85 (d, J = 6.5 Hz, 3H, minor) ppm; major isomer: ^{13}C NMR (125 MHz, $CDCl_3$) δ 205.8, 159.4, 124.7, 43.8, 41.1, 41.0, 35.5, 34.7, 27.9, 27.6, 23.9, 22.8, 15.2 ppm; minor isomer: ^{13}C NMR (125 MHz, $CDCl_3$) δ 204.7, 160.1, 124.8, 44.3, 38.3, 37.9, 33.0, 29.4, 26.5, 26.2, 24.2, 22.6, 20.4 ppm; IR ν_{max} 2917, 2869, 1663, 1443, 1377, 1260, 859 cm^{-1}; HRMS–DART (*m/z*): $[M+H]^+$ calcd for $C_{13}H_{21}O$, 193.1592; found, 193.1587.

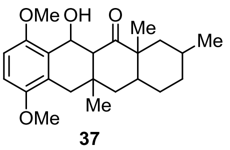

***β*-hydroxyl ketone 37** (54.4 mg, dr = 3.2:1) was prepared according to general procedure C from **7** (1.2 mmol) and **19** (0.3 mmol) in 48% yield. The reaction time was 30 min under λ_{max} = 366 nm light. The products were isolated through silica gel column chromatography (5% to 12% ethyl acetate-petroleum ether) for the first time and then **37a** was purified by preparative thin layer chromatography (20% acetone-hexane) to give a colorless oil (45 mg, 40%), **37b** was purified by preparative thin layer chromatography (15% acetone-hexane) to give a colorless oil (9.4 mg, 8%).

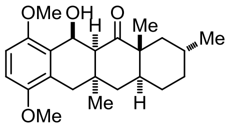
37a

***β*-hydroxyl ketone 37a:** R_f = 0.28 (20% ethyl acetate-petroleum ether); ^{1}H NMR (500 MHz, $CDCl_3$) δ 6.71 (d, J = 8.9 Hz, 1H), 6.66 (d, J = 8.9 Hz, 1H), 5.15 (t, J = 5.5 Hz, 1H), 4.49 (d, J = 6.0 Hz, 1H), 3.84 (s, 3H), 3.74 (s, 3H), 3.40 (dd, J = 6.0, 1.7 Hz, 1H), 2.57 (d, J = 17.5 Hz, 1H), 2.28 (dd, J = 17.5, 1.3 Hz, 1H), 1.82 – 1.75 (m, 2H), 1.73 – 1.58 (m, 3H), 1.49 – 1.42 (m, 2H), 1.41 – 1.34 (m, 2H), 1.27 – 1.17 (m, 1H), 1.22 (s, 3H), 1.03 (s, 3H), 0.87 (d, J = 6.4 Hz, 3H) ppm; ^{13}C NMR (125 MHz, $CDCl_3$) δ 215.9, 152.2, 151.1, 126.6, 125.2, 108.7, 108.3, 66.0, 56.0, 55.4, 52.6, 49.0, 42.2, 41.5, 40.1, 39.5, 35.0, 30.6, 27.6, 27.4, 27.3, 22.8, 16.1 ppm; IR ν_{max} 3510, 2918, 1688, 1478, 1251, 1083, 1038, 797, 712 cm^{-1}; HRMS–ESI (*m*/*z*): $[M+Na]^+$ calcd for $C_{23}H_{32}O_4Na$, 395.2198; found, 395.2191.

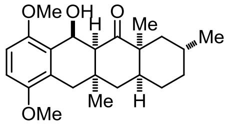
37b

***β*-hydroxyl ketone 37b:** R_f = 0.34 (20% ethyl acetate-petroleum ether); ^{1}H NMR (400 MHz, $CDCl_3$) δ 6.72 (d, J = 8.9 Hz, 1H), 6.66 (d, J = 8.8 Hz, 1H), 5.15 (t, J = 6.0 Hz, 1H), 4.47 (d, J = 6.8 Hz, 1H), 3.84 (s, 3H), 3.73 (s, 3H), 3.32 (dd, J = 5.7, 1.9 Hz, 1H), 2.45 (d, J = 17.4 Hz, 1H), 2.31 (dd, J = 17.4, 1.7 Hz, 1H), 2.18 (t, J = 13.7 Hz, 1H), 2.05 – 1.97 (m, 1H), 1.91 – 1.80 (m, 1H), 1.69 – 1.53 (s, 3H), 1.46 – 1.39 (m, 1H), 1.35 (dd, J = 13.9, 4.6 Hz, 1H), 1.32 – 1.23 (m, 2H), 1.13 (s, 3H), 1.02 (s, 3H), 0.92 (d, J = 5.7 Hz, 3H).ppm; ^{13}C NMR (100 MHz, $CDCl_3$) δ 216.7, 152.4, 151.2, 127.2, 125.2, 109.1, 108.6, 66.3, 56.2 (d, J = 1.9 Hz), 55.5 (d, J = 1.9 Hz), 52.4, 49.8, 40.4, 39.4, 38.6, 37.4, 30.4, 29.4, 27.6, 27.3, 26.9, 22.9, 20.2 ppm; IR ν_{max} 3503, 2920, 1685, 1472, 1250, 1080, 797, 722 cm^{-1}; HRMS–DART (*m*/*z*): $[M+NH_4]^+$ calcd for $C_{23}H_{36}O_4N$, 390.2644; found, 390.2640.

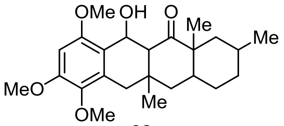
38

***β*-hydroxyl ketone 38** (75 mg, dr = 2.8:1) was prepared according to general procedure C from **13** (1.2 mmol) and **19** (0.3 mmol) in 62% yield. The reaction time was 45 min under λ_{max} = 366 nm light. The products were isolated through silica gel column chromatography (8% to 12% ethyl acetate-petroleum ether) to give **38a** (59 mg, 49%) and **38b** (16 mg, 13%) as colorless oils:

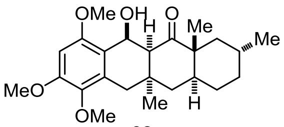
38a

***β*-hydroxyl ketone 38a:** R_f = 0.43 (30% ethyl acetate-petroleum ether); ^{1}H NMR (400 MHz, $CDCl_3$) δ 6.41 (s, 1H), 5.12 (t, J = 6.4 Hz, 1H), 4.08 (d, J = 7.2 Hz, 1H), 3.86 (s, 6H), 3.70 (s, 3H), 3.29 (dd, J = 6.1, 1.7 Hz, 1H), 2.65 (d, J = 16.9 Hz, 1H), 2.36 (dd, J = 16.9, 1.5 Hz, 1H), 1.80 – 1.57 (m, 5H), 1.55 – 1.47 (m, 1H), 1.46 – 1.34 (m, 3H), 1.26 – 1.14 (m, 1H), 1.19 (s, 3H), 0.98 (s, 3H), 0.88 (d, J = 6.3 Hz, 3H) ppm; ^{13}C NMR (100 MHz, $CDCl_3$) δ 217.1, 154.6, 151.8, 140.2, 130.8, 118.2, 95.9, 65.7, 60.3 (d, J = 1.8 Hz), 56.2 (d, J = 2.0 Hz), 55.9 (d, J = 2.0 Hz), 53.1, 48.8, 41.5 (2C), 40.4, 39.3, 34.9, 31.0, 27.6, 27.5, 27.3, 22.7, 16.2 ppm; IR ν_{max} 3479, 2921, 2855,

1691, 1597, 1489, 1458, 1326, 1233, 1203, 1089, 983, 812, 734 cm^{-1}; HRMS–EI (*m*/*z*): $[M]^+$ calcd for $C_{24}H_{34}O_5$, 402.2406; found, 402.2410.

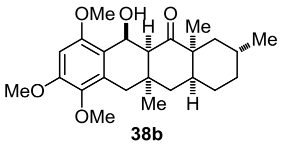

38b

***β*-hydroxyl ketone 38b:** R_f = 0.49 (30% ethyl acetate-petroleum ether); ^1H NMR (400 MHz, $CDCl_3$) δ 6.41 (s, 1H), 5.12 (t, J = 6.5 Hz, 1H), 4.00 (d, J = 7.5 Hz, 1H), 3.85 (s, 6H), 3.68 (s, 3H), 3.16 (dd, J = 5.8, 1.7 Hz, 1H), 2.57 (d, J = 16.8 Hz, 1H), 2.40 (dd, J = 16.7, 1.6 Hz, 1H), 2.15 (t, J = 13.6 Hz, 1H), 2.06 – 1.97 (m, 1H), 1.92 – 1.80 (m, 1H), 1.63 – 1.52 (m, 2H), 1.44 (d, J = 14.1 Hz, 1H), 1.38 – 1.20 (m, 4H), 1.15 (s, 3H), 0.97 (s, 3H), 0.92 (d, J = 6.0 Hz, 3H) ppm; ^{13}C NMR (100 MHz, $CDCl_3$) δ 218.1, 154.7, 151.9, 140.3, 130.8, 118.6, 96.0, 66.0, 60.2 (d, J = 1.9 Hz), 56.3 (d, J = 2.3 Hz), 55.9 (d, J= 2.3 Hz), 53.2, 49.4, 40.3, 39.4, 38.0, 36.7, 30.7, 29.4, 27.6 (2C), 27.0, 22.8, 20.5 ppm; IR ν_{max} 3461, 2924, 1684, 1598, 1489, 1459, 1330, 1232, 1202, 1082, 1048, 812, 732 cm^{-1}; HRMS–EI (*m*/*z*): $[M]^+$ calcd for $C_{24}H_{34}O_5$, 402.2406; found, 402.2408.

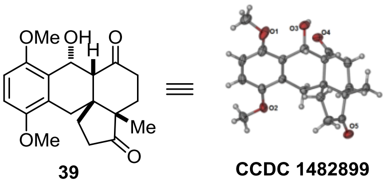

39 CCDC 1482899

***β*-hydroxyl ketone 39** (33.4 mg) was prepared according to general procedure C from **7** (1.2 mmol) and **20** (0.3 mmol) in 32% yield. The reaction time was 30 min under λ_{max} = 366 nm light. The products were isolated through silica gel column chromatography (20% to 50% ethyl acetate-petroleum ether) as a white solid: R_f = 0.19 (50% ethyl acetate-petroleum ether); m.p. 175 – 177 ^oC; Recrystallization of **39** from dichloromethane/hexane (v/v, 1:1), CCDC 1482899. $[\alpha]^{27}_D$ = -24.1 (c = 0.45, $CHCl_3$); ^1H NMR (400 MHz, $CDCl_3$) δ 6.73 (d, J = 8.9 Hz, 1H), 6.65 (d, J = 8.9 Hz, 1H), 5.03 (t, J = 6.0 Hz, 1H), 4.76 (d, J = 6.7 Hz, 1H), 3.84 (s, 3H), 3.68 (s, 3H), 3.33 (dd, J = 5.7, 3.0 Hz, 1H), 2.59 – 2.48 (m, 2H), 2.47 – 2.36 (m, 2H), 2.31 – 2.22 (m, 2H), 2.22 – 2.11 (m, 1H), 2.04 – 1.93 (m, 1H), 1.85 (dd, J = 14.0, 7.1 Hz, 1H), 1.62 – 1.52 (m, 1H), 1.43 (s, 3H) ppm; ^{13}C NMR (100 MHz, $CDCl_3$) δ 216.6, 210.7, 152.2, 151.2, 126.8, 123.1, 109.2, 108.7, 66.4, 55.9, 55.3, 52.4, 51.5, 47.4, 37.3, 33.4, 27.0, 26.0, 25.2, 17.7 ppm; IR ν_{max} 3485, 2926, 1737, 1694, 1471, 1432, 1251, 1081, 1048, 1016, 800, 717 cm^{-1}; HRMS–EI (*m*/*z*): $[M]^+$ calcd for $C_{20}H_{24}O_5$, 334.1624; found, 334.1626.

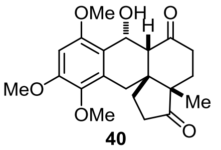

40

***β*-hydroxyl ketone 40** (27.8 mg) was prepared according to general procedure C from **13** (1.2 mmol) and **20** (0.3 mmol) in 24% yield. The reaction time was 1 h under λ_{max} = 366 nm light. The products were isolated through silica gel column chromatography (30% to 50% ethyl acetate-petroleum ether) as white solid: R_f = 0.18 (50% ethyl acetate-petroleum ether); $[\alpha]^{27}_D$ = -37.1 (c = 0.15, $CHCl_3$); ^1H NMR (400 MHz, $CDCl_3$) δ 6.44 (s, 1H), 5.00 (t, J = 6.5 Hz, 1H), 4.61 (d, J = 7.8 Hz, 1H), 3.88 (s, 3H), 3.86 (s, 3H), 3.64 (s, 3H), 3.32 (dd, J = 6.0, 3.0 Hz, 1H), 2.61 – 2.41 (m, 4H), 2.33 (d, J = 17.4 Hz, 1H), 2.29 – 2.10 (m, 2H), 2.02 – 1.91(m, 1H), 1.88 (dd, J = 14.0, 7.0 Hz, 1H), 1.54 (dd, J = 12.6,

8.7 Hz, 1H), 1.42 (s, 3H) ppm; ^{13}C NMR (100 MHz, $CDCl_3$) δ 216.4, 212.0, 155.1, 152.2, 140.4, 128.4, 118.1, 96.2, 66.2, 60.4 (d, J = 1.6 Hz), 56.1 (d, J = 2.0 Hz), 55.9 (d, J = 2.0 Hz), 52.6, 51.5, 47.8, 37.4, 33.4, 27.0, 25.8, 24.9, 17.8 ppm; IR ν_{max} 3473, 2938, 1737, 1692, 1595, 1462, 1327, 1234, 1204, 1077, 1011, 808, 728 cm^{-1}; HRMS–EI (*m*/*z*): $[M]^+$ calcd for $C_{21}H_{26}O_6$, 374.1729; found, 374.1731.

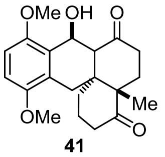

41

***β*-hydroxyl ketone 41** (65.4 mg) was prepared according to general procedure C from **7** (0.6 mmol) and **21** (0.3 mmol) in 61% yield. The reaction time was 30 min under λ_{max} = 366 nm light. The products were isolated through silica gel column chromatography (15% to 25% ethyl acetate-petroleum ether) as light yellow foam which was easy to eliminate: R_f = 0.19 (30% ethyl acetate-petroleum ether); m.p. 142 – 144 °C; ^{1}H NMR (500 MHz, $CDCl_3$) δ 6.71 (d, J = 9.0 Hz, 1H), 6.67 (d, J = 8.9 Hz, 1H), 4.98 (t, J = 4.5 Hz, 1H), 4.36 (d, J = 5.8 Hz, 1H), 3.82 (s, 3H), 3.73 (s, 3H), 3.03 (d, J = 4.2 Hz, 1H), 2.75 – 2.63 (m, 3H), 2.55 (ddd, J = 13.8, 7.1, 1.7 Hz, 1H), 2.48 – 2.38 (m, 2H), 2.24 – 2.16 (m, 1H), 1.98 – 1.83 (m, 2H), 1.81 – 1.70 (m, 2H), 1.63 (d, J = 14.8 Hz, 1H), 1.25 (s, 3H) ppm; ^{13}C NMR (125 MHz, $CDCl_3$) δ 213.5, 212.0, 151.9, 151.0, 126.2, 123.9, 108.8, 108.6, 65.0, 55.8, 55.4, 52.7, 51.9, 48.2, 40.0, 37.2, 31.5, 28.9, 28.3, 21.0, 20.6 ppm; IR ν_{max} 3540, 2948, 1718, 1703, 1484, 1460, 1443, 1257, 1071, 1047, 944, 821, 712 cm^{-1}; HRMS–EI (*m*/*z*): $[M]^+$ calcd for $C_{21}H_{26}O_5$, 358.1780; found, 358.1783.

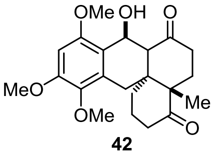

42

***β*-hydroxyl ketone 42** (62 mg) was prepared according to general procedure C from **13** (0.6 mmol) and **21** (0.3 mmol) in 53% yield. The reaction time was 30 min under λ_{max} = 366 nm light. The products were isolated through silica gel column chromatography (20% to 30% ethyl acetate-petroleum ether) as a light yellow solid which was easy to eliminate: R_f = 0.14 (40% ethyl acetate-petroleum ether); m.p. 179 – 181 °C; ^{1}H NMR (500 MHz, $CDCl_3$) δ 6.43 (s, 1H), 4.96 (t, J = 6.2 Hz, 1H), 4.09 (d, J = 7.1 Hz, 1H), 3.86 (s, 6H), 3.71 (s, 3H), 3.01 (d, J = 4.5 Hz, 1H), 2.77 (dd, J = 17.0, 2.1 Hz, 1H), 2.75 – 2.63 (m, 2H), 2.58 (ddd, J = 13.9, 7.2, 2.2 Hz, 1H), 2.50 (d, J = 16.9 Hz, 1H), 2.48 – 2.43 (m, 1H), 2.25 (ddd, J = 13.4, 5.4, 2.2 Hz, 1H), 2.00 – 1.82 (m, 2H), 1.80 – 1.69 (m, 2H), 1.62 (d, J = 14.6 Hz, 1H), 1.26 (s, 3H) ppm; ^{13}C NMR (125 MHz, $CDCl_3$) δ 213.5, 213.0, 154.6, 152.0, 140.3, 129.2, 117.6, 95.8, 64.6, 60.3, 56.0, 55.9, 52.7, 52.5, 48.4, 39.9, 37.2, 31.5, 28.5, 28.3, 21.0, 20.7 ppm; IR ν_{max} 3520, 3013, 1721, 1696, 1596, 1459, 1388, 1320, 1205, 1079, 1025, 807 cm^{-1}; HRMS–EI (*m*/*z*): $[M]^+$ calcd for $C_{22}H_{28}O_6$, 388.1886; found, 388.1884.

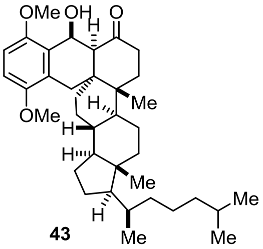

43

***β*-hydroxyl ketone 43** (113 mg) was prepared according to general procedure C from **7** (1.2 mmol) and **22** (0.3 mmol) in 67% yield. The reaction time was 30 min under

λ_{max} = 366 nm light. The products were isolated through silica gel column chromatography (10% to 20% ethyl acetate-petroleum ether) as a white solid: R_f = 0.38 (20% ethyl acetate-petroleum ether); m.p. 174 – 176 °C; $[\alpha]^{27}_{D}$ = +53.5 (c = 0.45, $CHCl_3$); ^{1}H NMR (400 MHz, $CDCl_3$) δ 6.70 (d, J = 8.9 Hz, 1H), 6.66 (d, J = 8.9 Hz, 1H), 4.99 (t, J = 5.7 Hz, 1H), 4.34 (d, J = 6.3 Hz, 1H), 3.83 (s, 3H), 3.74 (s, 3H), 3.70 (d, J = 5.3 Hz, 1H), 2.60 – 2.43 (m, 3H), 2.18 – 1.99 (m, 3H), 1.97 – 1.79 (m, 3H), 1.60 – 1.03 (m, 21H), 1.00 (s, 3H), 0.93 (d, J = 6.5 Hz, 3H), 0.88 (d, J = 1.7 Hz, 3H), 0.86 (d, J = 1.7 Hz, 3H), 0.69 (s, 3H) ppm; ^{13}C NMR (100 MHz, $CDCl_3$) δ 213.5, 151.9, 151.3, 126.3, 125.2, 108.5, 108.4, 65.6, 56.7, 56.2, 55.8, 55.4, 50.3, 46.5, 42.5, 42.4, 40.3, 39.4, 39.2, 38.9, 36.1, 35.8, 35.6, 33.4, 29.7, 29.3, 28.3, 28.0, 26.5, 24.1, 23.8, 22.8, 22.5, 21.9, 18.6, 16.5, 12.0 ppm; IR ν_{max} 3525, 2934, 1717, 1462, 1253, 1080, 797, 726 cm^{-1}; HRMS–EI (*m*/*z*): $[M]^+$ calcd for $C_{37}H_{56}O_4$, 564.4179; found, 564.4181.

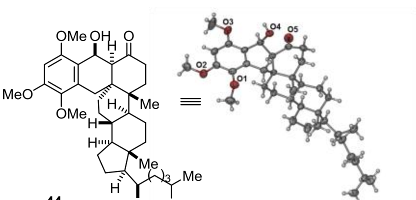

44 CCDC 1502440

***β*-hydroxyl ketone 44** (105 mg) was prepared according to general procedure C from **13** (1.2 mmol) and **22** (0.3 mmol) in 59% yield. The reaction time was 30 min under λ_{max} = 366 nm light. The products were isolated through silica gel column chromatography (10% to 20% ethyl acetate-petroleum ether) as white solid: R_f = 0.28 (40% ethyl acetate-petroleum ether); m.p. 186 – 188 °C; Recrystallization of **44** from dichloromethane/ethyl acetate (v/v, 1:1), CCDC 1502440. $[\alpha]^{27}_{D}$ = 74.9 (c = 0.45, $CHCl_3$); ^{1}H NMR (500 MHz, $CDCl_3$) δ 6.40 (s, 1H), 4.97 (t, J = 5.8 Hz, 1H), 3.95 (d, J = 6.7 Hz, 1H), 3.85 (s, 6H), 3.69 (s, 3H), 3.59 (d, J = 5.6 Hz, 1H), 2.61 (s, 2H), 2.51 (td, J = 13.8, 6.6 Hz, 1H), 2.19 – 2.13 (m, 1H), 2.11 – 1.99 (m, 2H), 1.94 (dd, J = 14.4, 4.9 Hz, 1H), 1.91 – 1.77 (m, 2H), 1.59 – 1.42 (m, 6H), 1.40 – 1.22 (m, 8H), 1.18 – 1.04 (m, 7H), 1.01 (s, 3H), 0.92 (d, J = 6.5 Hz, 3H), 0.87 (d, J = 2.4 Hz, 3H), 0.86 (d, J = 2.4 Hz, 3H), 0.68 (s, 3H) ppm; ^{13}C NMR (125 MHz, $CDCl_3$) δ 214.7, 154.4, 151.9, 140.5, 130.6, 117.9, 95.5, 65.4, 60.2, 56.7, 56.2, 56.0, 55.9, 50.9, 46.4, 42.7, 42.4, 40.2, 39.5, 39.1, 38.7, 36.1, 35.8, 35.5, 32.9, 29.5, 29.4, 28.3, 28.0, 26.7, 24.1, 23.9, 22.8, 22.5, 21.8, 18.6, 16.6, 12.0 ppm; IR ν_{max} 3494, 2919, 2855, 1690, 1600, 1493, 1464, 1330, 1234, 1207, 1116, 1087, 998, 816, 745 cm^{-1}; HRMS–EI (*m*/*z*): $[M]^+$ calcd for $C_{38}H_{58}O_5$, 594.4284; found, 594.4288.

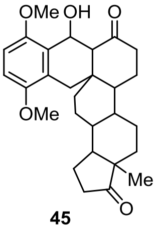

45

***β*-hydroxyl ketone 45** (104 mg, dr = 2.1:1) was prepared according to general procedure C from **7** (1.2 mmol) and **23** (0.3 mmol) in 76% yield. The reaction time was 30 min under λ_{max} = 366 light. The products were isolated through silica gel column chromatography (20% to 50% ethyl acetate-petroleum ether) to give a mixture of **45a** and **45b** as a white solid:

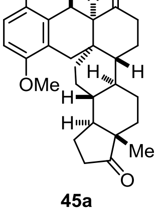

45a

***β*-hydroxyl ketone 45a:** R_f = 0.17 (30% ethyl acetate-petroleum ether); m.p. 112 – 114 °C; $[\alpha]^{27}_{D}$ = 118.7 (c = 0.15, $CHCl_3$); ^{1}H NMR (500 MHz, $CDCl_3$) δ 6.72 (d, J = 8.9 Hz, 1H), 6.69

(d, J = 8.9 Hz, 1H), 5.03 (t, J = 5.3 Hz, 1H), 4.35 (d, J = 6.4 Hz, 1H), 3.84 (s, 3H), 3.74 (s, 3H), 3.42 (d, J = 4.4 Hz, 1H), 2.81 (d, J = 17.2 Hz, 1H), 2.51 – 2.39 (m, 2H), 2.37 (dd, J = 17.3, 2.0 Hz, 1H), 2.29 – 2.08 (m, 4H), 1.99 – 1.87 (m, 3H), 1.76 (dt, J = 14.1, 2.8 Hz, 1H), 1.68 – 1.34 (m, 7H), 1.31 – 1.18 (m, 2H), 1.10 (td, J = 13.8, 3.1 Hz, 1H), 0.91 (s, 3H) ppm; ^{13}C NMR (125 MHz, $CDCl_3$) δ 220.8, 213.6, 152.2, 151.1, 126.4, 125.2, 108.9, 108.7, 65.5, 55.9, 55.6, 51.2, 50.6, 47.7, 45.4, 44.2, 41.2, 40.4, 38.0, 35.9, 34.5, 33.6, 31.7, 26.0, 25.0, 24.0, 21.6, 13.9 ppm; IR ν_{max} 3505, 2916, 2859, 1731, 1692, 1475, 1404, 1252, 1077, 1023, 912, 789, 723 cm^{-1}; HRMS–ESI (*m*/*z*): [M+Na]$^+$ calcd for $C_{28}H_{36}O_5Na$, 475.2460; found, 475.2454.

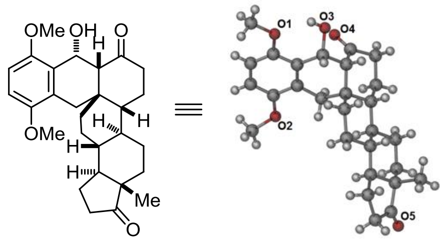

45b CCDC 1502441

***β*-hydroxyl ketone 45b:** R$_f$ = 0.14 (30% ethyl acetate-petroleum ether); m.p. 217 – 219 ^{o}C; Recrystallization of **45b** from dichloromethane/hexane (v/v, 1:2), CCDC 1502441. [α]$^{27}_{D}$ = -14.4 (c = 0.15, $CHCl_3$); ^{1}H NMR (500 MHz, $CDCl_3$) δ 6.72 (d, J = 8.9 Hz, 1H), 6.67 (d, J = 8.9 Hz, 1H), 5.10 (t, J = 5.1 Hz, 1H), 4.48 (d, J = 6.1 Hz, 1H), 3.84 (s, 3H), 3.74 (s, 3H), 2.99 (dd, J = 5.5, 1.0 Hz, 1H), 2.81 (dd, J = 17.8, 1.9 Hz, 1H), 2.50 – 2.34 (m, 3H), 2.25 – 2.15 (m, 2H), 2.11 – 2.01 (m, 1H), 1.92 – 1.67 (m, 6H), 1.59 – 1.45 (m, 2H), 1.44 – 1.30 (m, 4H), 1.28 – 1.07 (m, 3H), 0.91 (s, 3H) ppm; ^{13}C NMR (125 MHz, $CDCl_3$) δ 220.9, 211.9, 152.1, 151.4, 126.8, 124.3, 108.7, 108.6, 65.7, 59.3, 55.9, 55.5, 50.2, 49.0, 47.7, 43.6, 42.1, 41.8, 41.0, 35.8, 33.4, 31.5, 27.6, 25.5, 25.4, 22.8, 21.5, 13.8 ppm; IR ν_{max} 3543, 2927, 2842, 1727, 1461, 1248, 1072, 1037, 790, 714 cm^{-1}; HRMS–EI (*m*/*z*): [M]$^+$ calcd for $C_{28}H_{36}O_5$, 452.2563; found, 452.2565.

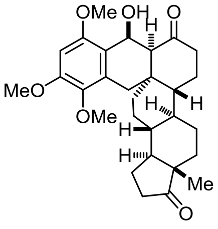

46a

***β*-hydroxyl ketone 46** (103 mg, dr = 7.1:1) was prepared according to general procedure C from **13** (1.2 mmol) and **23** (0.3 mmol) in 71% yield. The reaction time was 30 min under λ_{max} = 366 nm light. The products were isolated through silica gel column chromatography (30% to 40% ethyl acetate-petroleum ether) to give a mixture of **46** as white solid. Major isomer: R$_f$ = 0.16 (40% ethyl acetate-petroleum ether); m.p. 96 – 98 ^{o}C; [α]$^{27}_{D}$ = +102.2 (c = 0.15, $CHCl_3$); ^{1}H NMR (500 MHz, $CDCl_3$) δ 6.40 (s, 1H), 5.02 (t, J = 6.0 Hz, 1H), 3.89 (dd, J = 7.9, 1.2 Hz, 1H), 3.86 (s, 3H), 3.85 (s, 3H), 3.69 (s, 3H), 3.25 (d, J = 5.4 Hz, 1H), 2.93 (d, J = 16.5 Hz, 1H), 2.50 – 2.35 (m, 3H), 2.31 – 2.18 (m, 2H), 2.17 – 2.06 (m, 2H), 1.97 – 1.90 (m, 2H), 1.90 – 1.85 (m, 1H), 1.66 – 1.57 (m, 2H), 1.56 – 1.47 (m, 2H), 1.46 – 1.33 (m, 4H), 1.30 – 1.20 (m, 1H), 1.19 – 1.05 (m, 2H), 0.90 (s, 3H) ppm; ^{13}C NMR (125 MHz, $CDCl_3$) δ 220.7, 214.8, 154.5, 152.1, 140.1, 130.7, 117.7, 95.4, 65.3, 60.4, 56.0, 55.8, 51.9, 50.5, 47.7, 45.3, 43.7, 40.9, 40.5, 37.5, 35.8, 34.9, 33.8, 31.6, 25.9, 25.1, 23.2, 21.5, 13.8 ppm; IR ν_{max} 3534, 2919, 2859, 1733, 1692, 1597, 1459, 1329, 1234, 1204, 1082, 1013, 812, 751 cm^{-1}; HRMS–DART (*m*/*z*): [M+H]$^+$ calcd for $C_{29}H_{39}O_6$, 483.2747; found, 483.2738.

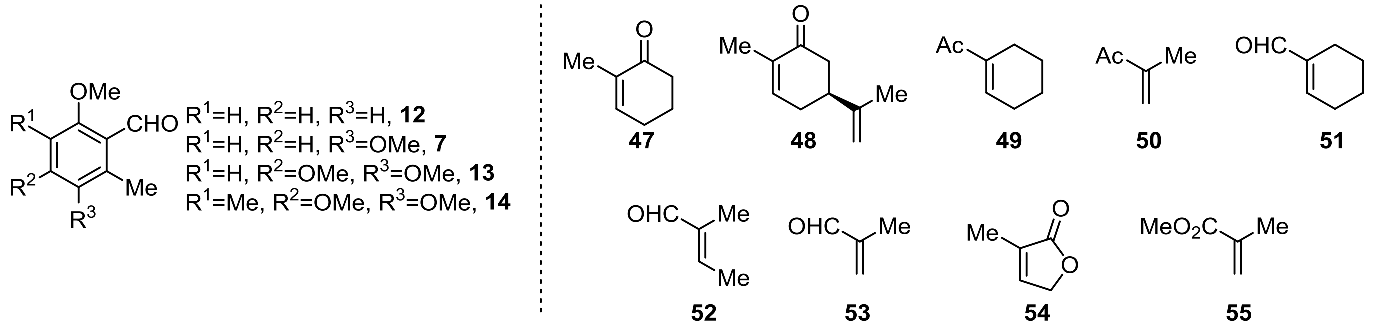

Supplementary Figure 94. Aromatic aldehyde and scope of the PEDA reaction involving 1,1-disubstituted olefins as dienophiles.

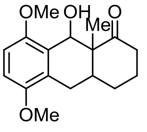
56

***β*-hydroxyl ketone 56** (58 mg, dr = 3:1) was prepared according to general procedure A from **7** (0.3 mmol) and **47** in 67% yield. The reaction time was 30 min under λ_{max} = 366 nm light. The products were isolated through silica gel column chromatography (2% to 5% ethyl acetate-petroleum ether) to give a mixture of **56a** and **56b** as white solid:

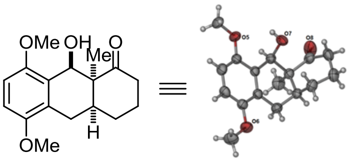
56a CCDC 1482890

***β*-hydroxyl ketone 56a:** R_f = 0.16 (10% ethyl acetate-petroleum ether); Recrystallization of **56a** from dichloromethane/hexane (v/v, 1:3), CCDC 1482890. ^{1}H NMR (400 MHz, $CDCl_3$) δ 6.74 (d, J = 8.8 Hz, 1H), 6.69 (d, J = 8.9 Hz, 1H), 4.69 (s, 1H), 3.81 (s, 3H), 3.80 (s, 3H), 2.96 (s, 1H), 2.88 – 2.71 (m, 3H), 2.53 (dddd, J = 16.4, 5.8, 3.1, 1.2 Hz, 1H), 2.19 (ddd, J = 24.6, 12.7, 4.4 Hz, 1H), 2.12 – 1.95 (m, 2H), 1.84 – 1.70 (m, 1H), 1.57 – 1.49 (m, 1H), 1.09 (s, 3H) ppm; ^{13}C NMR (100 MHz, $CDCl_3$) δ 215.4, 151.7, 151.4, 126.0, 123.8, 108.8, 107.2, 68.5, 55.6, 55.5, 50.3, 41.2, 38.9, 27.8, 26.4, 23.7, 20.9 ppm; IR ν_{max} 3525, 2926, 2862, 1694, 1460, 1255, 1079, 1014, 788, 710 cm^{-1}; HRMS–EI (*m*/*z*): $[M]^+$ calcd for $C_{17}H_{22}O_4$, 290.1518; found, 290.1519.

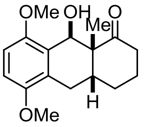
56b

***β*-hydroxyl ketone 56b:** R_f = 0.19 (10% ethyl acetate-petroleum ether); ^{1}H NMR (400 MHz, $CDCl_3$) δ 6.74 (d, J = 8.8 Hz, 1H), 6.69 (d, J = 8.9 Hz, 1H), 5.12 (d, J = 6.0 Hz, 1H), 3.82 (s, 3H), 3.79 (s, 3H), 3.07 (s, 1H), 2.99 – 2.86 (m, 2H), 2.79 – 2.73 (m, 1H), 2.66 (dd, J = 18.1, 7.2 Hz, 1H), 2.36 – 2.25 (m, 1H), 2.19 – 2.06 (m, 2H), 1.59 – 1.51 (m, 1H), 1.44 (qd, J = 13.0, 3.5 Hz, 1H), 1.11 (d, J = 6.7 Hz, 3H) ppm; ^{13}C NMR (100 MHz, $CDCl_3$) δ 214.7, 151.4, 151.3, 126.8, 124.6, 109.0, 107.3, 62.1, 55.7, 55.6, 52.4, 45.1, 34.3, 34.2, 28.2, 27.8, 15.3 ppm; IR ν_{max} 3405, 2929, 1674, 1597, 1479, 1256, 1168, 1087, 806, 715 cm^{-1}; HRMS–EI (*m*/*z*): $[M]^+$ calcd for $C_{17}H_{22}O_4$, 290.1518; found, 290.1522.

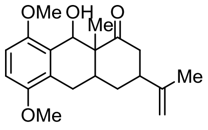
57

***β*-hydroxyl ketone 57** (82.5 mg, dr = 7:1) was prepared according to general procedure A from **7** (0.3 mmol) and **48** in 83% yield. The reaction time was 30 min under λ_{max} = 366 nm light. The products were isolated through silica gel column chromatography (2% to 6% ethyl acetate-petroleum ether) to give **57a** as a colorless oil (71.6mg, 72%) and **57b** as a white solid (10.9 mg, 11%).

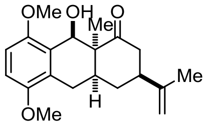
57a

***β*-hydroxyl ketone 57a:** R_f = 0.38 (20% ethyl acetate-petroleum ether); $[\alpha]^{27}_D$ = -73.1 (c = 0.45, $CHCl_3$); ^1H NMR (400 MHz, $CDCl_3$) δ 6.74 (d, J = 8.9 Hz, 1H), 6.70 (d, J = 8.9 Hz, 1H), 4.71 (d, J = 0.8 Hz, 1H), 4.68 (t, J = 1.6 Hz, 1H), 4.64 (d, J = 2.4 Hz, 1H), 3.81 (s, 3H), 3.80 (s, 3H), 3.01 (d, J = 3.0 Hz, 1H), 2.97 – 2.84 (m, 2H), 2.78 (dd, J = 18.5, 6.9 Hz, 1H), 2.61 – 2.46 (m, 2H), 2.30 (dd, J = 25.2, 12.6 Hz, 1H), 2.13 – 2.04 (m, 1H), 1.68 (s, 3H), 1.53 (ddd, J = 12.6, 5.4, 3.0 Hz, 1H), 1.10 (s, 3H) ppm; ^{13}C NMR (100 MHz, $CDCl_3$) δ 214.4, 151.7, 151.4, 148.4, 126.0, 123.5, 109.4, 108.8, 107.2, 68.2, 55.6, 55.5, 49.5, 46.9, 44.1, 38.6, 33.4, 26.5, 20.6, 19.8 ppm; IR ν_{max} 3560, 2934, 1695, 1475, 1253, 1080, 1019, 890, 797, 711 cm^{-1}; HRMS–EI (*m*/*z*): $[M]^+$ calcd for $C_{20}H_{26}O_4$, 330.1831; found, 330.1833.

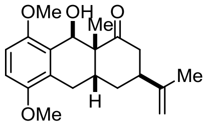
57b

***β*-hydroxyl ketone 57b:** R_f = 0.29 (20% ethyl acetate-petroleum ether); $[\alpha]^{27}_D$ = 36.2 (c = 0.15, $CHCl_3$); ^1H NMR (400 MHz, $CDCl_3$) δ 6.74 (d, J = 8.9 Hz, 1H), 6.70 (d, J = 8.8 Hz, 1H), 4.82 (s, 1H), 4.78 (s, 1H), 4.66 (s, 1H), 3.81 (s, 3H), 3.80 (s, 3H), 2.92 (s, 1H), 2.83 – 2.65 (m, 5H), 2.37 – 2.19 (m, 2H), 1.75 (s, 3H), 1.65 – 1.58 (m, 1H), 1.08 (s, 3H) ppm; ^{13}C NMR (100 MHz, $CDCl_3$) δ 216.2, 151.8, 151.4, 148.7, 126.0, 123.9, 110.5, 108.9, 107.4, 68.8, 55.7, 55.5, 49.6, 44.6, 38.9, 33.1, 30.8, 25.9, 21.4, 21.3 ppm; IR ν_{max} 3540, 2928, 1691, 1472, 1254, 1079, 1017, 803, 712 cm^{-1}; HRMS–EI (*m*/*z*): $[M]^+$ calcd for $C_{20}H_{26}O_4$, 330.1831; found, 330.1833.

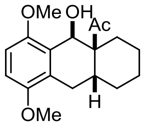
58

***β*-hydroxyl ketone 58** (72.5 mg) was prepared according to general procedure A from **7** (0.3 mmol) and **49** in 79% yield. The reaction time was 30 min under λ_{max} = 366 nm light. The products were isolated through silica gel column chromatography (8% to 12% ethyl acetate-petroleum ether) as a white solid: R_f = 0.26 (30% ethyl acetate-petroleum ether); ^1H NMR (400 MHz, $CDCl_3$) δ 6.72 (d, J = 8.8 Hz, 1H), 6.68 (d, J = 8.8 Hz, 1H), 4.90 (s, 1H), 3.83 (s, 3H), 3.80 (s, 3H), 2.80 (dd, J = 17.3, 6.1 Hz, 1H), 2.73 – 2.54 (m, 2H), 2.32 (s, 3H), 2.06 – 1.91 (m, 1H), 1.80 (d, J = 13.6 Hz, 1H), 1.61 – 1.45 (m, 4H), 1.25 (td, J = 13.5, 2.5 Hz, 1H), 1.19 – 1.05 (m, 1H) ppm; ^{13}C NMR (100 MHz, $CDCl_3$) δ 211.3, 151.5, 151.1, 125.8, 125.7, 108.7, 107.1, 68.3, 55.53, 55.48, 54.2, 27.0, 26.6, 26.2, 26.1, 24.6, 22.7, 20.0 ppm; IR ν_{max} 3456, 2922, 2854, 1701, 1462, 1253, 1079, 967, 798, 713 cm^{-1}; HRMS–EI (*m*/*z*): $[M]^+$ calcd for $C_{18}H_{24}O_4$, 304.1675; found, 304.1678.

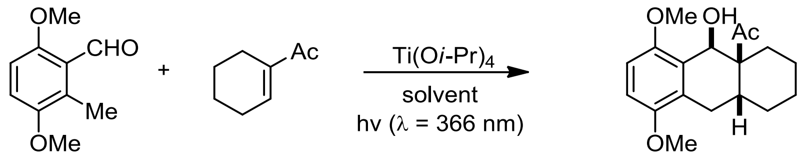

7 49 58

Supplementary Figure 95. Titanium(IV)-promoted PEDA reaction for synthesis of *β*-hydroxyl ketone **58**.

Supplementary Table 7. Effect of Ti(O*i*-Pr)$_4$ dosage on the reaction yield using 1,1-disubstituted olefin **49** as dienophile

entry	**7**	**49**	Ti(O*i*-Pr)$_4$	solvent	isolated yield
1	0.3 mmol	1.8 mmol, 6.0 equiv.	0.9 mmol, 3.0 equiv.	dioxane	79%
2	0.3 mmol	1.8 mmol, 6.0 equiv.	0.15 mmol, 0.5 equiv.	dioxane	19%
3	0.3 mmol	1.8 mmol, 6.0 equiv.	0.03 mmol, 0.1 equiv.	dioxane	9%
4	0.3 mmol	0.45 mmol, 1.5 equiv.	0.36 mmol, 1.2 equiv.	toluene	72%
5	0.3 mmol	0.45 mmol, 1.5 equiv.	0.15 mmol, 0.5 equiv.	toluene	53%
6	0.2 mmol	0.3 mmol, 1.5 equiv.	0.02 mmol, 0.1 equiv.	toluene	27%

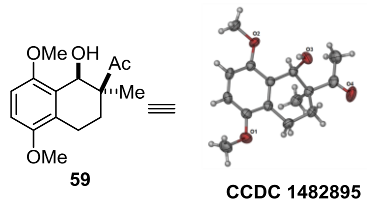

59 CCDC 1482895

***β*-hydroxyl ketone 59** (104 mg) was prepared according to general procedure A from **7** (0.5 mmol) and **50** (3.0 mmol) in 78% yield. The reaction time was 40 min under λ_{max} = 366 nm light. Two parallel reactions were conducted in two quartz tubes, combined for workup. The products were isolated through silica gel column chromatography (10% to 12% ethyl acetate-petroleum ether) as a white solid: R_f = 0.21 (30% ethyl acetate-petroleum ether); m.p. 103 – 105 ^oC; Recrystallization of **59** from dichloromethane/hexane (v/v, 1:3), CCDC 1482895. ^{1}H NMR (400 MHz, $CDCl_3$) δ 6.73 (d, J = 8.9 Hz, 1H), 6.70 (d, J = 8.9 Hz, 1H), 5.09 (d, J = 1.3 Hz, 1H), 3.84 (s, 3H), 3.79 (s, 3H), 2.94 (ddd, J = 18.5, 6.1, 1.3 Hz, 1H), 2.47 (ddd, J = 18.8, 12.7, 6.5 Hz, 1H), 2.33 (s, 3H), 2.15 – 2.05 (m, 1H), 1.94 – 1.87 (m, 1H), 1.00 (s, 3H). ppm; ^{13}C NMR (100 MHz, $CDCl_3$) δ 212.8, 152.0, 151.2, 126.2, 126.0, 109.0, 107.6, 67.4, 55.64, 55.60, 49.9, 25.5, 22.3, 20.0, 18.1 ppm; IR ν_{max} 3583, 2933, 1692, 1478, 1255, 1105, 1077, 1022, 845, 802, 709 cm^{-1}; HRMS–EI (*m*/*z*): $[M]^+$ calcd for $C_{15}H_{20}O_4$, 264.1362; found, 264.1361.

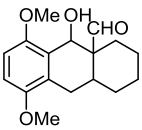

60

***β*-hydroxyl aldehyde 60** (65 mg, dr = 3:1) was prepared according to general procedure A from **7** (0.3 mmol) and **51** in 75% yield. The reaction time was 45 min under λ_{max} = 366 nm light. The products were isolated through silica gel column chromatography (2% to 4% ethyl

acetate-petroleum ether) to give **60a** (53.0 mg, 61%) and **60b** (12.0 mg, 14%) as colorless oil:

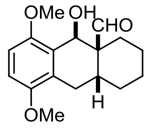
60a

***β*-hydroxyl aldehyde 60a:** R_f = 0.26 (20% ethyl acetate-petroleum ether); ^{1}H NMR (500 MHz, $CDCl_3$) δ 9.86 (s, 1H), 6.73 (d, J = 8.8 Hz, 1H), 6.70 (d, J = 8.9 Hz, 1H), 5.04 (s, 1H), 3.83 (s, 3H), 3.80 (s, 3H), 2.84 (dd, J = 18.7, 6.9 Hz, 1H), 2.63 (dd, J = 18.7, 7.9 Hz, 1H), 2.54 – 2.46 (m, 1H), 1.74 – 1.33 (m, 8H) ppm; ^{13}C NMR (125 MHz, $CDCl_3$) δ 207.7, 151.6, 151.3, 125.8, 125.6, 108.8, 107.4, 67.6, 55.6, 55.5, 51.2, 29.8, 27.2, 25.6, 25.0, 21.5 ppm; IR ν_{max} 3480, 2929, 1721, 1477, 1254, 1080, 799, 730 cm^{-1}; HRMS–EI (*m*/*z*): $[M]^+$ calcd for $C_{17}H_{22}O_4$, 290.1518; found, 290.1522.

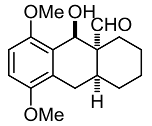
60b

***β*-hydroxyl aldehyde 60b:** R_f = 0.43 (20% ethyl acetate-petroleum ether); ^{1}H NMR (400 MHz, $CDCl_3$) δ 9.78 (s, 1H), 6.69 (s, 2H), 5.07 (s, 1H), 4.39 (s, 1H), 3.83 (s, 3H), 3.79 (s, 3H), 2.81 (dd, J = 18.2, 10.1 Hz, 1H), 2.64 (dd, J = 18.5, 6.5 Hz, 1H), 2.31 – 2.23 (m, 1H), 2.23 – 2.16 (m, 1H), 1.83 – 1.42 (m, 7H) ppm; ^{13}C NMR (100 MHz, $CDCl_3$) δ 207.5, 152.0, 151.2, 126.1, 125.5, 108.2, 107.9, 69.8, 55.7, 55.6, 52.1, 31.0, 27.2, 24.2, 21.7, 21.1, 20.3 ppm; IR ν_{max} 3545, 2925, 1725, 1476, 1455, 1253, 1082, 801, 715 cm^{-1}; HRMS–EI (*m*/*z*): $[M]^+$ calcd for $C_{17}H_{22}O_4$, 290.1518; found, 290.1519.

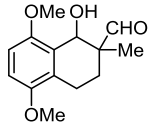
61

***β*-hydroxyl aldehyde 61** (62 mg, dr = 1.7:1) was prepared according to general procedure A from **7** (0.3 mmol) and **53** in 82% yield. The reaction time was 50 min under λ_{max} = 366 nm light. The products were isolated through silica gel column chromatography (3% to 5% ethyl acetate-petroleum ether) to give **61a** (40.0 mg, 53%) and **61b** (22.0 mg, 29%) as colorless oil:

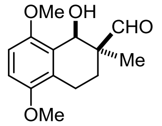
61a

***β*-hydroxyl aldehyde 61a:** R_f = 0.15 (10% ethyl acetate-petroleum ether); ^{1}H NMR (400 MHz, $CDCl_3$) δ 9.81 (s, 1H), 6.74 (d, J = 8.9 Hz, 1H), 6.71 (d, J = 8.9 Hz, 1H), 5.04 (s, 1H), 3.84 (s, 3H), 3.79 (s, 3H), 2.94 (ddd, J = 18.5, 6.1, 2.5 Hz, 1H), 2.51 (ddd, J = 18.4, 11.8, 6.3 Hz, 1H), 2.19 – 2.09 (m, 1H), 1.78 (dd, J = 13.6, 5.2 Hz, 1H), 0.99 (s, 3H) ppm; ^{13}C NMR (100 MHz, $CDCl_3$) δ 205.7, 152.0, 151.3, 126.4, 125.8, 109.1, 107.6, 67.3, 55.6, 55.6, 48.1, 21.8, 19.4, 16.3 ppm; IR ν_{max} 3480, 2935, 2835, 1630, 1478, 1254, 1079, 798, 714 cm^{-1}; HRMS–EI (*m*/*z*): $[M]^+$ calcd for $C_{14}H_{18}O_4$, 250.1205; found, 250.1206.

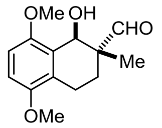
61b

***β*-hydroxyl aldehyde 61b:** R_f = 0.20 (10% ethyl acetate-petroleum ether); ^{1}H NMR (400 MHz, $CDCl_3$) δ 9.57 (s, 1H), 6.70 (s, 2H), 5.17 (s, 1H), 3.85 (s, 3H), 3.76 (s, 3H), 3.12 (br, 1H), 2.76 (dt, J = 18.5, 5.8 Hz, 1H), 2.63 – 2.52 (m, 1H), 1.94 – 1.79 (m, 2H), 1.24 (s, 3H) ppm; ^{13}C NMR (100 MHz, $CDCl_3$) δ 204.8, 151.9, 151.3, 127.1, 126.2, 108.8, 107.8, 65.8, 55.7, 55.6, 49.2, 25.1, 20.4, 16.6 ppm; IR ν_{max} 3490, 2934, 2835, 1720, 1476, 1251, 1077, 1024, 800, 713 cm^{-1}; HRMS–EI (*m*/*z*): $[M]^+$ calcd for $C_{14}H_{18}O_4$, 250.1205; found, 250.1204.

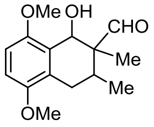
62

***β*-hydroxyl aldehyde 62** (63.8 mg, dr = 6.4:1:4.2:5) was prepared according to general procedure A from **7** (0.3 mmol) and **52** in 80% yield. The reaction time was 50 min under λ_{max} = 366 nm light. The products were isolated through silica gel column chromatography (2% to 7% ethyl acetate-petroleum ether) to give an inseparable mixture of **62a** (29 mg, 36%) as a colorless oil and an inseparable **62b** (34.8 mg, 44%) as a white solid:

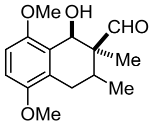
62a

***β*-hydroxyl aldehyde 62a:** R_f = 0.23 (20% ethyl acetate-petroleum ether); ^{1}H NMR (400 MHz, $CDCl_3$) δ 9.92 (s, 1H, major), 9.65 (s, 1H, minor), 6.73 (d, J = 8.9 Hz, 1H), 6.69 (d, J = 9.3 Hz, 1H), 5.09 (s, 1H, minor), 4.91 (s, 1H, major), 3.84 (s, 3H, minor), 3.807 (s, 3H, major), 3.795 (s, 3H, major), 3.77 (s, 3H, minor), 2.99 – 2.89 (m, 1H), 2.80 – 2.68 (m, 1H), 2.40 (dd, J = 18.2, 9.9 Hz, 1H, minor), 2.17 (dd, J = 18.3, 11.6 Hz, 1H, major), 1.28 (s, 3H, minor), 1.14 (d, J = 7.0 Hz, 3H, minor), 1.03 (d, J = 6.8 Hz, 3H, major), 0.85 (s, 3H, major) ppm; ^{13}C NMR (100 MHz, $CDCl_3$) δ 208.7, 151.7, 150.9, 126.3, 126.0, 109.1, 107.6, 70.0, 55.6 (2C), 50.5, 28.7, 25.9, 15.8, 10.6 ppm; IR ν_{max} 3430, 2925, 1720, 1600, 1479, 1254, 1081, 959, 799, 712 cm^{-1}; HRMS–EI (*m/z*): $[M]^+$ calcd for $C_{15}H_{20}O_4$, 264.1362; found, 264.1369.

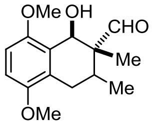
62b

***β*-hydroxyl aldehyde 62b:** R_f = 0.35 (20% ethyl acetate-petroleum ether); ^{1}H NMR (400 MHz, $CDCl_3$) δ 9.83 (s, 1H, minor), 9.65 (s, 1H, major), 6.78 – 6.67 (m, 2H), 5.27 (s, 1H, major), 4.90 (s, 1H, minor), 4.60 (s, 1H, major), 4.24 (s, 1H, minor), 3.87 (s, 3H, minor), 3.84 (s, 3H, major), 3.793 (s, 3H, minor), 3.785 (s, 3H, major), 2.85 (dd, J = 18.3, 5.5 Hz, 1H, minor), 2.75 (dd, J = 18.0, 5.2 Hz, 1H, major), 2.45 (dd, J = 18.4, 10.5 Hz, 1H, minor), 2.32 (dd, J = 18.1, 11.4 Hz, 1H, major), 2.15 – 2.03 (m, 1H, major), 2.02 – 1.91 (m, 1H, minor), 1.25 (s, 3H, minor), 1.07 (s, 3H, major), 1.06 (d, J = 6.9 Hz, 3H, minor), 0.96 (d, J = 6.8 Hz, 3H, major) ppm; ^{13}C NMR (100 MHz, $CDCl_3$) δ 206.5, 206.2, 152.4, 151.9, 151.14, 151.06, 126.8, 126.3, 126.2, 125.9, 108.7, 108.4 (2C), 108.1, 72.9, 70.3, 55.7, 55.63, 55.58, 55.54, 53.1, 51.8, 34.4, 30.7, 29.4, 28.7, 17.8, 16.3, 15.8, 6.8 ppm; IR ν_{max} 3431, 2926, 1724, 1600, 1478, 1254, 1082, 962, 799, 712 cm^{-1}; HRMS–EI (*m/z*): $[M]^+$ calcd for $C_{15}H_{20}O_4$, 264.1362; found, 264.1360.

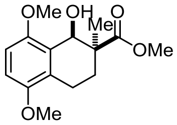
63

***β*-hydroxyl ester 63** (49.5 mg) was prepared according to general procedure A from **7** (0.3 mmol) and **55** in 59% yield, the solvent was replaced with anhydrous and degassed toluene (15 mL). The reaction time was 30 min under λ_{max} = 366 nm light. The products were isolated through silica gel column chromatography (10% to 15% ethyl acetate-petroleum ether) as a white solid: R_f = 0.33 (30% ethyl acetate-petroleum ether); m.p. 75 – 77 ^{o}C; ^{1}H NMR (400 MHz, $CDCl_3$) δ 6.72 (d, J = 8.9 Hz, 1H), 6.69 (d, J = 8.9 Hz, 1H), 5.06 (d, J = 1.1 Hz, 1H), 3.83 (s, 3H), 3.783 (s, 3H), 3.781 (s, 3H), 2.92 (ddd, J = 18.5, 6.2, 1.4 Hz, 1H), 2.58 (br, 1H), 2.53 – 2.42 (m, 1H), 2.30 – 2.18 (m, 1H), 1.98 – 1.90 (m, 1H), 1.09 (s, 3H) ppm; ^{13}C NMR (100 MHz, $CDCl_3$) δ 177.2, 152.1, 151.1, 125.8, 125.7, 108.9, 107.6, 67.1,

55.6, 55.5, 51.8, 45.5, 22.7, 19.8, 18.9 ppm; IR ν_{max} 3512, 2935, 1728, 1476, 1252, 1122, 1077, 1022, 799, 712 cm^{-1}; HRMS–EI (*m*/*z*): $[M]^+$ calcd for $C_{15}H_{20}O_5$, 280.1311; found, 280.1314.

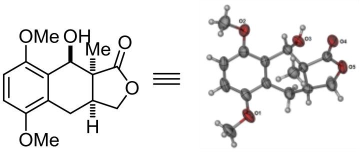

64 CCDC 1482897

***β*-hydroxyl lactone 64** (65 mg) was prepared according to general procedure A from **7** (0.3mmol) and **54** in 78% yield. The reaction time was 30 min under λ_{max} = 366 nm light. The products were isolated through silica gel column chromatography (8% to 15% ethyl acetate-petroleum ether) as a white solid: R_f = 0.17 (30% ethyl acetate-petroleum ether); m.p. 131 – 133 °C; Recrystallization of **64** from dichloromethane/hexane (v/v, 1:3), CCDC 1482897. ^{1}H NMR (400 MHz, $CDCl_3$) δ 6.79 (d, J = 8.9 Hz, 1H), 6.73 (d, J = 8.9 Hz, 1H), 5.25 (s, 1H), 4.50 (t, J = 8.6 Hz, 1H), 4.15 (t, J = 8.7 Hz, 1H), 3.80 (s, 3H), 3.79 (s, 3H), 3.24 (dd, J = 16.8, 8.6 Hz, 1H), 2.67 (dd, J = 16.9, 6.0 Hz, 1H), 2.63 – 2.52 (m, 1H), 2.35 (s, 1H), 1.08 (s, 3H) ppm; ^{13}C NMR (100 MHz, $CDCl_3$) δ 180.8, 151.4, 150.9, 125.8, 124.2, 110.6, 108.7, 72.4, 66.7, 56.0, 55.9, 47.3, 39.0, 22.7, 22.1 ppm; IR ν_{max} 3485, 2921, 2849, 1761, 1482, 1254, 1228, 1075, 1017, 804, 716 cm^{-1}; HRMS–EI (*m*/*z*): $[M]^+$ calcd for $C_{15}H_{18}O_5$, 278.1154; found, 278.1146.

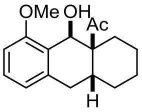

65

***β*-hydroxyl ketone 65** (59 mg) was prepared according to general procedure B from **12** (0.3 mmol) and **49** in 71% yield, the reaction time was 30 min under λ_{max} = 366 nm light. The products were isolated through silica gel column chromatography (5% to 15% ethyl acetate-petroleum ether) as a white solid: R_f = 0.21 (20% ethyl acetate-petroleum ether); ^{1}H NMR (400 MHz, $CDCl_3$) δ 7.20 (t, J = 7.9 Hz, 1H), 6.77 (d, J = 7.7 Hz, 1H), 6.71 (d, J = 8.1 Hz, 1H), 4.92 (s, 1H), 3.86 (s, 3H), 2.98 – 2.85 (m, 1H), 2.81– 2.68 (m, 2H), 2.32 (s, 3H), 2.20 (s, 1H), 1.97 (t, J = 12.0 Hz, 1H), 1.82 (d, J = 13.7 Hz, 1H), 1.62 – 1.40 (m, 4H), 1.29 (td, J = 13.5, 2.7 Hz, 1H), 1.20 – 1.05 (m, 1H) ppm; ^{13}C NMR (100 MHz, $CDCl_3$) δ 211.2, 157.8, 136.9, 128.6, 124.6, 121.1, 107.3, 68.2, 55.3, 54.6, 29.9, 27.1, 26.8, 26.5, 26.3, 22.7, 20.1 ppm; IR ν_{max} 3380, 2923, 2859, 1699, 1590, 1464, 1259, 1078, 1012, 737 cm^{-1}; HRMS–ESI (*m*/*z*): $[M+Na]^+$ calcd for $C_{17}H_{22}O_3Na$, 297.1467; found, 297.1462.

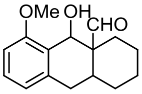

66

***β*-hydroxyl aldehyde 66** (66 mg, dr = 5.3:1) was prepared according to general procedure **A** from **12** (0.3 mmol) and **51** in 84% yield. The reaction time was 40 min under λ_{max} = 366 nm light. The products were isolated through silica gel column chromatography (1% to 6% ethyl acetate-petroleum ether) to give **66a** (54 mg, 69%) as a colorless oil and **66b** (12 mg, 15%) as a white solid.

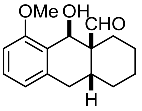

66a

***β*-hydroxyl aldehyde 66a:** R_f = 0.31 (20% ethyl acetate-petroleum ether); ppm; ^{1}H NMR (400 MHz, $CDCl_3$) δ 9.88 (s, 1H), 7.21 (t, J = 7.9 Hz, 1H), 6.78 (d, J = 7.7 Hz, 1H), 6.74 (d, J = 8.2

Hz, 1H), 5.08 (s, 1H), 3.87 (s, 3H), 3.04 (br, 1H), 2.97 (dd, J = 17.9, 6.6 Hz, 1H), 2.76 (dd, J = 17.9, 7.6 Hz, 1H), 2.52 – 2.41 (m, 1H), 1.75 – 1.36 (m, 8H) ppm; ^{13}C NMR (100 MHz, $CDCl_3$) δ 207.7, 157.9, 136.7, 128.6, 124.5, 121.6, 107.6, 67.4, 55.3, 51.6, 31.0, 30.7, 27.2, 25.7, 21.8, 21.5 ppm; IR ν_{max} 3462, 2924, 2859, 1719, 1590, 1464, 1255, 1078, 776, 747 cm^{-1}; HRMS–EI (*m/z*): $[M]^+$ calcd for $C_{16}H_{20}O_3$, 260.1412; found, 260.1415.

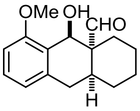

66b

***β*-hydroxyl aldehyde 66b:** R_f = 0.56 (20% ethyl acetate-petroleum ether); ^{1}H NMR (400 MHz, $CDCl_3$) δ 9.75 (s, 1H), 7.17 (t, J = 7.9 Hz, 1H), 6.76 (d, J = 7.7 Hz, 1H), 6.73 (d, J = 8.2 Hz, 1H), 5.11 (s, 1H), 4.16 (s, 1H), 3.87 (s, 3H), 3.09 (dd, J = 17.5, 10.3 Hz, 1H), 2.62 (dd, J = 17.5, 6.1 Hz, 1H), 2.35 – 2.25 (m, 1H), 2.23 – 2.15 (m, 1H), 1.86 – 1.76 (m, 1H), 1.72 – 1.43 (m, 6H) ppm; ^{13}C NMR (100 MHz, $CDCl_3$) δ 207.0, 158.1, 136.5, 128.0, 124.9, 121.6, 107.9, 69.2, 55.4, 52.3, 31.7, 29.9, 27.2, 21.8, 21.5, 20.6 ppm; IR ν_{max} 3489, 2925, 2861, 1722, 1586, 1459, 1380, 1245, 1070, 1026, 779, 751 cm^{-1}; HRMS–EI (*m/z*): $[M]^+$ calcd for $C_{16}H_{20}O_3$, 260.1412; found, 260.1411.

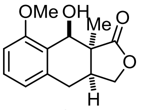

67

***β*-hydroxyl lactone 67** (59 mg) was prepared according to general procedure A from **12** (0.3mmol) and **54** in 79% yield, the solvent was replaced with anhydrous and degassed toluene (15 mL). The reaction time was 40 min under λ_{max} = 366 nm light. The products were isolated through silica gel column chromatography (8% to 13% ethyl acetate-petroleum ether) as a colorless oil which turned into a white solid in the refrigerator: R_f = 0.33 (30% ethyl acetate-petroleum ether); m.p. 118 – 120 °C; ^{1}H NMR (400 MHz, $CDCl_3$) δ 7.24 (d, J = 7.9 Hz, 1H), 6.80 (d, J = 2.8 Hz, 1H), 6.78 (d, J = 3.8 Hz, 1H), 5.35 (s, 1H), 4.57 (t, J = 8.7 Hz, 1H), 4.14 (t, J = 8.3 Hz, 1H), 3.83 (s, 3H), 3.07 – 2.93 (m, 2H), 2.64 – 2.53 (m, 1H), 2.31 (br, 1H), 1.05 (s, 3H) ppm; ^{13}C NMR (100 MHz, $CDCl_3$) δ 180.9, 157.1, 136.6, 129.5, 124.5, 120.6, 108.8, 72.9, 66.8, 55.5, 48.4, 39.8, 30.4, 23.3 ppm; IR ν_{max} 3432, 2924, 2854, 1766, 1588, 1467, 1226, 1015, 774 cm^{-1}; HRMS–EI (*m/z*): $[M]^+$ calcd for $C_{14}H_{16}O_4$, 248.1049; found, 248.1051.

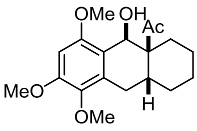

68

***β*-hydroxyl ketone 68** (72 mg) was prepared according to general procedure A from **13** (0.3mmol) and **49** in 72% yield, the reaction time was 3 h under λ_{max} = 366 nm light. The products were isolated through silica gel column chromatography (10% to 25% ethyl acetate-petroleum ether) as a light yellow oil which washed into a white solid with hexane: R_f = 0.15 (30% ethyl acetate-petroleum ether); ^{1}H NMR (400 MHz, $CDCl_3$) δ 6.40 (s, 1H), 4.87 (s, 1H), 3.88 (s, 3H), 3.86 (s, 3H), 3.77 (s, 3H), 2.84 (dd, J = 23.5, 12.1 Hz, 1H), 2.76 – 2.64 (m, 2H), 2.31 (s, 3H), 2.09 – 1.91 (m, 2H), 1.79 (d, J = 13.3 Hz, 1H), 1.59 – 1.42 (m, 4H), 1.28 – 1.05 (m, 2H) ppm; ^{13}C NMR (100 MHz, $CDCl_3$) δ 211.4, 154.3, 152.2, 139.7, 130.6, 117.1, 94.3, 68.1, 60.0, 55.8, 55.5, 54.3, 26.9, 26.5, 26.1 (2C), 24.6, 22.7, 19.9 ppm; IR ν_{max} 3462, 2926, 2864, 1703, 1599, 1445, 1332, 1209, 1085, 996 cm^{-1}; HRMS–EI (*m/z*): $[M]^+$ calcd for $C_{19}H_{26}O_5$, 334.1780; found, 334.1775.

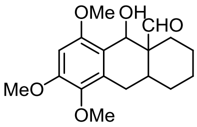

69

***β*-hydroxyl aldehyde 69** (85.5 mg, dr = 9.3:1) was prepared according to general procedure B from **13** (0.3 mmol) and **51** in 89% yield. The reaction time was 4 h under λ_{max} = 366 nm light. The products were isolated through silica gel column chromatography (5% to 25% ethyl acetate-petroleum ether) to give **69a** (79 mg, 82%) and **69b** (6.5 mg, 7%) as colorless oil:

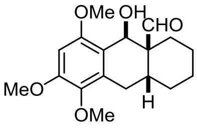

69a

***β*-hydroxyl aldehyde 69a:** R_f = 0.31 (30% ethyl acetate-petroleum ether); ^{1}H NMR (400 MHz, $CDCl_3$) δ 9.83 (s, 1H), 6.40 (s, 1H), 4.98 (s, 1H), 3.87 (s, 3H), 3.85 (s, 3H), 3.75 (s, 3H), 2.89 (dd, J = 18.6, 6.9 Hz, 1H), 2.77 (br, 1H), 2.72 (dd, J = 18.6, 8.2 Hz, 1H), 2.54 – 2.45 (m, 1H), 1.75 – 1.64 (m, 2H), 1.62 – 1.30 (m, 6H).ppm; ^{13}C NMR (101 MHz, $CDCl_3$) δ 207.7, 154.3, 152.3, 140.0, 130.6, 117.0, 94.5, 67.4, 60.0, 55.9, 55.6, 51.3, 29.5, 27.0, 25.5, 24.9, 21.5, 21.3 ppm; IR ν_{max} 2924, 2841, 1730, 1595, 1486, 1462, 1440, 1322, 1234, 1204, 1124, 1080, 1043, 988, 804 cm^{-1}; HRMS–EI (*m*/*z*): $[M]^+$ calcd for $C_{18}H_{24}O_5$, 320.1624; found, 320.1628.

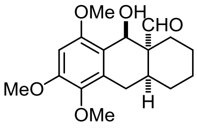

69b

***β*-hydroxyl aldehyde 69b:** R_f = 0.44 (30% ethyl acetate-petroleum ether); ^{1}H NMR (400 MHz, $CDCl_3$) δ 9.73 (s, 1H), 6.40 (s, 1H), 5.05 (s, 1H), 4.05 (s, 1H), 3.87 (s, 3H), 3.86 (s, 3H), 3.75 (s, 3H), 2.90 (dd, J = 18.2, 10.1 Hz, 1H), 2.69 (dd, J = 18.2, 6.2 Hz, 1H), 2.28 – 2.10 (m, 2H), 1.86– 1.75 (m, 1H), 1.73 – 1.44 (m, 6H) ppm; ^{13}C NMR (100 MHz, $CDCl_3$) δ 207.1, 154.4, 151.8, 140.1, 130.5, 117.5, 95.0, 69.0, 60.1, 56.0, 55.7, 52.1, 31.3, 27.3, 24.4, 21.7, 21.4, 20.6 ppm; IR ν_{max} 3518, 2926, 2848, 1724, 1596, 1463, 1322, 1207, 1083, 1019, 943, 811 cm^{-1}; HRMS–EI (*m*/*z*): $[M]^+$ calcd for $C_{18}H_{24}O_5$, 320.1624; found, 320.1627.

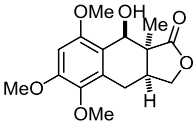

70

***β*-hydroxyl lactone 70** (75 mg) was prepared according to general procedure A from **13** (0.3 mmol) and **54** in 81% yield, the solvent was replaced with anhydrous and degassed toluene (15 mL). The reaction time was 40 min under λ_{max} = 366 nm light. The products were isolated through silica gel column chromatography (15% to 25% ethyl acetate-petroleum ether) as a white solid: R_f = 0.13 (30% ethyl acetate-petroleum ether); m.p. 151 – 153 °C; ^{1}H NMR (400 MHz, $CDCl_3$) δ 6.40 (s, 1H), 5.26 (s, 1H), 4.56 (t, J = 8.7 Hz, 1H), 4.15 (t, J = 8.2 Hz, 1H), 3.89 (s, 3H), 3.82 (s, 3H), 3.74 (s, 3H), 3.34 (dd, J = 15.7, 8.3 Hz, 1H), 2.73 (dd, J = 15.8, 8.0 Hz, 1H), 2.57 – 2.46 (m, 1H), 2.27 (s, 1H), 1.04 (s, 3H) ppm; ^{13}C NMR (100 MHz, $CDCl_3$) δ 181.0, 153.8, 153.2, 140.0, 129.8, 116.9, 94.9, 72.9, 66.8, 60.8, 56.1, 55.8, 48.2, 39.5, 23.4, 23.3 ppm; IR ν_{max} 3433, 2929, 1736, 1598, 1491, 1330, 1230, 1085, 1054, 1007, 825 cm^{-1}; HRMS–EI (*m*/*z*): $[M]^+$ calcd for $C_{16}H_{20}O_6$, 308.1260; found, 308.1264.

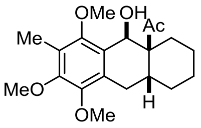
71

***β*-hydroxyl ketone 71** (77.4 mg) was prepared according to general procedure B from **14** (0.3 mmol) and **49** in 74% yield. The reaction time was 30 min under λ_{max} = 366 nm light. The products were isolated through silica gel column chromatography (10% to 15% ethyl acetate-petroleum ether) as a white solid: R_f = 0.26 (20% ethyl acetate-petroleum ether); m.p. 117 – 119 ^oC; ^1H NMR (400 MHz, $CDCl_3$) δ 4.80 (s, 1H), 3.82 (s, 6H), 3.79 (s, 3H), 2.84 (dd, J = 16.2, 5.4 Hz, 1H), 2.75 – 1.59 (m, 2H), 2.33 (s, 3H), 2.18 (s, 3H), 2.00 – 1.86 (m, 2H), 1.81 (d, J = 13.1 Hz, 1H), 1.59 – 1.45 (m, 4H), 1.27 – 1.06 (m, 2H) ppm; ^{13}C NMR (100 MHz, $CDCl_3$) δ 211.8, 153.4, 151.6, 146.8, 127.6, 125.3, 123.0, 68.9, 61.7, 60.0, 59.8, 54.6, 27.1, 26.7, 26.3, 26.0, 24.5, 22.6, 20.0, 9.4 ppm; IR ν_{max} 3406, 2931, 2861, 1703, 1678, 1459, 1345, 1069, 1017 cm^{-1}; HRMS–EI (*m*/*z*): $[M]^+$ calcd for $C_{20}H_{28}O_5$, 348.1937; found, 348.1940.

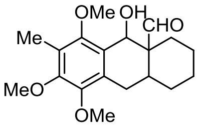
72

***β*-hydroxyl aldehyde 72** (87.7 mg, dr = 7.2:1) was prepared according to general procedure B from **14** (0.3 mmol) and **51** in 87% yield, the reaction time was 45 min under λ_{max} = 366 nm light. The products were isolated through silica gel column chromatography (4% to 8% ethyl acetate-petroleum ether) to give **72a** (75.2 mg, 75%) and **72b** (12.5 mg, 12%) as colorless oil:

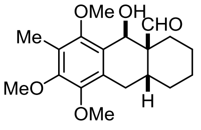
72a

***β*-hydroxyl aldehyde 72a:** R_f = 0.36 (20% ethyl acetate-petroleum ether); ^1H NMR (400 MHz, $CDCl_3$) δ 9.84 (s, 1H), 4.94 (s, 1H), 3.83 (s, 3H), 3.81 (s, 3H), 3.77 (s, 3H), 2.89 (dd, J = 18.4, 7.1 Hz, 1H), 2.78 (br, 1H), 2.67 (dd, J = 18.4, 8.4 Hz, 1H), 2.61 – 2.52 (m, 1H), 2.18 (s, 3H), 1.78 – 1.65 (m, 2H), 1.63 – 1.44 (m, 4H), 1.42 – 1.29 (m, 2H) ppm; ^{13}C NMR (100 MHz, $CDCl_3$) δ 207.9, 153.3, 151.6, 147.0, 127.6, 125.2, 123.2, 68.0, 61.3, 60.1, 59.8, 51.4, 28.9, 27.1, 25.6, 24.7, 21.6, 21.1, 9.3 ppm; IR ν_{max} 3460, 2930, 2858, 1724, 1456, 1331, 1113, 1073, 1011, 968, 656 cm^{-1}; HRMS–EI (*m*/*z*): $[M]^+$ calcd for $C_{19}H_{26}O_5$, 334.1780; found, 334.1783.

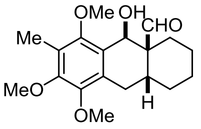
72b

***β*-hydroxyl aldehyde 72b:** R_f = 0.60 (20% ethyl acetate-petroleum ether); ^1H NMR (400 MHz, $CDCl_3$) δ 9.80 (s, 1H), 5.05 (s, 1H), 4.58 (br, 1H), 3.814 (s, 3H), 3.809 (s, 3H), 3.74 (s, 3H), 2.87 (dd, J = 18.3, 10.1 Hz, 1H), 2.63 (dd, J = 18.2, 6.6 Hz, 1H), 2.32 – 2.23 (m, 1H), 2.22 – 2.16 (m, 1H), 2.16 (s, 3H), 1.84 – 1.75 (m, 1H), 1.72 – 1.61 (m, 1H), 1.59 – 1.36 (m, 5H) ppm; ^{13}C NMR (100 MHz, $CDCl_3$) δ 207.8, 153.1, 150.9, 146.8, 127.1, 125.4, 123.2, 70.1, 60.3, 60.1, 59.9, 52.3, 30.9, 27.2, 24.2, 21.6, 21.3, 20.4, 9.2 ppm; IR ν_{max} 3448, 2930, 1726, 1460, 1407, 1328, 1115, 1078, 1011, 968, 736 cm^{-1}; HRMS–EI (*m*/*z*): $[M]^+$ calcd for $C_{19}H_{26}O_5$, 334.1780; found, 334.1783.

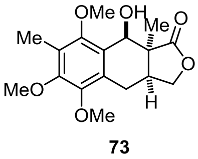

***β*-hydroxyl lactone 73** (63 mg) was prepared according to general procedure A from **14** (0.3 mmol) and **54** in 65% yield, the solvent was replaced with anhydrous and degassed toluene (15 mL). The reaction time was 30 min under λ_{max} = 366 nm light. The products were isolated through silica gel column chromatography (10% to 20% ethyl acetate-petroleum ether) as a white solid: R_f = 0.25 (30% ethyl acetate-petroleum ether); m.p. 126 – 128 °C; ^{1}H NMR (400 MHz, $CDCl_3$) δ 5.20 (s, 1H), 4.61 (t, J = 8.8 Hz, 1H), 4.16 (t, J = 7.9 Hz, 1H), 3.83 (s, 3H), 3.79 (s, 3H), 3.74 (s, 3H), 3.32 (dd, J = 15.3, 8.1 Hz, 1H), 2.71 (dd, J = 15.3, 8.9 Hz, 1H), 2.56 – 2.41 (m, 2H), 2.19 (s, 3H), 1.08 (s, 3H) ppm; ^{13}C NMR (100 MHz, $CDCl_3$) δ 181.0, 152.9, 152.1, 146.8, 127.0, 125.2, 123.4, 73.2, 68.1, 62.0, 60.7, 60.2, 48.8, 39.7, 23.8, 23.8, 9.3 ppm; IR ν_{max} 3408, 2936, 1755, 1467, 1397, 1247, 1114, 1060, 1002, 960 cm^{-1}; HRMS–EI (*m*/*z*): $[M]^+$ calcd for $C_{17}H_{22}O_6$, 322.1416; found, 322.1410.

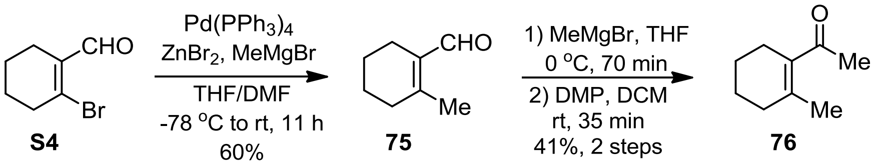

Supplementary Figure 96. Synthesis of alkenyl ketone **76**.

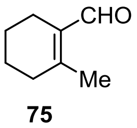

Alkenyl aldehyde 75: Zinc bromide (11.9 g, 52.9 mmol, 2.0 equiv.) in a round bottom flask was pre-activated at 140 °C under oil pump for 2 h, after cooling to rt under oil pump, the gas inside was replaced into N_2. Then anhydrous tetrahydrofuran (100 mL) was added, the resulting mixture was cooled to -78 °C, methyl magnesium bromide (17.6 mL, 3.0 M in diethyl ether, 52.9 mmol, 2.0 equiv.) was added dropwise to the above mixture, after the addition, the resulting mixture was moved to stir at rt for 1 h. Then the solution of **S4** (5.0 g, 26.45 mmol, 1.0 equiv.) and tetrakis(triphenyl-phosphine)palladium (1.53 g, 1.32 mmol, 0.05 equiv.) in anhydrous tetrahydrofuran/N,N-dimethylformamide (25 mL/25 mL) was added dropwise to the above mixture at rt, the resulting mixture was stirred at rt for 11 h. The reaction mixture was quenched with saturated ammonium chloride (100 mL). The resulting mixture was extracted with ethyl acetate (3×80 mL), the combined organic layer was sequentially washed with water (3×80 mL), brine (3×80 mL) and dried over anhydrous sodium sulfate. The dried solution was filtered and the filtrate was concentrated under vacuum. The residue was purified by silica gel column chromatography (0% to 1% ethyl acetate-petroleum ether) to give **75** as a light yellow oil (1.97 g, 60%). ^{1}H NMR (400 MHz, $CDCl_3$) δ 10.14 (s, 1H), 2.25 – 2.15 (m, 4H), 2.13 (s, 3H), 1.66 – 1.55 (m, 4H) ppm; ^{13}C NMR (100 MHz, $CDCl_3$) δ 191.1 (d, J = 1.5 Hz), 156.1, 133.7, 34.2, 22.1, 22.0, 21.7, 18.3 ppm. The spectroscopic data are identical in all respects to those previously reported.[8]

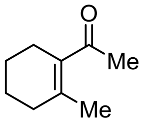
76

Alkenyl ketone 76: To a stirred solution of **75** (700 mg, 5.64 mmol, 1.0 equiv.) in anhydrous tetrahydrofuran (25 mL) was added methyl magnesium bromide (2.8 mL, 3.0 M in diethyl ether, 8.46 mmol, 1.5 equiv.) dropwise at 0 ^{o}C under N_2, the resulting mixture was stirred at 0 ^{o}C for 70 min. The reaction mixture was quenched with saturated ammonium chloride (20 mL) at 0 ^{o}C. The resulting mixture was extracted with ethyl acetate (3 ×15 mL), the combined organic layer was washed with brine (2 ×20 mL) and dried over anhydrous sodium sulfate. The dried solution was filtered and the filtrate was concentrated under vacuum. The residue (656 mg, 4.68 mmol, 1.0 equiv.) was dissolved in anhydrous dichloromethane (20 mL), then Dess-Martin periodinane (2.98 g, 7.02 mmol, 1.5 equiv.) was added to the above mixture at rt under N_2, after stirring for 2 min, 40 uL water (H_2O/DCM, 2 uL/1 mL) was added to the above mixture, the resulting suspension was stirred at rt for 35 min. Then the solvent was removed under vacuum, an aqueous solution of 1:1(v/v) 10% sodium thiosulfate /saturated sodium bicarbonate (40 mL) and ethyl acetate (40 mL) were added. After stirring for another 5 min, the mixture was separated to obtain organic layer with a separatory funnel, the aqueous layer was extracted with ethyl acetate (2 ×30 mL), the combined organic layer was washed with brine (2 ×40 mL) and dried over anhydrous sodium sulfate. The dried solution was filtered and the filtrate was concentrated under vacuum. The residue was purified by silica gel column chromatography (0% to 0.3% ethyl acetate-petroleum ether) to give **76** as a colorless oil (323 mg, 41% over 2 steps). ^{1}H NMR (400 MHz, $CDCl_3$) δ 2.25 – 2.21 (m, 2H), 2.21 (s, 3H), 2.10 – 2.04 (m, 2H), 1.85 (br, 3H), 1.66 – 1.54 (m, 4H) ppm, ^{13}C NMR (100 MHz, $CDCl_3$) δ 204.3, 141.0, 133.1, 33.1, 29.6, 26.7, 22.3, 22.2, 21.6 ppm. The spectroscopic data are identical in all respects to those previously reported.[9]

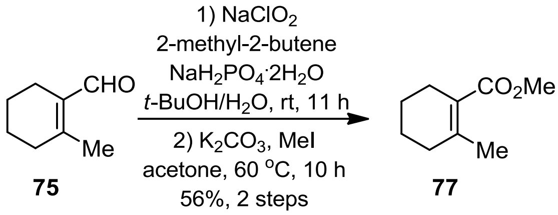

Supplementary Figure 97. Synthesis of alkenyl ester **77**.

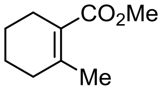
77

Alkenyl ester 77: To a stirred solution of **75** (700 mg, 5.64 mmol, 1.0 equiv.) and 2-methyl-2-butene (1.8 mL, 16.9 mmol, 3.0 equiv.) in tertiary butanol/water (28 mL, v/v, 3:1) was added sodium chlorite (1.02 g, 11.27 mmol, 2.0 equiv.), followed by sodium dihydrogen phosphate dihydrate (1.76 g, 11.27 mmol, 2.0 equiv.), the resulting mixture was stirred at rt for 11 h. Then the organic solvent was evaporated under vacuum, the mixture was diluted with water (10 mL) and extracted with ethyl acetate (3 ×15 mL), the combined organic layer was dried over anhydrous sodium sulfate. The dried solution was filtered and the filtrate was concentrated under vacuum. The residue was dissolved in acetone (20 mL), then potassium carbonate (1.56 g, 11.27 mmol, 2.0 equiv.) was added to the above mixture,

followed by methyl iodide (1.8 mL, 28.19 mmol, 5.0 equiv.), the resulting mixture was stirred at 60 °C for 10 h. Then the solvent was evaporated under vacuum, the mixture was diluted with water (15 mL) and extracted with ethyl acetate (3×15 mL), the combined organic layer was washed with brine (2×40 mL) and dried over anhydrous sodium sulfate. The dried solution was filtered and the filtrate was concentrated under vacuum. The residue was purified by silica gel column chromatography (0.3% to 0.5% ethyl acetate-petroleum ether) to give **77** as a light yellow oil (490 mg, 56% over 2 steps). ^{1}H NMR (400 MHz, $CDCl_3$) δ 3.70 (s, 3H), 2.28 – 2.20 (m, 2H), 2.14 – 2.06 (m, 2H), 1.97 (br, 3H), 1.62 – 1.53 (m, 4H) ppm, ^{13}C NMR (100 MHz, $CDCl_3$) δ 169.5, 146.2, 124.0, 51.0, 33.6, 26.3, 22.3, 22.2, 21.9 ppm. The spectroscopic data are identical in all respects to those previously reported.[10]

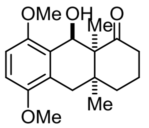

78

≡

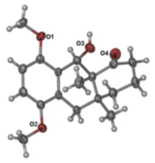

CCDC 1525307

***β*-hydroxyl ketone 78** (47 mg) was prepared according to general procedure D from **74**[11] (0.4 mmol) and **7** in 38% yield, the reaction time was 1.5 h under λ_{max} = 366 nm light. The products were isolated through silica gel column chromatography (2% to 5% ethyl acetate-petroleum ether) as a white solid: R_f = 0.52 (20% ethyl acetate-petroleum ether); m.p. 129 – 131 °C; Recrystallization of **78** from dichloromethane/hexane (v/v, 1:3), CCDC 1525307. ^{1}H NMR (500 MHz, $CDCl_3$) δ 6.72 (d, J = 8.9 Hz, 1H), 6.69 (d, J = 8.9 Hz, 1H), 4.66 (d, J = 1.3 Hz, 1H), 3.82 (s, 3H), 3.79 (s, 3H), 3.39 (d, J = 2.8 Hz, 1H), 2.83 (dt, J = 17.0, 9.4 Hz, 1H), 2.77 (d, J = 18.2 Hz, 1H), 2.48 (ddd, J = 16.8, 7.2, 3.0 Hz, 1H), 2.42 – 2.34 (m, 2H), 2.00 – 1.82 (m, 2H), 1.31 – 1.25 (m, 1H), 1.09 (s, 3H), 1.04 (s, 3H) ppm; ^{13}C NMR (125 MHz, $CDCl_3$) δ 216.0, 151.7, 151.1, 126.0, 124.3, 108.6, 107.3, 71.2, 55.6, 55.5, 53.8, 39.4, 36.0, 34.9, 32.4, 24.8, 19.8, 16.9.ppm; IR ν_{max} 3345, 2921, 1689, 1473, 1253, 1084, 1025, 969, 804, 720 cm^{-1}; HRMS–EI (*m/z*): $[M]^+$ calcd for $C_{18}H_{24}O_4$, 304.1675; found, 304.1674.

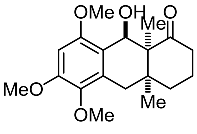

79

***β*-hydroxyl ketone 79** (75 mg) was prepared according to general procedure D from **74** (0.4 mmol) and **13** in 56% yield, the reaction time was 4 h under λ_{max} = 366 nm light. The products were isolated through silica gel column chromatography (5% to 8% ethyl acetate-petroleum ether) as a colorless oil: R_f = 0.45 (30% ethyl acetate-petroleum ether); ^{1}H NMR (500 MHz, $CDCl_3$) δ 6.42 (s, 1H), 4.63 (d, J = 3.9 Hz, 1H), 3.87 (s, 3H), 3.84 (s, 3H), 3.73 (s, 3H), 3.27 (d, J = 4.1 Hz, 1H), 2.84 – 2.76 (m, 2H), 2.51 – 2.42 (m, 2H), 2.33 (ddd, J = 13.3, 11.5, 5.7 Hz, 1H), 1.99 – 1.82 (m, 2H), 1.30 (dt, J = 13.3, 4.6 Hz, 1H), 1.09 (s, 3H), 1.02 (s, 3H) ppm; ^{13}C NMR (125 MHz, $CDCl_3$) δ 216.1, 154.4, 152.1, 139.9, 129.3, 117.5, 94.7, 71.0, 60.2, 55.9, 55.6, 53.8, 39.3, 36.2, 34.9, 32.4, 24.6, 19.8, 17.0 ppm; IR ν_{max} 3480, 2925, 1692, 1597, 1451, 1327, 1235, 1198, 1093, 913, 810, 729 cm^{-1}; HRMS–EI (*m/z*): $[M]^+$ calcd for $C_{19}H_{26}O_5$, 334.1780; found, 334.1777.

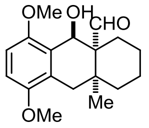
80

***β*-hydroxyl aldehyde 80** (70 mg, dr = 11:1) was prepared according to general procedure D from **75** (0.4 mmol) and **7** in 57% yield, the reaction time was 1.5 h under λ_{max} = 366 nm light. The products were isolated through silica gel column chromatography (2% to 5% ethyl acetate-petroleum ether) as a colorless oil: R_f = 0.48 (20% ethyl acetate-petroleum ether); ^{1}H NMR (500 MHz, $CDCl_3$) δ 9.84 (s, 1H), 6.75 (d, J = 8.9 Hz, 1H), 6.72 (d, J = 8.9 Hz, 1H), 5.13 (s, 1H), 3.86 (s, 3H), 3.80 (s, 3H), 3.63 (s, 1H), 2.76 (d, J = 18.3 Hz, 1H), 2.55 (d, J = 18.3 Hz, 1H), 1.92 – 1.82 (m, 1H), 1.62 – 1.40 (m, 7H), 1.16 (s, 3H).ppm; ^{13}C NMR (125 MHz, $CDCl_3$) δ 205.6, 151.7, 151.3, 125.4, 124.8, 108.7, 107.3, 68.3, 55.6, 55.5, 53.9, 35.0, 34.6, 33.7, 24.1, 20.8, 20.6 (2C) ppm; IR ν_{max} 3502, 2933, 2863, 1717, 1470, 1251, 1078, 1010, 797, 710 cm^{-1}; HRMS–EI (*m*/*z*): $[M]^+$ calcd for $C_{18}H_{24}O_4$, 304.1675; found, 304.1681.

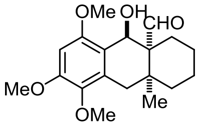
81

***β*-hydroxyl aldehyde 81** (55 mg, dr = 17:1) was prepared according to general procedure D from **75** (0.4 mmol) and **13** in 40% yield, the reaction time was 4 h under λ_{max} = 366 nm light. The products were isolated through silica gel column chromatography (5% to 8% ethyl acetate-petroleum ether) as a colorless oil: R_f = 0.30 (30% ethyl acetate-petroleum ether); ^{1}H NMR (500 MHz, $CDCl_3$) δ 9.84 (s, 1H), 6.44 (s, 1H), 5.07 (s, 1H), 3.893 (s, 3H), 3.887 (s, 3H), 3.75 (s, 3H), 3.37 (br, 1H), 2.84 (d, J = 18.0 Hz, 1H), 2.61 (d, J = 18.0 Hz, 1H), 1.91 – 1.82 (m, 1H), 1.60 – 1.38 (m, 7H), 1.17 (s, 3H) ppm; ^{13}C NMR (125 MHz, $CDCl_3$) δ 205.5, 154.4, 152.3, 140.2, 129.9, 116.9, 94.6, 68.3, 60.2, 56.0, 55.5, 53.9, 34.9, 34.6, 33.9, 24.1, 20.8, 20.5 (2C) ppm; IR ν_{max} 3540, 2935, 1723, 1599, 1459, 1326, 1236, 1205, 1086, 999, 912, 731 cm^{-1}; HRMS–EI (*m*/*z*): $[M]^+$ calcd for $C_{19}H_{26}O_5$, 334.1780; found, 334.1783.

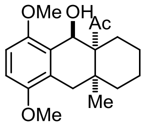
82

***β*-hydroxyl ketone 82** (15.7 mg) was prepared according to general procedure D from **76** (0.4 mmol) and **7** in 12% yield, the reaction time was 1.5 h under λ_{max} = 366 nm light. The products were isolated through silica gel column chromatography (2% to 7% ethyl acetate-petroleum ether) as a light yellow oil: R_f = 0.40 (20% ethyl acetate-petroleum ether); ^{1}H NMR (500 MHz, $CDCl_3$) δ 6.73 (d, J = 8.8 Hz, 1H), 6.70 (d, J = 8.9 Hz, 1H), 5.07 (s, 1H), 3.86 (s, 3H), 3.79 (s, 3H), 2.77 (br, 1H), 2.44 (br, 1H), 2.22 (s, 3H), 1.85 (br, 1H), 1.56 (s, 4H), 1.38 – 1.09 (m, 6H) ppm; ^{13}C NMR (125 MHz, $CDCl_3$) δ 210.4, 151.6, 151.4, 125.8, 125.4, 108.6, 106.9, 69.5, 56.5, 55.6, 55.4, 35.0, 33.8, 27.9, 24.6, 21.8 (2C), 21.0 (2C) ppm; IR ν_{max} 3539, 2924, 2853, 1708, 1462, 1256, 1236, 1080, 732, 713 cm^{-1}; HRMS–EI (*m*/*z*): $[M]^+$ calcd for $C_{19}H_{26}O_4$, 318.1831; found, 318.1833.

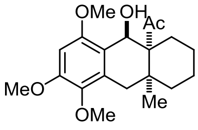

83

***β*-hydroxyl ketone 83** (35 mg) was prepared according to general procedure D from **76** (0.4 mmol) and **13** in 25% yield, the reaction time was 4 h under λ_{max} = 366 nm light. The products were isolated through silica gel column chromatography (5% to 8% ethyl acetate-petroleum ether) as a colorless oil: R_f = 0.44 (30% ethyl acetate-petroleum ether); ^{1}H NMR (500 MHz, $CDCl_3$) δ 6.42 (s, 1H), 5.03 (s, 1H), 3.88 (s, 6H), 3.74 (s, 3H), 2.83 (br, 1H), 2.50 (br, 1H), 2.21 (s, 3H), 1.84 (br, 1H), 1.55 (s, 4H), 1.41 – 1.09 (m, 6H) ppm; ^{13}C NMR (125 MHz, $CDCl_3$) δ 210.5, 154.2, 152.1, 140.2, 130.4, 117.3, 94.3, 69.0, 60.2, 56.5, 55.9, 55.5, 34.9, 34.0, 28.0, 24.5, 21.8 (2C), 20.9 (2C) ppm; IR ν_{max} 3472, 2934, 2861, 2251, 1703, 1598, 1459, 1327, 1233, 1202, 1089, 912, 728 cm^{-1}; HRMS–EI (*m*/*z*): $[M]^+$ calcd for $C_{20}H_{28}O_5$, 348.1937; found, 348.1942.

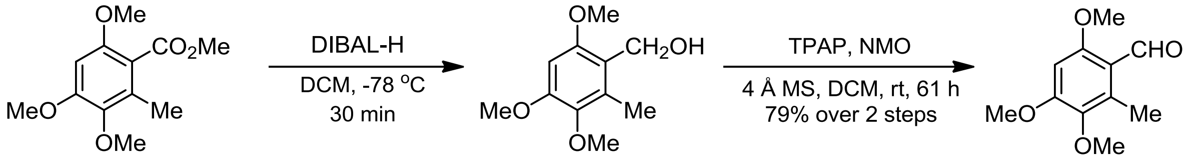

S5 **S6** **13**

Supplementary Figure 98. Synthesis of aromatic aldehyde **13**.

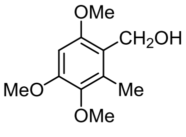

S6

Benzyl alcohol S6: To a stirred solution of **S5**[12] (2.0 g, 8.32 mmol, 1.0 equiv.) in anhydrous dichloromethane (30 mL) was added diisobutylaluminium hydride (1.5 M solution in toluene, 12.8 mL, 19.15 mmol, 2.3 equiv.) at -78 °C under N_2, the resulting mixture was stirred at -78 °C for 30 min. The reaction mixture was quenched with saturated Rochelle salt at -78 °C, then the mixture was removed to stir at rt for 1 h. The resulting mixture was extracted with ethyl acetate (3×40 mL), the combined organic layer was washed with brine (2×30 mL) and dried over anhydrous sodium sulfate. The dried solution was filtered and the filtrate was concentrated under vacuum to give crude product **S6** as a white solid (1.65 g, 93% crude yield). The residue was used directly in the next step without purification. R_f = 0.26 (30% ethyl acetate-petroleum ether); m.p. 104 – 106 °C; ^{1}H NMR (400 MHz, $CDCl_3$) δ 6.37 (s, 1H), 4.66 (s, 2H), 3.86 (s, 3H), 3.82 (s, 3H), 3.71 (s, 3H), 2.30 (s, 3H), 2.15 (s, 1H) ppm; ^{13}C NMR (100 MHz, $CDCl_3$) δ 154.4, 152.6, 141.1, 131.8, 119.7, 94.3, 60.4, 57.0, 55.79, 55.75, 11.6 ppm; IR ν_{max} 3382, 2961, 1593, 1471, 1330, 1232, 1197, 1085, 992, 805, 739 cm^{-1}; MS–EI (*m*/*z*): $[M]^+$ calcd for $C_{11}H_{16}O_4$, 212.1; found, 212.

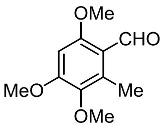

13

Aromatic aldehyde 13: 4 Å molecular sieve (996 mg) in a flask was pre-activated via alcohol blast burner for 10 min under vacuum, after cooling to rt, the flask was replaced with N_2 three times, then **S5** (996 mg, 4.69 mmol, 1.0 equiv.), tetrapropylammonium perruth-enate

(16.5 mg, 0.047 mmol, 0.01 equiv.) and 4-methylmorpholine N-oxide (1.10 g, 9.38 mmol, 2.0 equiv.) were added sequentially to the above flask under N_2. The mixture was diluted with anhydrous dichloromethane (30 mL), the resulting mixture was stirred vigorously at rt for 61 h. The reaction mixture was filtered through a short silica column and rinsed with ethyl acetate, the combined filtrate was concentrated under vacuum. The residue was purified by silica gel column chromatography (5% to 10% ethyl acetate-petroleum ether) to give **13** as a white solid (834 mg, 85%). R_f = 0.51 (30% ethyl acetate-petroleum ether); m.p. 99 – 101 ^{o}C; ^{1}H NMR (400 MHz, $CDCl_3$) δ 10.46 (s, 1H), 6.35 (s, 1H), 3.93 (s, 3H), 3.88 (s, 3H), 3.69 (s, 3H), 2.50 (s, 3H) ppm; ^{13}C NMR (100 MHz, $CDCl_3$) δ 190.7, 161.4, 158.0, 141.2, 135.5, 116.7, 93.4, 60.5, 56.0, 55.7, 12.9 ppm; IR ν_{max} 2945, 2842, 1663, 1588, 1438, 1327, 1242, 1207, 1074, 996, 834, 760 cm^{-1}; HRMS–EI (*m*/*z*): $[M]^+$ calcd for $C_{11}H_{14}O_4$, 210.0892; found, 210.0891.

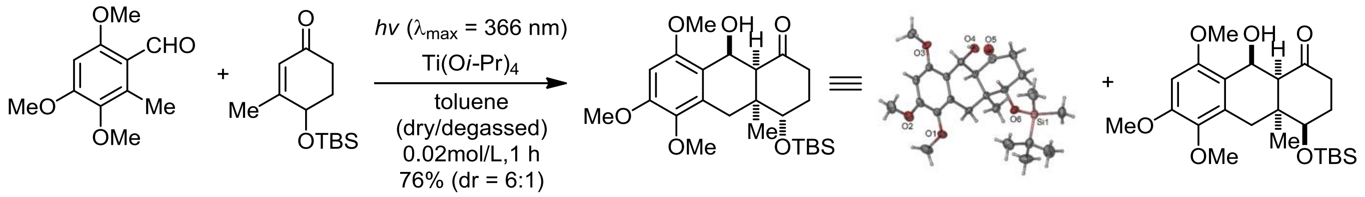

Supplementary Figure 99. Titanium(IV)-promoted PEDA reaction for synthesis of *β*-hydroxyl ketone **88** and its' epimer **88a**.

88 was obtained according to general procedure B. Specific experimental operation process: To a solution of **13** (1.513 g, 7.20 mmol, 1.0 equiv.) and **17** (2.60 g, 10.81 mmol, 1.5 equiv.) in anhydrous and degassed toluene (360 mL, 0.02 M) in a round-bottom flask sealed with rubber plug was added titanium(IV) isopropoxide (2.56 mL, 8.64 mmol, 1.2 equiv.) under N_2, after homogeneous mixing, the solution was divided into 24 parallel reactions in 24 quartz tubes. 12 parallel reactions were conducted with 12 quartz tubes once, the solution was photolyzed at rt in a Rayonet chamber reactor (16 lamps) at λ_{max} = 366 nm for 1 h. After the above 12 parallel reactions were over, the reaction mixture was poured into saturated sodium bicarbonate (150 mL) and stirred over 30 min. Then another 12 parallel reactions were conducted for another 1 h, the reaction mixture was also poured into saturated sodium bicarbonate (150 mL) and stirred over 30 min. All 24 parallel reactions were combined for subsequent workup. The above mixture was extracted with ethyl acetate (3×150 mL), the combined organic layer was washed with brine (2×200 mL) and dried over anhydrous sodium sulfate, the dried solution was filtered and the filtrate was concentrated under vacuum. The residue was purified by silica gel column chromatography (5% to 25% ethyl acetate-petroleum ether) to give **88** as light yellow solid (2.09 g, 64%) and **88a** as a dark green oil (390 mg, 12%).

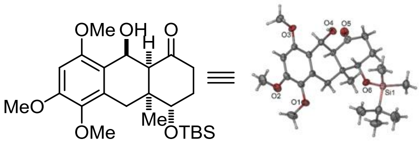

88 CCDC 1482901

***β*-hydroxyl ketone 88:** R_f = 0.28 (20% ethyl acetate-petroleum ether); m.p. 130 – 132 °C; Recrystallization of **88** from acetone/hexane (v/v, 1:1) in the refrigerator, CCDC 1482901. ^{1}H NMR (500 MHz, $CDCl_3$) δ 6.41 (s, 1H), 5.09 (d, J = 5.2 Hz, 1H), 4.06 (dd, J = 7.5, 4.2 Hz, 1H), 3.87 (s, 3H), 3.85 (s, 3H), 3.71 (s, 3H), 3.39 (s, 1H), 3.00 (d, J = 17.1 Hz, 1H), 2.78 (d, J = 5.8 Hz, 1H), 2.66 – 2.53 (m, 2H), 2.30 (d, J = 17.1 Hz, 1H), 2.16 – 2.09 (m, 1H), 1.97 – 1.87 (m, 1H), 1.01 (s, 3H), 0.92 (s, 9H), 0.02 (s, 3H), -0.03 (s, 3H) ppm; ^{13}C NMR (125 MHz, $CDCl_3$) δ 213.7, 153.9, 152.3, 140.2, 129.9, 118.2, 95.0, 70.8, 64.3, 60.2, 56.4, 55.9 55.8, 40.9, 38.6, 31.5, 29.7, 25.8 (3C), 22.5, 18.0, -4.2, -5.1 ppm; IR ν_{max} 3539, 2931, 2855, 1698, 1598, 1463, 1323, 1048, 1012, 836, 773 cm^{-1}; HRMS–EI (*m*/*z*): $[M]^+$ calcd for $C_{24}H_{38}O_6Si$, 450.2438; found, 450.2440.

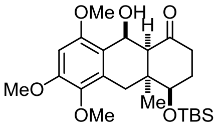

88a

***β*-hydroxyl ketone 88a:** R_f = 0.11 (20% ethyl acetate-petroleum ether); ^{1}H NMR (400 MHz, $CDCl_3$) δ 6.38 (s, 1H), 5.24 (dd, J = 8.3, 4.4 Hz, 1H), 4.35 (d, J = 8.4 Hz, 1H), 3.86 (s, 3H), 3.82 (s, 3H), 3.78 (t, J = 3.5 Hz, 1H), 3.73 (s, 3H), 3.09 (d, J = 16.7 Hz, 1H), 2.82 – 2.70 (m, 1H), 2.58 (d, J = 16.7 Hz, 1H), 2.45 – 2.36 (m, 2H), 2.30 – 2.19 (m, 1H), 2.08 – 1.97 (m, 1H), 1.07 (s, 3H), 0.79 (s, 9H), 0.14 (s, 3H), 0.04 (s, 3H) ppm; ^{13}C NMR (100 MHz, $CDCl_3$) δ 212.6, 153.8, 152.4, 140.0, 130.9, 118.9, 95.3, 74.8, 62.6, 60.3 (d, J = 2.2 Hz), 58.3 (d, J = 1.2 Hz), 56.3 (d, J = 2.4 Hz), 56.0 (d, J = 2.5 Hz), 40.6, 36.1, 31.4, 29.3, 28.4, 25.6 (3C), 17.9, -4.6, -5.1.; IR ν_{max} 3402, 2933, 2855, 1700, 1598, 1462, 1328, 1233, 1045, 972, 835, 774 cm^{-1}; HRMS–EI (*m*/*z*): $[M]^+$ calcd for $C_{24}H_{38}O_6Si$, 450.2438; found, 450.2434.

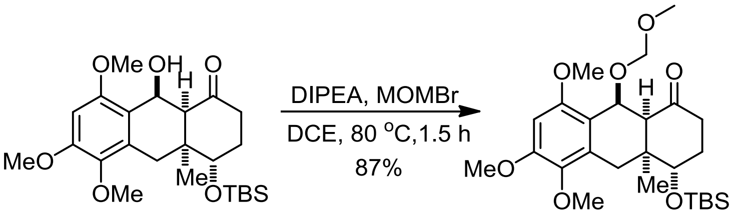

88 89

Supplementary Figure 100. Synthesis of ketone **89**.

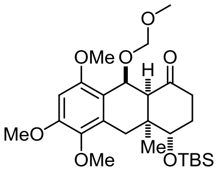

89

Ketone 89: To a stirred solution of **88** (1.816 g, 4.03 mmol, 1.0 equiv.) in anhydrous 1,2-dichloroethane (25 mL) was added N,N-diisopropylethylamine (4.0 mL, 24.18 mmol, 6.0 equiv.) quickly at rt under N_2, followed by addition of methoxymethyl bromide (1.65 mL, 20.15 mmol, 5.0 equiv.), the resulting mixture was vigorously stirred at 80 °C for 1.5 h. After cooling to rt, the reaction mixture was quenched with saturated sodium bicarbonate (30 mL). The resulting mixture was extracted with ethyl acetate (3×40 mL), the combined organic layer was washed with

brine (1×80 mL) and dried over anhydrous sodium sulfate. The dried solution was filtered and the filtrate was concentrated under vacuum. The residue was purified by silica gel column chromatography (5% to 15% ethyl acetate-petroleum ether) to give **89** as a yellow oil (1.73 g, 87%). R_f = 0.5 (30% ethyl acetate-petroleum ether); ^{1}H NMR (400 MHz, $CDCl_3$) δ 6.34 (s, 1H), 5.39 (d, J = 4.0 Hz, 1H), 4.42 (d, J = 6.5 Hz, 1H), 4.31 (d, J = 6.5 Hz, 1H), 3.98 (dd, J = 7.8, 2.1 Hz, 1H), 3.88 (s, 3H), 3.79 (s, 3H), 3.72 (s, 3H), 3.20 (s, 3H), 2.88 (d, J = 14.3 Hz, 1H), 2.76 (d, J = 14.4 Hz, 1H), 2.57 – 2.37 (m, 2H), 2.30 – 2.20 (m, 1H), 2.09 (d, J = 3.9 Hz, 1H), 1.99 – 1.86 (m, 1H), 0.90 (s, 9H), 0.85 (s, 3H), 0.10 (s, 3H), 0.09 (s, 3H) ppm; ^{13}C NMR (100 MHz, $CDCl_3$) δ 213.4, 153.4, 153.2, 140.4, 132.7, 115.9, 94.0, 93.9, 75.4, 69.1, 60.9, 58.8, 56.0, 55.7 (2C), 39.5, 36.4, 32.8, 26.5, 26.0, 25.8 (3C), 18.1, -4.2, -4.9 ppm; IR ν_{max} 2951, 2890, 2856, 1704, 1600, 1491, 1463, 1335, 1236, 1087, 1050, 1024, 974, 835, 773, 735 cm^{-1}; HRMS–EI (*m/z*): $[M]^+$ calcd for $C_{26}H_{42}O_7Si$, 494.2700; found, 494.2699.

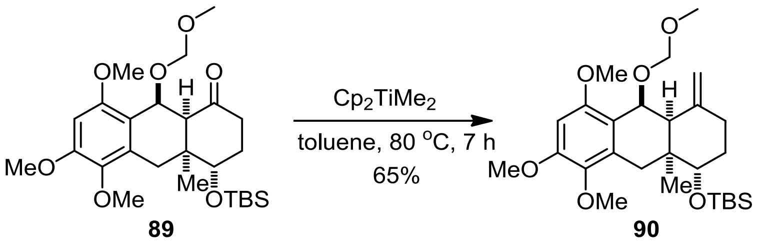

Supplementary Figure 101. Synthesis of methylene silyl ether **90**.

Preparation of petasis reagent:[13] To a stirred suspension of titanocene dichloride (3.0 g, 12.05 mmol, 1.0 equiv.) in anhydrous toluene (24 mL) was added methyllithium (1.6 M solution in diethyl ether, 17.32 mL, 27.7 mmol, 2.3 equiv.) dropwise at -5 °C under N_2, the resulting mixture was stirred at -5 °C for 1 h and the reaction mixture transformed from red precipitate into yellow suspension during this period. Then the mixture was quenched with dropwise addition of 6% aqueous ammonium chloride at -5 °C, the resulting mixture was extracted with diethyl ether (3×25 mL), the combined organic layer was washed with water (2×50 mL), brine (2×50 mL) and dried over anhydrous magnesium sulfate. The dried solution was filtered and the filtrate was concentrated under vacuum until only a small amount of toluene was remaining, the residue was protected under N_2. Then anhydrous tetrahydrofuran (2 mL) and anhydrous toluene (8 mL) were added sequentially to the above mixture. ^{1}H NMR assay indicated that dimethyltitanocene (11.86 mmol, yield: 98%) and the concentration is 0.9 mol/L. The reagent was stored in refrigerator.

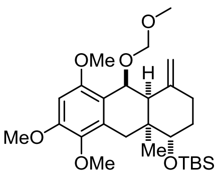

90

Methylene silyl ether 90: To a stirred solution of **89** (1.94 g, 3.92 mmol, 1.0 equiv.) in anhydrous toluene (20 mL) was added dimethyltitanocene (0.9 M solution in tetrahydrofuran/toluene, 13.06 mL, 11.76 mmol, 3.0 equiv.) at rt under N_2, the resulting mixture was vigorously stirred at 80 °C for 7 h. After cooling to rt, the reaction mixture

was diluted with petroleum ether (200 mL) to precipitate the Ti product, the resulting suspension was stirred for another 1 h. The orange precipitate was separated from the red filtrate using a pad of celite and rinsed with petroleum ether. The filtrate was concentrated under vacuum and the residue was purified by silica gel column chromatography (1% to 3% ethyl acetate-petroleum ether) to give **90** as a light yellow oil (1.25 g, 65%). R_f = 0.68 (30% ethyl acetate-petroleum ether); ^{1}H NMR (500 MHz, $CDCl_3$) δ 6.35 (s, 1H), 5.02 (d, J = 2.2 Hz, 1H), 4.86 (s, 1H), 4.75 (s, 1H), 4.57 (d, J = 6.2 Hz, 1H), 4.44 (d, J = 6.2 Hz, 1H), 4.15 (dd, J = 11.3, 4.1 Hz, 1H), 3.87 (s, 3H), 3.81 (s, 3H), 3.71 (s, 3H), 3.29 (s, 3H), 3.10 (d, J = 15.9 Hz, 1H), 2.64 (d, J = 16.0 Hz, 1H), 2.60 (d, J = 13.9 Hz, 1H) 2.31 (d, J = 13.4 Hz, 1H), 2.01 (s, 1H), 1.76 – 1.69 (m, 1H), 1.52 (ddd, J = 16.1, 12.9, 4.0 Hz, 1H), 0.94 (s, 9H), 0.93 (s, 3H), 0.09 (s, 3H), 0.08 (s, 3H) ppm; ^{13}C NMR (125 MHz, $CDCl_3$) δ 152.9, 152.7, 149.3, 140.6, 132.3, 119.3, 112.0, 94.6, 94.0, 78.6, 73.9, 60.7, 56.0, 55.9, 55.7, 55.1, 36.7, 34.9, 32.8, 31.6, 25.9 (3C), 22.2, 18.2, -3.7, -4.8 ppm; IR ν_{max} 2933, 1598, 1488, 1463, 1330, 1236, 1084, 1027, 886, 834, 772, 675 cm^{-1}; HRMS–EI (*m*/*z*): $[M]^+$ calcd for $C_{27}H_{44}O_6Si$, 492.2907; found, 492.2904.

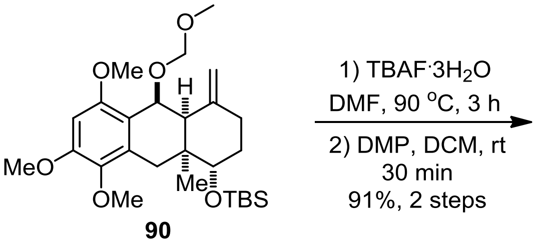

Supplementary Figure 102. Synthesis of methylene ketone **90-2**.

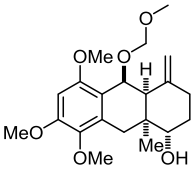
90-1

Alcohol 90-1: A stirred solution of **90** (1.075 g, 2.18 mmol, 1.0 equiv.) and tetrabutylammonium fluoride trihydrate (826 mg, 2.62 mmol, 1.2 equiv.) in anhydrous N,N-dimethylformamide (15 mL) under N_2 was heated at 90 °C for 3 h. After cooling to rt, the reaction mixture was diluted with saturated ammonium chloride (90 mL). The resulting mixture was extracted with diethyl ether (3×40 mL), the combined organic layer was washed sequentially with water (4×50 mL) and brine (3×50 mL). The washed solution was dried over anhydrous magnesium sulfate. The dried solution was filtered and the filtrate was concentrated under vacuum. The residue (**90-1**) was used directly in the next step without purification. R_f = 0.27 (30% ethyl acetate-petroleum ether); ^{1}H NMR (400 MHz, $CDCl_3$) δ 6.35 (s, 1H), 5.04 (d, J = 2.3 Hz, 1H), 4.88 (t, J = 2.0 Hz, 1H), 4.77 (s, 1H), 4.54 (d, J = 6.3 Hz, 1H), 4.40 (d, J = 6.3 Hz, 1H), 4.19 (dd, J = 11.7, 4.2 Hz, 1H), 3.88 (s, 3H), 3.81 (s, 3H), 3.72 (s, 3H), 3.28 (s, 3H), 3.19 (d, J = 15.7 Hz, 1H), 2.76 (d, J = 15.7 Hz, 1H), 2.70 – 2.59 (m, 1H), 2.35 (dt, J = 13.4, 3.3 Hz, 1H), 2.01 (s, 1H), 1.85 (ddd, J = 12.0, 7.5, 4.4 Hz, 1H), 1.53 (ddd, J = 25.4, 12.1, 4.1 Hz, 1H), 0.94 (s, 3H) ppm; ^{13}C NMR (100 MHz, $CDCl_3$) δ 152.9, 152.7, 148.8, 140.4, 131.5, 119.0, 112.6, 94.3, 93.8, 78.0, 73.6, 60.9, 55.9, 55.73, 55.71, 55.1, 36.2, 34.2, 32.7, 31.0, 21.5 ppm; IR ν_{max} 3450, 2934, 1597, 1488,

1461, 1329, 1233, 1204, 1081, 1026, 912, 731 cm^{-1}; HRMS–EI (*m*/*z*): $[M]^+$ calcd for $C_{21}H_{30}O_6$, 378.2042; found, 378.2045.

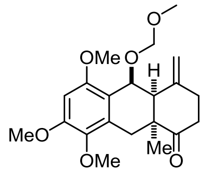

90-2

Methylene ketone 90-2: To a stirred solution of **90-1** (2.18 mmol, 1.0 equiv.) in anhydrous dichloromethane (15 mL) was added Dess-Martin periodinane (1.85 g, 4.36 mmol, 2.0 equiv.) at rt under N_2, after stirring for 2 min, 30 uL water (H_2O/DCM, 2 uL/1 mL) was added to the above mixture, the resulting suspension was stirred at rt for 30 min. Then the solvent was removed under vacuum, an aqueous solution of 1:1(v/v) 10% sodium thiosulfate /saturated sodium bicarbonate (20 mL) and ethyl acetate (30 mL) were added. After stirring for another 5 min, the above mixture was separated to obtain organic layer with a separatory funnel, the aqueous layer was extracted with ethyl acetate (2×30 mL), the combined organic layer was washed with brine (2×40 mL) and dried over anhydrous sodium sulfate. The dried solution was filtered and the filtrate was concentrated under vacuum. The residue was purified by silica gel column chromatography (5% to 15% ethyl acetate-petroleum ether) to give **90-2** as a light yellow oil (750 mg, 91% over 2 steps) which turned into solid in the refrigerator. R_f = 0.40 (30% ethyl acetate-petroleum ether); m.p. 53 – 55 °C; ^{1}H NMR (400 MHz, $CDCl_3$) δ 6.32 (s, 1H), 5.05 (d, J = 1.6 Hz, 1H), 5.02 (s, 1H), 4.91 (s, 1H), 4.41 (d, J = 6.4 Hz, 1H), 4.32 (d, J = 6.4 Hz, 1H), 3.85 (s, 3H), 3.78 (s, 3H), 3.73 (s, 3H), 3.60 (d, J = 17.2 Hz, 1H), 3.20 (s, 3H), 3.15 – 3.03 (m, 1H), 2.83 (d, J = 17.1 Hz, 1H), 2.66 – 2.46 (m, 3H), 2.33 (s, 1H), 1.20 (s, 3H) ppm; ^{13}C NMR (100 MHz, $CDCl_3$) δ 213.5, 152.9, 152.7, 146.5, 140.6, 131.3, 117.6, 113.7, 94.0, 93.7, 71.8, 60.6, 56.7, 55.9 (2C), 55.7, 44.0, 37.5, 32.2, 29.2, 29.0 ppm; IR ν_{max} 2926, 1700, 1596, 1489, 1462, 1335, 1235, 1203, 1084, 1018, 899, 812 cm^{-1}; HRMS–EI (*m*/*z*): $[M]^+$ calcd for $C_{21}H_{28}O_6$, 376.1886; found, 376.1890.

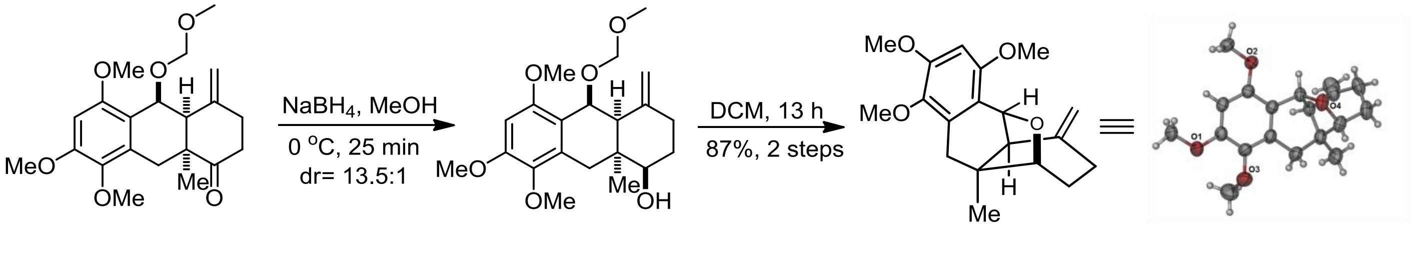

90-2 91 92 CCDC 1482900

Supplementary Figure 103. Synthesis of ether **92**.

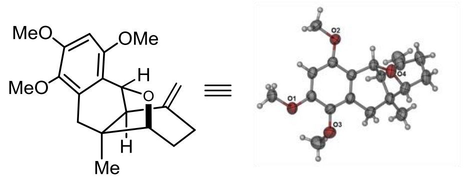

92 CCDC 1482900

Ether 92: To a stirred solution of **90-2** (750 mg, 1.99 mmol, 1.0 equiv.) in bottled methanol (15 mL) was added sodium borohydride (150 mg, 3.99 mmol, 2.0 equiv.) in portions at 0 °C, the resulting mixture was stirred at 0 °C for 25 min. The reaction mixture was quenched with addition of acetone (10 mL) at 0 °C, after stirring for another 5 min, the solvent was removed under vacuum.

The residue was diluted with water (15 mL) and ethyl acetate (20 mL), the resulting mixture was extracted with ethyl acetate (3×25 mL), the combined organic layer was washed with brine (2×35 mL) and dried over anhydrous sodium sulfate. The dried solution was filtered and the filtrate was concentrated under vacuum, the solvent was drained completely with oil pump. Then bottled dichloromethane (50 mL) was added to diluted the residue (**91**), the resulting mixture was stirred at rt for 13 h. The solvent was removed under vacuum and the residue was purified by silica gel column chromatography (0% to 10% ethyl acetate-petroleum ether) to give **92** as a colorless oil (552 mg, 87% over 2 steps) which turned into a white solid in the refrigerator. R_f = 0.55 (30% ethyl acetate-petroleum ether); m.p. 111 – 113 °C; Recrystallization of **92** from ethyl acetate/hexane (v/v, 1:3) in the refrigerator, CCDC 1482900. ^{1}H NMR (400 MHz, $CDCl_3$) δ 6.31 (s, 1H), 5.30 (s, 1H), 4.77 (t, J = 2.1 Hz, 1H), 4.62 (t, J = 2.3 Hz, 1H), 4.01 (d, J = 4.2 Hz, 1H), 3.85 (s, 3H), 3.78 (s, 3H), 3.76 (s, 3H), 2.99 (d, J = 18.6 Hz, 1H), 2.90 (d, J = 18.6 Hz, 1H), 2.66 – 2.53 (m, 1H), 2.34 (s, 1H), 2.20 (dd, J = 15.2, 6.3 Hz, 1H), 1.80 – 1.63 (m, 2H), 1.11 (s, 3H) ppm; ^{13}C NMR (100 MHz, $CDCl_3$) δ 152.1, 150.5, 147.1, 140.8, 130.7, 123.6, 109.3, 94.9, 84.9, 73.5, 59.9, 56.2, 55.9, 55.3, 42.1, 39.7, 28.6, 26.1, 20.5 ppm; IR ν_{max} 2932, 1598, 1491, 1462, 1430, 1333, 1232, 1203, 1083, 1050, 986, 890, 810, 747 cm^{-1}; HRMS–EI (*m*/*z*): $[M]^+$ calcd for $C_{19}H_{24}O_4$, 316.1675; found, 316.1673.

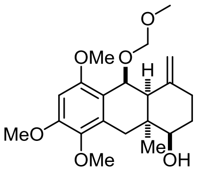

91

Alcohol 91: R_f = 0.31 (30% ethyl acetate-petroleum ether); ^{1}H NMR (400 MHz, $CDCl_3$) δ 6.35 (s, 1H), 5.10 (d, J = 2.4 Hz, 1H), 5.01 (d, J = 9.7 Hz, 1H), 4.95 (s, 1H), 4.83 (s, 1H), 4.67 (d, J = 6.5 Hz, 1H), 4.52 (d, J = 6.5 Hz, 1H), 3.88 (s, 3H), 3.82 (s, 3H), 3.72 (s, 3H), 3.52 (d, J = 10.4 Hz, 1H), 3.36 (d, J = 16.6Hz, 1H), 3.33 (s, 3H), 2.84 (d, J = 16.6 Hz, 1H), 2.72 – 2.60 (m, 1H), 2.19 (d, J = 14.0 Hz, 1H), 2.10 (s, 1H), 1.94 – 1.75 (m, 2H), 1.05 (s, 3H) ppm; ^{13}C NMR (100 MHz, $CDCl_3$) δ 153.1, 152.9, 148.2, 140.6, 132.7, 117.3, 112.7, 94.1, 93.5, 72.7, 71.6, 60.7, 56.1, 55.8, 55.7, 52.7, 36.5, 30.6, 30.0, 29.7, 27.4 ppm; HRMS–EI (*m*/*z*): $[M]^+$ calcd for $C_{21}H_{30}O_6$, 378.2042; found, 378.2044.

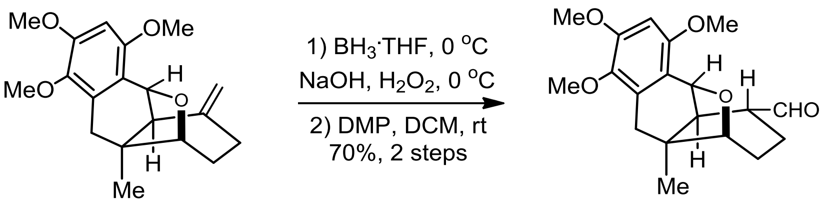

Supplementary Figure 104. Synthesis of aldehyde **93**.

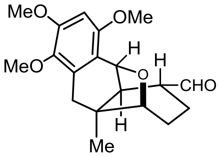

93

Aldehyde 93: To a stirred solution of **92** (220 mg, 0.695 mmol, 1.0 equiv.) in anhydrous tetrahydrofuran (8 mL) was added borane-tetrahydrofuran complex (1.0 M solutiom in tetrahydrofuran, 2.78 mL, 2.78 mmol, 4.0 equiv.) at 0 °C under N_2, the resulting mixture

was stirred at 0 °C for 1 h. Then methanol (1 mL) was added dropwise to quench this process at 0 °C, after stirring at 0 °C for 5 min, an aqueous sodium hydroxide (3.0 M in water, 0.93 mL, 4.0 equiv.) was added slowly to the above mixture at 0 °C, followed by dropwise addition of 30% hydrogen peroxide (0.93 mL), the resulting mixture was stirred at 0 °C for 1 h 40 min. Then the reaction mixture was quenched with saturated sodium sulfite, the resulting mixture was extracted with ethyl acetate (3×15 mL), the combined organic layer was washed with brine (2×25 mL) and dried over anhydrous sodium sulfate. The dried solution was filtered and the filtrate was concentrated under vacuum, the solvent was drained completely with oil pump. The residue (**92-1**) was used quickly for the next step without purification.

To a stirred solution of **92-1** (0.695 mmol, 1.0 equiv.) in anhydrous dichloromethane (8 mL) was added Dess-Martin periodinane (590 mg, 1.39 mmol, 2.0 equiv.) at rt under N_2, after stirring for 2 min, 16 uL water (H_2O/DCM, 2 uL/1 mL) was added to the above mixture, the resulting suspension was stirred at rt for 30 min. Then the solvent was removed under vacuum, an aqueous solution of 1:1(v/v) 10% sodium thiosulfate /saturated sodium bicarbonate (20 mL) and ethyl acetate (20 mL) were added. After stirring for another 5 min, the above mixture was separated to obtain organic layer with a separatory funnel, the aqueous layer was extracted with ethyl acetate (2×20 mL), the combined organic layer was washed with brine (2×30 mL) and dried over anhydrous sodium sulfate. The dried solution was filtered and the filtrate was concentrated under vacuum. The residue was purified by silica gel column chromatography (5% to 12% ethyl acetate-petroleum ether) to give **93** as a white foam. (163 mg, 70% over 2 steps). R_f = 0.57 (40% ethyl acetate-petroleum ether); ^{1}H NMR (400 MHz, $CDCl_3$) δ 9.78 (s, 1H), 6.31 (s, 1H), 5.29 (s, 1H), 3.94 (d, J = 4.2 Hz, 1H), 3.83 (s, 3H), 3.78 (s, 3H), 3.74 (s, 3H), 2.99 (d, J = 18.6 Hz, 1H), 2.77 (d, J = 18.6 Hz, 1H), 2.64 (dd, J = 8.7, 2.1 Hz, 1H), 2.49 (d, J = 2.2 Hz, 1H), 2.22 (dd, J = 14.2, 6.3 Hz, 1H), 2.04 – 1.88 (m, 1H), 1.79 – 1.56 (m, 2H), 1.02 (s, 3H) ppm; ^{13}C NMR (100 MHz, $CDCl_3$) δ 203.9, 152.2, 150.4, 140.7, 130.4, 123.3, 94.9, 84.9, 74.2, 59.9, 56.1, 55.9, 50.6, 45.6, 41.0, 40.4, 26.1, 21.2, 14.6 ppm; IR ν_{max} 2928, 1717, 1600, 1491, 1460, 1329, 1230, 1203, 1083, 1055, 967, 732 cm^{-1}; HRMS–EI (*m*/*z*): $[M]^+$ calcd for $C_{19}H_{24}O_5$, 332.1624; found, 332.1620.

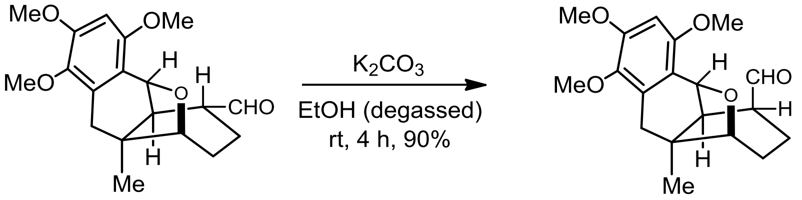

93 **94**

Supplementary Figure 105. Synthesis of aldehyde **94**.

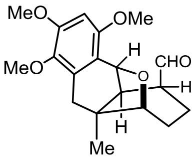

94

Aldehyde 94: To a flamed-dried flask containing **93** (163 mg, 0.49 mmol, 1.0 equiv.) and anhydrous potassium carbonate (34 mg, 0.245 mmol, 0.5 equiv.) was added degassed ethanol (5 mL) under N_2, the resulting suspension was vigorously stirred at rt for 4 h. Then the reaction mixture was quenched with saturated ammonium chloride (20 mL), the resulting mixture was extracted with dichloromethane (3×20 mL), the combined organic layer was washed with brine (2×30 mL) and dried over anhydrous sodium sulfate. The dried solution was filtered and the filtrate was concentrated under vacuum. The residue was purified by silica gel column chromatography (5% to 12% ethyl acetate-petroleum ether) to give **94** as a white foam (146.5 mg, 90%). R_f = 0.57 (40% ethyl acetate-petroleum ether); ^{1}H NMR (500 MHz, $CDCl_3$) δ 9.71 (s, 1H), 6.29 (s, 1H), 5.16 (s, 1H), 3.96 (d, J = 4.5 Hz, 1H), 3.82 (s, 3H), 3.75 (s, 3H), 3.74 (s, 3H), 3.03 (d, J = 18.5 Hz, 1H), 2.86 (d, J = 18.5 Hz, 1H), 2.76 (ddd, J = 13.0, 5.9, 2.0 Hz, 1H), 2.23 (d, J = 1.7 Hz, 1H), 2.05 – 1.94 (m, 1H), 1.81 – 1.66 (m, 3H), 1.26 (s, 3H) ppm; ^{13}C NMR (125 MHz, $CDCl_3$) δ 203.9, 152.2, 150.5, 140.7, 130.3, 123.4, 95.0, 84.7, 70.7, 59.9, 56.2, 55.9, 46.6, 45.0, 41.0, 40.1, 26.9, 19.5, 16.8 ppm; IR ν_{max} 2925, 2854, 1718, 1599, 1491, 1460, 1331, 1230, 1204, 1081, 1054, 984, 953, 757 cm^{-1}; HRMS–EI (*m*/*z*): $[M]^+$ calcd for $C_{19}H_{24}O_5$, 332.1624; found, 332.1621.

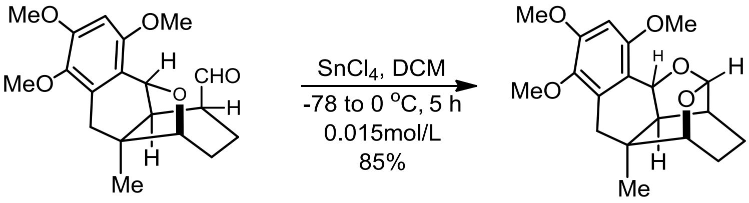

Supplementary Figure 106. Synthesis of acetal **95**.

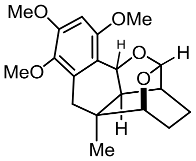

95

Acetal 95: To a stirred solution of **94** (50.6 mg, 0.152 mmol, 1.0 equiv.) in anhydrous dichloromethane (10.2 mL, 0.015mo/L) was added tin tetrachloride (1.0 M solution in heptane, 15.2 uL, 0.0152 mmol, 0.1 equiv.) dropwise at -78 °C under N_2, the resulting mixture was stirred at -78 °C for 1 h, then the reaction mixture was removed to stir at 0 °C for another 4 h. The reaction was quenched with saturated sodium bicarbonate (15 mL) at 0 °C, the resulting mixture was extracted with dichloromethane (4×15 mL), the combined organic layer was washed with brine (1×30 mL) and dried over anhydrous sodium sulfate. The dried solution was filtered and the filtrate was concentrated under vacuum. The residue was purified by silica gel column chromatography (6% to 12% ethyl acetate-petroleum ether) to give **95** as a light yellow oil (43 mg, 85%). R_f = 0.57 (40% ethyl acetate-petroleum ether); ^{1}H NMR (400 MHz, $CDCl_3$) δ 6.38 (s, 1H), 5.37 (d, J = 3.7 Hz, 1H), 5.21 (d, J = 2.8 Hz, 1H), 3.88 (s, 3H), 3.82 (s, 3H), 3.71 (s, 3H), 3.50 (d, J = 3.7 Hz, 1H), 2.92 (d, J = 15.3 Hz, 1H), 2.83 (d, J = 15.3 Hz, 1H), 2.23 – 2.18 (m, 1H), 1.98 – 1.72 (m, 4H), 1.69 (t, J = 3.2 Hz, 1H), 1.03 (s, 3H) ppm; ^{13}C NMR

(100 MHz, $CDCl_3$) δ 154.1, 153.1, 140.2, 132.9, 114.5, 100.4, 94.6, 73.9, 71.1, 60.8, 56.2, 55.8, 46.4, 37.9, 36.5, 31.4, 27.9, 20.5, 15.7 ppm; IR ν_{max} 2929, 2867, 1731, 1601, 1492, 1461, 1331, 1237, 1205, 1125, 1080, 1036, 949, 817, 733 cm^{-1}; HRMS–EI (*m/z*): $[M]^+$ calcd for $C_{19}H_{24}O_5$, 332.1624; found, 332.1620.

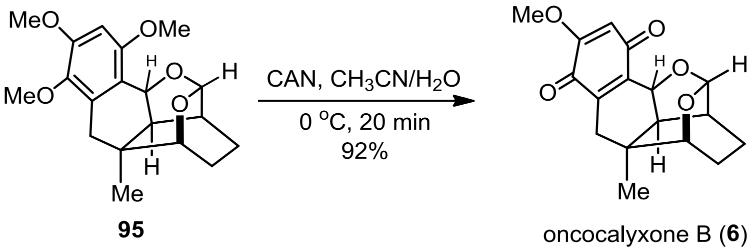

Supplementary Figure 107. Total **S**ynthesis of Oncocalyxone B (**6**).

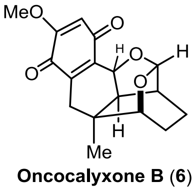

Oncocalyxone B (6): To a stirred solution of **95** (43 mg, 0.129 mmol, 1.0 equiv.) in acetonitrile (4 mL) was added a solution of ceric ammonium nitrate (248 mg, 0.453 mmol, 3.5 equiv.) in water (2 mL) dropwise at 0 °C, the resulting mixture was stirred at 0 °C for 20 min. Then the reaction mixture was diluted with water (10 mL) and dichloromethane (15 mL), the above mixture was separated to obtain organic layer with a separatory funnel, the aqueous layer was extracted with dichloromethane (3×20 mL), the combined organic layer was washed with brine (2×30 mL) and dried over anhydrous sodium sulfate. The dried solution was filtered and the filtrate was concentrated under vacuum. The residue was purified by silica gel column chromatography (10% to 18% ethyl acetate-petroleum ether) to give **oncocalyxone B** (**6**) as a amorphous light yellow solid (36 mg, 92%). R_f = 0.21 (30% ethyl acetate-petroleum ether); ^{1}H NMR (500 MHz, $CDCl_3$) δ 5.90 (s, 1H), 5.34 (d, J = 3.6 Hz, 1H), 4.96 (d, J = 3.0 Hz, 1H), 3.80 (s, 3H), 3.49 (d, J = 2.6 Hz, 1H), 2.73 (d, J = 17.6 Hz, 1H), 2.56 (d, J = 17.6 Hz, 1H), 2.19 (m, 1H), 1.96 – 1.67 (m, 5H), 1.02 (s, 3H) ppm; ^{13}C NMR (125 MHz, $CDCl_3$) δ 185.1, 181.2, 158.7, 143.1, 137.3, 106.9, 100.5, 74.2, 68.5, 56.2, 46.5, 37.8, 37.0, 31.4, 28.3, 20.4, 15.5 ppm; IR ν_{max} 2935, 1677, 1654, 1627, 1603, 1226, 1200, 1034, 941, 840, 784 cm^{-1}; HRMS–EI (*m/z*): $[M]^+$ calcd for $C_{17}H_{18}O_5$, 302.1154; found, 302.1152.

Supplementary Table 8.

Comparison of ^{1}H NMR spectroscopic data of natural and synthetic oncocalyxone B

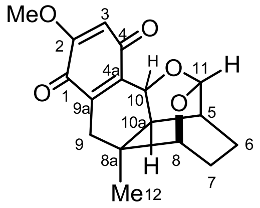

Oncocalyxone B

**position**	**natural**<br>δ ^{1}H [ppm; mult; *J* (Hz)]<br>200 MHz	**synthetic**<br>δ ^{1}H [ppm; mult; *J* (Hz)]<br>500 MHz	**deviation**<br>(natural–synthetic)<br>Δδ (ppm)
1			
2			
3	5.90; s	5.90; s	
4			
4a			
5	2.2; m	2.2; m	
6	1.8; m	1.8; m	
7	1.8; m	1.8; m	
8	3.49; br s	3.49; d; 2.6	
8a			
9 (H-9a)	2.70; d; 17.6	2.73; d; 17.6	-0.03
9 (H-9e)	2.60; d; 17.6	2.56; d; 17.6	0.04
9a			
10	4.96; d; 3.0	4.96; d; 3.0	
10a	1.8; m	1.8; m	
11	5.34; d; 3.6	5.34; d; 3.6	
12	1.02; s	1.02; s	
MeO-2	3.80; s	3.80; s	

Supplementary Table 9

Comparison of ^{13}C NMR spectroscopic data of natural and synthetic oncocalyxone B

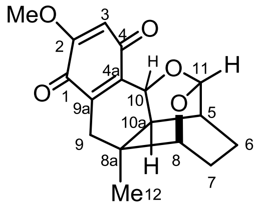

Oncocalyxone B

**position**	**natural** δ ^{13}C [ppm] 50 MHz	**synthetic** δ ^{13}C [ppm] 125 MHz	**deviation** (natural–synthetic) Δδ (ppm)
1	181.2	181.2	
2	158.7	158.7	
3	106.9	106.9	
4	185.1	185.1	
4a	143.1	143.1	
5	37.8	37.8	
6	20.4	20.4	
7	15.5	15.5	
8	74.2	74.2	
8a	37.0	37.0	
9	31.4	31.4	
9a	137.3	137.3	
10	68.5	68.5	
10a	46.4	46.5	-0.1
11	100.5	100.5	
12	28.3	28.3	
MeO-2	56.2	56.2	

HMBC of Oncocalyxone B was attached in SI (**Supplementary Figure 83**). After carefully analyzing the HMBC spectrum, we think that chemical shift of C4a and C9a need to swap. The carbon at 137.3 ppm has an HMBC cross peak with H3, which is only possible for C4a, not C9a. Therefore, C4a should have chemical shift of 137.3ppm and the adjacent C9a have chemical shift of 143.1ppm. An swapped comparison of ^{13}C NMR spectroscopic data of natural and synthetic oncocalyxone B was as follows: the swapped chemical shifts of C4a and C9a were showed in bold.

Supplementary Table 10

Comparison of ^{13}C NMR spectroscopic data of natural and synthetic oncocalyxone B

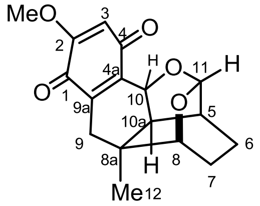

Oncocalyxone B

**position**	**natural** δ ^{13}C [ppm] 50 MHz	**synthetic** δ ^{13}C [ppm] 125 MHz	**deviation** (natural–synthetic) Δδ (ppm)
1	181.2	181.2	
2	158.7	158.7	
3	106.9	106.9	
4	185.1	185.1	
**4a**	**137.3**	**137.3**	
5	37.8	37.8	
6	20.4	20.4	
7	15.5	15.5	
8	74.2	74.2	
8a	37.0	37.0	
9	31.4	31.4	
**9a**	**143.1**	**143.1**	
10	68.5	68.5	
10a	46.4	46.5	-0.1
11	100.5	100.5	
12	28.3	28.3	
MeO-2	56.2	56.2	

Supplementary References

1. Still, W. C., Kahn, M. & Mitra, A. Rapid chromatographic technique for preparative separations with moderate resolution. *J. Org. Chem.* **43**, 2923-2925 (1978).
2. Azadi-Ardakani, M. & Wallace, T. W. 3,6-Dimethoxybenzocyclobutenone: a reagent for quinone synthesis. *Tetrahedron* **44**, 5939-5952 (1988).
3. Uttaro, J. P., Audran, G., Galano, J. M. & Monti, H. A stereocontrolled approach towards highly oxygenated taxane C and CD-ring precursors. *Tetrahedron Lett.* **43**, 2757-2760 (2002).
4. Rafferty, R. J. & Williams, R. M. Synthetic studies on the ambiguine family of alkaloids: construction of

the ABCD ring system. *Tetrahedron Lett.* **52**, 2037-2040 (2011).

5. Bauer, D. M., Rogge, A., Stolzer, L., Barner-Kowollik, C. & Fruk, L. Light induced DNA–protein conjugation. *Chem. Commun.* **49**, 8626-8628 (2013).
6. Nicolaou, K. C., Gray, D. L. F. & Tae, J. Total synthesis of hamigerans and analogues thereof. photochemical generation and Diels−Alder trapping of hydroxy-*o*-quinodimethanes. *J. Am. Chem. Soc.* **126**, 613-627 (2004).
7. Nicolaou, K. C.; Shi, L.; Lu, M.; Pattanayak, M. R., Shah, A. A., Ioannidou, H. A. & Lamani, M. Total synthesis of myceliothermophins C, D, and E. *Angew. Chem.*, *Int. Ed.* **53**, 10970-10974 (2014).
8. Lifchits, O., Mahlau, M., Reisinger, C. M., Lee, A., Fares, C., Polyak, I., Gopakumar, G., Thiel, W. & List, B. The cinchona primary amine-catalyzed asymmetric epoxidation and hydroperoxidation of *α,β*-unsaturated carbonyl compounds with hydrogen peroxide. *J. Am. Chem. Soc.* **135**, 6677-6693 (2013),.
9. Stanetty, P. & Mihovilovic, M. D. A novel hetero-Diels-Alder approach towards perhydro quinolinones bearing an angular methyl group. *Tetrahedron* **54**, 875-894 (1998).
10. Sum, F. W. & Weiler, L. Stereoselective synthesis of *β*-substituted *α,β*-unsaturated esters by dialkylcuprate coupling to the eno1 phosphate of *β*-keto esters. *Can. J. Chem*. **57**, 1431-1441 (1979).
11. Kamijo, S. & Dudley, G. B. Tandem nucleophilic addition/fragmentation reactions and synthetic versatility of vinylogous acyl triflates. *J. Am. Chem. Soc.* **128**, 6499-6507 (2006).
12. Tatsuta, K., Fukuda, T., Ishimori, T., Yachi, R., Yoshida, S., Hashimoto, H. & Hosokawa, S. The first total synthesis of hibarimicinone, a potent v-Src tyrosine kinase inhibitor. *Tetrahedron Lett.* **53**, 422-425 (2012).
13. Blauvelt, M. L. & Howell, A. R. Synthesis of *epi*-oxetin via a serine-derived 2-methyleneoxetane. *J. Org. Chem*. **73**, 517-521 (2008).
